# Supplementary material for: Flexible Mixture Model Approaches That Accommodate Footprint Size Variability for Robust Detection of Balancing Selection
Source: Mol Biol Evol. 2020 Oct 4;37(11):3267–91. doi: 10.1093/molbev/msaa134 (PMC7820363; doi:10.1093/molbev/msaa134)
Supplement: msaa134_supplementary_data [file msaa134_supplementary_data.zip › BallerMix_final/mixture_rev3_nomark.pdf]

Flexible mixture model approaches that accommodate footprint size variability for robust detection of balancing selection

Xiaoheng Cheng[1,2] and Michael DeGiorgio*[3]

[1]Huck Institutes of Life Sciences, Pennsylvania State University, University Park, PA, USA
[2]Department of Biology, Pennsylvania State University, University Park, PA, USA
[3]Department of Computer and Electrical Engineering and Computer Science, Florida Atlantic University, Boca Raton, FL, USA

Abstract

Long-term balancing selection typically leaves narrow footprints of increased genetic diversity, and therefore most detection approaches only achieve optimal performances when sufficiently small genomic regions (*i.e.*, windows) are examined. Such methods are sensitive to window sizes and suffer substantial losses in power when windows are large. Here, we employ mixture models to construct a set of five composite likelihood ratio test statistics, which we collectively term B statistics. These statistics are agnostic to window sizes and can operate on diverse forms of input data. Through simulations, we show that they exhibit comparable power to the best-performing current methods, and retain substantially high power regardless of window sizes. They also display considerable robustness to high mutation rates and uneven recombination landscapes, as well as an array of other common confounding scenarios. Moreover, we applied a specific version of the B statistics, termed B_2, to a human population-genomic dataset and recovered many top candidates from prior studies, including the then-uncharacterized *STPG2* and *CCDC169-SOHLH2*, both of which are related to gamete functions. We further applied B_2 on a bonobo population-genomic dataset. In addition to the *MHC-DQ* genes, we uncovered several novel candidate genes, such as *KLRD1*, involved in viral defense, and *SCN9A*, associated with pain perception. Finally, we show that our methods can be extended to account for multi-allelic balancing selection, and integrated the set of statistics into open-source software named `BalLeRMix` for future applications by the scientific community.

Introduction

Balancing selection maintains polymorphism at selected genetic loci, and can operate through a variety of mechanisms (Charlesworth, 2006). In addition to overdominance (Charlesworth and Charlesworth, 2010), other processes such as sexual selection (Cho et al., 2006), periodical environmental shifts (Bergland et al., 2014), pleiotropy (Andrés, 2001; Mitchell-Olds et al., 2007), meiotic drive (Ubeda and Haig, 2004; Charlesworth and Charlesworth, 2010), and negative frequency-dependent selection (Charlesworth and Charlesworth, 2010) can also maintain diversity at underlying loci. Due to the increasing availability of population level genomic data, in which allele frequencies and genomic density of polymorphisms can be assessed in detail, there is an expanding interest in studying balancing selection and detecting its genomic footprints (*e.g.*, Andrés et al., 2009; Leffler et al., 2013; DeGiorgio et al., 2014; Gao et al., 2015; Hunter-Zinck and Clark, 2015; Sheehan and Song, 2016; Lonn et al., 2017; Sweeney et al., 2017; Guirao-Rico et al., 2017; Siewert and Voight, 2017, 2020; Bitarello et al., 2018; Ye et al., 2018; Cheng and DeGiorgio, 2019). However, despite multiple efforts to design statistics for identifying balanced loci (*e.g.*, DeGiorgio et al., 2014; Siewert and Voight, 2017, 2020; Bitarello et al., 2018; Cheng and DeGiorgio, 2019), performances of existing methods still leave room for improvement.

*mdegiorg@fau.edu

Early methods applied to this problem evaluated departures from neutral expectations of genetic diversity at a particular genomic region. For example, the Hudson-Kreitman-Aguadé (HKA) test (Hudson et al., 1987) uses a chi-square statistic to assess whether genomic regions have higher density of polymorphic sites when compared to a putative neutral genomic background. In contrast, Tajima's D (Tajima, 1989) measures the distortion of allele frequencies from the neutral site frequency spectrum (SFS) under a model with constant population size. However, these early approaches were not tailored for balancing selection and have limited power. Recently, novel and more powerful summary statistics (Siewert and Voight, 2017, 2020; Bitarello et al., 2018) and model-based approaches (DeGiorgio et al., 2014; Cheng and DeGiorgio, 2019) have been developed to specifically target regions under balancing selection. In general, the summary statistics capture deviations of allele frequencies from a putative equilibrium frequency of a balanced polymorphism. In particular, the non-central deviation statistic (Bitarello et al., 2018) adopts an assigned value as this putative equilibrium frequency, whereas the β and $\beta^{(2)}$ statistics of Siewert and Voight (2017, 2020) use the frequency of the central polymorphic site instead. On the other hand, the T statistics of DeGiorgio et al. (2014) and Cheng and DeGiorgio (2019) compare the composite likelihood of the data under an explicit coalescent model of long-term balancing selection (Hudson et al., 1987; Hudson and Kaplan, 1988) to the composite likelihood under the genome-wide distribution of variation, which is taken as neutral.

Nevertheless, all extant approaches are limited by their sensitivity to the size of the region that the statistics are computed on (hereafter referred to as the "window size"). Because the footprints of long-term balancing selection are typically narrow (Hudson and Kaplan, 1988; Charlesworth, 2006), small windows with fixed size comparable to that of the theoretical footprint based on a genome-wide recombination rate estimate are commonly used in practice, especially for summary statistics. However, such small fixed window sizes not only lead to increased noise in the estimation of each statistic, but also render the statistic incapable of adapting to varying footprint sizes across the genome due to factors such as the uneven recombination landscape (Smukowski and Noor, 2011). Though adopting a larger window may reduce noise, true signals will likely be overwhelmed by the surrounding neutral regions, diminishing method power as shown by Cheng and DeGiorgio (2019). Available model-based approaches (DeGiorgio et al., 2014; Cheng and DeGiorgio, 2019) could have been made robust to window sizes if they instead adopted the SFS expected under a neutrally-evolving population of constant size as the null hypothesis, because their model of balancing selection for the alternative hypothesis converges to this constant-size neutral model for large recombination rates. However, this neutral model does not account for demographic factors that can impact the genome-wide distribution of allele frequencies, such as population size changes. To guard against such demographic influences, the model-based T_1 and T_2 statistics (DeGiorgio et al., 2014; Cheng and DeGiorgio, 2019) employ the genome-wide SFS instead, compromising the robustness against large windows. Moreover, Cheng and DeGiorgio (2019) showed that although the power of the T_2 statistic decays much slower than other approaches as window size increases, the loss of power is still substantial.

In this article, we describe a set of composite likelihood ratio test statistics that are based on a mixture model (Figures 1A and B) that integrates both the genome-wide level of variation and the enrichment of sites with allele frequencies close to the equilibrium allele frequency of long-term balancing selection. Note that the latter has been successfully captured by the summary statistics β (Siewert and Voight, 2017, 2020) and NCD (Bitarello et al., 2018). This framework of nested models allows for robust and flexible detection of balancing selection that can augment the size of genomic regions considered in each test to best fit the data. Dependent on the types of data available, we propose a set of five likelihood ratio test statistics termed B_2, $B_{2,\text{MAF}}$, B_1, B_0, and $B_{0,\text{MAF}}$, which respectively accommodate data with substitutions and derived (B_2) or minor ($B_{2,\text{MAF}}$) allele frequency polymorphisms, with substitutions and polymorphisms with unknown allele frequency (B_1), and with derived (B_0) or minor ($B_{0,\text{MAF}}$) allele frequency polymorphisms only. We comprehensively evaluated their performances under an array of diverse simulated scenarios, including their powers for balancing selection with varying ages, distinct strengths and equilibrium frequencies, robustness against window sizes, and robustness against confounding factors such as demographic history, recombination rate variation, and mutation rate variation. We also compared

and discussed their performances with other leading approaches—namely HKA, β, β^*, $\beta^{(2)}$, NCD, T_1, and T_2. To gauge the performance of B statistics on empirical data, we re-examined contemporary human populations in the 1000 Genomes Project dataset (The 1000 Genomes Project Consortium, 2015) to uncover previously hypothesized candidates. Furthermore, we performed an exploratory whole-genome scan with B_2 on bonobo genomic data (Prado-Martinez et al., 2013) to probe for long-term balancing selection in the other close relative of humans. We further extended our framework to consider multi-allelic balancing selection, and examined the performances of extant methods on cases of multi-locus balancing selection. Lastly, we developed the software `BalLeRMix` (BALancing selection LikElihood Ratio MIXture models) to implement these novel tests for the convenience of the scientific community.

Model Description

A classical footprint of balancing selection is the increase in the proportion of sites with moderate allele frequencies that are close to the equilibrium frequency at the balanced locus (Kaplan et al., 1988; Siewert and Voight, 2017). Previous modeling attempts (Kaplan et al., 1988; Song and Steinrücken, 2012; DeGiorgio et al., 2014; Cheng and DeGiorgio, 2019) primarily focused on delineating the underlying population-genetic processes, such as through coalescent or diffusion theory. Though these models are able to capture the distortion in the SFS resulting from balancing selection, their intricate mathematical formulations bring challenges to further model extensions to more complicated scenarios as well as the associated computations. As an alternative, it may be appealing to model the effect of balancing selection through statistical approximations of the expected features in the data.

Based on this idea, for a locus under balancing selection that is maintaining a pair of allelic classes, we can approximate the process of observing k_0 copies of the selected allele balanced at equilibrium frequency $x \in (0,1)$ in n samples, as following a binomial sampling process with n trials and a success rate x. For a bi-allelic neutral site that is linked to this selected locus, we assume that the k derived alleles observed from the n samples at this neutral site are all on the same haplotype with the k_0 selected alleles balanced at frequency x. That is, we assume $k = k_0$ and consider the k derived alleles on the neutral site as surrogates for the balanced alleles of the allelic class with which they are fixed. Therefore, when these two sites are in complete linkage, k can also be considered as binomially distributed with n trials and a success rate x. Meanwhile, for a neutral site not linked to this selected locus, we assume that k follows the distribution expected by the genome-wide SFS. Taken together, the probability $P_n(k)$ of observing k derived alleles out of n sampled alleles at a neutral site can be written as

$$\begin{aligned} P_n(k) = {} & \mathbb{P}[\text{Completely linked to the selected locus}] \cdot \mathbb{P}[k = k_0 \text{ out of } n \text{ binomially sampled with rate } x] \\ & + \mathbb{P}[\text{Not completely linked to the selected locus}] \cdot \mathbb{P}[k \text{ out of } n \text{ observed in the genome}]. \end{aligned}$$

Alternatively, this integration of two conditional probabilities can also be viewed as a mixture model, in which the two mixing components represent probabilities under balancing selection and neutrality (based on the genome-wide empirical distribution), with their respective mixing proportions α and $1-\alpha$ representing the probabilities of being completely linked to the selected locus or not, respectively. To approximate α, we chose to consider the exponential decay function, which has been adopted as a proxy for linkage disequilibrium (*e.g.* Nielsen et al., 2005; Moorjani et al., 2011; Loh et al., 2013). To accommodate the varying rates of linkage decay, we introduce a free parameter $A > 0$ for the statistic to optimize over, which essentially determines the size of the footprint of balancing selection, with smaller values of A having wider footprints than larger values. Hence, for a neutral site d recombination units away from the selected locus, the probability that it is linked to the selected locus can be approximated by

$$\mathbb{P}[\text{Completely linked to the selected locus}] = \alpha_A(d) = e^{-Ad}.$$

Therefore, for a neutral site d recombination units away from the selected locus, we approximate the probability mass function for sampling k derived alleles out of n sampled alleles as

$$f_{n,x,A}(k,d) = \alpha_A(d) \cdot h_{n,x}(k) + [1 - \alpha_A(d)] \cdot g_n(k),$$

where $h_{n,x}(k)$ denotes the normalized binomial probability of sampling k successes out of n trials with success rate x, and $g_n(k)$ is the normalized genome-wide SFS denoting the proportion of sites with k derived alleles observed out of n sampled alleles. This formulation also applies when k represents the number of minor allele copies, for situations in which the ancestral allele cannot be polarized with an outgroup. See subsequent subsection for precise definitions of normalized $h_{n,x}(k)$ and $g_n(k)$.

Note that although we constructed this mixture-model framework by combining conditional probabilities of the derived alleles at a neutral site to be on the same haplotype with one of the two balanced allele classes, the interpretation of the mixing weight $\alpha_A(d)$ is in effect not constrained to linkage and recombination. Other factors that can affect the local SFS, such as the accumulation of low-frequency mutations, can be accounted for by incorporating the genome-wide SFS as well. Although these factors can also vary by recombination distance, we formulate $\alpha_A(d)$ based on the properties of linkage decay alone to simplify our model.

In the following subsections, we describe a set of composite likelihood ratio statistics (B_2, $B_{2,\text{MAF}}$, B_1, B_0, and $B_{0,\text{MAF}}$) constructed based on this mixture model approach for identifying loci undergoing bi-allelic balancing selection. We also extended this framework to consider multi-allelic balancing selection, and describe these models in *Supplementary Note 1*. Note that all the composite likelihood ratio statistics considered here assume that balancing selection is acting on a single locus. This set of composite likelihood ratio statistics have been implemented in the open-source software package `BalLeRMix`, which is available at `https://github.com/bioXiaoheng/BalLeRMix/tree/master/software`.

Probability distributions given derived allele polymorphisms and substitutions

For n sampled alleles at an informative site (*i.e.*, polymorphism or substitution), when the ancestral state to each site can be confidently assigned, denote the number of derived alleles as k, $k = 1, 2, \ldots, n$. Let $\xi_n(k)$ be the total number of informative sites across the whole-genome with k derived alleles observed out of n sampled alleles. The probability of observing such a site is therefore

$$g_n^{(2)}(k) = \frac{\xi_n(k)}{\sum_{j=1}^{n} \xi_n(j)}.$$

When balancing selection maintains an equilibrium frequency of x on the site under selection, the outcomes of observing derived alleles on this site (out of n lineages) can be approximated by a binomial distribution of n trials with a success probability of x. Following this binomial model, the probability of observing the selected site with k observed derived alleles is

$$h_{n,x}^{(2)}(k) = \frac{\text{Bin}(k; n, x)}{\sum_{j=1}^{n} \text{Bin}(j; n, x)}.$$

Note the values of $g_n(k)$ and $h_{n,x}(k)$ are conditional on the number of sampled alleles n, and therefore our model requires that the sample size be made explicit at each informative site. Permitting the sample size to differ across sites is important, as missing genotype calls are often common in empirical studies, with sample sizes naturally varying across the genome.

For an informative site d recombination units away from the presumed site under selection, it can either be linked to the derived (with equilibrium frequency x) or ancestral (with equilibrium frequency $1-x$) haplotype under balancing selection, resulting in a bimodal distribution (Figure 1C). Therefore, the probability of observing k derived alleles out of n sampled alleles is

$$f_{n,x,A}^{(2)}(k,d) = \alpha_A(d)\left[\frac{1}{2}h_{n,x}^{(2)}(k) + \frac{1}{2}h_{n,1-x}^{(2)}(k)\right] + \left[1 - \alpha_A(d)\right]g_n^{(2)}(k),$$

where $\alpha_A(d) = \exp(-Ad)$ and where A is a model parameter that determines the size of the genomic footprint of balancing selection. When allele frequency information is unavailable at polymorphic sites, the probability of observing a polymorphic site ($k \neq n$) or substitution ($k = n$) would be

$$f^{(1)}_{n,x,A}(k,d) = f^{(2)}_{n,x,A}(n,d)\mathbf{1}_{\{k=n\}} + \left[1 - f^{(2)}_{n,x,A}(n,d)\right]\mathbf{1}_{\{k\neq n\}},$$

where $\mathbf{1}_{\{E\}}$ is a dummy variable that takes the value one if the expression E is true, and zero otherwise.

Similarly, when substitutions are not considered or are missing in the data (*i.e.*, only observe derived allele counts $k = 1, 2, \ldots, n-1$), the two mixing components can be normalized as

$$g^{(0)}_n(k) = \frac{\xi_n(k)}{\sum_{j=1}^{n-1}\xi_n(j)},$$

and

$$h^{(0)}_{n,x}(k) = \frac{\text{Bin}(k; n, x)}{\sum_{j=1}^{n-1}\text{Bin}(j; n, x)}.$$

The probability of observing a polymorphic site with k derived alleles out of n sampled alleles is then

$$f^{(0)}_{n,x,A}(k,d) = \alpha_A(d)\left[\frac{1}{2}h^{(0)}_{n,x}(k) + \frac{1}{2}h^{(0)}_{n,1-x}(k)\right] + \left[1 - \alpha_A(d)\right]g^{(0)}_n(k).$$

Probability distributions given minor allele polymorphisms and substitutions

When alleles cannot be confidently polarized, minor allele frequencies are often used instead. For informative sites with n sampled alleles, denote the minor allele count as k, $k = 0, 1, \ldots, \lfloor n/2 \rfloor$, and the total number of such sites in the genome as $\eta_n(k)$. Substitutions are assigned to $\eta_n(0)$, as the minor allele count is zero. The probability of observing a site with k minor alleles out of n sampled alleles in the genome is

$$g^{(2,\text{MAF})}_n(k) = \frac{\eta_n(k)}{\sum_{j=0}^{\lfloor n/2 \rfloor}\eta_n(j)}.$$

Assume the equilibrium minor allele frequency at the locus undergoing long-term balancing selection is $x \in (0, 0.5]$. The probability of observing k minor alleles out of n sampled alleles is then

$$h^{(2,\text{MAF})}_{n,x}(k) = \frac{\text{Bin}(k; n, x) + \text{Bin}(n-k; n, x)\mathbf{1}_{\{k\neq n/2\}}}{\sum_{j=1}^{n}\text{Bin}(j; n, x)}.$$

Hence, for an informative site d recombination units away from the presumed site under selection, the probability of observing k minor alleles out of n sampled alleles is

$$f^{(2,\text{MAF})}_{n,x,A}(k,d) = \alpha_A(d)h^{(2,\text{MAF})}_{n,x}(k) + [1 - \alpha_A(d)]\; g^{(2,\text{MAF})}_n(k).$$

Similarly, when substitutions are not considered or are missing in the data (*i.e.*, only observed minor alleles counts $k = 1, 2, \ldots, \lfloor n/2 \rfloor$), the two mixing components can be normalized as

$$g^{(0,\text{MAF})}_n(k) = \frac{\eta_n(k)}{\sum_{j=1}^{\lfloor n/2 \rfloor}\eta_n(j)}$$

and

$$h^{(0,\text{MAF})}_{n,x}(k) = \frac{\text{Bin}(k; n, x) + \text{Bin}(n-k; n, x)\mathbf{1}_{\{k\neq n/2\}}}{\sum_{j=1}^{n-1}\text{Bin}(j; n, x)}.$$

The probability of observing a polymorphic site with k minor alleles out of n sampled alleles is then

$$f^{(0,\text{MAF})}_{n,x,A}(k,d) = \alpha_A(d)h^{(0,\text{MAF})}_{n,x}(k) + [1 - \alpha_A(d)]\; g^{(0,\text{MAF})}_n(k).$$

Composite likelihood ratio tests based on the mixture models

In the preceding subsection, we have provided the marginal probability distributions for the number of observed copies of either a derived or a minor allele at an informative site that is a certain distance from a locus undergoing bi-allelic balancing selection. Because we cannot obtain the full likelihood that accounts for the joint distribution of allele frequencies across all informative sites that are in high linkage disequilibrium, we instead make the simplifying assumption that neighboring informative sites are independent. This assumption, albeit invalid, allows us to gain insight from the composite likelihood, which is computed by multiplying the marginal probability distributions for all informative sites. By maximizing the resulting composite likelihood from the full model across our parameter space, we can also obtain estimates of the optimal parameter values (*i.e.*, $\widehat{x}$ and $\widehat{A}$), which confer information about the features of the footprints consistent with balancing selection.

Based on the probability distributions described for the five models, for each model $X \in \{$"2", "2,MAF", "1", "0", "0,MAF"$\}$, the composite likelihood of a genomic region with L informative sites under the null hypothesis of neutrality is

$$\mathcal{L}_0^{(X)}(\mathbf{n},\mathbf{k}) = \prod_{i=1}^{L} g_{n_i}^{(X)}(k_i),$$

where $\mathbf{n} = [n_1, n_2, \ldots, n_L]$ and $\mathbf{k} = [k_1, k_2, \ldots, k_L]$ are the vectors of sample sizes and derived or minor allele counts, respectively, at the L informative sites in the genomic region. Recall that the probabilities of sampling a certain number of derived or minor alleles under our model depend on the sample sizes at informative sites, and because sample sizes often vary across the genome due to missing data in empirical studies, we make explicit the sample sizes across all informative sites in the vector $\mathbf{n}$. Similarly, the composite likelihood under the alternative hypothesis of model X would be

$$\mathcal{L}_a^{(X)}(x, A\,;\,\mathbf{n},\mathbf{k},\mathbf{d}) = \prod_{i=1}^{L} f_{n_i,x,A}^{(X)}(k_i, d_i),$$

where $\mathbf{d} = [d_1, d_2, \ldots, d_L]$ is the vector of recombination distances between the test site and each of the L informative sites. This likelihood is maximized at

$$(\widehat{x}, \widehat{A}) = \underset{(x,A)}{\arg\max}\, \mathcal{L}_a^{(X)}(x, A\,;\,\mathbf{n},\mathbf{k},\mathbf{d}).$$

Hence, under model $X \in \{$"2", "2,MAF", "1", "0", "0,MAF"$\}$, the log composite likelihood ratio test statistic for the test site is

$$B_X = 2\Big[\ln \mathcal{L}_a^{(X)}(\widehat{x}, \widehat{A}\,;\,\mathbf{n},\mathbf{k},\mathbf{d}) - \ln \mathcal{L}_0^{(X)}(\mathbf{n},\mathbf{k})\Big].$$

Note that although log-likelihood ratio test statistics can be considered as following χ^2 distributions (of which the degree of freedom is the number of free parameters, *e.g.*, two in the full models described above), B statistics are a set of composite log-likelihood ratio (CLR) statistics, which do not follow regular χ^2 distributions (Varin et al., 2011; Pace et al., 2011). In order for a CLR statistic to approximately follow an asymptotic χ^2 distribution, it needs to undergo adjustment (Pace et al., 2011) that also yields the effective degree of freedom of the asymptotic distribution the adjusted CLR statistic conforms to. This adjustment process is based on the set of observations used to compute the CLR, which is different for every test site. Because for B statistics, the size of the genomic region considered by each test varies across the genome and because the informative sites included in the region are highly correlated, the effective degree of freedom also varies across test sites. Therefore, we cannot infer significance from the values of B statistic alone by referencing the χ^2 distribution.

Moreover, and probably even more important, is that because the model under the null hypothesis only accounts for mean demographic effects based on the genome-wide SFS and not its higher moments (*e.g.*,

variance), the resulting p-value obtained from a χ^2 distribution after the statistical adjustment would still deviate from what is commonly expected when the test rejects neutrality (*i.e.*, neutral evolution under an explicit demographic model). We therefore would recommend mass simulation under an appropriate demographic model to generate the "null" distribution of B statistics in order to accurately infer the significance of each test, with the caveat that such an endeavour would require extensive computational resources due to the millions of simulations needed, the lengths of the simulated segments, and the optimization of the B statistics on each of these simulated segments. Lastly, in order to infer genome-wide significance, p-values need to be corrected for multiple testing, *e.g.*, through Bonferroni correction (Bonferroni, 1935), Simes method (Simes, 1986), or Benjamini-Hochberg procedures (Benjamini and Hochberg, 1995).

Interpretation of estimated A and x parameters

The likelihood for the alternative model is maximized over the parameters A and x, where, in our formulation for bi-allelic balancing selection in the previous subsections, x represents the presumed equilibrium minor allele frequency, and A decides the rate of exponential decay for the probability of two sites being linked, which essentially describes the influence of balancing selection on neutral sites of varying distance away from the test site. After optimizing over this parameter space, the parameter values under the optimal likelihood, $\widehat{A}$ and $\widehat{x}$, provide information on the nature of detected genomic footprints. The value of $\widehat{x}$ should reflect the enriched minor allele frequency across the region. Note that not all mechanisms for balancing selection will maintain the balanced alleles at fixed frequencies (Asmussen and Basnayake, 1990; Bergland et al., 2014), and so $\widehat{x}$ rather represents the value around which our model presumes the allele frequencies across the region are enriched. Therefore, we advise that caution be used when interpreting $\widehat{x}$ as the equilibrium frequencies without further information about the potential mechanisms that may have acted to maintain the polymorphisms.

Meanwhile, $\widehat{A}$ describes the rate of the exponential decay of the probability $\alpha_A(d) = \exp(-Ad)$ of the two loci being linked, and should intuitively be informative of the impact of balancing selection on nearby neutral sites. The smaller the $\widehat{A}$, the wider the footprint would be, and likely the younger the balanced polymorphism. However, multi-locus balancing selection can also give rise to wide footprints (Barton and Navarro, 2002; Navarro and Barton, 2002; Tennessen, 2018), which could induce small $\widehat{A}$ values. Furthermore, a large A reduces the number of informative sites that yield meaningful likelihood ratios, and can thus also occur when data in the examined area fit the alternative model poorly. Therefore, we advise only comparing the $\widehat{A}$ values among regions with reasonably high composite likelihood ratios, and that caution be used when making inferences from these values as they do not map to an explicit evolutionary model.

Results

Performances on simulated data

We simulated 50 kilobase (kb) long sequences using SLiM3.2 (Haller and Messer, 2019), under the three-species demographic model (Figure S1) inspired by the demographic history of great apes (see *Methods*), and extensively evaluated the performances of all five B statistic variants. We also compared the B statistics to the summary statistics β, β^*, HKA, NCD2, and $\beta^{(2)}$, which are respectively analogues to B_0, $B_{0,\mathrm{MAF}}$, B_1, $B_{2,\mathrm{MAF}}$, and B_2, and to the likelihood statistics T_1 and T_2, which are respectively analogues to B_1 and B_2.

Robust high power under varying window sizes

We first examined the robustness of the B statistics to overly large window sizes, under a scenario of strong heterozygote advantage (selective coefficient $s = 0.01$ with dominance coefficient $h = 20$) acting on a mutation that arose 7.5×10^4 generations prior to sampling, with all sites flanking the selected locus

evolving neutrally. Because `BetaScan` (Siewert and Voight, 2017, 2020) (which implements the standardized and nonstandardized β, β^*, and $\beta^{(2)}$ statistics, among which we only consider the standardized) operates on windows of fixed physical length, we adopted window sizes of 1, 1.5, 2.5, 3, 5, 10, 15, 20, and 25 kb for all summary statistics and B statistics. The T statistics were applied on windows with matching expected numbers of informative sites. *Supplementary Note* 2 details the calculation for matching the number of informative sites to physical length of a genomic region.

To reduce potential stochastic fluctuations in the number of true positives when the false positive rate is controlled at a low level, we examined the area under a partial curve with no greater than a 5% false positive rate (hereafter referred to as "partial AUC"). As shown in Figure 2A (see split views for separate groups of statistics in Figure S2), under optimal window sizes for most other statistics, all variants of B statistics display substantial partial AUCs comparable to that of the respective T statistic variant, which has outperformed other equivalent summary statistics in most previous simulation studies (DeGiorgio et al., 2014; Siewert and Voight, 2017, 2020; Bitarello et al., 2018; Cheng and DeGiorgio, 2019). Most remarkably, as the window size increases, while all other statistics exhibit drastic decays in power, the powers of all variants of the B statistic only show minor decreases. In fact, when comparing the powers under 25 kb windows against those under optimal window sizes for each statistic, the powers of all statistics drop more than twice as much as B_1 and B_2 (Figure 2B). In comparison with each method's optimal performance, most statistics (except all B statistics and T_2, the model-based analog of B_2) lose more than 80% of their optimal power under the largest window size examined (Figure 2C). Although T_2 still retains considerably higher partial AUC compared to all other extant methods, it still decreases to a value substantially lower than that of B_2. Such robustness of B statistics to large windows is reasonable and expected, because the probability distribution of allele frequencies at sites far enough from the test site will match the genome-wide SFS, thereby contributing little to the overall likelihood ratio.

Among all statistics evaluated, we found that those considering polymorphism data only (*i.e.*, B_0 variants and β variants) demonstrated relatively poor robustness to increases in window size. This result indicates that the detectable footprint of balancing selection in polymorphism data by itself may decay faster than other types of information, and that incorporating substitution data may help improve robustness to large windows.

Considering that the powers of all B statistics stabilize at a fixed level as the window size increases (Figure 2), we permit the B statistics to employ all informative sites on a chromosome. However, to reduce computational load, we only consider sites with mixing proportion $\alpha_A(d) \geq 10^{-8}$ for each value of A considered during optimization, which does not create discernible differences in performance from when all data are considered (Figure S3). However, to ensure that other methods still display considerable power for their comparisons, we applied the summary statistics with their optimal window sizes of one kb, and T statistics with numbers of informative sites expected in a one kb window (see *Methods*), unless otherwise stated.

High power for detecting balancing selection of varying age and selective strength

Next, we explored the powers of B statistics when the selective strength s, equilibrium frequency (controlled by the dominance parameter h), and the age of balancing selection vary. Specifically, we examined scenarios where the selective coefficients were moderate ($s = 0.01$, Figures 3A, C, D, and E) or weak ($s = 10^{-3}$, Figure 3B), and when the equilibrium frequency of the minor allele is approximately 0.5 ($h = 20$, Figures 3A and B), 0.4 ($h = 3$, Figure 3C), 0.3 ($h = 1.75$, Figure 3D), or 0.2 ($h = 1.33$, Figure 3E). Across all scenarios considered, T_2 and β^* show the highest power for old balancing selection. The best-performing B variants, B_2 and $B_{2,\mathrm{MAF}}$, display high power as well, and are often comparable to that of the $\beta^{(2)}$ statistic. The power of B_1 is also similar to HKA, which is its summary statistic analogue. Furthermore, we noticed that B statistics exhibit superior power for younger balanced alleles, particularly when balancing selection is more recent than 2×10^5 generations, and when the equilibrium frequency does not equal to 0.5 (Figure S4). For older selected polymorphisms, although several statistics outperform B statistics, it is important to point out that all previous methods were provided optimal window sizes, whereas B statistics

were set to use all sites with considerable $\alpha_A(d)$, under which they show lower power than when window sizes are optimized (Figures 2A and S2C). This difference in performance between previous methods applied with their optimal window sizes and B statistics can also explain the seemingly inferior performance of the two B_0 variants when compared with the analogous β statistics, as the B_0 variants lose more power than other B variants when computed on extended windows. When applied with the same window size, however, B_0 outperforms β by a large margin (Figures 2A and S2C). Nevertheless, these results give us confidence that B statistics have generally high power to detect young and old balancing selection, even when adopting large windows.

Robustness to recombination rate variation and elevated mutation rates

Despite their flexibility in window size and high power for detecting balancing selection, model-based methods, such as the T and B statistics, incorporate recombination distances in their inference framework, and can therefore be especially susceptible to potential inaccuracies in input recombination maps. Additionally, because many approaches for detecting balancing selection aim to identify genomic regions with increased genetic diversity, the elevation of mutation rates is also a common and potent confounding factor for detecting balancing selection (Charlesworth, 2006; Siewert and Voight, 2020; Cheng and DeGiorgio, 2019).

To test their robustness to inaccurate recombination rates, we applied B and T statistics on simulated sequences with uneven recombination maps (10^2-fold fluctuations in recombination rates; see *Methods*). When the sequences evolve neutrally, neither approach is misled (Figures S5 and S6). When the fluctuation in recombination rate is even more drastic (*e.g.*, 10^4-fold instead of 10^2), all methods tend to report fewer false signals than they would under a uniform map (Figures S7 and S8). This result suggests that the misleading effects of inaccurate recombination maps are limited.

To examine their robustness against unexpected mutation rate variation, we next simulated a 10 kb mutational hotspot at the center of the 50 kb sequence with a mutation rate five times higher than original and surrounding rate μ, and applied each statistic with parameters derived from the original neutral replicates with constant mutation rate μ across the entire sequence. All methods exhibit considerable robustness against this regional increase of mutation rate (Figure S9 and S10).

We further considered an elevated mutation rate of 5μ across the entire 50 kb sequence, and re-examined the robustness of each method. As expected, most statistics display substantially inflated proportions of false signals (*i.e.*, reported signals of balancing selection from sequences neutrally evolving with 5μ mutation rate; Figures S11A and D and S12). Among them, the B_2 statistic reports the least proportion of false signals, followed by the B_1 statistic. Meanwhile, at low false positive rates, B_2 and $B_{2,\mathrm{MAF}}$ statistics report higher proportions of false signals than T_2, their coalescence model-based analogue, whereas B_1 outperformed T_1. Additionally, all statistics that consider only polymorphism data, namely the B_0, $B_{0,\mathrm{MAF}}$, β, and β^* statistics, are substantially misled. The $\beta^{(2)}$ statistic, albeit taking substitutions into account, also displays surprisingly high proportions of false signals.

We next explored how the regional mutation rate elevation in the genome could affect the detection of balancing selection. To this end, we mixed neutral sequences evolving with 5μ mutation rates with those with the original μ mutation rate at varying proportions (5, 10, 25, or 50%), and used these mixed pools of neutral sequences as the "whole-genome" to compute their SFSs, inter-species coalescent times, and polymorphism-substitution ratios to inform T, B, β, and HKA statistics of the neutral variation levels. We then scanned these sequences with summary statistics adopting one-kb windows, T statistics adopting 12-site windows, and B statistics using the whole sequence. We found that as the proportion of fast-mutating neutral sequences increases, most methods show substantially compromised powers (Figure S13). Among them, however, T_2 and NCD consistently exhibit considerable power throughout all scenarios examined, followed by T_1, $B_{2,\mathrm{MAF}}$, B_1, and B_2, which still retain some power despite substantial drops. Meanwhile, the methods that do not effectively utilize substitutions, *i.e.*, B_0, $B_{0,\mathrm{MAF}}$ and β statistics, almost lose all the power. This is consistent with previous results, suggesting that the absence of substitution renders methods for detecting balancing selection susceptible to the confounding effects of unexpected mutation

rates.

With knowledge of their robustness against unexpected mutation rate elevation, we further examined the powers of each method to detect balancing selection within sequences evolving with high mutation rates when they are correctly informed. That is, T and β statistics are provided the correct population-scaled mutation rate and inter-species coalescent time, and all except for B statistics adopt their optimal window sizes of one kb (60 informative sites for T statistics). We simulated sequences undergoing balancing selection that initiated 250,000 generations ago with a neutral mutation rate of 5μ across the simulated segment, and applied summary and T statistics on the sequences mutating at a rate of 5μ, with their optimal window sizes under the correct mutation rate. Figure S14 demonstrates that the powers of all methods are substantially higher than for the identical scenario with sequences evolving under the original neutral mutation rate μ (compare to Figures 3C and S4C). This improved detection ability likely results from the roughly five-fold increase in the number of informative sites included within each window. The T statistics display lower areas under their receiver operating characteristic curves than their equivalent B statistics (Figure S14A), and the $B_{0,\text{MAF}}$ and $B_{2,\text{MAF}}$ statistics perform substantially worse than their respective derived allele frequency counterparts B_0 and B_2. Moreover, as with other simulated scenarios, we find that the power of $B_{0,\text{MAF}}$ is lower than others (Figure S14B). However, when the window size for all summary statistics is expanded from the optimal one kb to a sub-optimal five kb, their powers substantially decrease to levels similar to $B_{0,\text{MAF}}$.

Robust power under realistic demographic models

The influence of demographic history was the major motivation for T statistics to adopt the genome-wide SFS instead of the coalescence-based constant-size neutral model as the null hypothesis, despite that the latter being nested under the alternative model for balancing selection used by the T statistics. This trade-off has endowed T statistics with considerable robustness to population size changes (DeGiorgio et al., 2014; Cheng and DeGiorgio, 2019), but has also potentially compromised their robustness to large windows, as shown in *Robust high power under varying window sizes* subsection of the *Results*. For B statistics, however, because their null models both reflect the genome-wide SFS and are nested under the alternative models, they should exhibit considerable robustness to both oversized windows and demographic changes.

To evaluate their performances under recent population expansions and bottlenecks, we considered the demographic histories of contemporary European humans (Terhorst et al., 2017, CEU; Figure S15A) and bonobos (Prado-Martinez et al., 2013, Figure S16A; see details in *Methods*), respectively. The former have been extensively characterized (*e.g.,* Lohmueller et al., 2009; Gravel et al., 2011; Terhorst et al., 2017), and therefore can reliably reflect the performance of each method under realistic scenarios. On the other hand, because we intend to apply the B statistics on bonobo genomic data, we are also interested in evaluating their performance under an inferred bonobo demographic model.

As previously described, we applied the B statistics with unlimited window sizes, whereas the other statistics were provided with smaller window sizes matching the theoretical size for a footprint of long-term balancing selection (see *Supplementary Note* 2). Despite being provided disadvantageous window sizes, B statistics still demonstrate comparable to, and often higher power than, current summary statistic approaches, both under the human (Figure S15) and the bonobo (Figure S16) demographic models. Although T_2 has higher power than the B statistics, we note that the T statistics were operating with optimal window sizes, whereas the window sizes for B statistics are identified across a parameter range. When B_1 and B_2 are applied with identical window sizes as T_1 and T_2 (Figures S17 and S18), the margins between their performances are no longer substantial. Additionally, we noticed that most statistics tend to have higher power for sequences evolving under the bonobo demographic history than under that of the Europeans (notice that the y-axes in Figures S15 and S16 have different scales).

Robust power under varying mutation rates across target and outgroup species

In addition to temporally-varying population sizes, differing mutation rates between closely-related species may also affect the performance of the coalescence-based T statistics, as they assume a uniform neutral mutation rate along the genealogy relating the lineages from the ingroup and outgroup species. Among great apes, for example, accumulating evidence suggests that humans have substantially lower mutation rates than other great apes (as reviewed by Scally and Durbin, 2012).

To examine the behavior of each method when mutation rates of the target and outgroup species differ, we simulated a two-species demographic history, with the target and outgroup species respectively evolving at neutral rates $\mu = 1.2 \times 10^{-8}$ and $\mu = 2.5 \times 10^{-8}$ mutations per site per generation (see *Methods* for details). We introduced an adaptive mutation evolving under balancing selection at varying time points prior to sampling along this demographic history, and examined the power of each statistic to detect balancing selection across a diverse array of selection parameters (Figure S19).

Across all six combinations of selection parameters considered, we observe similar trends for each statistic when compared with simulations under the constant population size (Figure 3) and CEU (Figure S15) demographic histories evolving with a constant neutral mutation rate. The T_2 statistic performs the best when $s = 0.01$ with $h = 20$ (Figure S19A), under which the equilibrium frequency is closest to 0.5 and when heterozygotes are most advantageous. As the selective advantage hs and equilibrium frequency decrease, the margin between the powers of T_2 and B_2 shrinks, and even reverses for all scenarios with small dominance h (Figures S19C-F). Furthermore, methods based solely on polymorphism and substitution calls (*i.e.*, T_1, B_1, and HKA) show improvements in power as the equilibrium frequency decreases, and some even outperform most of the other statistics (Figures S19D and E). Statistics that ignore substitutions (*i.e.*, B_0, $B_{0,\mathrm{MAF}}$, β, and β^*), on the other hand, perform especially well for recent balancing selection with high heterozygote advantage (large hs; Figures S19A and B). As the balanced alleles reach their equilibrium frequencies sooner when the selective advantage of heterozygotes (*i.e.*, hs) is high, sequences with mutations of higher hs would have older footprints than those with mutations introduced at the same time but with lower hs. In this respect, it is understandable that B_0 and β variants outperform others only for selection with large hs that are introduced within 150,000 generations prior to sampling.

Based on this two-species model with diverging mutation rates, we further integrated changes in population size of the target species in accordance with the demographic history of the CEU (Terhorst et al., 2017, Figure S20). From the four sets of selection parameters tested, we found that most methods exhibit lower power compared with those under constant population sizes (Figure S19). This result is consistent with the lower powers under simulations with a constant mutation rate when the target population size evolves under the CEU demographic history (Figure S15) compared with the setting in which the target evolves with constant size (Figure 3). Despite their lower powers in general, we still observe similar relative performances across statistics, with T_1 and B_1 exhibiting higher powers when the heterozygote advantage hs is small. Moreover, we found that $B_{2,\mathrm{MAF}}$ shows superior power to B_2.

Re-examining long-term balancing selection in human populations

We applied B_2 on contemporary European (Europeans in Utah; CEU, Figure S22) and west African (Yoruban; YRI, Figure S21) human populations from the 1000 Genomes Project dataset (The 1000 Genomes Project Consortium, 2015) (see *Methods*) to re-examine the footprints of long-term balancing selection, which previous studies (DeGiorgio et al., 2014; Siewert and Voight, 2017) have provided cases for reference. The most outstanding candidates in both scans localize in the HLA-D region (human leukocyte antigen, also known as major histo-compatibility [MHC] Class II region) (Figures S23 and S24), agreeing with previous findings (Sanchez-Mazas, 2007; Leffler et al., 2013; DeGiorgio et al., 2014; Teixeira et al., 2015; Siewert and Voight, 2017; Meyer et al., 2017; Bitarello et al., 2018). Within the HLA-D region, the B_2 scores computed for both populations show extraordinary peaks around *HLA-DQ* and *HLA-DP* gene clusters, although CEU (Figure S23) scores remarkably higher on *HLA-DP* genes than YRI (Figure S24). Echoing the critical roles of HLA-D genes in adaptive immunity, the gene *ERAP2* exhibits extraordinary

scores in both populations (Figures S25 and S26). This gene has been reported in past studies of balancing selection in humans (Andrés et al., 2009, 2010; Bitarello et al., 2018), and Andrés et al. (2010) demonstrated that its splicing variants can alter the level of MHC-I presentation on B cells. Additionally, we also observed high B_2 scores on *CADM2* (Figures S27 and S28) and *WFS1* (Figures S29 and S30), on which Siewert and Voight (2017) characterized potential non-synonymous mutations segregating in both populations.

In addition to these previously characterized candidates, both scans display extreme B_2 scores on another two top-ranking regions in the T_2 scans by DeGiorgio et al. (2014): the *STPG2* gene (formerly named *C4orf37*; Figures S31 and S32) and the *CCDC169-SOHLH2* (formerly named *C13orf38-SOHLH2*; Figures S33 and S34) region, with *STPG2* particularly more outstanding in the scan of YRI than in CEU. Intriguingly, both these genes are associated with gametes. The *STPG2* gene encodes sperm-tail PG-rich repeat-containing protein 2, which, despite the paucity of literature that describes its function, is found in sperm (Uhlén et al., 2015). The high-scoring region on this gene harbors a number of tissue-specific eQTLs for its expression, especially in brain and reproductive tissues (GTEx Consortium, 2017). The *SOHLH2* gene, on the other hand, encodes the transcription factor Spermatogenesis and Oogenesis Specific Basic Helix-Loop-Helix-containing protein 2, which plays important roles in both spermatogenesis and oogenesis (Toyoda et al., 2009; Suzuki et al., 2012). We observed drastically elevated B_2 scores (Figure S33) across an extended region upstream of *SOHLH2* that covers the naturally occurring *CCDC169-SOHLH2* readthrough transcript (as introduced in RefSeq database; O'Leary et al., 2015). Similar to *STPG2*, this region also features numerous eQTLs for the expression of *SOHLH2*, especially in endocrine glands, brain, and reproductive tissues (GTEx Consortium, 2017).

Other regions with outstanding peaks shared by both scans include the genes *CPE* (Figures S35 and S36) and *MYOM2* (Figures S37 and S38). *CPE* encodes carboxypeptidase E, a key enzyme for synthesizing peptide hormones such as insulin and oxytocin, and its mutant mice strain (Cpe^{fat}) exhibits endocrinic disorders such as obesity and infertility (Naggert et al., 1995). *MYOM2* encodes endosacromeric cytoskeleton M-protein 2, which serves as a structural component of muscle tissues (van der Ven et al., 1999). Both genes harbor eQTLs reported by GTEx Consortium (2017) around the high-scoring regions.

Probing for footprints of balancing selection in bonobo genomes

We further applied the B_2 statistic on the variant calls of 13 bonobos (Prado-Martinez et al., 2013) lifted-over to human genome assembly GRCh38/hg38. Only bi-allelic single nucleotide polymorphisms (SNPs) were considered, and substitutions were called using bonobo panPan2 reference sequence (Prüfer et al., 2012), with the human sequence as the ancestral state. Stringent filters were applied to remove repetitive regions and regions with poor mappability (see *Methods*). We observed many genomic regions with outstanding B_2 scores (Figure 4), which include both the *MHC-DQ* and *MHC-DP* genes and a few novel candidates.

Among the outstanding peaks, the top two cover the *MHC-DQA1*, *MHC-DQB1*, *MHC-DPA1*, *MHC-DPB1*, and *MHC-DPB2* genes, which harbor all the top 0.01 percent B_2 scores. (Figure 5A). Such high scores can be explained both by the elevated proportion of polymorphic sites, 0.299 as compared with the genome-wide proportion of 0.237 (Figure 5B; note that genes are labeled based on human hg38 genome annotations), as well as the enrichment of polymorphic sites with moderate minor allele frequencies (Figure 5C). Furthermore, the region exhibits a multimodal SFS, which may correlate to the multiple B_2 peaks observed in the region.

In addition to the *MHC-DQ* and *MHC-DP* genes, *KLRD1* also presents prominent B_2 scores (Figure S39) on its first intron. This gene expresses a natural killer (NK) cell surface antigen, also known as CD94, and plays a pivotal role in viral defense. Unlike the region covering *MHC-DQ* genes, the minor allele frequencies at polymorphic sites around the *KLRD1* region are clearly enriched near a frequency of 0.45, instead of the multimodal distribution observed around the *MHC-DQ* genes. We also found other high-scoring regions associated with innate immunity, such as the gene *GPNMB* (Figure S40), gene *LY86* (Figure S41), and the intergenic region between *BPIFB4* and *BPIFA2* (Figure S42).

Another interesting candidate is the pain perception gene *SCN9A* (Figure S43), on which the highest scores overlap with the transcript of its anti-sense RNA gene that regulates its expression. Instead of enriching toward a single value, the minor allele frequencies at the polymorphic sites across the region are dispersed, with at least two modes (approximate minor allele frequencies of 0.25 and 0.40). This finding may correlate with the multiple peaks identified around this region, which may be sensible given the large number of exons covered. Similarly, the anti-sense RNA gene *ARHGEF26-AS1* (Figure S44) harbors high B_2 scores, with allele frequencies enriched around 0.15 and 0.45. Other notable candidates include *PDE1A* (Figure S45), which encodes a pivotal enzyme in cellular Ca^{2+}- and cyclic nucleotide signaling (Michibata et al., 2001) with multiple splicing variants and plays roles in both neurodevelopment (Pekcec et al., 2018) and sperm functionality (Lefièvre et al., 2002). A few other genes scoring in the top 0.05% are also involved in spermatogenesis or gamete functionality while serving other important functions, such as a Ca^{2+}/calmodulin-dependent protein kinase gene *CAMK4* (Figure S46; Sikela et al., 1990) and a cancer-related gene *SUSD2* (Figure S47; Watson et al., 2013; Harichandan et al., 2013).

Discussion

In this study, we introduced a novel set of composite likelihood ratio statistics—B_2, $B_{2,\mathrm{MAF}}$, B_1, B_0, and $B_{0,\mathrm{MAF}}$—to robustly detect footprints of balancing selection with high power and flexibility. The B statistics are based on a mixture model creating a proper nested likelihood ratio test, which helps them overcome the common susceptibility to oversized windows held by current methods. We have extensively evaluated their performances on simulated data compared with current state-of-the-art methods, and have demonstrated the superior properties of the B statistics under various scenarios. We re-examined balancing selection in human populations (The 1000 Genomes Project Consortium, 2015), and recovered well-established candidates including the HLA-D genes and *ERAP2*. We further applied B_2 onto the genomic data of bonobos (Prado-Martinez et al., 2013), and uncovered not only the *MHC-DQ* and *MHC-DP* gene cluster, but also intriguing candidates that are involved in innate immunity, neuro-sensory development, and gamete functionality.

Evaluating the performance of B statistics through simulations

In our simulation study, the B statistics showed remarkable robustness to large window sizes, with only minor decays in power under oversized windows, whereas other methods exhibited large declines in power. Moreover, even when considering all data available as input (*i.e.*, the most disadvantageous window size) all variants of B statistics still exhibit comparable power to extant methods and displayed satisfactory performance across varying types and strengths of balancing selection. Under scenarios with confounding factors, such as high mutation rate and non-equilibrium demographic history, the B statistics demonstrated satisfactory robustness as well.

The robustness against varying window sizes is of particular interest in this study, not only because it ensures high power under large windows, but it also allows the statistics to augment the size of genomic regions from which they make meaningful inferences. This flexibility grants a key advantage over previous methods that require the window size to be fixed throughout the scan in order to yield comparable results across the genome. In particular, because many factors (such as recombination rates) can influence the footprint size of balancing selection, it is not ideal to adopt a fixed window size for a whole-genome scan based on a uniform population-scaled recombination rate, and B statistics naturally accommodate such variability across the genome.

Admittedly, in practice, as the genomic region considered in the tests expands, non-neutral sites will inevitably be included. This indeed violates our assumption that the test locus is surrounded by neutral sites only. Nonetheless, because both positive and purifying selection reduce the presentation of sites with intermediate frequencies (Tajima, 1989; Braverman et al., 1995; Fay and Wu, 2000; Bamshad and Wooding, 2003), their effect on the SFS is in general opposite to the features expected from balancing

selection. This suggests that including such sites in the window is unlikely to hamper the power to detect balancing selection. Meanwhile, when multiple sites in the considered region undergo balancing selection, the pattern of polymorphisms across the region will indeed differ from that in regions with a single selected locus. We will discuss the effects of such multi-locus balancing selection in the subsequent subsection *Performance of single-locus methods on multi-locus balancing selection.*

One important consideration is that, so far our simulation study (as well as previous ones by DeGiorgio et al., 2014; Bitarello et al., 2018; Siewert and Voight, 2020) only evaluates the method performance in the context of single-locus heterozygote advantage. For many other balancing selection mechanisms, such as negative frequency-dependent selection (Asmussen and Basnayake, 1990) and periodic environmental fluctuations (Bergland et al., 2014), a stable equilibrium cannot be guaranteed (Cockerham et al., 1972; Asmussen and Feldman, 1977; Ginzburg, 1977). In non-overdominance settings for which particular equilibrium frequencies indeed exist, the balanced alleles are still maintained near these fixed frequencies, thereby satisfying the general assumptions of the statistical models underlying our B statistics. Moreover, when such intrinsic equilibrium frequencies do not exist, allele frequencies may still fluctuate around some mean values. Even if such mean values are unattainable, there will still persist an enrichment of sites with intermediate frequencies, thereby presenting characteristic footprints of balancing selection. We therefore believe that our mixture model framework should still have high power to detect footprints of non-overdominance balancing selection, and that overall, our results have comprehensively characterized the promising performance of the B statistics.

Confounding effects of mutation rate or recombination rate variation

In our simulation study, sequences with a central 10 kb mutational hotspot did not mislead methods as much as those with the mutation rate elevated across the entire sequence (Figure S9). This result may seem counter-intuitive at first, as a smaller region of increased mutation rate may better resemble the footprints of long-term balancing selection. However, upon a closer examination of the site frequency spectra and proportions of polymorphic sites (Figure S48), sequences with an extended region of high mutation rate exhibit a greater departure in these features under scenarios with no elevated mutation rate than for scenarios with a central mutational hotspot. Specifically, these sequences have more sites with high derived allele frequencies and a higher proportion of polymorphic sites overall (Figure S48B), likely resulting from the recurrent mutation on sites that were originally substitutions. The increase is also more profound on sites with high derived allele frequency. For example, the proportions of sites with derived allele frequency of 0.96 increased by almost two-fold from approximately 0.00104 to 0.00190, and the proportions of sites with derived allele frequency of 0.98 increased by almost three-fold from 0.00105 to 0.00273. By contrast, the difference in scale between the proportions of polymorphisms (0.182 versus 0.189) is minor. The larger fold-change in the proportions of high-frequency polymorphisms (*i.e.*, sites with $k = n - 1$, $n - 2$, and $n - 3$ derived alleles) relative to that of substitutions ($k = n$ derived alleles) could explain the more profound inflation in power for the statistics relying only on information at polymorphic sites. Similarly, after folding the SFS, the large changes in the proportions of low-frequency alleles were substantially mitigated, echoing the superior performance of $B_{2,\mathrm{MAF}}$ and β relative to their unfolded counterparts.

Another unexpected result from the simulations of elevated mutation rate is the drastic inflation of false signals reported by β statistics (Figure S11), which can also be observed in the non-standardized β statistics (Figure S49). Although Siewert and Voight (2020) tested their power to detect balancing selection under high mutation rate, it was unexplored whether their β statistics would mis-classify highly mutable neutral sequences as those undergoing balancing selection, and our results show that they could be easily misled. However, we further found that the performances of the standardized β statistics largely improve when provided with the correct mutation rate and divergence time (Figure S49B). This result partly confirms the superiority of standardized β statistics over the unstandardized ones. It also suggests that β statistics are considerably susceptible to the confounding effect of mutation rate elevation, and that their performance relies highly on the accuracy of the provided mutation rate. Instead of using a

constant mutation rate for the entire scan, we propose that providing locally-inferred population-scaled mutation rates θ may help improve the robustness of β statistics. Indeed, when we instead estimate θ using the mean pairwise sequence difference $\widehat{\theta}_\pi$ (Tajima, 1983) for each replicate and provided `BetaScan` the respective inferred value as the θ parameter, the standardized statistics no longer report as many false signals (Figure S49C). However, we also observed that providing a locally-inferred θ estimate compromises the power of standardized β statistics to detect balancing selection, both under normal (*i.e.*, μ) and elevated (*i.e.*, 5μ) mutation rates (Figures S50 and S51, respectively), especially for the unfolded β^* and $\beta^{(2)}$ statistics. This result is probably because, in addition to an elevation in mutation rate, the locally-inferred θ can also be inflated by footprints of balancing selection, thereby decreasing the β statistic's sensitivity.

In contrast to mutation rate variation, all statistics are robust to recombination rate variation, with B_0 and $B_{0,\mathrm{MAF}}$ reporting substantially fewer false signals than the others (Figure S5). This robustness to recombination rate variation may be explained by the high similarity in the SFS and proportion of polymorphic sites to sequences evolving under a uniform recombination rate (Figure S52).

Effect of multiple testing on sequences with high mutation rates

Because B, T, and β statistics are computed on every informative site, as suggested by Cheng and DeGiorgio (2019), multiple-testing can account for some inflation in their powers because sequences with a higher mutation rate will have a greater number of informative sites. To evaluate the effect of multiple testing for sequences with high mutation rates, we down-sampled the test sites (see *Methods*) such that the number of test scores being computed approximately matches that under the original mutation rate μ. Although all statistics show varying levels of improvements in performance (Figures S11B, C, E, and F), some still report high proportions of false signals, especially all β statistics and $B_{0,\mathrm{MAF}}$. That is, multiple-testing cannot account for all the factors that drive these statistics to mis-identify features of elevated mutation rates as footprints of balancing selection. This result corresponds to the fact that both the SFS and the density of polymorphic sites are altered under scenarios with extended regions of elevated mutation rate (Figure S48), likely due to recurrent mutation.

Furthermore, we observed that both before and after down-sampling, the T statistics report fewer false signals than their respective B statistic analogues. One potential factor behind their marginally superior performance may be that T statistics perform tests on fixed numbers of informative sites, instead of genomic regions measured by physical lengths (as did B statistics and the summary statistics). For T statistics, the size of the genomic region covered by the same number of informative sites would be much narrower under rapidly mutating sequences than in sequences with the original mutation rate. This means that the resulting T scores in either scenario are reflective of the levels of variation for sequences with drastically different lengths. To account for this factor, we provide B_1 and B_2 with informative site-based windows identical to that of T statistics and re-examined their performances (Figures S53 and S54). After matching the windows, B_1 and B_2 variants in turn display higher robustness than T_1 and T_2 to elevated mutation rates, suggesting that B statistics are at least comparably robust to T statistics. Meanwhile, we also matched the window size for B_0 variants and β to gauge the effect of adopting large windows on the proportions of false signals from B_0 variants. When B_0 scans the sequences with one kb windows, though there is an increase in the resulting number of false signals (Figures S53A and S54C), at a 1% false positive rate the proportions of false signals for the two B_0 variants only increase by less than 0.1, and are still substantially lower than that of β and β^* (Figures S53B and S54C and D).

Comparing the B statistics with the T statistics

Because the T statistics of DeGiorgio et al. (2014) have previously been the only model-based approach for the detection of long-term balancing selection from polymorphism data in a single species, the comparisons between the model-based B and T approaches is particularly intriguing for researchers with empirical data suitable for the application of either. The T statistics are based on an explicit coalescent model (Hudson

and Kaplan, 1988; Kaplan et al., 1988), and have been shown to have superior power to a number of other methods in previous studies (DeGiorgio et al., 2014; Siewert and Voight, 2017, 2020; Bitarello et al., 2018; Cheng and DeGiorgio, 2019), consistent with our simulation results. The B statistics, on the other hand, employ a mixture model, where the component modeling balancing selection is not based on an explicit evolutionary model, but nevertheless shows impressive performance on simulated data, as the shape of the distribution of allele frequencies is similar to what might be expected under balancing selection. The often superior performances of both approaches over summary statistics is understandable, as both utilize the genomic spatial distribution of genetic diversity in their inferences.

However, within the T statistic framework, the model for the null hypothesis (neutrality) is not nested in the alternative hypothesis (balancing selection). Although the T_1 and T_2 statistics could have adopted nested models by employing the standard neutral coalescent as the model for the null hypothesis, doing so would increase susceptibility to demographic factors, which can also alter the genome-wide SFS. To better account for these factors, DeGiorgio et al. (2014) instead employed the genome-wide distribution of genetic variation to compute probabilities under the null hypothesis of neutrality. This explains the substantial decay in power for both T statistics as the window size increases (Figures 2 and S2A and B), as well as its robust performance under varying sized demographic models (DeGiorgio et al., 2014; Cheng and DeGiorgio, 2019, Figures S15 and S16). In contrast to the T statistics, the null model for B statistics (which also employs the genome-wide SFS) is nested within the alternative, due to their mixture model framework. This feature mitigates the biases introduced by sites far from the test site, while simulataneously accounting for demographic factors. Consequently, the B statistics display robust performance under oversized windows and realistic demographic models in our simulations (Figures 2, S2, S15, and S16).

Another advantage of the B statistics over the T statistic approach, especially for B_2 compared with T_2, is the computational load. Because the probability distribution of allele frequencies under the Kaplan-Darden-Hudson (Kaplan et al., 1988) model is difficult to compute, the T_2 statistic relies on previously-generated sets of simulated site frequency spectra over a grid of equilibrium frequencies $x \in \{0.05, 0.10, \ldots, 0.95\}$ for each distinct sample size n and recombination distance d. Generation of such frequency spectra is computationally intensive, and the load increases substantially with the increase in sample size, thereby limiting the application of T_2 to datasets with larger sample sizes. However, this is not a limitation of B_2, as the SFS under balancing selection is determined simply as a mixture of the given genome-wide distribution of allele frequencies and a statistical distribution with closed-form solutions whose computational cost is minor, and only increases linearly with the sample size. Moreover, the rapid computation of this spectrum permits a finer grid of equilibrium frequencies x to be interrogated.

Considering multi-allelic or multi-locus balancing selection

Both model-based approaches employed by the T and B statistics assume that balancing selection acts on a single bi-allelic locus. Whereas this case may be the most intuitive and simplistic scenario to model and simulate, many well-established empirical examples of balancing selection—such as the MHC locus in animals (Wills, 1991; Hedrick, 2002), the ABO blood group in primates (Saitou and Yamamoto, 1997; Fumagalli et al., 2009; Ségurel et al., 2012; Leffler et al., 2013), and the plant self-incompatibility locus (Charlesworth et al., 2000)—feature multiple alleles balanced across an extended genomic region. It therefore brings into question how these methods perform on genomic regions evolving under multi-allelic or multi-locus balancing selection, and whether current frameworks can be extended to consider these more complicated cases of balancing selection.

Extending mixture models to account for multi-allelic balancing selection

There exist theoretical models of multi-allelic balancing selection based on the coalescent (Hey, 1991; Muirhead and Wakeley, 2009). However, possibly due to computational constraints, such models have not been implemented within a likelihood-ratio framework for detecting the footprints they characterize. Here, instead of following DeGiorgio et al. (2014) to compute the densities of polymorphisms and substitutions

or to approximate the SFS using simulations under an explicit coalescent model, our mixture models can be readily extended to account for multi-allelic balancing selection at a single locus without the extensive computational burden of coalescent-based approaches that integrate selection. Specifically, we consider samples with multiple balanced alleles as following multinomial distributions (see *Supplementary Note 1*), and henceforth use the mixture models to approximate the SFS at bi-allelic neutral sites that are linked to a selected locus with $m \in \{2, 3, 4, \ldots\}$ balanced allelic classes. This extension is also implemented in our `BalLeRMix` software, with the special case of $m = 2$ reducing to the model introduced in the *Model Description* section.

To simulate single-locus multi-allelic balancing selection, we employed SLiM version 3.3, which can simultaneously incorporate the four standard nucleotides of DNA, and thus allows these distinct nucleotides to coexist at the same site. We introduced two, three, or four distinct mutations with fitness parameters $s = 0.001$ and $h = 20$ in each simulated replicate 500,000 generations in the past to examine the relative performances of T, bi-allelic B, and multi-allelic B statistics. Under this fitness scheme, the equilibrium frequencies when two, three, or four alleles are balanced in the population are approximately $(1/2, 1/2)$, $(1/3, 1/3, 1/3)$, or $(1/4, 1/4, 1/4, 1/4)$, respectively (see *Methods* for details). As the number of balanced alleles assumed by B statistics (*i.e.*, parameter m) increases, the powers of B statistics barely change when two (Figures S55A-C) overdominant mutations are introduced. When more than two overdomiant alleles are balanced in the population, it is remarkable that B statistics with m set to three or four (Figures S55E and F, respectively) outperform those with $m = 2$ (Figure S55D). Furthermore, we also observe that the optimal equilibrium minor allele frequencies reported by the B statistics match well with the true equilibrium frequencies in the simulated replicates (Figure S56).

To further dissect the relative performances of B statistics (with $m = 4$), we also applied other statistics with their optimal window sizes on these simulated sequences (Figure S57). As the number of balanced alleles increases, each statistic demonstrated improvements in their power. Furthermore, the B_1, B_2 and $B_{2,\text{MAF}}$ statistics outperform their respective T- or summary-statistic analogs under all three scenarios considered.

Taken together, these results suggest that the multi-allelic B statistics can substantially improve the detection power for balancing selection with more than two balanced alleles. Moreover, B statistics with larger m parameters, the presumed number of balanced alleles, are downward compatible with population samples carrying fewer than m balanced alleles, as the presumed equilibrium minor allele frequencies of the extra allelic classes would be optimized close to zero (see Figure S56).

Performance of single-locus methods on multi-locus balancing selection

Similar to multi-allelic balancing selection, despite previous theoretical work to model or simulate multi-locus balancing selection (Navarro and Barton, 2002; Barton and Navarro, 2002; Tennessen, 2018), no detection approach has yet been developed accordingly. Meanwhile, neither model-based detection framework underlying the T statistics nor the B statistics can address these cases without jointly accounting for allelic combinations at multiple informative sites as the target of selection. Therefore, without shifting the paradigm to consider such site-to-site combinations so as to accurately locate the set of neighboring selected loci, one can still examine the performance of extant balancing selection approaches for locating genomic regions containing more than one locus under balancing selection.

To this end, we tested the simplest case with two nearby loci carrying independent overdominant alleles (see *Methods*). To ensure individuals heterozygous at both loci are as advantageous as in the single-locus balancing selection simulations with $s = 0.001$ and $h = 20$ (Figures S58A and B), we set the selective coefficients of both overdominant mutations to $s = 0.0005$. That is, a two-locus genotype that is heterozygous at each of the loci would have fitness approximately equal to $1 + 2hs = 1.2$. Despite this adjustment, we observed that all statistics show drastic improvements in their powers (Figure S58C and D), with the lowest power among them of 0.8 (Figure S58D). This result suggests that multi-locus balancing selection can potentially create more-prominent footprints compared with single-locus balancing selection. To further gauge the extent to which the additional selected locus can boost detection power, we simulated

sequences with two nearby loci each evolving under $s = 10^{-5}$ and $h = 20$, such that the selective coefficient s is two orders of magnitude smaller than that of the mutations introduced in the sequences evolving under single-locus balancing selection (Figures S58A and B). Remarkably, all methods still exhibit substantially higher powers for sequences with two nearby loci with weakly-advantageous ($s = 10^{-5}$) alleles undergoing balancing selection (Figures S58E and F).

The higher powers observed for simulated multi-locus balancing selection scenarios is understandable, as Tennessen (2018) demonstrated that two non-interacting neighboring loci tend to reinforce the maintenance of polymorphisms when both are independently subjected to balancing selection. However, multi-locus balancing selection can also be achieved by epistasis (Barton and Navarro, 2002; Navarro and Barton, 2002), whereby the fitness effect of one locus is contingent on the allelic state of another locus, and has been shown by a growing body of empirical studies to be pervasive in the genome (as reviewed by Shao et al., 2008; Lehner, 2011; Mackay, 2014). Though we did not simulate such scenarios in this study, because two interacting loci would better maintain polymorphisms at the selected loci than would two non-interacting ones (Barton and Navarro, 2002; Navarro and Barton, 2002; Tennessen, 2018), it would not be surprising that they would produce even stronger footprints than what we observe here.

Furthermore, genomic sequences with multiple nearby balanced loci will have more extended footprints of balancing selection. With the capability to optimize over window sizes, B statistics should be more sensitive to such regions than other approaches applied with small fixed windows. Indeed, B_2 substantially outperforms T_2 (applied with 12 informative sites on either side of a test site) when the two neighboring loci under selection are weakly advantageous themselves (Figures S58E and F). The margins between their powers still persist even when T statistics adopt windows with 122 informative sites on either side of the test site (Figures S59E and F), despite the marginal increases in their powers for two-locus balancing selection.

Our exploratory results not only imply that extant approaches for detecting balancing selection have high power when applied to genomic regions carrying multiple balanced loci, but that such power are also likely much higher than they would have for single-locus regions. For B statistics in particular, because they optimize over window sizes, the gap between their sensitivity for multi-locus balancing selection and that for single-locus settings may be more profound than other methods when applied with small windows. Our results also support the speculation that top candidates identified in previous scans for balancing selection may be more likely to carry more than one functional polymorphic site, as is the case for the MHC locus, considering all methods we evaluated show higher powers for multi-locus balancing selection than for the single-locus process.

Application of B_2 to empirical data

In this study, we applied the B_2 statistic on both human and bonobo genomic data, and identified sensible candidate targets in each species. We first re-examined the CEU and YRI human populations in the 1000 Genomes Project dataset (The 1000 Genomes Project Consortium, 2015) with B_2, which have been previously probed for long-term balancing selection in multiple studies (DeGiorgio et al., 2014; Siewert and Voight, 2017; Bitarello et al., 2018). We found that top candidates reported by B_2 overlap largely with previous scans, lending confidence in the power of B statistics to make replicable discoveries. Next, we performed the first model-based scan for footprints of balancing selection on bonobo polymorphism data. In addition to the genomic regions previously reported to be under ancient balancing selection in humans and chimpanzees (*e.g.*, the *MHC-DQ* genes at the MHC locus; Leffler et al., 2013; Teixeira et al., 2015; Cheng and DeGiorgio, 2019), we have also uncovered novel candidates such as *KLRD1* and *SCN9A*, which play roles in pathogen defense and pain perception, respectively. Our results may correspond to the unique features and evolutionary history of bonobos, as suggested by accumulating evidence (de Waal, 1990; Hare et al., 2012; de Groot et al., 2017; Wroblewski et al., 2017; Maibach and Vigilant, 2019) on bonobo behavior and physiology.

Potential balancing selection on gamete-associated genes in humans

In the scans of human populations, we recovered previously reported candidates *STPG2* (formerly *C4orf13*) and *CCDC169* (formerly *C13orf38*), in addition to the HLA-D locus and *ERAP2*. Neither of the two former genes was discussed in previous studies after reporting them as top candidates, probably due to their late characterization. Intriguingly, both genes are related to gametogenesis, with recent association and clinical studies underscoring their functional importance. In particular, the expression of *STPG2* has been detected in male tissues, endocrine tissues, as well as the brain (Uhlén et al., 2015). Structural mutations deleting this gene have been linked to azoospermia (Yakut et al., 2013) and velocardiofacial syndrome (Wu et al., 2019), and association studies of SNPs in this have correlated it with autism (Connolly et al., 2017) and preclampsia (Johnson et al., 2012). A recent study even reported footprints of ongoing positive selection on a segregating preclampsia-associated SNP in this gene (Arthur, 2018). Note that these authors only analyzed the disease-associating variants and applied haplotype-based selection tests, which tend to reveal regions with at least one dominant haplotype. The footprints reported by Arthur (2018) can result from either recent partial sweeps or balancing selection, with only the latter matching the kilobase-scale size of the increased diversity surrounding the region (Figures S31 and S32).

The conjoined gene *CCDC169-SOHLH2* encodes a read-through transcript of the gene *CCDC169* and its immediate downstream *SOHLH2*, a crucial gene for gametogenesis. In addition to its potential to initiate the transcription of *SOHLH2* on occasions of read-through, *CCDC169* has also been found to have specific expression in pre-natal brain tissues (Pletikos et al., 2014). More interestingly, the B_2 scores across this gene do not form a typical peak as seen in many other candidate regions (Figures S33 and S34). Instead, we observed a plateau of elevated B_2 scores above the region joining the two genes. Furthermore, both the mean pairwise sequence difference (π) and T_2 with a 22-informative-site-radius window show two minor peaks across this region. Considering our results for multi-locus balancing selection (Figure S58), such footprints may be reflective of multiple loci undergoing balancing selection, probably interactively via epistasis, which can create footprints of extended tracks of elevated genetic diversity (Barton and Navarro, 2002; Navarro and Barton, 2002).

Lastly, despite the intriguing functional implications behind our candidates, we are aware that some of our candidate regions show worrying signs for artifacts. For example, *STPG2* (also a top candidate in the scan by DeGiorgio et al., 2014) has low 35-mer sequence uniqueness scores across the whole 40 kb region examined, despite surviving the 50-mer mappability filter. The peak linking *CCDC169* and *SOHLH2* shows overall higher sequence uniqueness than *STPG2*, but the few regions with relatively lower uniqueness co-localize with the peaks reported by π and T_2. This co-localization is also observed in the gene *CPE*, where peak regions with a drop in sequence uniqueness also display lower sequencing depths than other regions. Though not all regions with low mappability necessarily yield outstanding scores for balancing selection, these signs could still be indicative of erroneous mapping and warrant further investigation and caution in interpretation.

Footprints of balancing selection in bonobos and their implications

As one of the two sister species to humans, bonobos (initially known as the pygmy chimpanzees; Prüfer et al., 2012) have been drawing increasing attention from the genomics community (*e.g.*, Prüfer et al., 2012; Prado-Martinez et al., 2013; de Manuel et al., 2016). However, compared with chimpanzees (the other sister species), bonobos are relatively understudied, despite their close relationship to humans and unique social behaviors. For bonobos, one of their most idiosyncratic traits is their high prevalence of sociosexual activities (de Waal, 1990; Kano, 1992; Wrangham, 1993), which serve important non-reproductive functions and include frequent same-sex encounters. As a close relative to humans, their female-dominance, low-aggression, and hypersexual social behaviors contrast fiercely with those of humans and chimpanzees (Kano, 1992; Wrangham, 1993). A growing number of recent studies have also characterized the differences in physiological responses between bonobos and chimpanzees behind their social behaviors (Heilbronner et al., 2008; Hohmann et al., 2009; Wobber et al., 2010; Deschner et al., 2012; Surbeck et al., 2012), yet the

genetic component underlying their unique behaviors, however, remains largely elusive. From the B_2 scan of bonobo genomes, we identified a number of interesting top candidates involved in pathogen defense. Despite that most of the MHC region was removed by a mappability filter (see *Methods*), we still observed extraordinary signals from the remainder of this region. More specifically, the *MHC-DQ* and *MHC-DP* genes harbor the highest peaks across the genome (Figures 4 and 5). These genes encode the component proteins of MHC-DQ and MHC-DP molecules, which are cell-surface receptors on antigen-presenting cells (Ball and Stastny, 1984), and has long been known to be highly polymorphic in great apes (Takahata et al., 1992; Prüfer et al., 2012; Teixeira et al., 2015).

Another immune-related gene, *KLRD1*, which encodes the cell surfacr antigen CD94, also exhibited outstanding B_2 scores. The interaction between KLRD1 (CD94) and NKG2 family proteins can either inhibit or activate the cytotoxic activity of NK cells (Pende et al., 1997; Cantoni et al., 1998; Masilamani et al., 2006), as well as pivot the generation of cell memory in NK cells (Cerwenka and Lanier, 2016). Furthermore, KLRD1 (CD94) has been shown to play an important role in combating viral infections such as cytomegalovirus (CMV; Cerwenka and Lanier, 2016) and influenza (Bongen et al., 2018) in humans, as well as the mousepox virus in mice (Fang et al., 2011). In humans and chimpanzees, *KLRD1* is highly conserved (Khakoo et al., 2000; Shum et al., 2002). Here, the involvement in viral defense of *KLRD1* presents an especially intriguing case for bonobos. Bonobos have been recently shown to harbor reduced levels of polymorphism in MHC class I genes (Maibach et al., 2017; Wroblewski et al., 2017), which were further predicted to have lower ability to bind with viral peptides when compared with chimpanzees (Maibach and Vigilant, 2019). The genes encoding another regulator of MHC class I molecules, the Killer cell Immunoglobin-like Receptors (KIR), were also found to have contracted haplotypes in bonobos (Rajalingam et al., 2001; Walter, 2014; Wroblewski et al., 2019), with the lineage III *KIR* genes serving reduced functions (Wroblewski et al., 2019). In fact, many studies have pointed out that these reduced features are unlikely the natural consequences of demographic factors—even after considering the harsher bottlenecks bonobos have undergone compared with chimpanzees—and speculate that selective sweeps in bonobos on these regions (Prüfer et al., 2012; Walter, 2014; Maibach et al., 2017; Wroblewski et al., 2017, 2019) may have eliminated the diversity in these critical immunity genes. In this light, the polymorphisms on *KLRD1* may be compensating the reduced diversity in their binding partners in bonobos.

Several other genes in high-scoring regions are also found to be involved in immunity. For one, the highest peak on chromosome 7 encompasses the entire gene *GPNMB* (Figure S40), with elevated B_2 scores particularly on exons. This gene encodes osteoactivin, a transmembrane glycoprotein found on osteoclast cells, macrophages, and melanoblast (Loftus et al., 2009; Yu et al., 2016), and is shown to regulate proinflammatory responses (Ripoll et al., 2007). Aside from its heavy involvement in cancer (Zhou et al., 2012), the protein GPNMB has also been shown to facilitate tissue repair (Li et al., 2010; Rose et al., 2010; Hu et al., 2013) as well as influence iris pigmentation (Bächner et al., 2002; Maric et al., 2013). Other potential evidence for balancing selection operating on innate immunity-related genes includes the high B_2 scores observed around the intergenic region between *BPIFB4* and *BPIFA2* (Figure S42), which encode two Bacterialcidal Permeability-Increasing Fold-containing (BPIF) family proteins (Levy, 2000). The *BPIFA2* genic region is recently shown to harbor many SNPs significantly associated with enteropathy (Fujimori et al., 2019), whereas the *BPIFB4* gene is better-known by its association with longevity (Villa et al., 2015b; Spinetti et al., 2017; Villa et al., 2018), speculated to partly result from its protection of vascular functions (Villa et al., 2015a; Puca et al., 2016; Spinelli et al., 2017).

In addition to pathogen defense, we also found other interesting candidates relating to neurosensory and neurodevelopment. One such gene is *SCN9A* (Figure S43), which encodes $\mathrm{Na_V}1.7$, a voltage-gated sodium channel, with mutations on the gene associated with various pain disorders (Yang et al., 2004; Cox et al., 2006; Reimann et al., 2010). The peak we observe covers the overlapping RNA gene encoding its anti-sense transcript, *SCN1A-AS1*, which regulates the expression of *SCN9A* (Koenig et al., 2015), suggestive of diversified regulation of pain perception in bonobos. A few other candidate genes are also involved in neurodevelopment, such as *EPHA6* (Das et al., 2016), *SUSD2* (Figure S47; Nadjar et al., 2015), and *HPCAL1* (Tam, 2015).

Lastly, we noticed that some candidate genes carry multiple distinct functions, and may have been undergoing balancing selection due to potential evolutionary conflicts between some of their functions. For example, the gene *GPNMB* plays roles not only in tissue repair (Li et al., 2010), but also in iris pigmentation (Bächner et al., 2002). Another candidate, *PDE1A* gene (Figure S45), encodes a phosphodiesterase that is pivotal to Ca^{2+}- and cyclic nucleotide-signaling (Lefièvre et al., 2002). It is expressed in brain, endocrine tissues, kidneys, and gonads (Uhlén et al., 2015), and has multiple splicing variants. In fact, the high-scoring peak we observed on this gene happens to locate around the exons that are spliced out in some variants (Figure S45). Studies have demonstrated the relation of this gene to brain development (Yan et al., 1994), mood and cognitive disorders (Xu et al., 2011; Martinez and Gil, 2013; Pekcec et al., 2018; Betolngar et al., 2019), and hypertension (Kimura et al., 2017). Meanwhile, the PDE1A protein is also a conserved component of mammalian spermatozoa (Lefièvre et al., 2002; Vasta et al., 2005), and is involved in the movement of its flagella. Similarly, the gene *CAMK4* encodes Ca^{2+}- and calmodulin-dependent kinase 4, which also plays important roles in both immunity (Koga and Kawakami, 2018) and spermatogenesis (Wu et al., 2000). The cancer-related protein Sushi-domain containing 2, encoded by *SUSD2* (Watson et al., 2013), not only regulates neurite growth in the brain (Nadjar et al., 2015), but can also be used as a marker molecule for human spermatogonial progenitors (Harichandan et al., 2013). Though it is difficult to judge for these genes which functions may be subject to selective pressures, they nonetheless indicate that pleiotropy can be an important driver of balancing selection.

Concluding remarks

Extant methods for detecting long-term balancing selections are constrained by the pliability of their inferences as a function of genomic window size. In this study, we presented B statistics, a set of composite likelihood ratio statistics based on nested mixture models. We have comprehensively evaluated their performances through simulations and demonstrated their robust high performances over varying window sizes in uncovering genomic loci undergoing balancing selection. Moreover, we showed that even when applied with the least optimal window sizes, the B statistics still exhibit high power comparable to current methods, which operated under optimal window sizes, in uncovering balancing selection of varying age and selection parameters, as well as robust performance under confounding scenarios such as elevated mutation rates, variable recombination rates, and population size changes. We re-examined the 1000 Genomes Project YRI and CEU populations with B_2 statistics, and have recovered well-characterized genes previously-hypothesized to be undergoing long-term balancing selection in humans, such as the HLA-D genes, *ERAP2*, and *CSMD2*. We also characterized previously-reported top candidates *STPG2* and *CCDC169-SOHLH2*, both of which are related to gametogenesis. We further applied the B_2 statistic on the whole-genome polymorphism data of bonobos, and discovered not only the well-established *MHC-DQ* and *MHC-DP* genes, but also novel candidates such as *KLRD1*, *PDE1A*, *SCN9A*, and *SUSD2*, with functional implications in pathogen defense, neuro-development, as well as gamete functions. Moreover, we have extended the B statistics to consider multi-allelic balancing selection, with these extensions demonstrating superior properties to all previous methods for detecting selected loci with more than two balanced alleles. We also extended our bi-allelic modeling framework to better account for potential increases in variability of the allele frequency distribution under balancing selection centered on particular equilibrium allele frequencies. Further, we show that all current methods tend to have higher powers for two-locus balancing selection than for single-locus processes. Lastly, we have implemented these statistics in the open source software `BalLeRMix`, which, along with other key scripts used in this study, can be accessed at `https://github.com/bioXiaoheng/BalLeRMix/`. We have also released the empirical scan results for balancing selection in both humans and bonobos, which can be downloaded at `http://degiorgiogroup.fau.edu/ballermix.html`.

Methods

In this section, we discuss sets of simulations used to evaluate the performances of the B statistics relative to previously-published state-of-the-art approaches (Hudson et al., 1987; DeGiorgio et al., 2014; Siewert and Voight, 2017, 2020; Bitarello et al., 2018). Finally, we describe the application of our B statistics to an empirical bonobo dataset (Prado-Martinez et al., 2013).

Evaluating methods through simulations

We employed the forward-time genetic simulator SLiM (version 3.2; Haller and Messer, 2019) to generate sequences of 50 kb in length evolving with or without balancing selection. Based on the respective levels in humans and other great apes, we assumed a mutation rate of $\mu = 2.5 \times 10^{-8}$ per base per generation (Nachman and Crowell, 2000), and a recombination rate of $r = 10^{-8}$ per base per generation (Payseur and Nachman, 2000). In scenarios with constant population sizes, we set the diploid effective population size as $N = 10^4$. To create baseline genetic variation, each replicate simulation was initiated with a burn-in period of $10N = 10^5$ generations. To speed up simulations, we applied the scaling parameter λ to the number of simulated generations, population size, mutation rate, recombination rate, and selection coefficient, which allows for the generation of the same levels of variation with a speed up in computational time by a factor λ^2. For scenarios based on a model of constant population size, we used $\lambda = 100$. For the demographic models of European humans and bonobos, we used $\lambda = 20$. We simulated 500 replicates for each scenario considered, and sampled 50 haploid lineages from the target population and one lineage from the outgroup in each simulation for downstream analyses.

We simulated data from two other diverged species, under the demographic history inspired by that of humans, chimpanzees (Kumar et al., 2005), and gorillas (Scally et al., 2012). Specifically, the closer and farther outgroups diverged 2.5×10^5 and 4×10^5 generations ago, respectively, which correspond to five million and eight million years ago, assuming a generation time of 20 years.

To evaluate the power of each method to detect balancing selection with varying selective coefficient s, dominance coefficient h, and age, for each combination of s and h, we considered 15 time points at which the selected allele was introduced, ranging from 5×10^4 to 6.5×10^5 generations prior to sampling with time points separated by intervals of 5×10^4 generations. Assuming a generation time of 20 years, these time points are equivalent to $1, 2, 3, \ldots, 15$ million years before sampling. In each scenario, a single selected mutation was introduced at the center of each sequence at the assigned time point, and we only considered simulations where the introduced allele was not lost.

Accelerated mutation rate

To evaluate whether the B statistics are robust to high mutation rates, we applied the methods on simulated sequences evolving neutrally along the same demographic history (Figure S1), but instead with a five-fold higher mutation rate of $5\mu = 1.25 \times 10^{-7}$ per site per generation. To generate sequences with regional increases in mutation rate, we simulated 50 kb sequences with a five-fold higher mutation rate of $5\mu = 1.25 \times 10^{-7}$ per site per generation at the central 10 kb of the sequence, and the surrounding region with the original rate μ.

Recombination rate estimation error

For evaluating the robustness to erroneous estimation of recombination rates, we simulated sequences with uneven recombination maps, and applied the model-based methods with the assumption that the recombination rate is uniform. In particular, we divided the 50 kb sequence into 50 regions of one kb each, and in turns inflate or deflate the recombination rate of each region by m fold, such that the recombination rates of every pair of neighboring regions have a m^2-fold difference. We tested $m = 10$ and $m = 100$ in this study.

Demographic history

To examine the performance of methods under realistic demographic parameters, we considered the demographic histories of a European human population (CEU; Terhorst et al., 2017) and of bonobos (Prado-Martinez et al., 2013). For the human population, we adopted the history of population size changes inferred by SMC++ (Terhorst et al., 2017) that spans 10^5 generations, assuming a mutation rate of $\mu = 1.25 \times 10^{-8}$ per site per generation (assumed when estimating the CEU demographic history in Terhorst et al., 2017), a generation time of 20 years, and a scaling effective size of 10^4 diploids. To account for recombination rate variation, we allowed each simulated replicate to have a uniform recombination rate drawn uniformly at random between $r = 5 \times 10^{-9}$ and $r = 1.5 \times 10^{-8}$ per site per generation. We also simulated an additional population that split from the human population 2.5×10^5 generations ago, which is identical to the outgroup (named O1) in the demographic model depicted in Figure 3A, with an effective size of $N = 10^4$ diploid individuals.

For the bonobo population history, we scaled the PSMC history inferred from the genome of individual A917 (Dzeeta; sample SRS396202) by Prado-Martinez et al. (2013) with a mutation rate of $\mu = 2.5 \times 10^{-8}$ per site per generation, identical to the simulations on the three-population demographic history (Figure 3A). Because the inferred PSMC model provides a specific ratio of the mutation and recombination rates, we set the recombination rate to $r = 2.84 \times 10^{-9}$ per site per generation. To be consistent with the three-population demographic history, we set the population size prior to 71,640 generations ago, which is the maximum time covered by the PSMC inference, to $N = 10^4$ diploid individuals, and had the outgroup split 2.5×10^5 generations ago with the same diploid population size, identical to the outgroup O1 in the three-population demographic history (Figure 2A).

To simulate species with distinct mutation rates, we split the simulation into two stages, with the first stage concerning the sequences in the ancestral species up until the two populations diverge five million years ago. Upon divergence, two separate SLiM simulations are used to distinguish the mutation rates in the target and outgroup populations, and samples are output separately before being integrated in subsequent analyses. We set the target species to mutate at a rate of $\mu = 1.2 \times 10^{-8}$ per site per generation (Scally and Durbin, 2012) after divergence, and the other species (including the ancestral species) evolving with the mutation rate of $\mu = 2.5 \times 10^{-8}$ per site per generation (Nachman and Crowell, 2000). The recombination rate across all simulations is $r = 10^{-8}$ per site per generation (Payseur and Nachman, 2000). For the simulations with constant population sizes, we set the effective size of all populations as $N = 10^4$ diploid individuals, and adopted the scaling parameter $\lambda = 100$. For simulations employing realistic demographic histories, we used $\lambda = 20$, set the effective population size of the ancestral and the outgroup species as $N = 10^4$ diploids (Takahata et al., 1995), and the target species following the demographic history inferred from the CEU human population (Terhorst et al., 2017) for 10^5 generations prior to sampling. Additionally, we set the generation time of the target species to be 25 years (akin to humans; Scally and Durbin, 2012), while for the outgroup and ancestral species we used 20 years (akin to non-human great apes; Prado-Martinez et al., 2013). Consequently, the species divergence occurred 200,000 generations ago for the target species, and 250,000 generations ago for the outgroup.

Three- and four-allelic balancing selection at a single site

To simulate balancing selection on a single site with more than two balanced alleles, we used SLiM3.3 (Haller and Messer, 2019) so that all four nucleotides, instead of binary representations of 0s and 1s, can be incorporated into the simulations. We adopted the same three-species demographic history as illustrated in Figure S1, and simulated sequences of length 50 kb consisting of randomly-generated strings of four nucleotides at the beginning of each replicate, with equal chance of occurrence for each nucleotide. We considered the Jukes-Cantor substitution model and set the between-nucleotide mutation rate as $\mu = 8.3 \times 10^{-9}$ per site per generation, such that the total mutation rate (three times the between-nucleotide mutation rate) is $\mu = 2.49 \times 10^{-8}$ per site per generation—roughly the same as adopted in the bi-allelic balancing selection simulations. We also assumed a uniform recombination rate of $r = 10^{-8}$ per site per

generation (Payseur and Nachman, 2000). At 500,000 generations before sampling, we introduced two, three, or four mutations of distinct nucleotides that have selective coefficient $s = 0.001$ and dominance coefficient $h = 20$. Note that SLiM considers co-localized mutations of distinct types as if they were at different positions, and computes fitness for the individual by multiplying fitness values of each mutation. That is, a diploid individual who is heterozygous at a site harboring two distinct selectively advantageous mutant alleles with parameters $s = 0.001$ and $h = 20$ would have fitness $(1 + hs)(1 + hs) = 1.44$, whereas a homozygote for either selectively advantageous mutation would have fitness $1 + s = 1.001$. At the completion of the simulation, we sampled 25 diploid individuals uniformly at random from each of the sister species (P and O1), and one diploid individual was sampled uniformly at random from species O2, with only one haplotype of this individual being considered as the reference sequence. Only bi-allelic sites were considered in the downstream analysis.

Application to empirical data

Human genomic data from the 1000 Genomes Project

We obtained variant calls from the 1000 Genomes Project dataset (The 1000 Genomes Project Consortium, 2015), which were mapped to human reference genome hg19, and extracted the haplotypes for the CEU and YRI populations. We used the chimpanzee reference genome panTro5 downloaded from the UCSC Genome Browser (Kent et al., 2002; Haeussler et al., 2018) to call ancestral alleles, and only retained mappable monomorphic or bi-allelic polymorphic sites based on the variation in the CEU (or YRI) population together with the chimpanzee reference genome. For mappable sites not included in the variant call dataset, we assumed the site is monomorphic for the hg19 reference genome, and called substitutions accordingly.

To avoid making inference on potentially problematic regions, we applied the RepeatMasker filter and removed segmental duplications, both of which were downloaded from the UCSC Genome Browser (Kent et al., 2002; Haeussler et al., 2018). Genomic regions with mappability 50-mer score (Derrien et al., 2012) lower than 0.9 were discarded as well. Moreover, we performed one-tailed Fisher's exact tests for Hardy-Weinberg equilibrium (Wigginton et al., 2005) on each polymorphic site and removed those with a significant ($p < 10^{-4}$) excess of heterozygous genotypes.

We applied B_2 to each CEU and YRI dataset separately, assuming the human recombination map of the hg19 reference genome (International HapMap Consortium, 2007). We did not fix the window size of these scans, and instead permitted B_2 to optimize over both free parameters A and x. To better compare our results with previous studies, we also applied the T_2 statistic (DeGiorgio et al., 2014) to the same input datasets, adopting window sizes of 22 or 100 informative sites on either side of a test informative site. We also computed sequence diversity π averaged across each five kb window as a reference.

For downstream examination of the mappability of candidate regions, we consulted the 35-mer uniqueness score (UCSC hg19 database; Kent et al., 2002; Haeussler et al., 2018) averaged across each one kb region. Furthermore, we also downloaded the BAM files for each individual in the CEU or YRI population and generated per-base read depths with BEDTools 2.26 (Quinlan, 2014). We then computed sample-wide mean read depths, their standard deviations, and the number of individuals without coverage for each population after merging read depths of all samples with BEDTools. These references further aided in flagging potentially problematic regions that survived initial filters, as they typically feature lower mappability (mean 35-mer uniqueness) or abnormally low or high read depths.

Bonobo genomic data from the Great Ape Project

We obtained the genotype calls of 13 bonobos from the Great Ape Project (Prado-Martinez et al., 2013), which were originally mapped to human genome assembly NCBI36/hg18. We lifted over the variant calls to human genome assembly GRCh38/hg38, so that the bonobo genome assembly panPan2 can be used for polarizing the allele frequencies, with the sequence in hg38 considered as the ancestral allele. Only genomic regions mappable across hg38 and panPan2 were considered for further analyses. For mappable

polymorphic sites, we only considered bi-allelic SNPs. For mappable sites without variant calls in bonobo, we assumed these sites were monomorphic for the panPan2 reference genome sequence, and called substitutions based on whether the panPan2 reference allele was different from the hg38 reference allele.

To circumvent potential artifacts, we performed one-tailed Hardy-Weinberg equilibrium tests on each site and removed sites with an excess of heterozygotes ($p < 0.01$). This p-value was determined by the distribution of the p-values of all polymorphic sites across the genome, such that 0.035% of such sites are outliers. We also applied the RepeatMasker, segmental duplication, simple repeat, and interrupted repeat filters (all downloaded from UCSC Genome Browser) to remove repetitive regions. To assess the mappability of each genomic region, we employed the mappability scores (obtained by setting the maximum mismatches tolerated to zero; Derrien et al., 2012) of 50-mers. Regions with 50-mer mappability scores lower than 0.9 were removed. Because `BalLeRMix` employs a pre-specified grid of A values to accompany the distances d in centi-Morgans (cM), when applying the method, we assumed a uniform recombination rate of 10^{-6} cM per site, which is the approximate recombination rate in humans (Payseur and Nachman, 2000).

Acknowledgments

We thank J. Terhorst and J. Prado-Martinez for sharing the inferred parameters for the demographic history of CEU and great apes, repectively. We thank P. Ribeca and T. Derrien for providing the up-to-date GEMtools software and the ENCODE mappability tracks for human hg38 genome assembly after adjusting for indels. We appreciate the assistance from B. Haller on the fitness scheme of multi-allelic balancing selection with SLiM. We thank Hillary Koch for helpful discussions on the properties of composite likelihood ratio statistics. We are also grateful to the editor and anonymous reviewers for their comments that helped improve this manuscript. This work was funded by Pennsylvania State University, by National Institutes of Health grant R35-GM128590, by National Science Foundation grants DEB-1753489, DEB-1949268, and BCS-2001063, and by the Alfred P. Sloan Foundation. Portions of this research were conducted with Advanced CyberInfrastructure computational resources provided by the Institute for CyberScience at Pennsylvania State University.

References

A. M. Andrés. *Balancing Selection in the Human Genome.* John Wiley and Sons, Ltd, 2001.

A. M. Andrés, M. J. Hubisz, A. Indap, D. G. Torgerson, J. D. Degenhardt, A. R. Boyko, R. N. Gutenkunst, T. J. White, E. D. Green, C. D. Bustamante, A. G. Clark, and R. Nielsen. Targets of balancing selection in the human genome. *Molecular Biology and Evolution*, 26(12):2755, 2009.

A. M. Andrés, M. Y. Dennis, W. W. Kretzschmar, J. L. Cannons, S.-Q. Lee-Lin, B. Hurle, P. L. Schwartzberg, S. H. Williamson, C. D. Bustamante, R. Nielsen, et al. Balancing selection maintains a form of erap2 that undergoes nonsense-mediated decay and affects antigen presentation. *PLoS genetics*, 6(10):e1001157, 2010.

A. Arthur. Molecular evolution of genes associated with preeclampsia: Genetic conflict, antagonistic coevolution and signals of selection. *Journal of Evolutionary Medicine*, 6(1):1–9, 2018.

M. Asmussen and E. Basnayake. Frequency-dependent selection: the high potential for permanent genetic variation in the diallelic, pairwise interaction model. *Genetics*, 125(1):215–230, 1990.

M. A. Asmussen and M. W. Feldman. Density dependent selection 1: A stable feasible equilibrium may not be attainable. *Journal of Theoretical Biology*, 64(4):603–618, 1977.

D. Bächner, D. Schröder, and G. Gross. mRNA expression of the murine glycoprotein (transmembrane) NMB (*GPNMB*) gene is linked to the developing retinal pigment epithelium and iris. *Gene Expression Patterns*, 1(3-4):159–165, 2002.

E. Ball and P. Stastny. Antigen-specific HLA-restricted human T-cell lines. II. A GAT-specific T-cell line restricted by a determinant carried by an HLA-DQ molecule. *Immunogenetics*, 20(5):547564, 1984. ISSN 0093-7711. doi: 10.1007/bf00364357.

M. Bamshad and S. P. Wooding. Signatures of natural selection in the human genome. *Nature Reviews Genetics*, 4(2):99, 2003.

N. H. Barton and A. Navarro. Extending the coalescent to multilocus systems: the case of balancing selection. *Genetics Research*, 79(2):129–140, 2002.

Y. Benjamini and Y. Hochberg. Controlling the false discovery rate: a practical and powerful approach to multiple testing. *Journal of the Royal statistical society: series B (Methodological)*, 57(1):289–300, 1995.

A. O. Bergland, E. L. Behrman, K. R. O'Brien, P. S. Schmidt, and D. A. Petrov. Genomic evidence of rapid and stable adaptive oscillations over seasonal time scales in drosophila. *PLoS Genetics*, 10(11): e1004775, 2014.

D. B. Betolngar, É. Mota, A. Fabritius, J. Nielsen, C. Hougaard, C. T. Christoffersen, J. Yang, J. Kehler, O. Griesbeck, L. R. Castro, et al. Phosphodiesterase 1 bridges glutamate inputs with no-and dopamine-induced cyclic nucleotide signals in the striatum. *Cerebral Cortex*, 2019.

B. D. Bitarello, C. de Filippo, J. C. Teixeira, J. M. Schmidt, P. Kleinert, D. Meyer, and A. M. Andrs. Signatures of long-term balancing selection in human genomes. *Genome Biology and Evolution*, 10(3): 939–955, 2018.

C. E. Bonferroni. Il calcolo delle assicurazioni su gruppi di teste. *Studi in onore del professore salvatore ortu carboni*, pages 13–60, 1935.

E. Bongen, F. Vallania, P. J. Utz, and P. Khatri. Klrd1-expressing natural killer cells predict influenza susceptibility. *Genome Medicine*, 10(1):45, 2018.

J. M. Braverman, R. R. Hudson, N. L. Kaplan, C. H. Langley, and W. Stephan. The hitchhiking effect on the site frequency spectrum of dna polymorphisms. *Genetics*, 140(2):783–796, 1995.

C. Cantoni, R. Biassoni, D. Pende, S. Sivori, L. Accame, L. Pareti, G. Semenzato, L. Moretta, A. Moretta, and C. Bottino. The activating form of CD94 receptor complex: CD94 covalently associated with the Kp39 protein that represents the product of the *NKG2-C* gene. *European Journal of Immunology*, 28 (1):327–338, 1998.

A. Cerwenka and L. L. Lanier. Natural killer cell memory in infection, inflammation and cancer. *Nature Reviews Immunology*, 16(2):112, 2016.

B. Charlesworth and D. Charlesworth. *Elements of evolutionary genetics*. Roberts and Company Publishers Greenwood Village, 2010.

D. Charlesworth. Balancing selection and its effects on sequences in nearby genome regions. *PLoS Genetics*, 2(4):1–6, 04 2006.

D. Charlesworth, P. Awadalla, B. Mable, and M. Schierup. Population-level studies of multiallelic self-incompatibility loci, with particular reference to brassicaceae. *Annals of Botany*, 85:227–239, 2000.

X. Cheng and M. DeGiorgio. Detection of shared balancing selection in the absence of trans-species polymorphism. *Molecular Biology and Evololution*, 36:177–199, 2019.

S. Cho, Z. Y. Huang, D. R. Green, D. R. Smith, and J. Zhang. Evolution of the complementary sex-determination gene of honey bees: Balancing selection and trans-species polymorphisms. *Genome Research*, 16(11):1366–1375, 2006.

C. C. Cockerham, P. Burrows, S. Young, and T. Prout. Frequency-dependent selection in randomly mating populations. *The American Naturalist*, 106(950):493–515, 1972.

S. Connolly, R. Anney, L. Gallagher, and E. A. Heron. A genome-wide investigation into parent-of-origin effects in autism spectrum disorder identifies previously associated genes including shank3. *European Journal of Human Genetics*, 25(2):234, 2017.

J. J. Cox, F. Reimann, A. K. Nicholas, G. Thornton, E. Roberts, K. Springell, G. Karbani, H. Jafri, J. Mannan, Y. Raashid, et al. An SCN9A channelopathy causes congenital inability to experience pain. *Nature*, 444(7121):894, 2006.

G. Das, Q. Yu, R. Hui, K. Reuhl, N. W. Gale, and R. Zhou. Epha5 and epha6: regulation of neuronal and spine morphology. *Cell & bioscience*, 6(1):48, 2016.

N. G. de Groot, C. M. Heijmans, P. Helsen, N. Otting, Z. Pereboom, J. M. Stevens, and R. E. Bontrop. Limited MHC class I intron 2 repertoire variation in bonobos. *Immunogenetics*, 69(10):677–688, 2017.

M. de Manuel, M. Kuhlwilm, P. Frandsen, V. C. Sousa, T. Desai, J. Prado-Martinez, J. Hernandez-Rodriguez, I. Dupanloup, O. Lao, P. Hallast, J. M. Schmidt, J. M. Heredia-Genestar, A. Benazzo, G. Barbujani, B. M. Peter, L. F. K. Kuderna, F. Casals, S. Angedakin, M. Arandjelovic, C. Boesch, H. Kühl, L. Vigilant, K. Langergraber, J. Novembre, M. Gut, I. Gut, A. Navarro, F. Carlsen, A. M. Andrés, H. R. Siegismund, A. Scally, L. Excoffier, C. Tyler-Smith, S. Castellano, Y. Xue, C. Hvilsom, and T. Marques-Bonet. Chimpanzee genomic diversity reveals ancient admixture with bonobos. *Science*, 354(6311):477–481, 2016. doi: 10.1126/science.aag2602.

F. B. de Waal. Sociosexual behavior used for tension regulation in all age and sex combinations among bonobos. In *Pedophilia*, pages 378–393. Springer, 1990.

M. DeGiorgio, K. E. Lohmueller, and R. Nielsen. A Model-Based Approach for Identifying Signatures of Ancient Balancing Selection in Genetic Data. *PLoS Genetics*, 10(8):e1004561, 2014.

T. Derrien, J. Estellé, S. M. Sola, D. G. Knowles, E. Raineri, R. Guigó, and P. Ribeca. Fast computation and applications of genome mappability. *PLoS One*, 7(1):e30377, 2012.

T. Deschner, B. T. Fuller, V. M. Oelze, C. Boesch, J.-J. Hublin, R. Mundry, M. P. Richards, S. Ortmann, and G. Hohmann. Identification of energy consumption and nutritional stress by isotopic and elemental analysis of urine in bonobos (pan paniscus). *Rapid Communications in Mass Spectrometry*, 26(1):69–77, 2012.

M. Fang, M. T. Orr, P. Spee, T. Egebjerg, L. L. Lanier, and L. J. Sigal. CD94 is essential for NK cell-mediated resistance to a lethal viral disease. *Immunity*, 34(4):579–589, 2011.

J. C. Fay and C.-I. Wu. Hitchhiking under positive darwinian selection. *Genetics*, 155(3):1405–1413, 2000.

S. Fujimori, K. Fukunaga, A. Takahashi, T. Mushiroda, M. Kubo, R. Hanada, M. Hayashida, T. Sakurai, K. Iwakiri, and C. Sakamoto. Bactericidal/Permeability-Increasing Fold-Containing Family B member 4 may be associated with NSAID-induced enteropathy. *Digestive Diseases and Sciences*, 64(2):401–408, 2019.

M. Fumagalli, R. Cagliani, U. Pozzoli, S. Riva, G. P. Comi, G. Menozzi, N. Bresolin, and M. Sironi. Widespread balancing selection and pathogen-driven selection at blood group antigen genes. *Genome research*, 19(2):199–212, 2009.

Z. Gao, M. Przeworski, and G. Sella. Footprints of ancient-balanced polymorphisms in genetic variation data from closely related species. *Evolution*, 69(2):431–446, 2015.

L. R. Ginzburg. The equilibrium and stability for n alleles under the density-dependent selection. *Journal of theoretical biology*, 68(4):545–550, 1977.

S. Gravel, B. M. Henn, R. N. Gutenkunst, A. R. Indap, G. T. Marth, A. G. Clark, F. Yu, R. A. Gibbs, C. D. Bustamante, . G. Project, et al. Demographic history and rare allele sharing among human populations. *Proceedings of the National Academy of Sciences*, 108(29):11983–11988, 2011.

GTEx Consortium. Genetic effects on gene expression across human tissues. *Nature*, 550(7675):204, 2017.

S. Guirao-Rico, A. Sánchez-Gracia, and D. Charlesworth. Sequence diversity patterns suggesting balancing selection in partially sex-linked genes of the plant silene latifolia are not generated by demographic history or gene flow. *Molecular Ecology*, 26(5):1357–1370, 2017.

M. Haeussler, A. S. Zweig, C. Tyner, M. L. Speir, K. R. Rosenbloom, B. J. Raney, C. M. Lee, B. T. Lee, A. S. Hinrichs, J. N. Gonzalez, D. Gibson, M. Diekhans, H. Clawson, J. Casper, G. P. Barber, D. Haussler, R. M. Kuhn, and W. J. Kent. The UCSC Genome Browser database: 2019 update. *Nucleic Acids Research*, 47(D1):D853–D858, 11 2018. ISSN 0305-1048.

B. C. Haller and P. W. Messer. Slim 3: Forward genetic simulations beyond the wrightfisher model. *Molecular Biology and Evolution*, 36:632–637, 2019.

B. Hare, V. Wobber, and R. Wrangham. The self-domestication hypothesis: evolution of bonobo psychology is due to selection against aggression. *Animal Behaviour*, 83(3):573–585, 2012.

A. Harichandan, K. Sivasubramaniyan, J. Hennenlotter, C. Schwentner, A. Stenzl, and H.-J. Bühring. Isolation of adult human spermatogonial progenitors using novel markers. *Journal of molecular cell biology*, 5(5):351–353, 2013.

P. W. Hedrick. Pathogen resistance and genetic variation at mhc loci. *Evolution*, 56(10):1902–1908, 2002.

S. R. Heilbronner, A. G. Rosati, J. R. Stevens, B. Hare, and M. D. Hauser. A fruit in the hand or two in the bush? divergent risk preferences in chimpanzees and bonobos. *Biology Letters*, 4(3):246–249, 2008.

J. Hey. A multi-dimensional coalescent process applied to multi-allelic selection models and migration models. *Theoretical population biology*, 39(1):30–48, 1991.

G. Hohmann, R. Mundry, and T. Deschner. The relationship between socio-sexual behavior and salivary cortisol in bonobos: tests of the tension regulation hypothesis. *American Journal of Primatology: Official Journal of the American Society of Primatologists*, 71(3):223–232, 2009.

X. Hu, P. Zhang, Z. Xu, H. Chen, and X. Xie. GPNMB enhances bone regeneration by promoting angiogenesis and osteogenesis: potential role for tissue engineering bone. *Journal of Cellular Biochemistry*, 114(12):2729–2737, 2013.

R. R. Hudson and N. L. Kaplan. The coalescent process in models with selection and recombination. *Genetics*, 120(3):831–840, 1988.

R. R. Hudson, M. Kreitman, and M. Aguadé. A test of neutral molecular evolution based on nucleotide data. *Genetics*, 116(1):153–159, 1987.

H. Hunter-Zinck and A. G. Clark. Aberrant time to most recent common ancestor as a signature of natural selection. *Molecular Biology and Evolution*, 32(10):2784–2797, 2015.

International HapMap Consortium. A second generation human haplotype map of over 3.1 million snps. *Nature*, 449(7164):851, 2007.

M. P. Johnson, S. P. Brennecke, C. E. East, H. H. Göring, J. W. Kent Jr, T. D. Dyer, J. M. Said, L. T. Roten, A.-C. Iversen, L. J. Abraham, et al. Genome-wide association scan identifies a risk locus for preeclampsia on 2q14, near the inhibin, beta B gene. *PloS one*, 7(3):e33666, 2012.

T. Kano. *The last ape: Pygmy chimpanzee behavior and ecology*, volume 155. Stanford University Press Stanford, 1992.

N. L. Kaplan, T. Darden, and R. R. Hudson. The coalescent process in models with selection. *Genetics*, 120(3):819–829, 1988.

W. J. Kent, C. W. Sugnet, T. S. Furey, K. M. Roskin, T. H. Pringle, A. M. Zahler, and D. Haussler. The Human Genome Browser at UCSC. *Genome Research*, 12(6):996–1006, 2002.

S. I. Khakoo, R. Rajalingam, B. P. Shum, K. Weidenbach, L. Flodin, D. G. Muir, F. Canavez, S. L. Cooper, N. M. Valiante, L. L. Lanier, et al. Rapid evolution of NK cell receptor systems demonstrated by comparison of chimpanzees and humans. *Immunity*, 12(6):687–698, 2000.

M. Kimura, Y. Tamura, C. Guignabert, M. Takei, K. Kosaki, N. Tanabe, K. Tatsumi, T. Saji, T. Satoh, M. Kataoka, et al. A genome-wide association analysis identifies pde1a— dnajc10 locus on chromosome 2 associated with idiopathic pulmonary arterial hypertension in a japanese population. *Oncotarget*, 8 (43):74917, 2017.

J. Koenig, R. Werdehausen, J. E. Linley, A. M. Habib, J. Vernon, S. Lolignier, N. Eijkelkamp, J. Zhao, A. L. Okorokov, C. G. Woods, et al. Regulation of $Na_v1.7$: a conserved SCN9A natural antisense transcript expressed in dorsal root ganglia. *PLoS One*, 10(6):e0128830, 2015.

T. Koga and A. Kawakami. The role of camk4 in immune responses. *Modern rheumatology*, 28(2):211–214, 2018.

S. Kumar, A. Filipski, V. Swarna, A. Walker, and S. B. Hedges. Placing confidence limits on the molecular age of the human-chimpanzee divergence. *Proceedings of the National Academy of Sciences*, 102(52): 18842–18847, 2005.

E. M. Leffler, Z. Gao, S. Pfeifer, L. Ségurel, A. Auton, O. Venn, R. Bowden, R. Bontrop, J. D. Wall, G. Sella, P. Donnelly, G. McVean, and M. Przeworski. Multiple instances of ancient balancing selection shared between humans and chimpanzees. *Science*, 339(6127):1578–1582, 2013.

L. Lefièvre, E. de Lamirande, and C. Gagnon. Presence of cyclic nucleotide phosphodiesterases pde1a, existing as a stable complex with calmodulin, and pde3a in human spermatozoa. *Biology of Reproduction*, 67(2):423–430, 2002.

L. Lefièvre, K. N. Jha, E. de Lamirande, P. E. Visconti, and C. Gagnon. Activation of protein kinase a during human sperm capacitation and acrosome reaction. *Journal of Andrology*, 23(5):709–716, 2002.

B. Lehner. Molecular mechanisms of epistasis within and between genes. *Trends in Genetics*, 27(8): 323–331, 2011.

O. Levy. A neutrophil-derived anti-infective molecule: bactericidal/permeability-increasing protein. *Antimicrobial Agents and Chemotherapy*, 44(11):2925–2931, 2000.

B. Li, A. P. Castano, T. E. Hudson, B. T. Nowlin, S.-L. Lin, J. V. Bonventre, K. D. Swanson, and J. S. Duffield. The melanoma-associated transmembrane glycoprotein gpnmb controls trafficking of cellular debris for degradation and is essential for tissue repair. *The FASEB Journal*, 24(12):4767–4781, 2010.

S. K. Loftus, A. Antonellis, I. Matera, G. Renaud, L. L. Baxter, D. Reid, T. G. Wolfsberg, Y. Chen, C. Wang, N. C. S. Program, et al. Gpnmb is a melanoblast-expressed, MITF-dependent gene. *Pigment Cell & Melanoma Research*, 22(1):99–110, 2009.

P.-R. Loh, M. Lipson, N. Patterson, P. Moorjani, J. K. Pickrell, D. Reich, and B. Berger. Inferring admixture histories of human populations using linkage disequilibrium. *Genetics*, 193(4):1233–1254, 2013.

K. E. Lohmueller, C. D. Bustamante, and A. G. Clark. Methods for human demographic inference using haplotype patterns from genomewide single-nucleotide polymorphism data. *Genetics*, 182(1):217–231, 2009.

E. Lonn, E. Koskela, T. Mappes, M. Mokkonen, A. M. Sims, and P. C. Watts. Balancing selection maintains polymorphisms at neurogenetic loci in field experiments. *Proceedings of the National Academy of Sciences*, page 201621228, 2017.

T. F. Mackay. Epistasis and quantitative traits: using model organisms to study gene–gene interactions. *Nature Reviews Genetics*, 15(1):22–33, 2014.

V. Maibach and L. Vigilant. Reduced bonobo MHC class I diversity predicts a reduced viral peptide binding ability compared to chimpanzees. *BMC Evolutionary Biology*, 19(1):14, 2019.

V. Maibach, J. B. Hans, C. Hvilsom, T. Marques-Bonet, and L. Vigilant. MHC class I diversity in chimpanzees and bonobos. *Immunogenetics*, 69(10):661–676, 2017.

G. Maric, A. A. Rose, M. G. Annis, and P. M. Siegel. Glycoprotein non-metastatic b (GPNMB): A metastatic mediator and emerging therapeutic target in cancer. *OncoTargets and therapy*, 6:839, 2013.

A. Martinez and C. Gil. *Phosphodiesterase Inhibitors as a New Therapeutic Approach for the Treatment of Parkinsons.* Royal Society of Chemistry, 2013.

M. Masilamani, C. Nguyen, J. Kabat, F. Borrego, and J. E. Coligan. CD94/NKG2A inhibits NK cell activation by disrupting the actin network at the immunological synapse. *The Journal of Immunology*, 177(6):3590–3596, 2006.

D. Meyer, V. R. Aguiar, B. D. Bitarello, D. Y. Brandt, and K. Nunes. A genomic perspective on hla evolution. *Immunogenetics*, pages 1–23, 2017.

H. Michibata, N. Yanaka, Y. Kanoh, K. Okumura, and K. Omori. Human Ca2+/calmodulin-dependent phosphodiesterase PDE1A: novel splice variants, their specific expression, genomic organization, and chromosomal localization. *Biochimica et Biophysica Acta (BBA)-Gene Structure and Expression*, 1517 (2):278–287, 2001.

T. Mitchell-Olds, J. H. Willis, and D. B. Goldstein. Which evolutionary processes influence natural genetic variation for phenotypic traits? *Nature Reviews Genetics*, 8(11):845–56, 11 2007. Copyright - Copyright Nature Publishing Group Nov 2007; Last updated - 2014-03-31.

P. Moorjani, N. Patterson, J. N. Hirschhorn, A. Keinan, L. Hao, G. Atzmon, E. Burns, H. Ostrer, A. L. Price, and D. Reich. The history of african gene flow into southern europeans, levantines, and jews. *PLoS genetics*, 7(4):e1001373, 2011.

C. A. Muirhead and J. Wakeley. Modeling multiallelic selection using a moran model. *Genetics*, 182(4): 1141–1157, 2009.

M. W. Nachman and S. L. Crowell. Estimate of the mutation rate per nucleotide in humans. *Genetics*, 156(1):297–304, 2000.

Y. Nadjar, A. Triller, J.-L. Bessereau, and A. Dumoulin. The susd2 protein regulates neurite growth and excitatory synaptic density in hippocampal cultures. *Molecular and Cellular Neuroscience*, 65:82–91, 2015.

J. K. Naggert, L. D. Fricker, O. Varlamov, P. M. Nishina, Y. Rouille, D. F. Steiner, R. J. Carroll, B. J. Paigen, and E. H. Leiter. Hyperproinsulinaemia in obese fat/fat mice associated with a carboxypeptidase e mutation which reduces enzyme activity. *Nature genetics*, 10(2):135, 1995.

A. Navarro and N. H. Barton. The effects of multilocus balancing selection on neutral variability. *Genetics*, 161(2):849–863, 2002.

R. Nielsen, S. Williamson, Y. Kim, M. J. Hubisz, A. G. Clark, and C. Bustamante. Genomic scans for selective sweeps using snp data. *Genome research*, 15(11):1566–1575, 2005.

N. A. O'Leary, M. W. Wright, J. R. Brister, S. Ciufo, D. Haddad, R. McVeigh, B. Rajput, B. Robbertse, B. Smith-White, D. Ako-Adjei, et al. Reference sequence (refseq) database at ncbi: current status, taxonomic expansion, and functional annotation. *Nucleic acids research*, 44(D1):D733–D745, 2015.

L. Pace, A. Salvan, and N. Sartori. Adjusting composite likelihood ratio statistics. *Statistica Sinica*, 21 (1):129–148, 2011. ISSN 10170405, 19968507.

B. A. Payseur and M. W. Nachman. Microsatellite variation and recombination rate in the human genome. *Genetics*, 156(3):1285–1298, 2000.

A. Pekcec, N. Schülert, B. Stierstorfer, S. Deiana, C. Dorner-Ciossek, and H. Rosenbrock. Targeting the dopamine D1 receptor or its downstream signalling by inhibiting phosphodiesterase-1 improves cognitive performance. *British Journal of Pharmacology*, 175(14):3021–3033, 2018.

D. Pende, S. Sivori, L. Accame, L. Pareti, M. Falco, D. Geraghty, P. Le Bouteiller, L. Moretta, and A. Moretta. HLA-G recognition by human natural killer cells. Involvement of CD94 both as inhibitory and as activating receptor complex. *European Journal of Immunology*, 27(8):1875–1880, 1997.

M. Pletikos, A. M. Sousa, G. Sedmak, K. A. Meyer, Y. Zhu, F. Cheng, M. Li, Y. I. Kawasawa, and N. Šestan. Temporal specification and bilaterality of human neocortical topographic gene expression. *Neuron*, 81(2):321–332, 2014.

J. Prado-Martinez, P. H. Sudmant, J. M. Kidd, H. Li, J. L. Kelley, B. Lorente-Galdos, K. R. Veeramah, A. E. Woerner, T. D. OConnor, G. Santpere, et al. Great ape genetic diversity and population history. *Nature*, 499(7459):471–475, 2013.

K. Prüfer, K. Munch, I. Hellmann, K. Akagi, J. R. Miller, B. Walenz, S. Koren, G. Sutton, C. Kodira, R. Winer, J. R. Knight, J. C. Mullikin, S. J. Meader, C. P. Ponting, G. Lunter, S. Higashino, A. Hobolth, J. Dutheil, E. Karako, C. Alkan, S. Sajjadian, C. R. Catacchio, M. Ventura, T. Marques-Bonet, E. E. Eichler, C. Andr, R. Atencia, L. Mugisha, J. Junhold, N. Patterson, M. Siebauer, J. M. Good, A. Fischer, S. E. Ptak, M. Lachmann, D. E. Symer, T. Mailund, M. H. Schierup, A. M. Andrs, J. Kelso, and S. Pbo. The bonobo genome compared with the chimpanzee and human genomes. *Nature*, 486(7404):527, 2012.

A. A. Puca, G. Spinetti, R. Vono, C. Vecchione, and P. Madeddu. The genetics of exceptional longevity identifies new druggable targets for vascular protection and repair. *Pharmacological Research*, 114: 169–174, 2016.

A. R. Quinlan. BEDTools: the Swiss-army tool for genome feature analysis. *Current protocols in bioinformatics*, 47(1):11–12, 2014.

R. Rajalingam, M. Hong, E. J. Adams, B. P. Shum, L. A. Guethlein, and P. Parham. Short KIR haplotypes in pygmy chimpanzee (Bonobo) resemble the conserved framework of diverse human KIR haplotypes. *Journal of Experimental Medicine*, 193(1):135–146, 2001.

F. Reimann, J. J. Cox, I. Belfer, L. Diatchenko, D. V. Zaykin, D. P. McHale, J. P. Drenth, F. Dai, J. Wheeler, F. Sanders, et al. Pain perception is altered by a nucleotide polymorphism in *SCN9A*. *Proceedings of the National Academy of Sciences*, 107(11):5148–5153, 2010.

V. M. Ripoll, K. M. Irvine, T. Ravasi, M. J. Sweet, and D. A. Hume. Gpnmb is induced in macrophages by IFN-γ and lipopolysaccharide and acts as a feedback regulator of proinflammatory responses. *The Journal of Immunology*, 178(10):6557–6566, 2007.

A. A. Rose, M. G. Annis, Z. Dong, F. Pepin, M. Hallett, M. Park, and P. M. Siegel. ADAM10 releases a soluble form of the GPNMB/Osteoactivin extracellular domain with angiogenic properties. *PLoS One*, 5(8):e12093, 2010.

N. Saitou and F.-i. Yamamoto. Evolution of primate abo blood group genes and their homologous genes. *Molecular Biology and Evolution*, 14(4):399–411, 1997.

A. Sanchez-Mazas. An apportionment of human hla diversity. *Tissue Antigen*, 69(s1):198–202, 2007.

A. Scally and R. Durbin. Revising the human mutation rate: implications for understanding human evolution. *Nature Reviews Genetics*, 13(10):745, 2012.

A. Scally, J. Y. Dutheil, L. W. Hillier, G. E. Jordan, I. Goodhead, J. Herrero, A. Hobolth, T. Lappalainen, T. Mailund, T. Marques-Bonet, S. McCarthy, S. H. Montgomery, P. C. Schwalie, Y. A. Tang, M. C. Ward, Y. Xue, B. Yngvadottir, C. Alkan, L. N. Andersen, Q. Ayub, E. V. Ball, K. Beal, B. J. Bradley, Y. Chen, C. M. Clee, S. Fitzgerald, T. A. Graves, Y. Gu, P. Heath, A. Heger, E. Karakoc, A. Kolb-Kokocinski, G. K. Laird, G. Lunter, S. Meader, M. Mort, J. C. Mullikin, K. Munch, T. D. O'Connor, A. D. Phillips, J. Prado-Martinez, A. S. Rogers, S. Sajjadian, D. Schmidt, K. Shaw, J. T. Simpson, P. D. Stenson, D. J. Turner, L. Vigilant, A. J. Vilella, W. Whitener, B. Zhu, D. N. Cooper, P. de Jong, E. T. Dermitzakis, E. E. Eichler, P. Flicek, N. Goldman, N. I. Mundy, Z. Ning, D. T. Odom, C. P. Ponting, M. A. Quail, O. A. Ryder, S. M. Searle, W. C. Warren, R. K. Wilson, M. H. Schierup, J. Rogers, C. Tyler-Smith, and R. Durbin. Insights into hominid evolution from the gorilla genome sequence. *Nature*, 483(7388): 169–75, 2012.

L. Ségurel, E. E. Thompson, T. Flutre, J. Lovstad, A. Venkat, S. W. Margulis, J. Moyse, S. Ross, K. Gamble, G. Sella, et al. The abo blood group is a trans-species polymorphism in primates. *Proceedings of the National Academy of Sciences*, 109(45):18493–18498, 2012.

H. Shao, L. C. Burrage, D. S. Sinasac, A. E. Hill, S. R. Ernest, W. O'Brien, H.-W. Courtland, K. J. Jepsen, A. Kirby, E. Kulbokas, et al. Genetic architecture of complex traits: large phenotypic effects and pervasive epistasis. *Proceedings of the National Academy of Sciences*, 105(50):19910–19914, 2008.

S. Sheehan and Y. S. Song. Deep learning for population genetic inference. *PLoS Computational Biology*, 12(3):e1004845, 2016.

B. P. Shum, L. R. Flodin, D. G. Muir, R. Rajalingam, S. I. Khakoo, S. Cleland, L. A. Guethlein, M. Uhrberg, and P. Parham. Conservation and variation in human and common chimpanzee *CD94* and *NKG2* genes. *The Journal of Immunology*, 168(1):240–252, 2002.

K. M. Siewert and B. F. Voight. Detecting long-term balancing selection using allele frequency correlation. *Molecular Biology and Evolution*, 32(11):2996–3005, 2017.

K. M. Siewert and B. F. Voight. Betascan2: Standardized statistics to detect balancing selection utilizing substitution data. *Genome Biology and Evolution*, 12:3873–3877, 2020. doi: 10.1093/gbe/evaa013.

J. Sikela, M. Adamson, D. Wilson-Shaw, and C. Kozak. Genetic mapping of the gene for ca2+ calmodulin-dependent protein kinase iv (camk-4) to mouse chromosome 18. *Genomics*, 8(3):579–582, 1990.

R. J. Simes. An improved Bonferroni procedure for multiple tests of significance. *Biometrika*, 73(3): 751–754, 1986.

C. Smukowski and M. Noor. Recombination rate variation in closely related species. *Heredity*, 107(6):496, 2011.

Y. S. Song and M. Steinrücken. A simple method for finding explicit analytic transition densities of diffusion processes with general diploid selection. *Genetics*, 190(3):1117–1129, 2012.

C. C. Spinelli, A. Carrizzo, A. Ferrario, F. Villa, A. Damato, M. Ambrosio, M. Madonna, G. Frati, S. Fucile, M. Sciaccaluga, et al. LAV-BPIFB4 isoform modulates eNOS signalling through Ca^{2+}/PKC-α-dependent mechanism. *Cardiovascular Research*, 113(7):795–804, 2017.

G. Spinetti, E. Sangalli, C. Specchia, F. Villa, C. Spinelli, R. Pipolo, A. Carrizzo, S. Greco, C. Voellenkle, C. Vecchione, et al. The expression of the BPIFB4 and CXCR4 associates with sustained health in long-living individuals from Cilento-Italy. *Aging (Albany NY)*, 9(2):370, 2017.

M. Surbeck, T. Deschner, G. Schubert, A. Weltring, and G. Hohmann. Mate competition, testosterone and intersexual relationships in bonobos, pan paniscus. *Animal Behaviour*, 83(3):659–669, 2012.

H. Suzuki, H. W. Ahn, T. Chu, W. Bowden, K. Gassei, K. Orwig, and A. Rajkovic. SOHLH1 and SOHLH2 coordinate spermatogonial differentiation. *Developmental biology*, 361(2):301–312, 2012.

C. G. Sweeney, J. M. Rando, H. N. Panas, G. M. Miller, D. M. Platt, and E. J. Vallender. Convergent balancing selection on the mu-opioid receptor in primates. *Molecular Biology and Evolution*, 34(7): 1629–1643, 2017.

F. Tajima. Evolutionary relationship of dna sequences in finite populations. *Genetics*, 105(2):437–460, 1983.

F. Tajima. Statistical method for testing the neutral mutation hypothesis by dna polymorphism. *Genetics*, 123(3):585–595, 1989.

N. Takahata, Y. Satta, and J. Klein. Polymorphism and balancing selection at major histocompatibility complex loci. *Genetics*, 130(4):925–938, 1992.

N. Takahata, Y. Satta, and J. Klein. Divergence time and population size in the lineage leading to modern humans. *Theoretical population biology*, 48(2):198–221, 1995.

A. H. T. Tam. *Characterization of hippocalcin-like protein 1 (HPCAL1), a neuronal calcium sensor protein in the retina.* PhD thesis, University of British Columbia, 2015.

J. C. Teixeira, C. de Filippo, A. Weihmann, J. R. Meneu, F. Racimo, M. Dannemann, B. Nickel, A. Fischer, M. Halbwax, C. Andre, et al. Long-term balancing selection in lad1 maintains a missense trans-species polymorphism in humans, chimpanzees, and bonobos. *Molecular Biology and Evolution*, 32(5):1186–1196, 2015.

J. A. Tennessen. Gene buddies: Linked balanced polymorphisms reinforce each other even in the absence of epistasis. *PeerJ*, 6:e5110, 2018.

J. Terhorst, J. A. Kamm, and Y. S. Song. Robust and scalable inference of population history from hundreds of unphased whole genomes. *Nature Genetics*, 49(2):303, 2017.

The 1000 Genomes Project Consortium. A global reference for human genetic variation. *Nature*, 526(7571): 68–74, 2015.

S. Toyoda, T. Miyazaki, S. Miyazaki, T. Yoshimura, M. Yamamoto, F. Tashiro, E. Yamato, and J.-i. Miyazaki. Sohlh2 affects differentiation of KIT positive oocytes and spermatogonia. *Developmental biology*, 325(1):238–248, 2009.

F. Ubeda and D. Haig. Sex-specific meiotic drive and selection at an imprinted locus. *Genetics*, 167(4): 2083–2095, 2004.

M. Uhlén, L. Fagerberg, B. M. Hallström, C. Lindskog, P. Oksvold, A. Mardinoglu, Å. Sivertsson, C. Kampf, E. Sjöstedt, A. Asplund, et al. Tissue-based map of the human proteome. *Science*, 347 (6220):1260419, 2015.

P. F. van der Ven, E. J. Speel, J. C. Albrechts, F. C. Ramaekers, A. H. Hopman, and D. O. Fürst. Assignment of the human gene for endosarcomeric cytoskeletal M-protein (MYOM2) to 8p23. 3. *Genomics*, 55 (2):253–255, 1999.

C. Varin, N. Reid, and D. Firth. An overview of composite likelihood methods. *Statistica Sinica*, 21(1): 5–42, 2011. ISSN 10170405, 19968507.

V. Vasta, W. K. Sonnenburg, C. Yan, S. H. Soderling, M. Shimizu-Albergine, and J. A. Beavo. Identification of a new variant of pde1a calmodulin-stimulated cyclic nucleotide phosphodiesterase expressed in mouse sperm. *Biology of Reproduction*, 73(4):598–609, 2005.

F. Villa, A. Carrizzo, C. C. Spinelli, A. Ferrario, A. Malovini, A. Maciag, A. Damato, A. Auricchio, G. Spinetti, E. Sangalli, et al. Genetic analysis reveals a longevity-associated protein modulating endothelial function and angiogenesis. *Circulation Research*, 117(4):333–345, 2015a.

F. Villa, A. Malovini, A. Carrizzo, C. C. Spinelli, A. Ferrario, A. Maciag, M. Madonna, R. Bellazzi, L. Milanesi, C. Vecchione, et al. Serum BPIFB4 levels classify health status in long-living individuals. *Immunity & Ageing*, 12(1):27, 2015b.

F. Villa, E. Ciaglia, A. Maciag, F. Montella, A. Ferrario, M. Cattaneo, and A. Puca. Longevity associated variant of *BPIFB4* mitigates monocyte mediated acquired immune response. *Innovation in Aging*, 2 (Suppl 1):884, 2018.

L. Walter. Immunogenetics of NK cell receptors and MHC Class I ligands in non-human primates. In *Natural Hosts of SIV*, pages 269–285. Elsevier, 2014.

A. P. Watson, R. L. Evans, and K. A. Egland. Multiple functions of sushi domain containing 2 (susd2) in breast tumorigenesis. *Molecular Cancer Research*, 11(1):74–85, 2013.

J. E. Wigginton, D. J. Cutler, and G. R. Abecasis. A note on exact tests of hardy-weinberg equilibrium. *The American Journal of Human Genetics*, 76(5):887–893, 2005.

C. Wills. Maintenance of multiallelic polymorphism at the mhc region. *Immunological reviews*, 124:165–220, 1991.

V. Wobber, B. Hare, J. Maboto, S. Lipson, R. Wrangham, and P. T. Ellison. Differential changes in steroid hormones before competition in bonobos and chimpanzees. *Proceedings of the National Academy of Sciences*, 107(28):12457–12462, 2010.

R. W. Wrangham. The evolution of sexuality in chimpanzees and bonobos. *Human Nature*, 4(1):47–79, Mar 1993. ISSN 1936-4776.

E. E. Wroblewski, L. A. Guethlein, P. J. Norman, Y. Li, C. M. Shaw, A. S. Han, J.-B. N. Ndjango, S. Ahuka-Mundeke, A. V. Georgiev, M. Peeters, et al. Bonobos maintain immune system diversity with three functional types of MHC-B. *The Journal of Immunology*, 198(9):3480–3493, 2017.

E. E. Wroblewski, P. Parham, and L. A. Guethlein. Two to tango: Co-evolution of hominid natural killer cell receptors and MHC. *Frontiers in Immunology*, 10, 2019.

D. Wu, Y. Chen, Q. Chen, G. Wang, X. Xu, A. Peng, J. Hao, J. He, L. Huang, and J. Dai. Clinical presentation and genetic profiles of chinese patients with velocardiofacial syndrome in a large referral centre. *Journal of Genetics*, 98(2):42, May 2019.

J. Y. Wu, T. J. Ribar, D. E. Cummings, K. A. Burton, G. S. McKnight, and A. R. Means. Spermiogenesis and exchange of basic nuclear proteins are impaired in male germ cells lacking camk4. *Nature genetics*, 25(4):448–452, 2000.

Y. Xu, H.-T. Zhang, and J. M. ODonnell. Phosphodiesterases in the central nervous system: implications in mood and cognitive disorders. In *Phosphodiesterases as Drug Targets*, pages 447–485. Springer, 2011.

S. Yakut, Z. Cetin, O. A. Clark, M. F. Usta, S. Berker, and G. Luleci. Exceptional complex chromosomal rearrangement and microdeletions at the 4q22. 3q23 and 14q31. 1q31. 3 regions in a patient with azoospermia. *Gene*, 512(1):157–160, 2013.

C. Yan, J. K. Bentley, W. K. Sonnenburg, and J. A. Beavo. Differential expression of the 61 kda and 63 kda calmodulin-dependent phosphodiesterases in the mouse brain. *Journal of Neuroscience*, 14(3): 973–984, 1994.

Y. Yang, Y. Wang, S. Li, Z. Xu, H. Li, L. Ma, J. Fan, D. Bu, B. Liu, Z. Fan, et al. Mutations in *SCN9A*, encoding a sodium channel alpha subunit, in patients with primary erythermalgia. *Journal of Medical Genetics*, 41(3):171–174, 2004.

C. J. Ye, J. Chen, A.-C. Villani, R. E. Gate, M. Subramaniam, T. Bhangale, M. N. Lee, T. Raj, R. Raychowdhury, W. Li, et al. Genetic analysis of isoform usage in the human anti-viral response reveals influenza-specific regulation of erap2 transcripts under balancing selection. *Genome Research*, 28(12): 1812–1825, 2018.

B. Yu, G. R. Sondag, C. Malcuit, M.-H. Kim, and F. F. Safadi. Macrophage-associated Osteoactivin/GPNMB mediates mesenchymal stem cell survival, proliferation, and migration via a cd44-dependent mechanism. *Journal of Cellular Biochemistry*, 117(7):1511–1521, 2016.

L. Zhou, F. Liu, Y. Li, Y. Peng, Y. Liu, and J. Li. Gpnmb/osteoactivin, an attractive target in cancer immunotherapy. *Neoplasma*, 59(1):1–5, 2012.

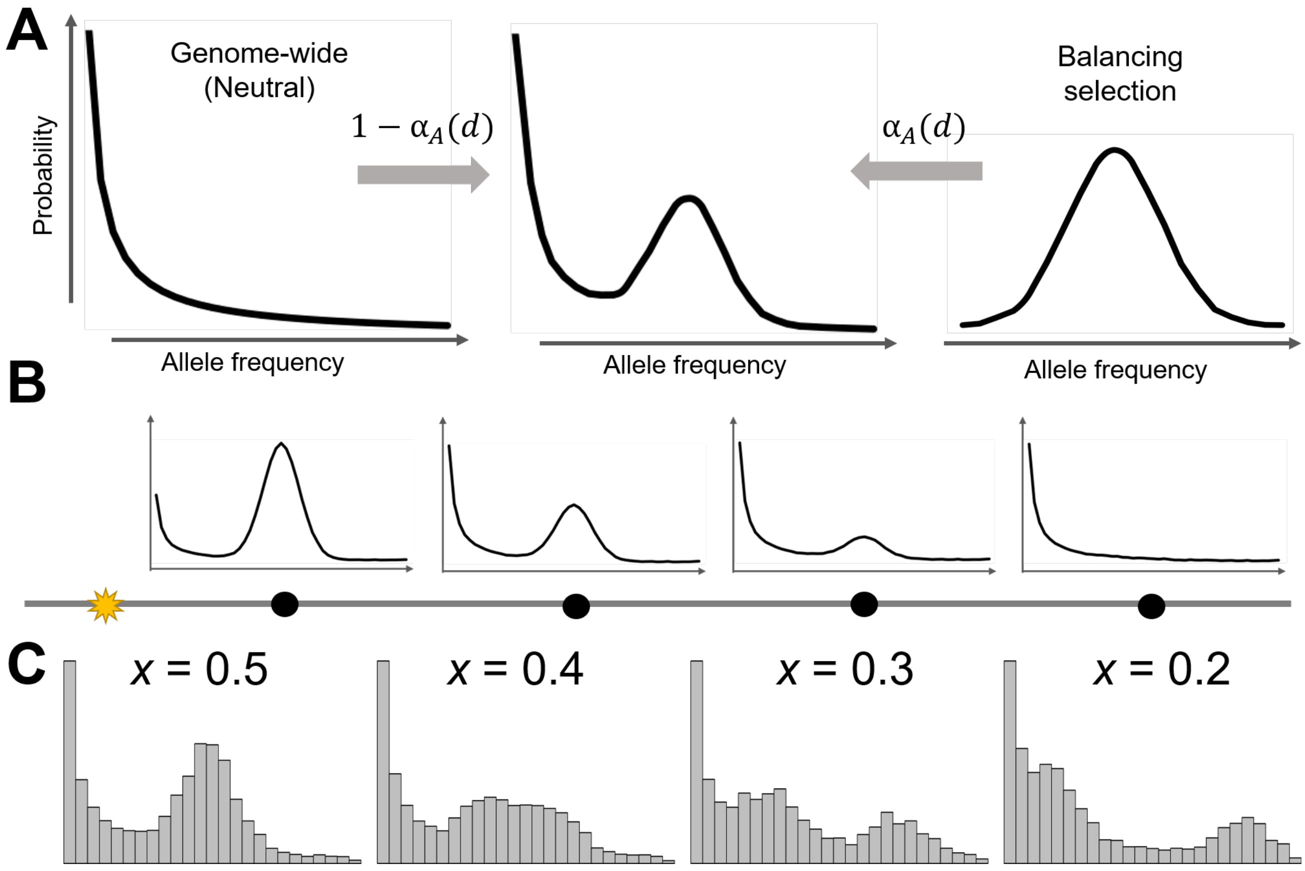

Figure 1: Schematic of the mixture model underlying the B statistics. (A) The model for the alternative hypothesis is a mixture of the distribution of allele frequencies under balancing selection at proportion $\alpha_A(d)$, modeled by a binomial distribution, and the distribution under neutrality at proportion $1-\alpha_A(d)$, modeled by the genome-wide site frequency spectrum. Here, $\alpha_A(d)$ decays as a function of recombination distance d, and so sites close to (*i.e*, small d) the putative selected site will be modeled mostly by the distribution expected under balancing selection, whereas sites far from (*i.e.*, large d) the selected site will be modeled mostly by the distribution expected under neutrality. (B) Distributions of allele frequencies at neutral sites (black dots) under the mixture model at varying distances d from the putative selected site (yellow star). (C) Distributions of allele frequencies from the center 10 kb (0.01 centiMorgan) of the simulated sequences when balancing selection maintains the equilibrium frequency of $x = 0.2$, 0.3, 0.4, or 0.5.

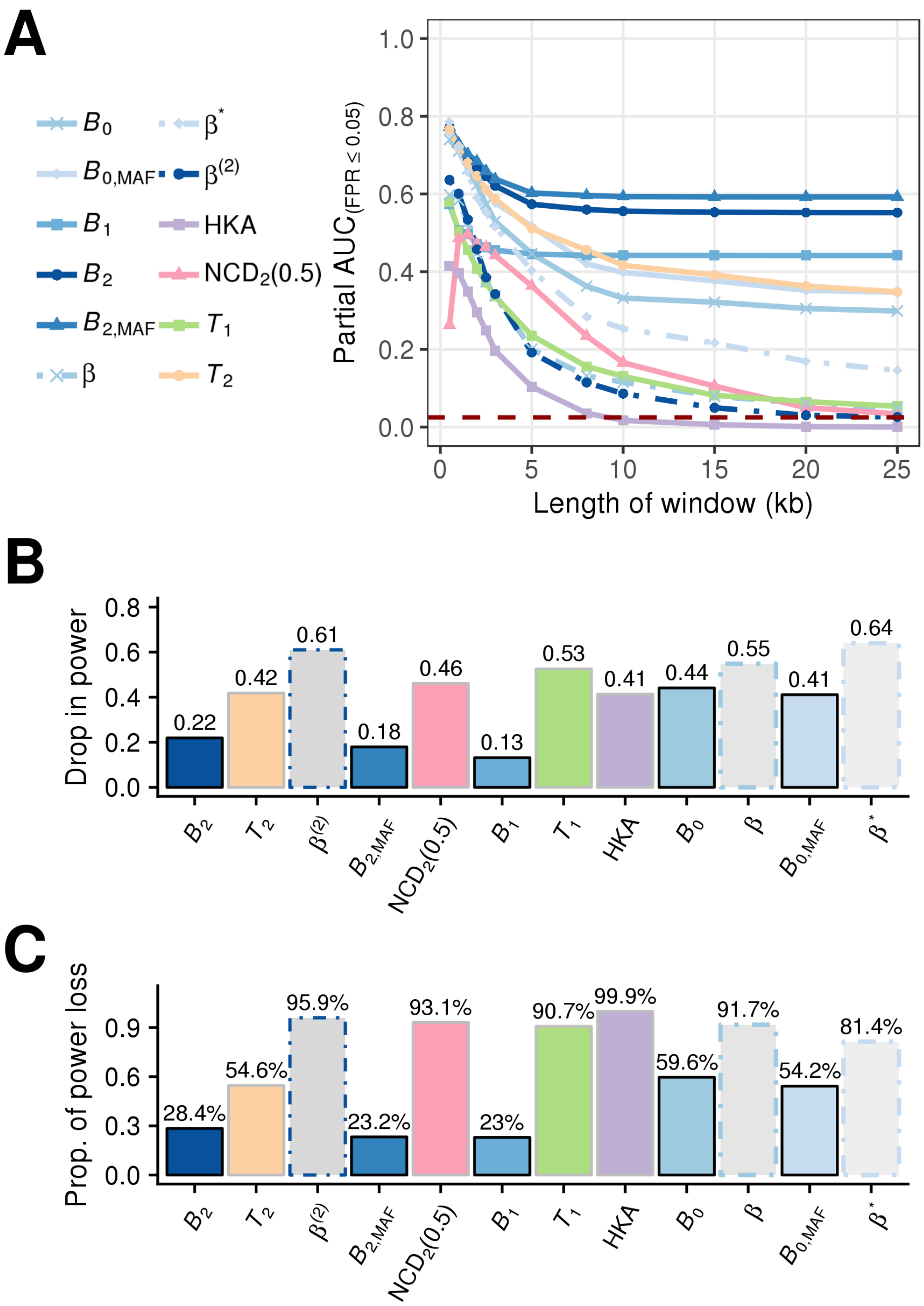

Figure 2: Partial area under the curve (AUC) conditioned on false positive rates (FPRs) less than or equal to 5% (defined such that the maximal value is 1) as a function of window size measured in kilobases (kb) for B statistics (varying shades of blue), β statistics (dotted line with varying shades of blue), T_2 (orange), T_1 (green), HKA (purple), and NCD$_2$(0.5) (pink), under a scenario in which a mutation undergoing ancient balancing selection (selective coefficient $s = 0.01$ and dominance coefficient $h = 20$) arose 15 million years ago (assuming a generation time of 20 years). Statistics that consider the same input type share the same point shape. The dark red dashed line marks the level of partial AUC expected at the y=x line, or the baseline of randomly choosing between balancing selection and neutrality. (B) The amount of partial AUC lost, and (C) the proportion of the AUC loss as compared with the optimal value for each statistic when the window size increased from the optimum to 25 kb (*e.g.*, largest evaluated).

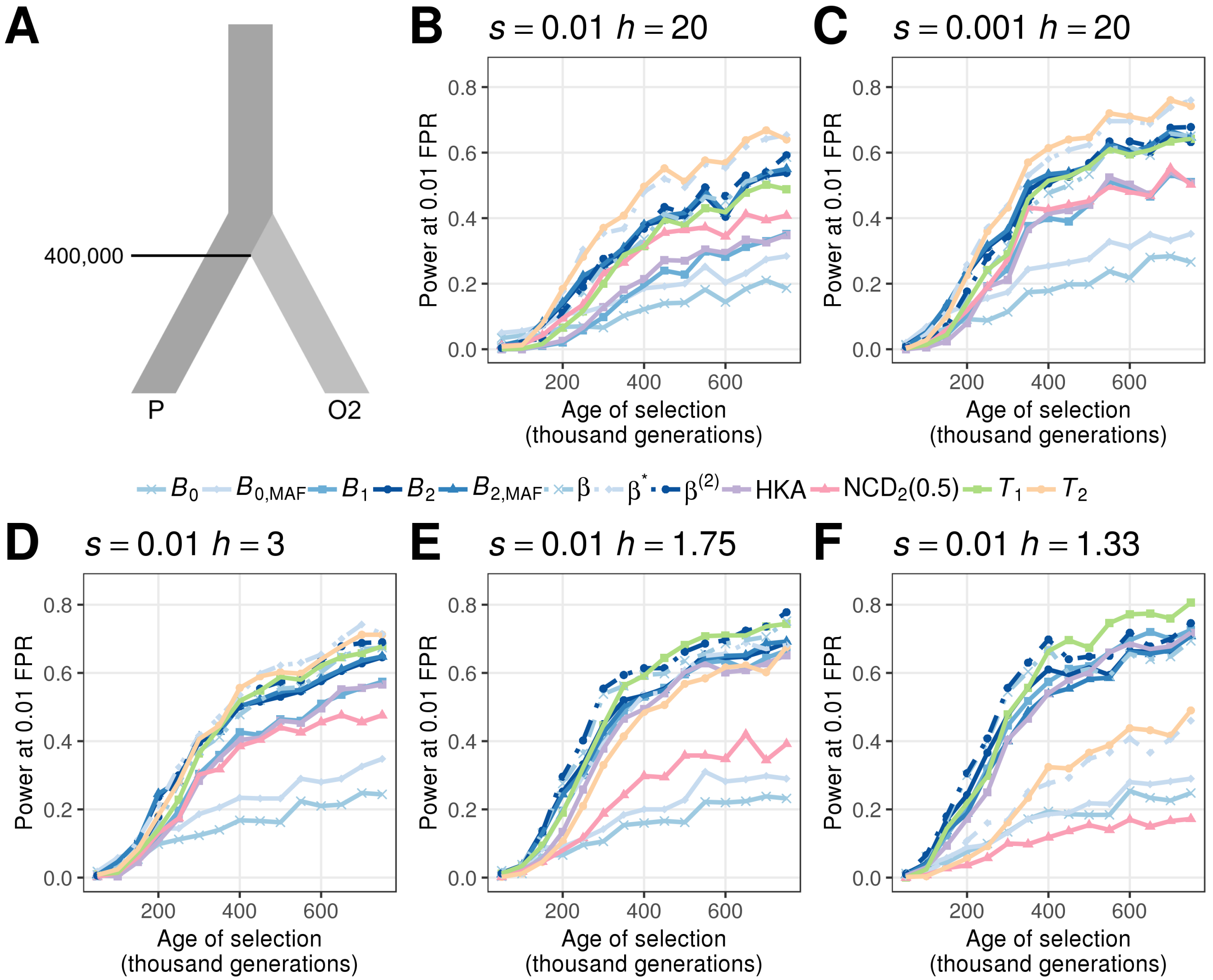

Figure 3: Ability to detect balancing selection for different heterozygote advantage scenarios. (A) Demographic model relating the ingroup (P) and outgroup (O2) populations, with one sample from O2 used as the outgroup sequence. (B-F) Powers at a 1% false positive rate (FPR) for each statistic as a function of age of the allele undergoing balancing selection for different selection (s) and dominance (h) coefficients. The scenarios considered are (B) $s = 0.01$ with $h = 20$, (C) $s = 0.001$ with $h = 20$, (D) $s = 0.01$ with $h = 3$, (E) $s = 0.01$ with $h = 1.75$, and (F) $s = 0.01$ with $h = 1.33$. Note that the equilibrium frequencies for panels D, E, and F are 0.4, 0.3, and 0.2, respectively.

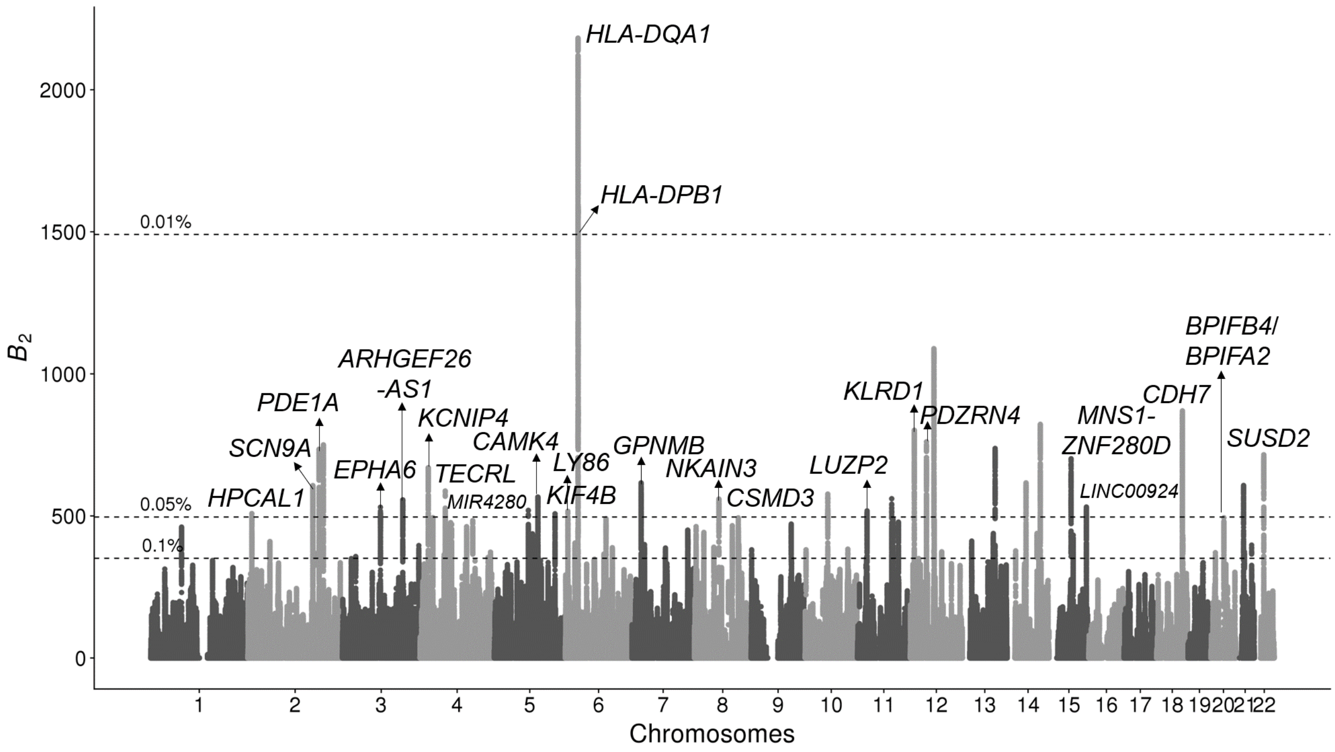

Figure 4: Manhattan plot displaying B_2 scores across the 22 human autosomes for which the bonobo genomic data were mapped, with the candidates scoring in the top 0.05 percent annotated. RNA genes are annotated with smaller fonts. Horizontal dotted lines represent cutoff scores for the top 0.1, 0.05, and 0.01 percent across the genome. Peaks higher than 0.05 percent cutoff but without annotations do not have neighboring protein-coding regions within a 250 kb radius.

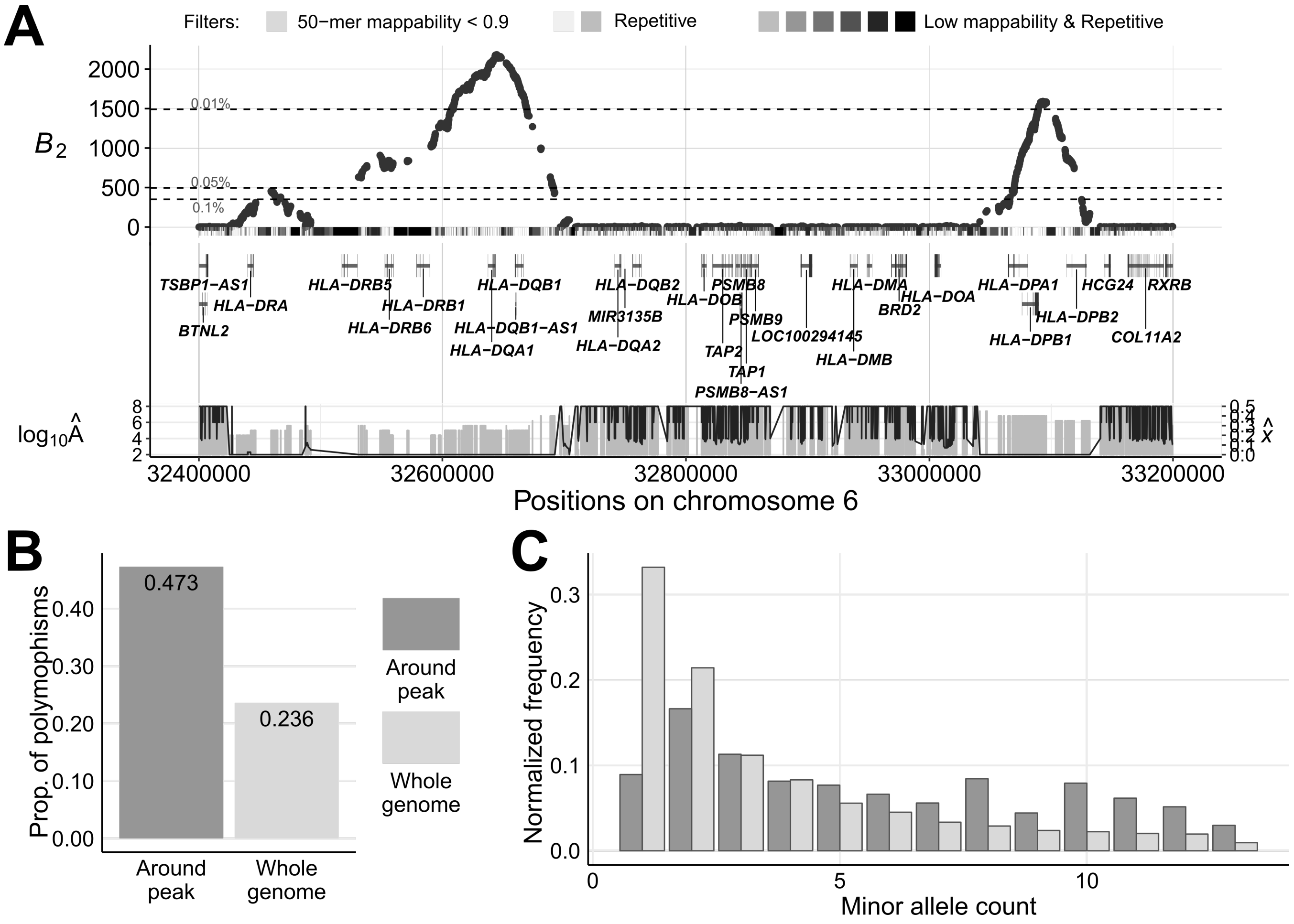

Figure 5: Evidence for balancing selection on *MHC-DQ* and *MHC-DP* genes in bonobos. Note that the plotted gene names are based on the annotations of human hg38 reference genome. (A) B_2 scores across the genomic region on chromosome 6 surrounding the *MHC-DQ* and *MHC-DP* genes. The gray bars directly under the B_2 scores represent the masked regions, as well as the features in these regions. The darker the shade, the greater number of types of repetitive sequences (*e.g.*, RepeatMasker mask, segmental duplication, simple repeats, or interrupted repeats) overlapping the region. Vertical gray bars below display the estimated equilibrium minor allele frequency $\widehat{x}$ for each maximum likelihood ratio B_2, and the black line traces the value for the respective inferred footprint size $\log_{10}(\widehat{A})$. (B) Proportion of informative sites that are polymorphic in the 800 kb region centered on the peak compared with the whole-genome average. (C) Minor allele frequency distribution in the 500 kb region centered on the peak compared with the whole-genome average.

Supplementary Note

1 Mixture models for multi-allelic balancing selection

1.1 Balancing selection with m alleles

For balancing selection with m classes of balanced alleles, denote each allelic class as $A_1, A_2, \ldots, A_m$, and their corresponding equilibrium frequencies as $x_1, x_2, \ldots, x_m$, with $\sum_{i=1}^m x_i = 1$. To avoid redundancy, we index the alleles by decreasing frequency, such that $x_1 \geq x_2 \geq \cdots \geq x_m$.

1.1.1 Probability distributions for allele frequencies of bi-allelic neutral sites linked to an m-allelic balanced locus

Consider n sampled lineages, and denote the counts and frequencies of each of the m balanced alleles, $A_1, A_2, \ldots, A_m$, at the selected locus as $\mathbf{n} = [n_1, n_2, \ldots, n_m]$ and $\mathbf{x} = [x_1, x_2, \ldots, x_m]$, respectively. For any neutral bi-allelic site linked to the selected locus, suppose k derived alleles are observed out of the n sampled lineages. Assume that alleles in each balanced class are either all in complete linkage (*i.e.*, on the same haplotype) with the derived alleles or all in complete linkage with the ancestral alleles on the neutral site. Consider a set of j distinct allele classes at the balanced locus, with allelic class indices $\{i_1, i_2, \ldots, i_j\}$ that make up a proper subset of the entire set of allelic class indices $\{1, 2, \ldots, m\}$. We consider these allelic classes as equally likely to be linked to the peripheral derived alleles at the neutral site, and therefore given that derived alleles are linked to exactly $j \in \{1, 2, \ldots, m-1\}$ classes, the probability that any particular set of j classes $\{A_{i_1}, A_{i_2}, \ldots, A_{i_j}\}$, with the set $\{i_1, i_2, \ldots, i_j\}$ denoting the indices of these classes, is this particular set is

$$P_1(j, m) = \frac{1}{\binom{m}{j}}.$$

Given that all k derived alleles are linked to exactly j, $j \in \{1, 2, \ldots, m-1\}$, balanced allelic classes, the ancestral alleles would be linked to the remaining allelic classes, which we merge into a single class that we denote by A_{anc}, which has equilibrium frequency $x_{\text{anc}} = 1 - \sum_{\ell=1}^{j} x_{i_l}$. Let $\mathbf{k} = [k_{i_1}, k_{i_2}, \ldots, k_{i_j}, n-k]$ denote that the sample configuration of the derived and ancestral alleles, indicating that there are $k_{i_\ell} > 0$ derived alleles linked to balanced allelic class A_{i_ℓ} for $\ell \in \{1, 2, \ldots, j\}$ and $n - k$ ancestral alleles linked to the remaining allelic classes A_{anc}. Moreover, let $\mathbf{x} = [x_{i_1}, x_{i_2}, \ldots, x_{i_j}, x_{\text{anc}}]$ denote the vector of equilibrium frequencies corresponding to these $j+1$ allelic classes. The probability of this sampled configuration $\mathbf{k}$ given frequencies $\mathbf{x}$ can be described by multinomial probability

$$\begin{aligned} P_2(\mathbf{k}, \mathbf{x}) &= \text{MN}(\mathbf{k}, n; \mathbf{x}) \\ &= \frac{n!}{(n-k)! \prod_{\ell=1}^{j} (k_{i_\ell})!} x_{\text{anc}}^{n-k} \prod_{\ell=1}^{j} x_{i_\ell}^{k_{i_\ell}}. \end{aligned}$$

Putting this all together, the probability of observing k derived alleles at the neutral site when it is in complete linkage with the locus under selection can be modeled as

$$\begin{aligned} h_{n,m,\mathbf{x}}(k) &= \sum_{j=1}^{m-1} \sum_{\{i_1, i_2, \ldots, i_j\} \subset \{1, 2, \ldots, m\}} P_1(j, m) \sum_{\substack{k_{i_1} + \cdots + k_{i_j} = k \\ k_{i_\ell} > 0, \ell = 1, 2, \ldots, j}} P_2(\mathbf{k}, \mathbf{x}), \\ &= \sum_{j=1}^{m-1} \frac{1}{\binom{m}{j}} \sum_{\{i_1, i_2, \ldots, i_j\} \subset \{1, 2, \ldots, m\}} \sum_{\substack{k_{i_1} + \cdots + k_{i_j} = k \\ k_{i_\ell} > 0, \ell = 1, 2, \ldots, j}} \frac{n!}{(n-k)! \prod_{\ell=1}^{j} (k_{i_\ell})!} x_{\text{anc}}^{n-k} \prod_{\ell=1}^{j} x_{i_\ell}^{k_{i_\ell}}. \end{aligned} \tag{1}$$

To tailor the distribution to different input types, normalize $h_{n,m,\mathbf{x}}(k)$ based on the possible values of k. Following the nomenclature adopted for the two-allele balancing selection (see *Model Description*), we use "0", "0,MAF", "1", "2", and "2,MAF" to annotate the distributions normalized based on derived allele frequencies only (*i.e.*, $k = 1, 2, \ldots, n-1$), minor allele frequencies only (*i.e.*, $k = 1, 2, \ldots, \lfloor \frac{n}{2} \rfloor$), polymorphic or substitution calls only (*i.e.*, $k \neq n$ or $k = n$), substitution calls with derived allele frequencies (*i.e.*, $k = 1, 2, \ldots, n$), and substitution calls with minor allele frequencies (*i.e.*, $k = 1, 2, \ldots, \lfloor \frac{n}{2} \rfloor$ for polymorphisms and $k = 0$ for substitutions), respectively. The probability mass functions for these normalized distributions can be computed as

$$\begin{aligned}
h^{(0)}_{n,m,\mathbf{x}}(k) &= \frac{h_{n,m,\mathbf{x}}(k)}{\sum_{k=1}^{n-1} h_{n,m,\mathbf{x}}(k)} & k \in \{1, 2, \ldots, n-1\} \\
h^{(0,\mathrm{MAF})}_{n,m,\mathbf{x}}(k) &= \frac{h_{n,m,\mathbf{x}}(k) + \mathbf{1}_{\{k \neq n/2\}} h_{n,m,\mathbf{x}}(n-k)}{\sum_{k=1}^{n-1} h_{n,m,\mathbf{x}}(k)} & k \in \{1, 2, \ldots, \lfloor n/2 \rfloor\} \\
h^{(1)}_{n,m,\mathbf{x}}(k) &= \frac{\mathbf{1}_{\{k=n\}} h_{n,m,\mathbf{x}}(n) + \mathbf{1}_{\{k \neq n\}} \sum_{i=1}^{n-1} h_{n,m,\mathbf{x}}(i)}{\sum_{k=1}^{n} h_{n,m,\mathbf{x}}(k)} & k \in \{1, 2, \ldots, n\} \\
h^{(2)}_{n,m,\mathbf{x}}(k) &= \frac{h_{n,m,\mathbf{x}}(k)}{\sum_{k=1}^{n} h_{n,m,\mathbf{x}}(k)} & k \in \{1, 2, \ldots, n\} \\
h^{(2,\mathrm{MAF})}_{n,m,\mathbf{x}}(k) &= \frac{\mathbf{1}_{k \neq 0} h_{n,m,\mathbf{x}}(k) + \mathbf{1}_{\{k \neq n/2\}} h_{n,m,\mathbf{x}}(n-k)}{\sum_{k=1}^{n} h_{n,m,\mathbf{x}}(k)} & k \in \{0, 2, \ldots, \lfloor n/2 \rfloor\}.
\end{aligned}$$

The probabilities under the null hypothesis of neutrality $g^{(\mathrm{X})}_n(k)$ ($X \in \{$"0", "0,MAF", "1", "2", "2,MAF"$\}$) are identical to their respective quantities provided in the main text.

1.1.2 Composite likelihood ratios based on the mixture models

For neutral site d recombination units away from the selected locus, use $\alpha_A(d) = e^{-Ad}$ to describe the chance of being linked to the balanced locus, the full model for observing k derived alleles on this site can be considered as the mixture of the influence of balancing selection (the $h^{(X)}_{n,m,\mathbf{x}}(k)$ in Equation 1 multiplied by $\alpha_A(d) = e^{-Ad}$) and neutrality (the $g^{(X)}_n(k)$ multiplied by $1 - \alpha_A(d)$).

$$f^{(X)}_{n,m,\mathbf{x},A}(k, d) = \alpha_A(d) \cdot h^{(X)}_{n,m,\mathbf{x}}(k) + [1 - \alpha_A(d)] g^{(X)}_n(k). \tag{2}$$

Let $\mathbf{d} = [d_1, d_2, ..., d_L]$ denote the vector of distances to the test site for L informative sites across the genomic region of interest. The composite likelihood for the null hypothesis of neutrality would be $\mathcal{L}^{(X)}_0(\mathbf{n}, \mathbf{k}) = \prod_{i=1}^{L} g^{(X)}_{n_i}(k_i)$, and the composite likelihood for the alternative hypothesis, $\mathcal{L}^{(X)}_a(m, \mathbf{x}, A; \mathbf{n}, \mathbf{k}, \mathbf{d})$, of being linked to balancing selection can be maximized at

$$(\widehat{\mathbf{x}}, \widehat{A}) = \underset{\mathbf{x}, A}{\arg\max} \prod_{i=1}^{L} f^{(X)}_{n_i,m,\mathbf{x},A}(k_i, d_i).$$

The general B statistic can thus be formulated as

$$B_X = 2\Big[\ln \mathcal{L}^{(X)}_a(m, \widehat{\mathbf{x}}, \widehat{A}; \mathbf{n}, \mathbf{k}, \mathbf{d}) - \ln \mathcal{L}^{(X)}_0(\mathbf{n}, \mathbf{k})\Big]. \tag{3}$$

1.2 Probability distributions for balancing selection with $m = 2$ alleles

When $m = 2$, equation 1 can be reduced to

$$
\begin{aligned}
h_{n,2,\mathbf{x}}(k) &= \sum_{j=1}^{2-1} \frac{1}{\binom{m}{j}} \sum_{\{A_{i_1}, A_{i_2}, \ldots, A_{i_j}\} \subset \{A_1, A_2\}} \frac{n!}{k!(n-k)!} \left(\sum_{\ell=1}^{j} x_{i_\ell} \right)^k \left(1 - \sum_{\ell=1}^{j} x_{i_\ell} \right)^{n-k} \\
&= \frac{1}{2} \left[\binom{n}{k} x_1^k (1 - x_1)^{n-k} + \binom{n}{k} x_2^k (1 - x_2)^{n-k} \right] \\
&= \frac{1}{2} \Big[\text{Bin}(k, n; x) + \text{Bin}(k, n; 1 - x) \Big]
\end{aligned}
$$

This formulation is consistent with the ones described in *Model Description.* Because the probability mass function for neutrality stays the same regardless of selection, the formulation of $g_n(k)$ would not change. Therefore, the resulting mixture models are consistent with those adopted in the main manuscript.

2 Matching the number of sites in a genomic region with fixed length

In general, we considered two types of sliding windows in the simulation study when evaluating each statistic's robustness to large window sizes. The first type of sliding window was a region with a fixed number of informative sites, whereas the second was a region with a fixed number of nucleotides (nt). Because `BALLET` (*i.e.*, T_1 and T_2) and `BetaScan` (*i.e.*, β and $\beta^{(2)}$) adopt different types of sliding windows, we applied B statistics in both ways separately so as to better compare their performances with current methods. To approximately match the lengths of genomic regions analyzed by T statistics and the summary statistics, we calculated the expected number of informative sites I_n for a sample of n lineages, based on the parameters of the simulated neutral demographic histories.

2.1 Constant-size demographic history relating three species with a uniform mutation rate

For the majority of our simulations, we adopted a constant-size demographic history inspired by great apes that relates a population P to two outgroup populations O1 and O2 (depicted in Figure S1). Under this scenario, O1 and O2 diverged from P $t_1 = 2.5 \times 10^5$ and $t_2 = 4 \times 10^5$ generations ago, respectively. For these calculations, we assumed the population size is a constant $N = 10^4$ diploid individuals across the entire demographic model. In our analyses, we randomly sampled $n = 50$ lineages from population P and used one lineage in either population O1 or O2 to call substitutions and to polarize allele frequencies in the sample from P. Let C_1 and C_2 denote the inter-species coalescence time between P and O1 or O2, respectively, and let H_n and L_n be the expected tree height and tree length for a sample of n lineages taken from P, respectively. Under neutrality, these quantities can be computed as (**??**)

$$\begin{aligned} C_1 &= \frac{t_1}{2N} + 1 \\ &= \frac{2.5 \times 10^5}{2 \times 10^4} + 1 = 13.5 \\ C_2 &= \frac{t_2}{2N} + 1 \\ &= \frac{4 \times 10^5}{2 \times 10^4} + 1 = 21 \end{aligned}$$

and

$$\begin{aligned} H_{50} &= 2\left(1 - \frac{1}{n}\right) \\ &= 2\left(1 - \frac{1}{50}\right) = 1.96 \\ L_{50} &= 2\sum_{k=1}^{n-1} \frac{1}{k} \\ &= 2\sum_{k=1}^{50-1} \frac{1}{k} \approx 8.96. \end{aligned}$$

Therefore, the expected numbers of informative sites for using either O1 or O2 as the outgroup are respectively

$$\begin{aligned} I_{50}(\text{P-O1}) &= 2N\mu(2C_1 - H_n + L_n) \\ &= 2(10^4)(2.5 \times 10^{-8})(2C_1 - H_{50} + L_{50}) \approx 0.016999 \text{ per nucleotide} \\ I_{50}(\text{P-O2}) &= 2N\mu(2C_2 - H_n + L_n) \\ &= 2(10^4)(2.5 \times 10^{-8})(2C_2 - H_{50} + L_{50}) \approx 0.024499 \text{ per nucleotide,} \end{aligned}$$

assuming a per-site per-generation mutation rate of $\mu = 2.5 \times 10^{-8}$.

That is, when using O2 as the outgroup, a window with a radius of 10 informative sites (21 informative sites in total) on average approximately spans a genomic region of length 857 nt, and when using O1 as the outgroup this length is approximately 1235 nt. With O2 as the outgroup, window sizes of 1, 2, 3, 5, 10, 15, 20, or 25 kb should on average approximately correspond to windows of a fixed radius of 12, 24, 36, 60, 122, 183, 245, and 367 informative sites, respectively.

2.2 Demographic history relating two species with realistic parameters

For simulations adopting the realistic demographic histories of the human CEU population or of bonobos, mutation rates and recombination rates are altered to correspond to the parameters used to infer the demographic histories. The theoretical footprint sizes as well as the density of informative sites consequently vary from sequences simulated under constant population size models.

Instead of $\mu = 2.5 \times 10^{-8}$ mutation and $r = 10^{-8}$ recombination events per site per generation, the CEU demographic history adopts a per site per generation mutation rate of $\mu = 1.25 \times 10^{-8}$ and a recombination rate drawn uniformly at random between $r = 5 \times 10^{-9}$ and $r = 1.5 \times 10^{-8}$ per site per generation. Given the range of recombination rates, the theoretical size of footprints would vary between five kb to approximately 1.67 kb. With the halved mutation rate, on the other hand, I_{50}(P-O1) would fall around 0.0085 per nucleotide, which translates to genomic regions covering on average 14.2 to 42.5 informative sites. To ensure that T statistics consider enough data to reduce noise, we allow it to employ 10 informative sites at either side of the test informative site (21 informative sites in total covered by each window), and the corresponding mean physical length is approximately 2.5 kb.

For sequences simulated under the bonobo demographic history, the recombination rate was set to $r = 2.84 \times 10^{-9}$ per site per generation, under which the theoretical footprint size of long-term balancing selection is expected to be approximately 8.8 kb. Considering most tested statistics showed optimal power with one kb windows when the theoretical footprints was 2.5 kb, we adopted a window size of 3.6 kb here each summary statistic. With the I_{50}(P-O1) obtained under the mutation rate of $\mu = 2.5 \times 10^{-8}$, a 3.6 kb region should on average cover approximately 60 informative sites. We therefore set the window size for T statistics as 29 sites on either side of the test informative site, such that each test considers a genomic region covering 59 informative sites.

2.3 Demographic history relating two species with species-specific mutation rates

When simulating a demographic history with species-specific mutation rates, sequences of the target population mutates at a rate of $\mu_{\text{H}} = 1.2 \times 10^{-8}$ per generation per site after splitting with the outgroup population, which has a constant per generation mutation rate of $\mu = 2.5 \times 10^{-8}$ per site, which is also the same mutation rate as the ancestral population. Moreover, assume that the target population and the group population diverge at the same number of generations as populations P and O1 (depicted in Figure S1).

Recall that we found the inter-species coalescence time between P and O1 to be $C_1 = 13.5$ coalescent units, and that we estimate the tree height and tree length for 50 sampled lineages in the target population to be $H_{50} = 1.96$ and $L_{50} \approx 8.96$, respectively.

It follows that the expected number of informative sites using O1 as the outgroup is

$$\begin{aligned} I_{50}(\text{P-O1}) &= 2N\mu_{\text{H}}(C_1 - 1 - H_{50} + L_{50}) + 2N\mu(C_1 + 1) \\ &= 2(10^4)(1.2 \times 10^{-8})(13.5 - 1 - 1.96 + 8.96) + 2(10^4)(2.5 \times 10^{-8})(13.5 + 1) \\ &= 0.01193 \text{ per nucleotide.} \end{aligned}$$

Therefore, a one kb region would cover 11.93 informative sites, and a 2.5 kb region would cover approximately 29.825 informative sites. To again ensure T statistics have enough data for each test, we adopted a window size of 15 informative sites on either side of the test informative site, and applied the summary statistics with windows of length 2.5 kb.

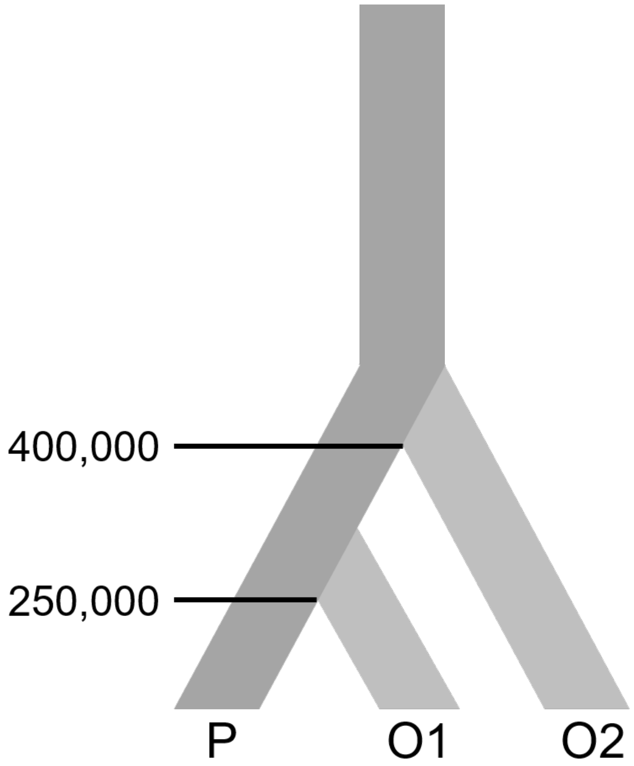

Figure S1: Schematic for the three-species demographic history considered in our simulation study. Divergence times of the closer (O1) and more distant (O2) outgroup are labeled in terms of the numbers of generations, and the population size across the entire phylogeny is $N = 10^4$ diploid individuals. In scenarios with balancing selection, the mutation under selection is only introduced in the lineage ancestral to population P, indicated in darker gray shading.

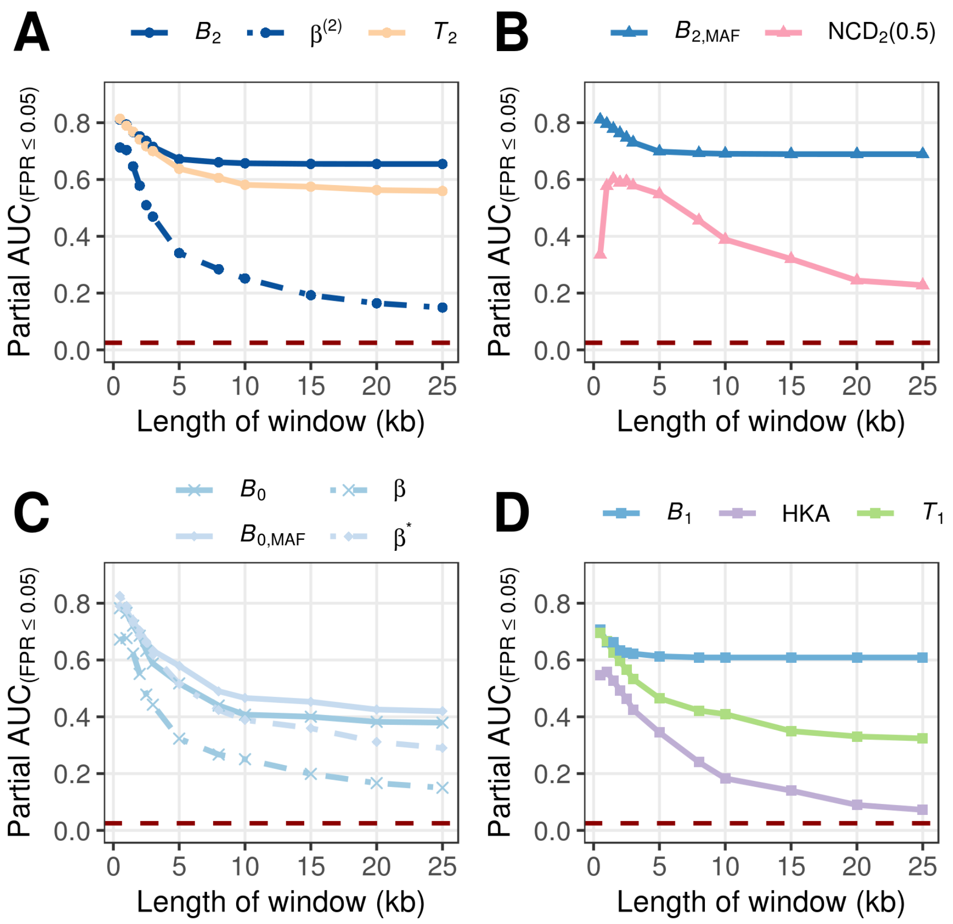

Figure S2: Partial area under the curve (AUC) conditioned on false positive rates (FPRs) less than or equal to 5% (defined such that the maximal value is one) as a function of window size measured in kilobases (kb) for (A) B_2, T_2, and $\beta^{(2)}$, all of which consider both substitution calls and polarized allele frequencies; (B) $B_{2,\text{MAF}}$ and NCD(0.5) statistics, both using substitution calls and minor allele frequency; (C) B_0, β, $B_{0,\text{MAF}}$, and β^*, which only consider allele frequencies of polymorphic sites, with the former two (marked with crosses) using derived allele frequencies, and the latter two (marked with dots) using minor allele frequencies; (D) B_1, T_1, and HKA, all using polymorphism and substitution calls only.

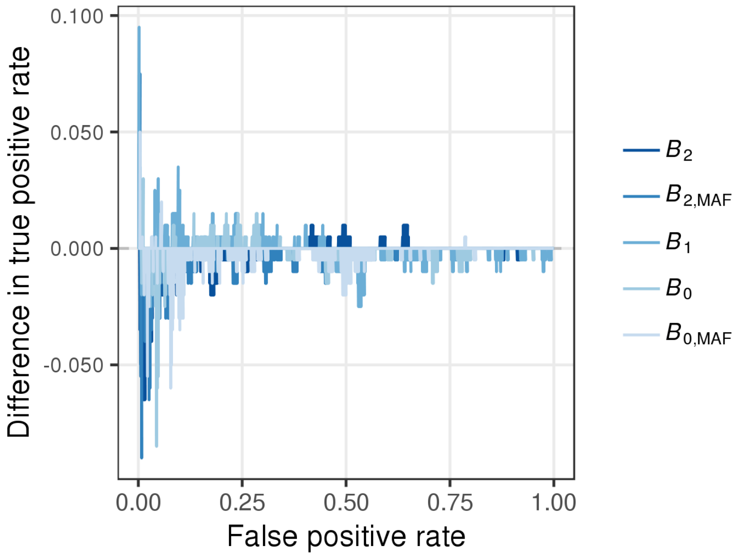

Figure S3: Effect of truncating the calculation of B statistics, illustrated by the difference in true positive rate for each B statistic as a function of false positive rate, where the difference in true positive rate for a B statistic is computed as the true positive rate when using all available informative sites on a simulated sequence minus the true positive rate when using only sites in which $\alpha_A(d) \geq 10^{-8}$.

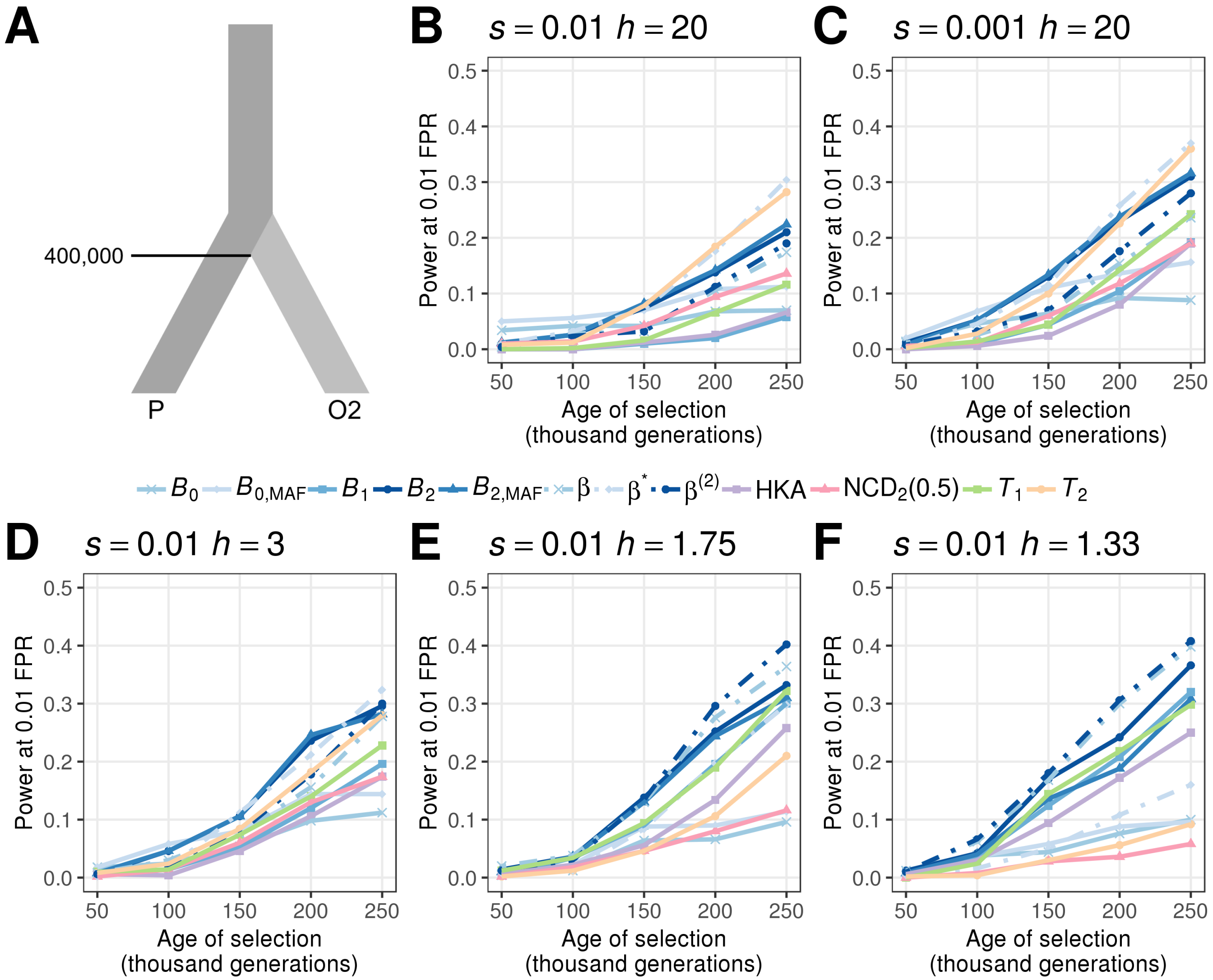

Figure S4: Ability to detect balancing selection for different heterozygote advantage scenarios occurring within 250,000 generations ago. (A) Demographic model relating the ingroup (P) and outgroup (O2) populations, with one sample from O2 used as the outgroup sequence. (B-F) Powers at a 1% false positive rate (FPR) for each statistic as a function of age of the allele undergoing balancing selection for different selection (s) and dominance (h) coefficients. The scenarios considered are (B) $s = 0.01$ with $h = 20$, (C) $s = 0.001$ with $h = 20$, (D) $s = 0.01$ with $h = 3$, (E) $s = 0.01$ with $h = 1.75$, and (F) $s = 0.01$ with $h = 1.33$. Note that the equilibrium frequencies for panels D, E, and F are 0.4, 0.3, and 0.2, respectively.

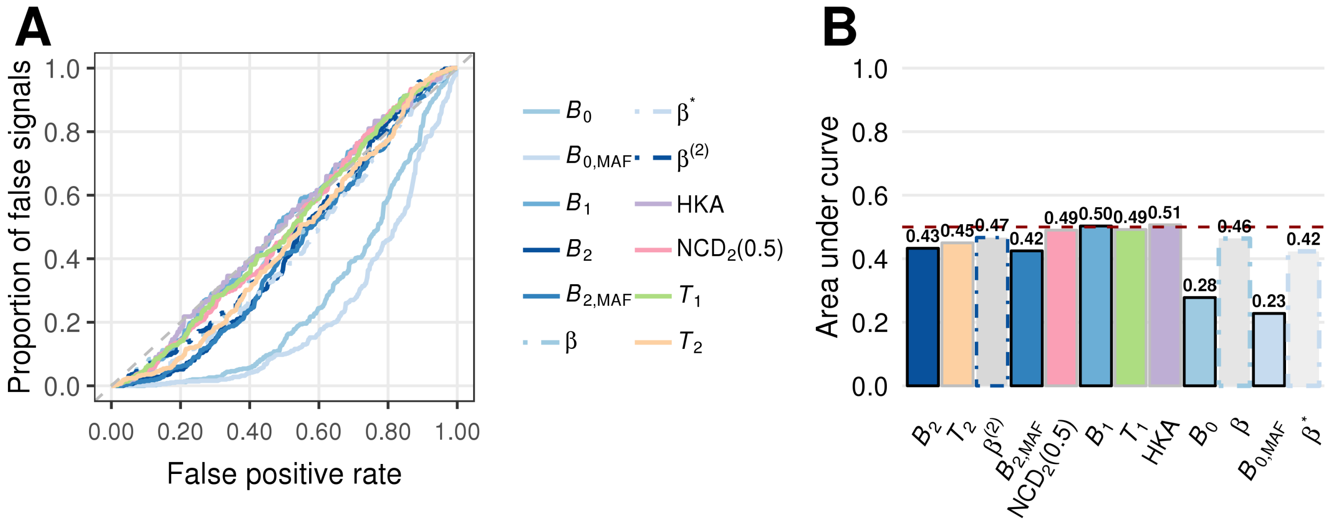

Figure S5: Performance of all statistics on neutral sequences with an uneven recombination map, with the recombination rate fluctuating from 10-fold higher to 10-fold lower than the uniform recombination rate r between neighboring one kb windows. (A) Proportions of false signal as a function of false positive rate. (B) Area under the curve for all statistics. Bars for all B statistics are bordered by black lines, and all β statistics are filled in gray. The false positive rate was based on neutral simulations with the original recombination rate $r = 10^{-8}$ per site per generation, and the proportion of false signals was based on neutral simulations with recombination rate fluctuating between $r/10$ and $10r$. We define a false signal as a simulated replicate that has a higher score than the score associated with a given false positive rate cutoff.

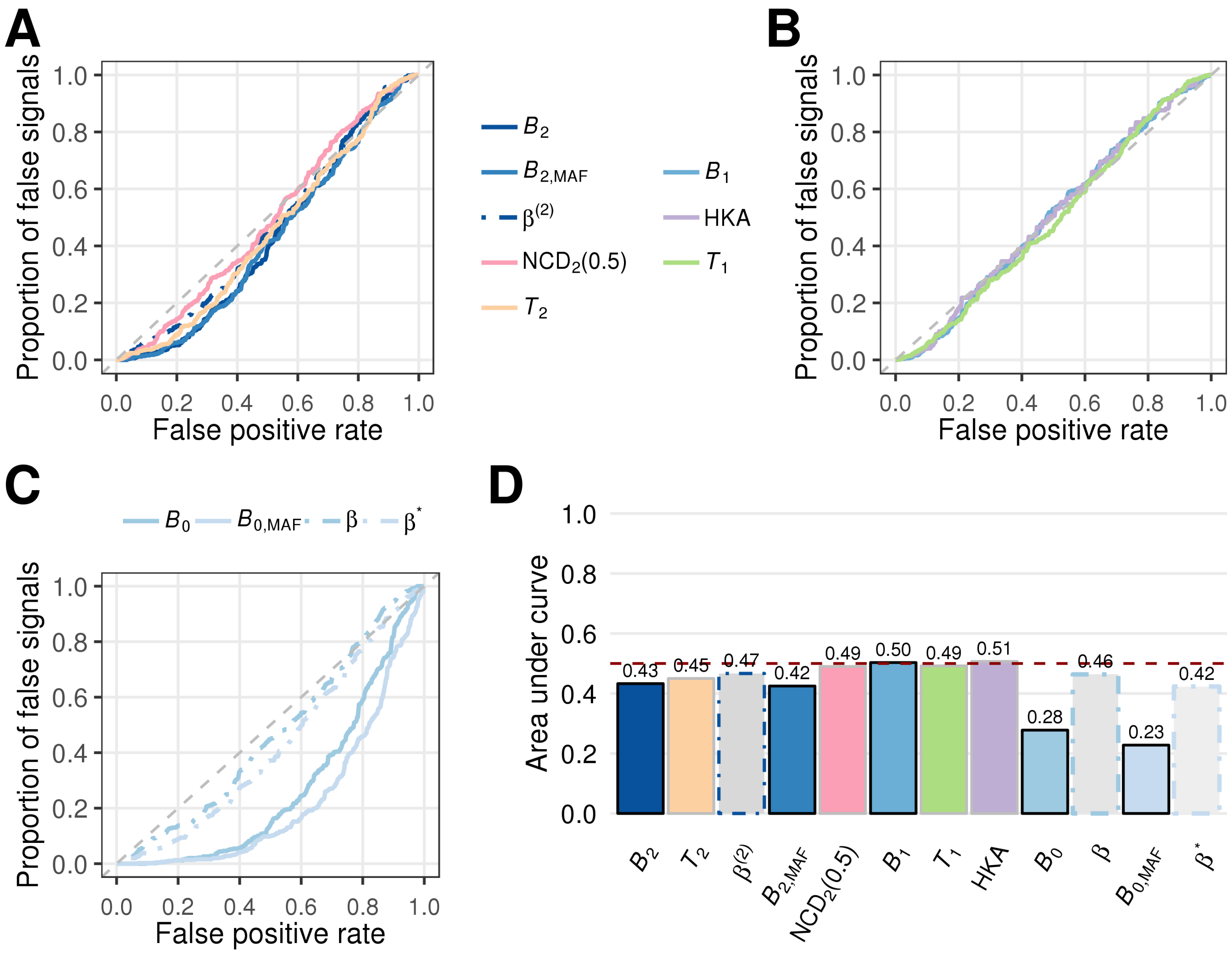

Figure S6: Split view for performance of three groups of analogous statistics on neutral sequences with an uneven recombination map, with the recombination rate fluctuating from 10-fold higher to 10-fold lower than the uniform recombination rate r between neighboring one kb windows. (A-C) Proportions of false signals as a function of false positive rate for (A) statistics that use both substitutions and allele frequencies, namely B_2, T_2, $B_{2,\text{MAF}}$, $\beta^{(2)}$, and NCD, (B) statistics that only use substitution and polymorphism calls, *i.e.*, B_1, T_1, and HKA, and (C) statistics that only use allele frequencies, which are B_0, β, $B_{0,\text{MAF}}$, and β^*. (D) Area under the curve for all statistics. Bars for all B statistics are bordered by black lines, and all β statistics are filled in gray. In all panels, the false positive rate was based on neutral simulations with the original recombination rate $r = 10^{-8}$ per site per generation, and the proportion of false signals was based on neutral simulations with recombination rate fluctuating between $r/10$ and $10r$. We define a false signal as a simulated replicate that has a higher score than the score associated with a given false positive rate cutoff.

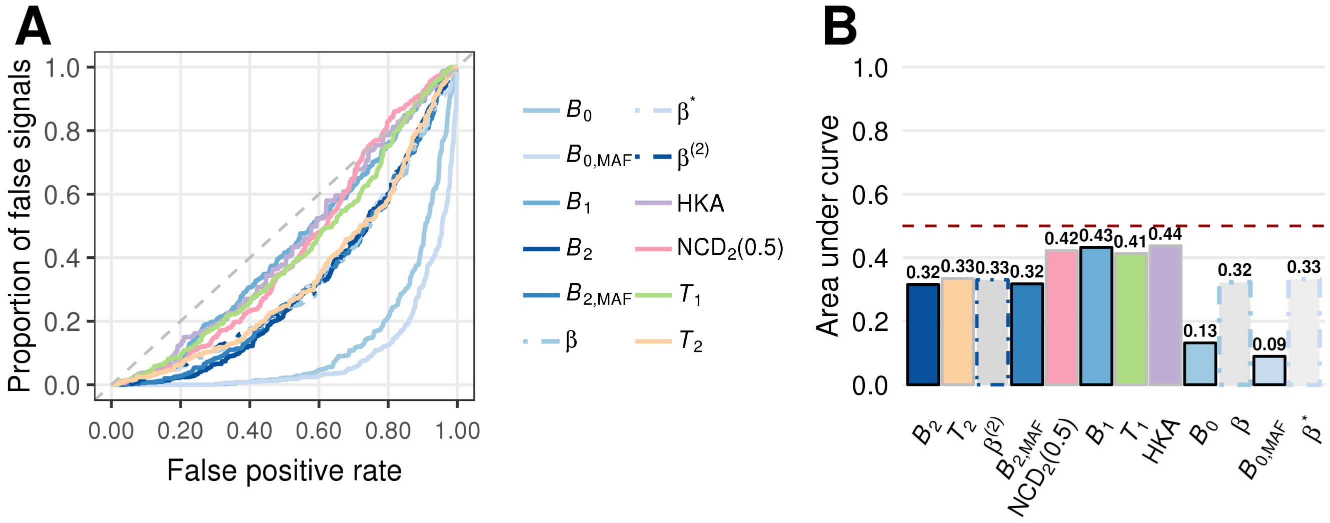

Figure S7: Robustness of each statistic to neutral sequences evolving under an uneven recombination map, with the recombination rate fluctuating from 100-fold higher to 100-fold lower than the uniform recombination rate r between neighboring one kb windows. (A) Proportions of false signal as a function of false positive rate. (B) Area under the curve for all statistics. Bars for all B statistics are bordered by black lines, and all β statistics are filled in gray. The false positive rate was based on neutral simulations with the original recombination rate $r = 10^{-8}$ per site per generation, and the proportion of false signals was based on neutral simulations with recombination rate fluctuating between $r/100$ and $100r$. We define a false signal as a simulated replicate that has a higher score than the score associated with a given false positive rate cutoff.

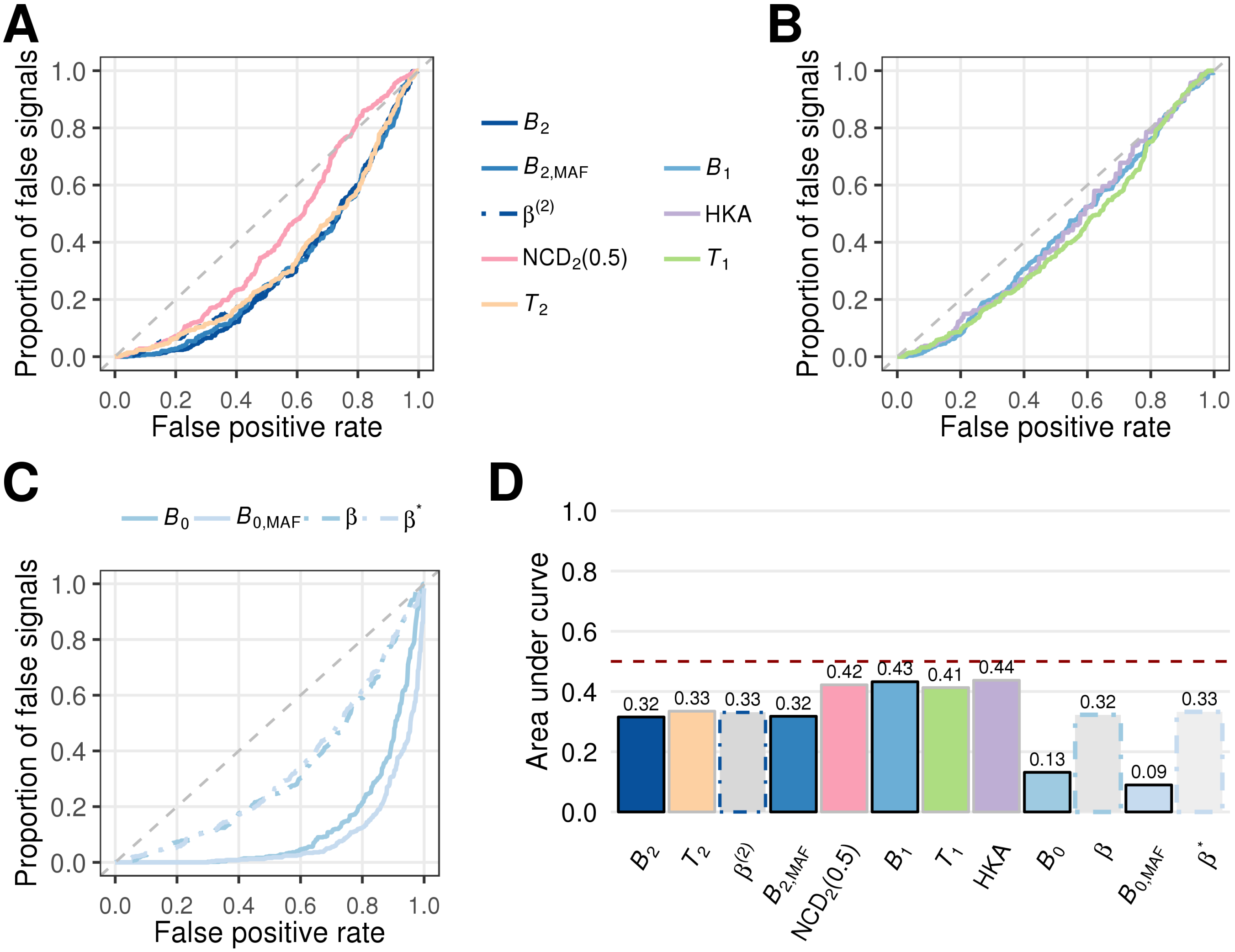

Figure S8: Split view for performance of three groups of analogous statistics on neutral sequences with an uneven recombination map, with the recombination rate fluctuating from 100-fold higher to 100-fold lower than the uniform recombination rate r between neighboring one kb windows. (A-C) Proportions of false signals as a function of false positive rate for (A) statistics that use both substitutions and allele frequencies, namely B_2, T_2, $B_{2,\mathrm{MAF}}$, $\beta^{(2)}$, and NCD, (B) statistics that only use substitution and polymorphism calls, *i.e.*, B_1, T_1, and HKA, and (C) statistics that only use allele frequencies, which are B_0, β, $B_{0,\mathrm{MAF}}$, and β^*. (D) Area under the curve for all statistics. Bars for all B statistics are bordered by black lines, and all β statistics are filled in gray. In all panels, the false positive rate was based on neutral simulations with the original recombination rate $r = 10^{-8}$ per site per generation, and the proportion of false signals was based on neutral simulations with recombination rate fluctuating between $r/100$ and $100r$. We define a false signal as a simulated replicate that has a higher score than the score associated with a given false positive rate cutoff.

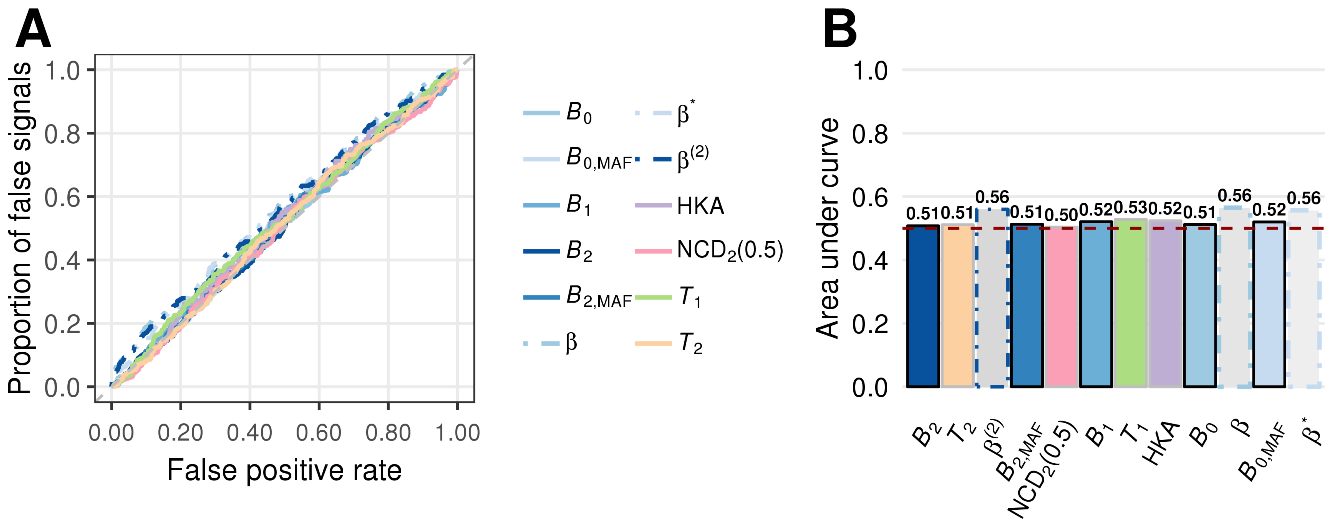

Figure S9: Robustness of each statistic evaluated when the mutation rate is elevated five-fold in a central 10 kb genomic region. (A) Proportions of false signals as a function of false positive rate (FPR). (B) Area under the curve for all statistics. Bars for all B statistics are bordered by black lines, and all β statistics are filled in gray. The false positive rate was based on neutral simulations with the original mutation rate $\mu = 2.5 \times 10^{-8}$ per site per generation, and the proportion of false signals was based on neutral simulations with the elevated mutation rate of 5μ in a central 10 kb region. We define a false signal as a simulated replicate that has a higher score than the score associated with a given false positive rate cutoff.

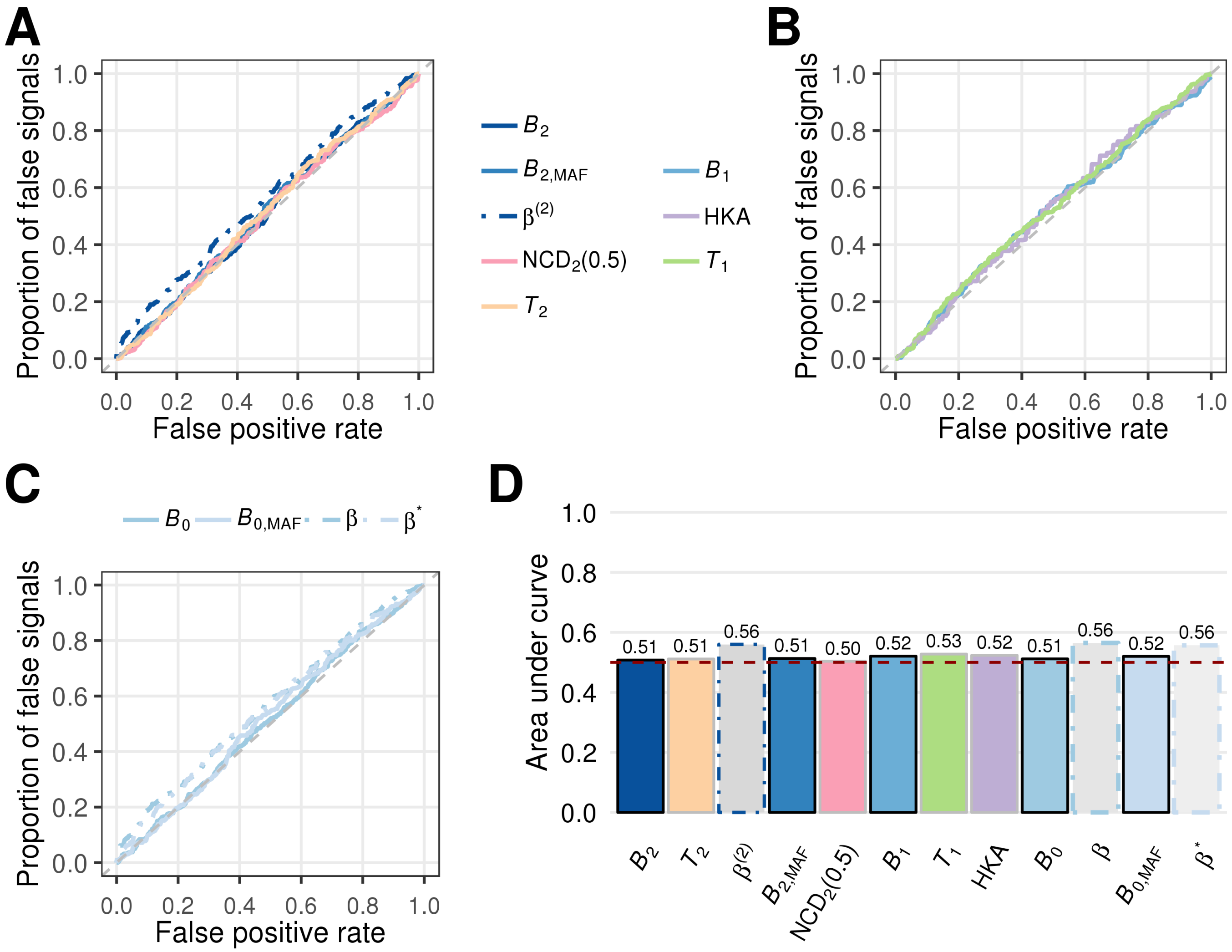

Figure S10: Split view for performance of three groups of analogous statistics on neutral sequences with the mutation rate on the center 10 kb region elevated five-fold. (A-C) Proportions of false signals as a function of false positive rate for (A) statistics that use both substitutions and allele frequencies, namely B_2, T_2, $B_{2,\mathrm{MAF}}$, $\beta^{(2)}$, and NCD, (B) statistics that only use substitution and polymorphism calls, *i.e.*, B_1, T_1, and HKA, and (C) statistics that only use allele frequencies, which are B_0, β, $B_{0,\mathrm{MAF}}$, and β^*. (D) Area under the curve for all statistics. Bars for all B statistics are bordered by black lines, and all β statistics are filled in gray. In all panels, the false positive rate was based on neutral simulations with the original mutation rate $\mu = 2.5 \times 10^{-8}$ per site per generation, and the proportion of false signals was based on neutral simulations with the elevated mutation rate of 5μ in a central 10 kb region. We define a false signal as a simulated replicate that has a higher score than the score associated with a given false positive rate cutoff.

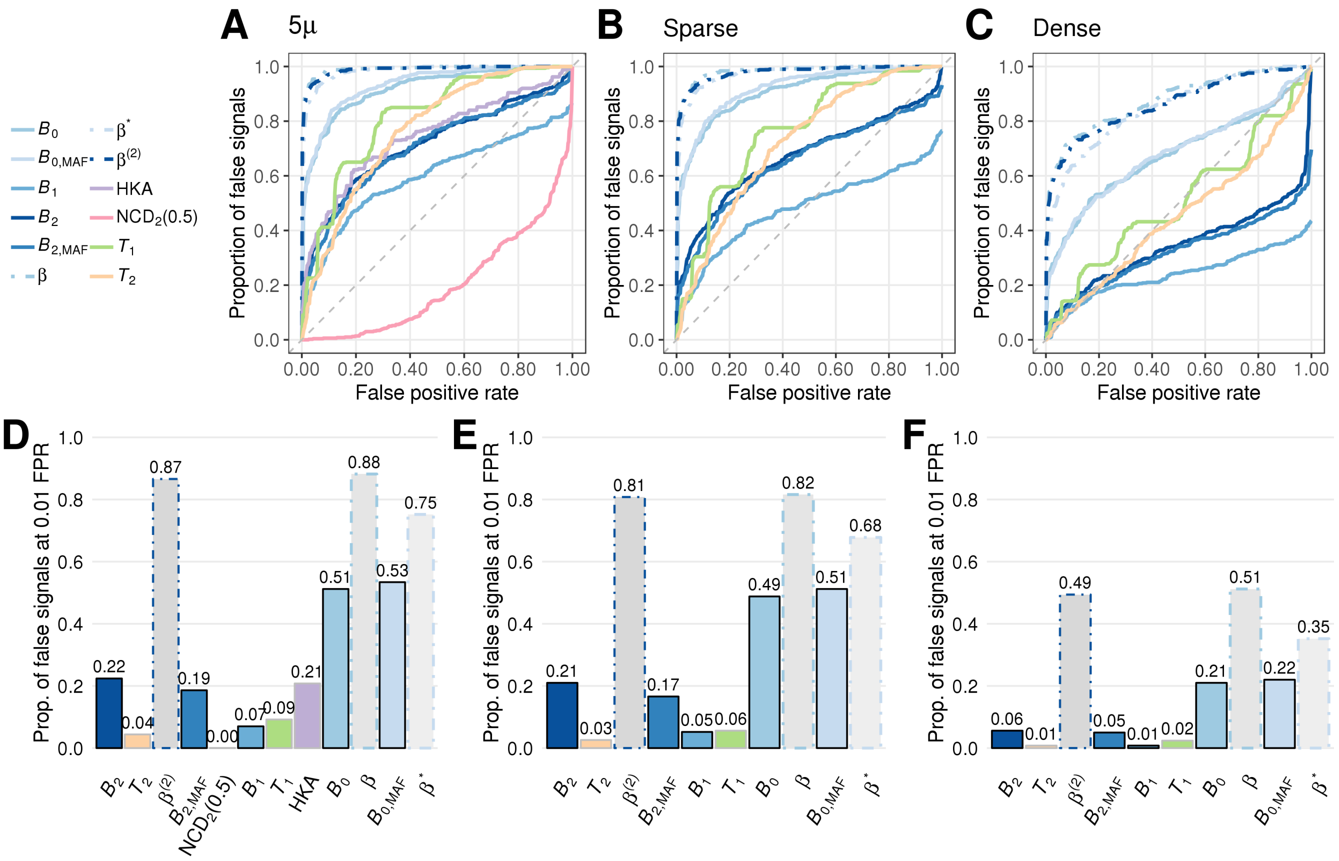

Figure S11: Propensity of each statistic to misidentify sequences with an elevated mutation rate of 5μ as undergoing balancing selection. (A-C) Proportions of false signals as a function of false positive rate (FPR) when (A) all test sites were considered to compute proportions of false signals and when (B, C) the number of test sites were down-sampled to match the number of sites expected on sequences with the original mutation rate μ. In panel B, five informative sites flanking either side of a test site considered were skipped ("sparse" down-sampling), whereas in panel C only the first 1200 informative sites were considered for calculating T, B_2, and B_1 statistics, and the first 240 polymorphic sites were considered for B_0 and β statistics ("dense" down-sampling). (D-F) Proportions of false signals at a 1% FPR for each statistic for (D) all test sites, (E) sparse down-sampling, and (F) dense down-sampling. Bars for all B statistics are bordered by black lines, and all β statistics are bordered by gray lines in panels D-F. HKA and $NCD_2(0.5)$ are applied with a fixed step size, and are therefore not considered when down-sampling in panels B, C, E, and F. In all panels, the false positive rate was based on neutral simulations with the original mutation rate $\mu = 2.5 \times 10^{-8}$ per site per generation, and the proportion of false signals was based on neutral simulations with the elevated mutation rate of 5μ. We define a false signal as a simulated replicate that has a higher score than the score associated with a given false positive rate cutoff.

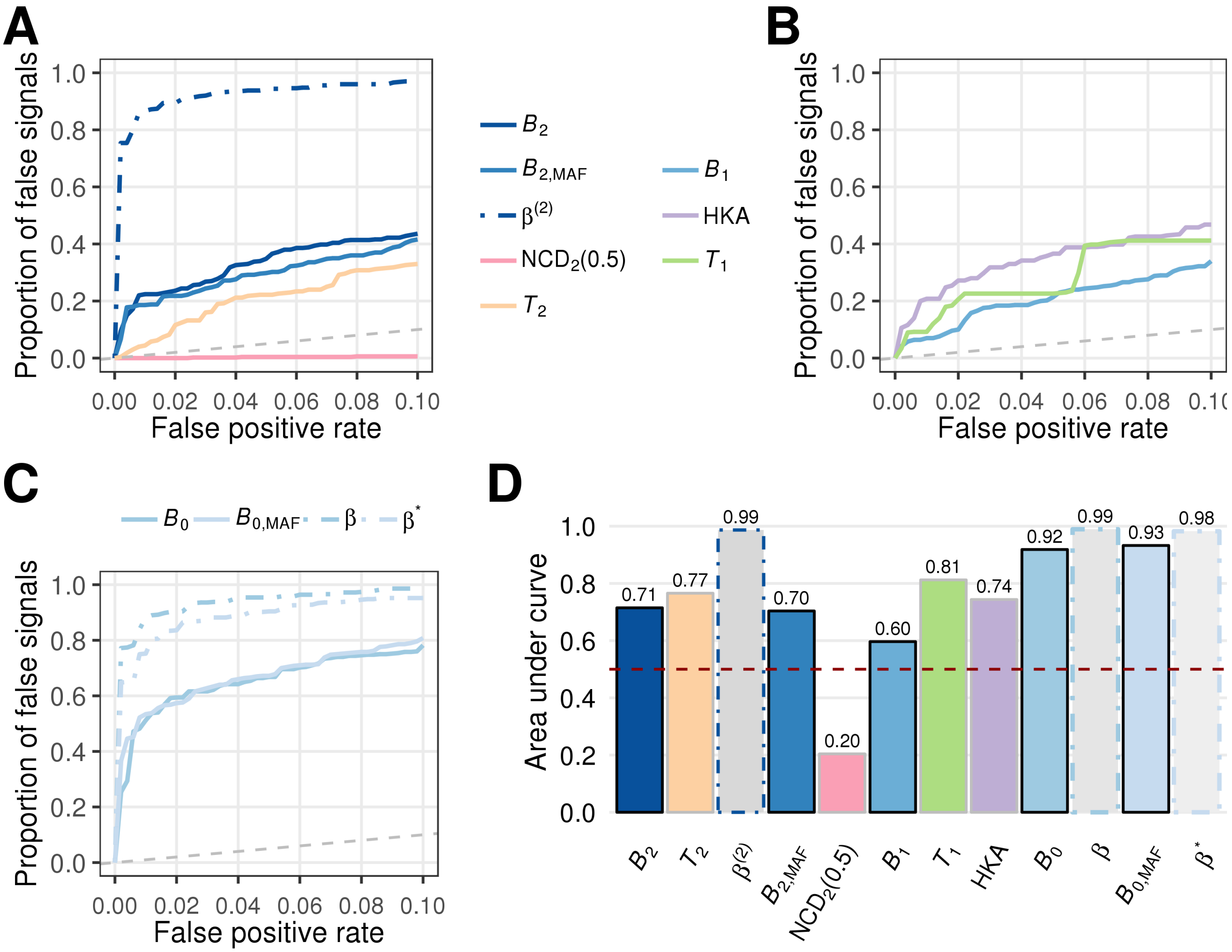

Figure S12: Split view for the propensity to misidentify neutral sequences with an elevated mutation rate of 5μ as undergoing balancing selection of three groups of analogous statistics. (A-C) Proportions of false signals as a function of false positive rate for (A) statistics that use both substitutions and allele frequencies, namely B_2, T_2, $B_{2,\text{MAF}}$, $\beta^{(2)}$, and NCD, (B) statistics that only use substitution and polymorphism calls, *i.e.*, B_1, T_1, and HKA, and (C) statistics that only use allele frequencies, which are B_0, β, $B_{0,\text{MAF}}$, and β^*. (D) Area under the curve for all statistics. Bars for all B statistics are bordered by black lines, and all β statistics are filled in gray. In all panels, the false positive rate was based on neutral simulations with the original mutation rate $\mu = 2.5 \times 10^{-8}$ per site per generation, and the proportion of false signals was based on neutral simulations with the elevated mutation rate of 5μ. We define a false signal as a simulated replicate that has a higher score than the score associated with a given false positive rate cutoff.

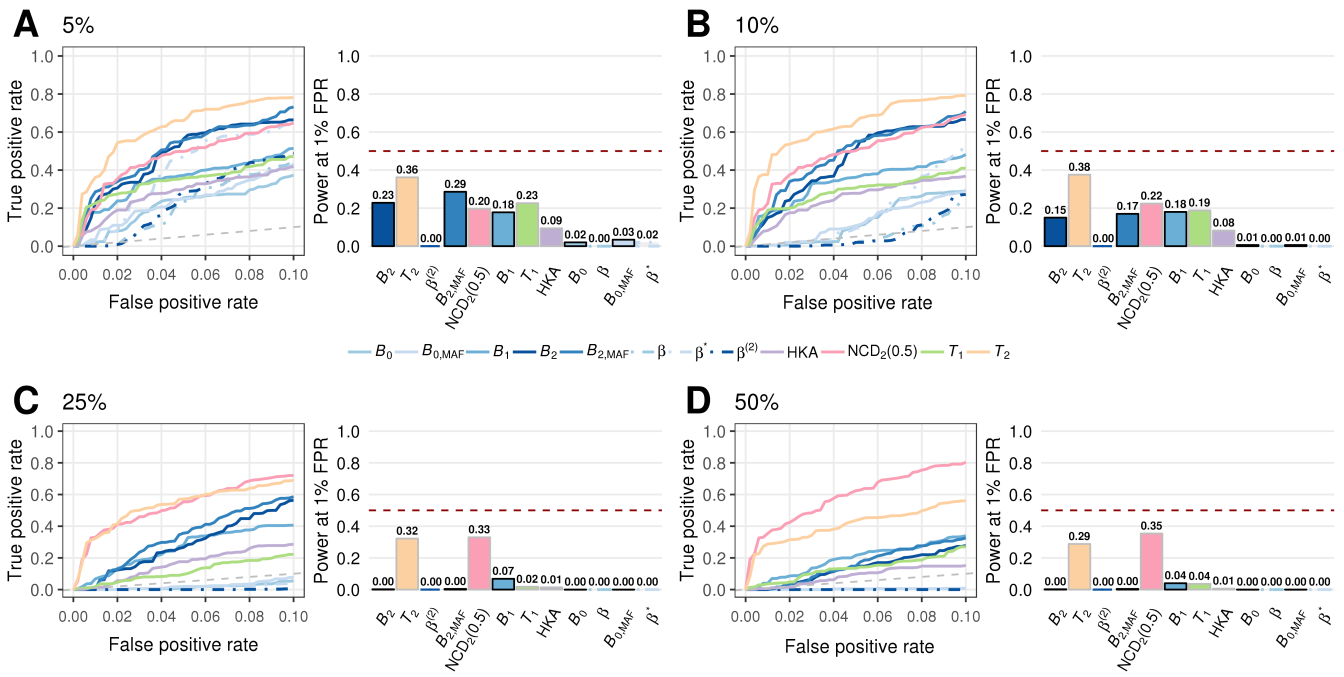

Figure S13: Receiver operating characteristic curves (left panel) and power at a 1% false positive rate (FPR; right panel) for each statistic to detect balancing selection when (A) 5%, (B) 10% (C) 25%, or (D) 50% of the sequences in the pool of neutral sequences have a mutation rate of 5μ, which is five times higher than that of the other sequences.

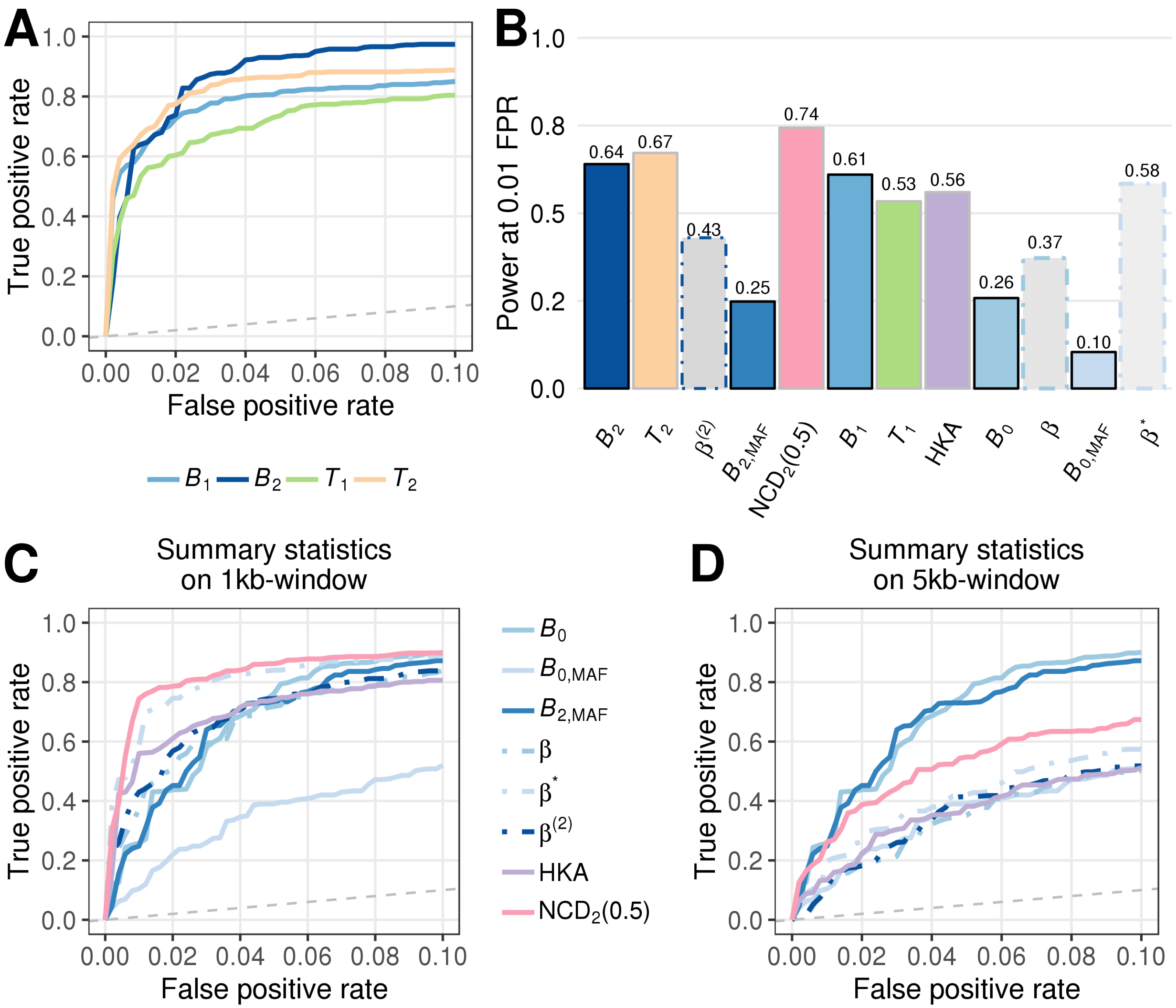

Figure S14: Power of each statistic to detect balancing selection when the mutation rate is elevated five-fold. (A) Receiver characteristic operation (ROC) curves for the model-based T statistics and their equivalent B statistics. (B) Powers at 1% false positive rate (FPR) for all statistics evaluated. Bars for all B statistics are bordered by black lines, and all β statistics are filled in gray. (C,D) ROC curves for B_1, B_0, and $B_{0,\mathrm{MAF}}$ using the whole sequence, and all summary statistics when they adopt a window size of (C) one kb or (D) five kb.

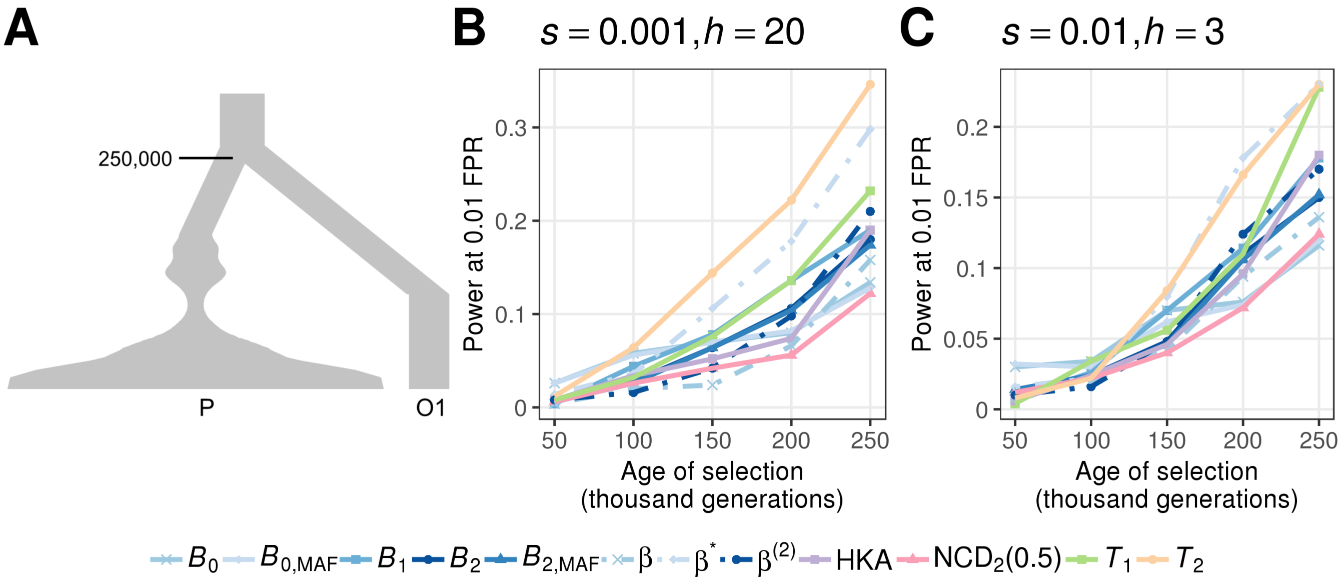

Figure S15: Power of all statistics as a function of the age of selection under the demographic model of CEU. (A) Schematic for the simulated demographic history. The outgroup O1 diverged from population P 2.5×10^5 generations ago. (B, C) Power at a 1% false positive rate (FPR) of each statistics for detecting balancing selection with selection parameters (B) $s = 0.001$ with $h = 20$ and (C) $s = 0.01$ with $h = 3$.

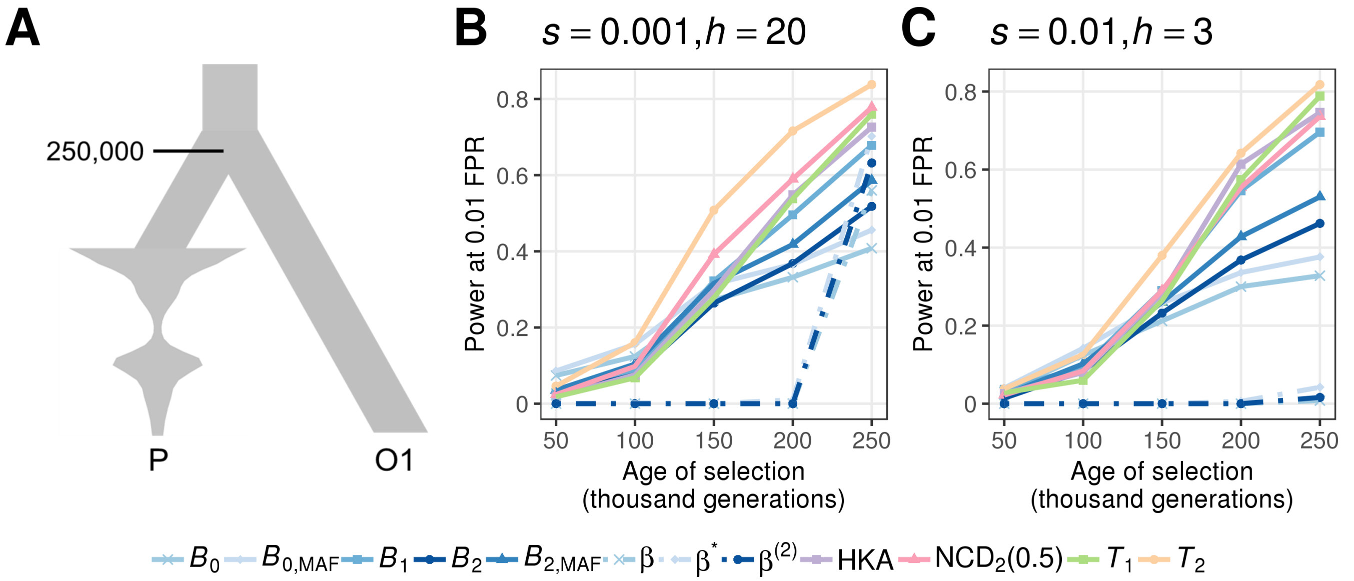

Figure S16: Power of all statistics as a function of the age of selection under the demographic model of bonobos. (A) Schematic for the simulated demographic history. The outgroup O1 diverged from population P 2.5×10^5 generations ago. (B, C) Power at a 1% false positive rate (FPR) of each statistics for detecting balancing selection with selection parameters (B) $s = 0.001$ with $h = 20$ and (C) $s = 0.01$ with $h = 3$.

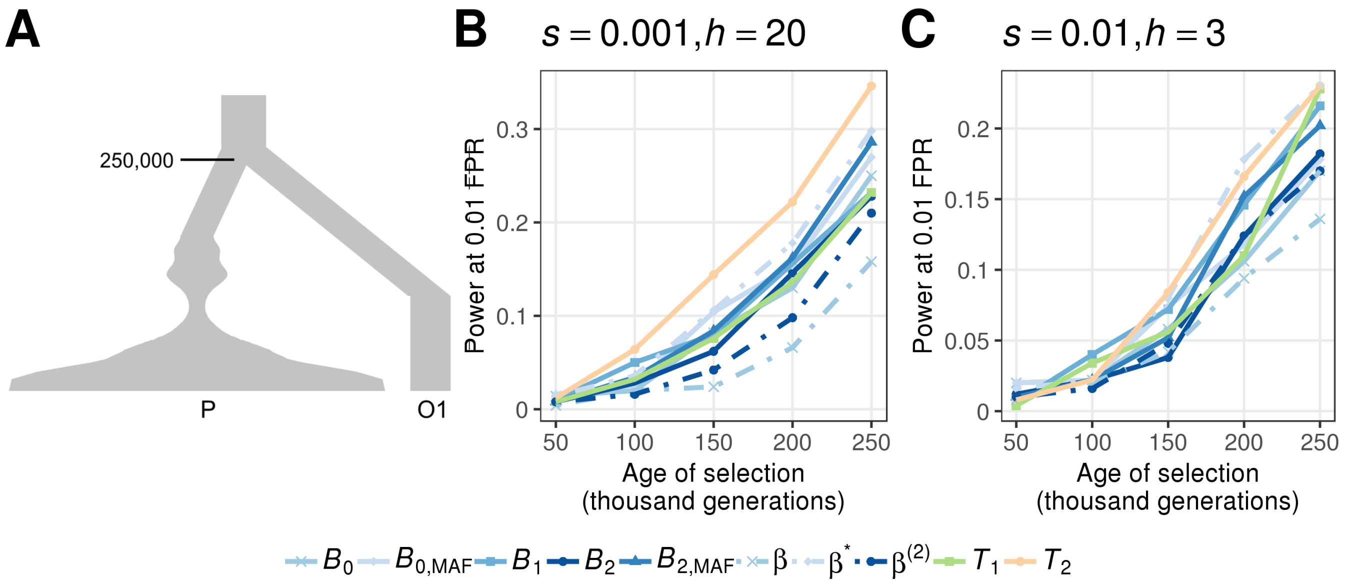

Figure S17: Power of B statistics when provided with identical window sizes as T statistics or β statistics, in comparison with other statistics for detecting balancing selection with varying age and parameters under the demographic history of CEU. (A) Schematic of the demographic model of CEU. (B, C) Power at a 1% false positive rate (FPR) of each statistics for detecting balancing selection with selection parameters (B) $s = 0.001$ with $h = 20$ and (C) $s = 0.01$ with $h = 3$.

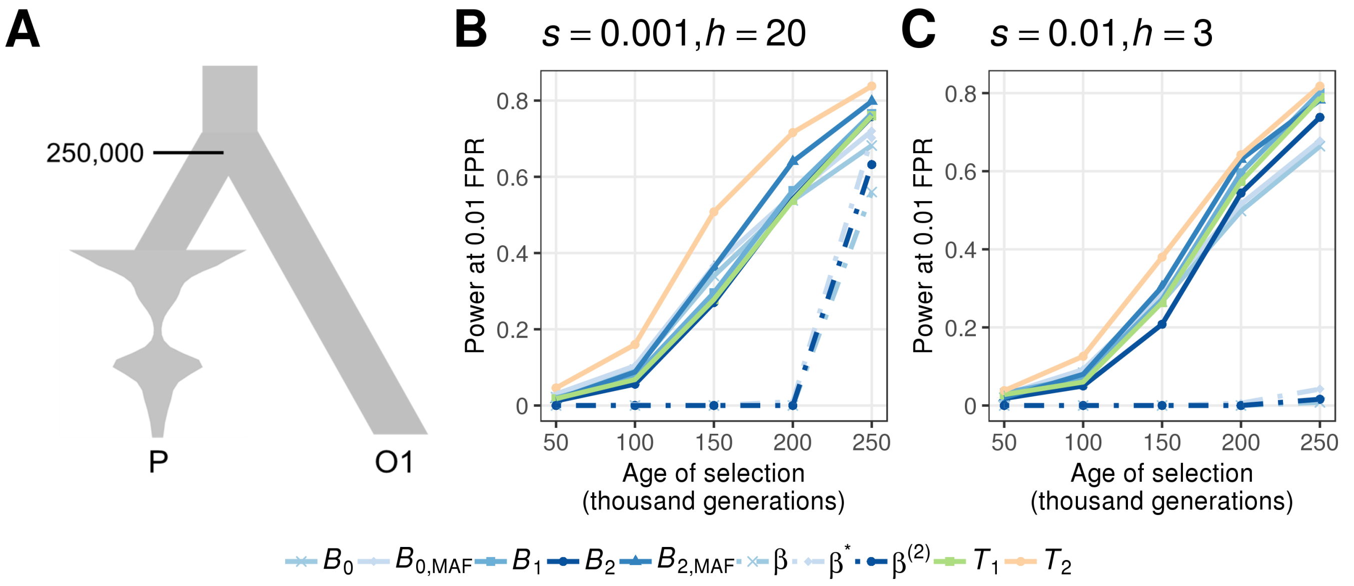

Figure S18: Power of B statistics when provided with identical window sizes as T statistics or β statistics, in comparison with other statistics for detecting balancing selection with varying age and parameters under the demographic history of bonobos. (A) Schematic of the demographic model of bonobos. (B, C) Power at a 1% false positive rate (FPR) of each statistic for detecting balancing selection with selection parameters (B) $s = 0.001$ with $h = 20$ and (C) $s = 0.01$ with $h = 3$.

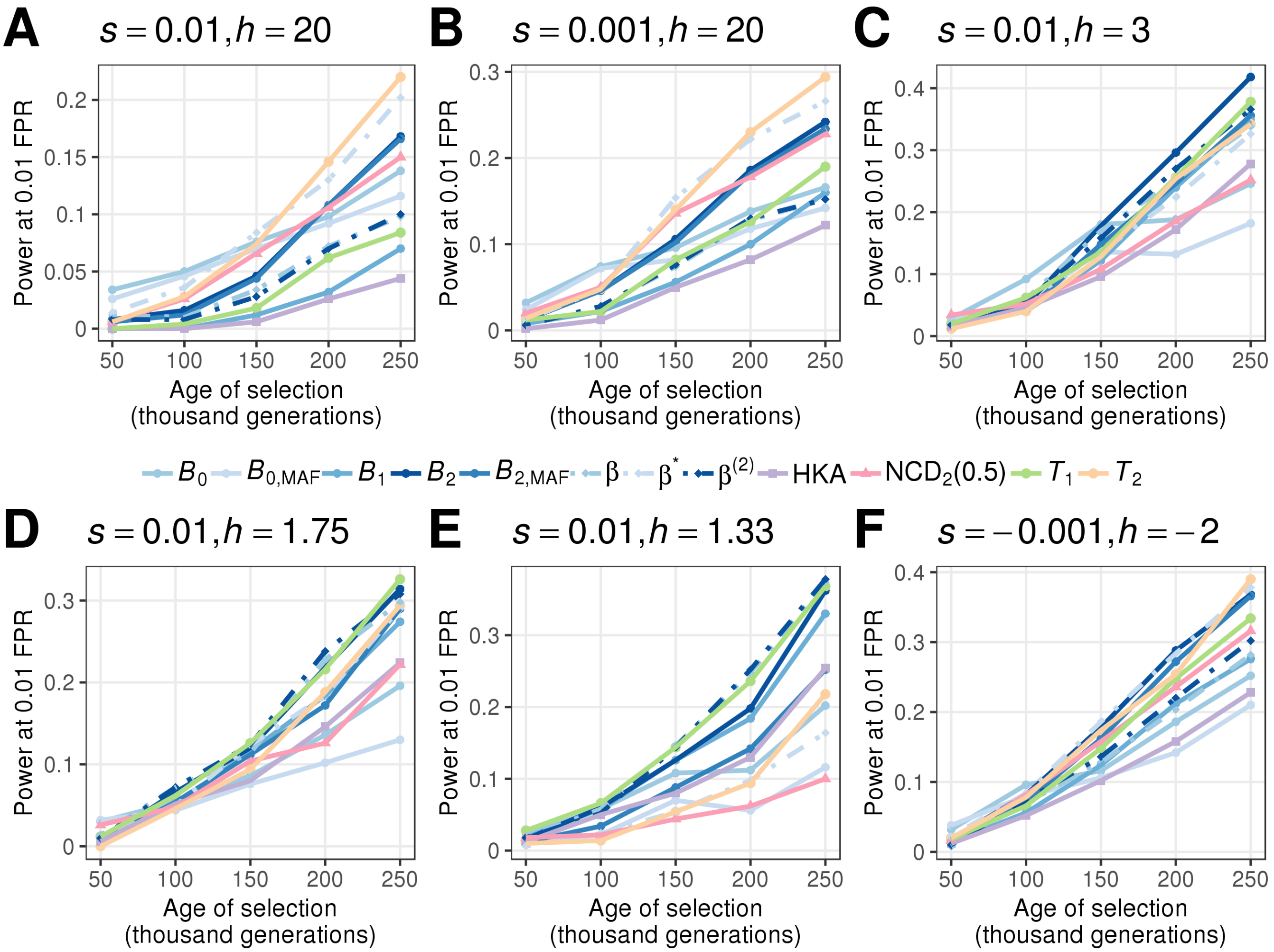

Figure S19: Powers at a 1% false positive rate (FPR) for each statistic as a function of age of the allele undergoing balancing selection for different selection (s) and dominance (h) coefficients when mutation rates vary between the target and outgroup species. The scenarios considered are (A) $s = 0.01$ with $h = 20$, (B) $s = 0.001$ with $h = 20$, (C) $s = 0.01$ with $h = 3$, (D) $s = 0.01$ with $h = 1.75$, (E) $s = 0.01$ with $h = 1.33$, and (F) $s = -0.001$ with $h = -2$.

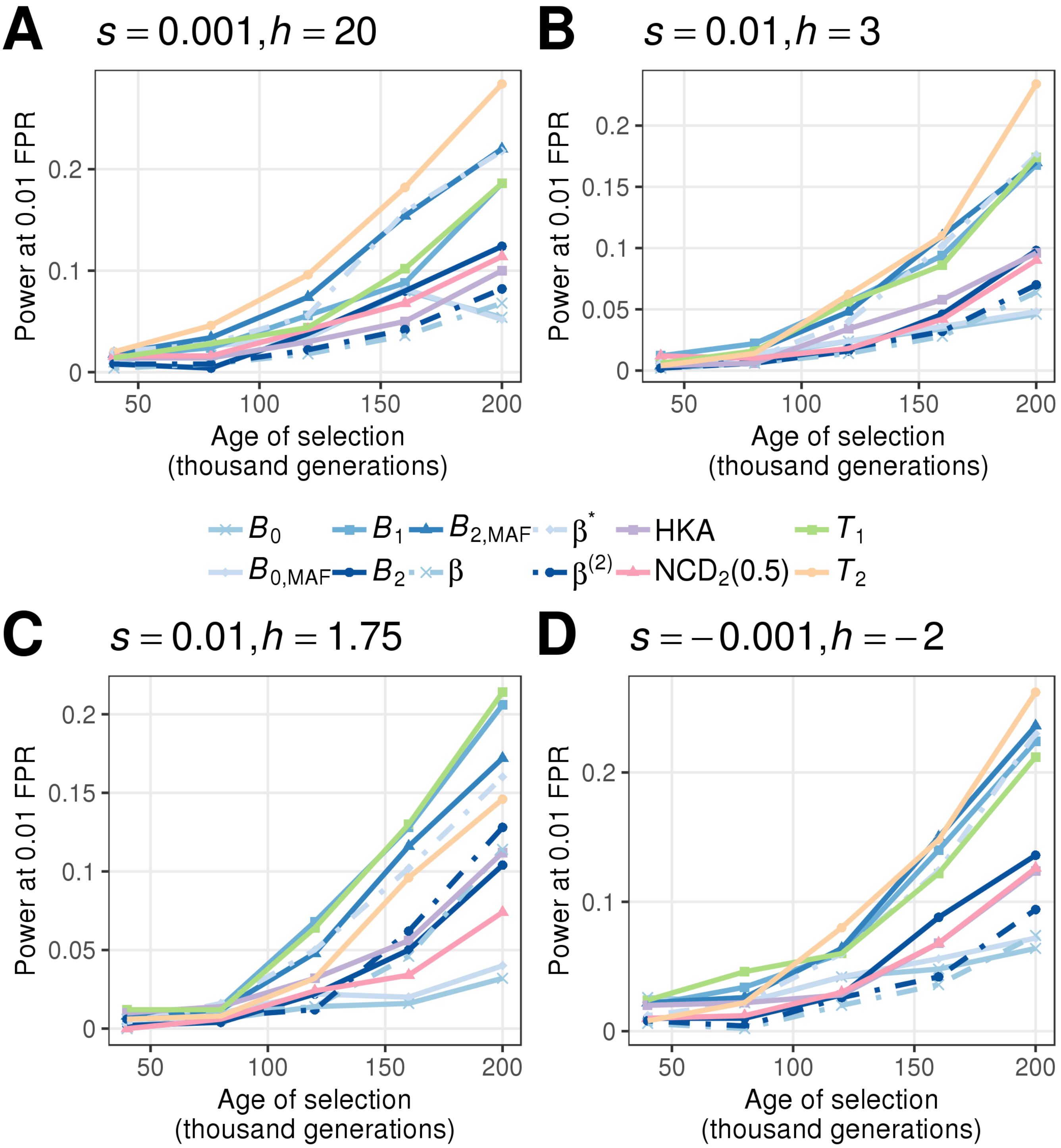

Figure S20: Powers at a 1% false positive rate (FPR) for each statistic as a function of age of the allele undergoing balancing selection for different selection (s) and dominance (h) coefficients when mutation rates vary between the target and outgroup species, and where the target species evolves under the demographic history of the CEU human population. The scenarios considered are (A) $s = 0.001$ with $h = 20$, (B) $s = 0.01$ with $h = 3$, (C) $s = 0.01$ with $h = 1.75$, and (D) $s = -0.001$ with $h = -2$.

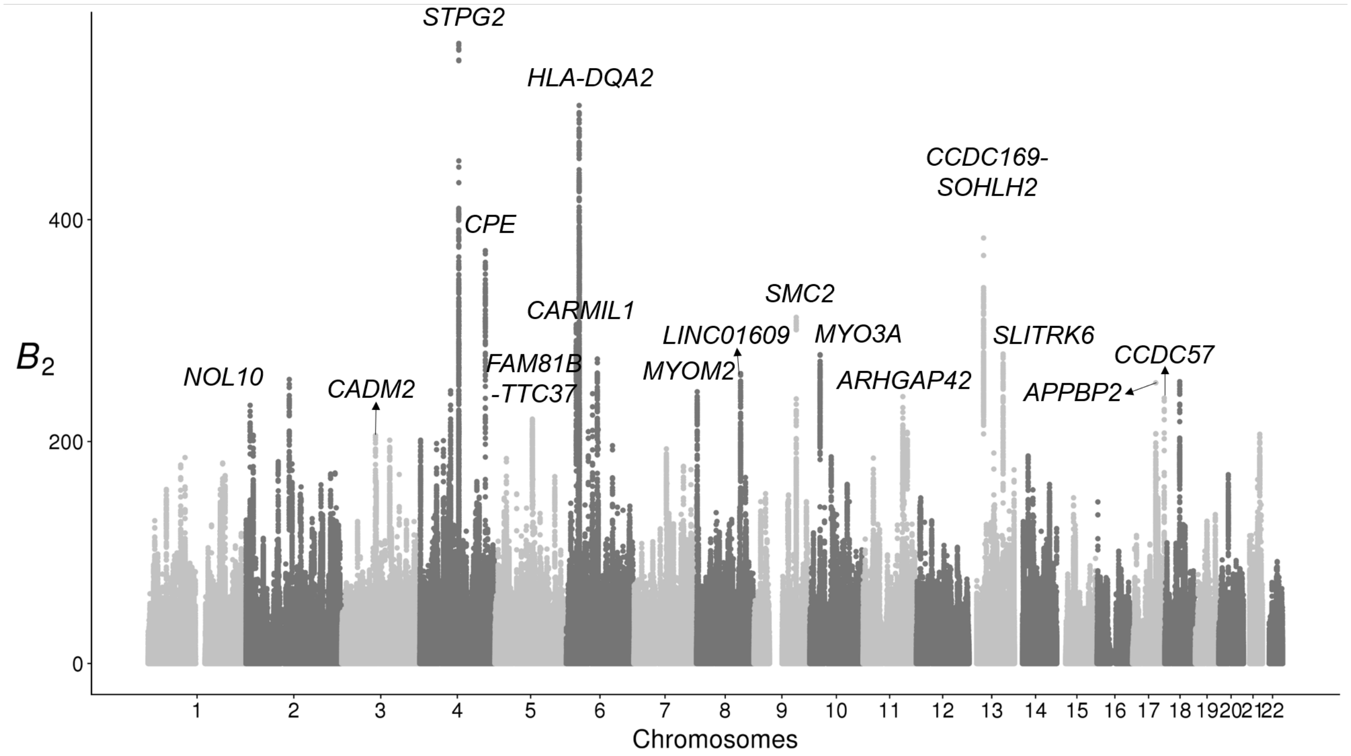

Figure S21: Manhattan plot displaying B_2 scores across the 22 human autosomes for the YRI population, with top candidates annotated. Outstanding peaks ($B_2 > 213$, top 0.01%) without annotations either fall in regions with abnormal sequencing depths or low sequence uniqueness, or do not neighbor any coding regions within a 100 kb radius.

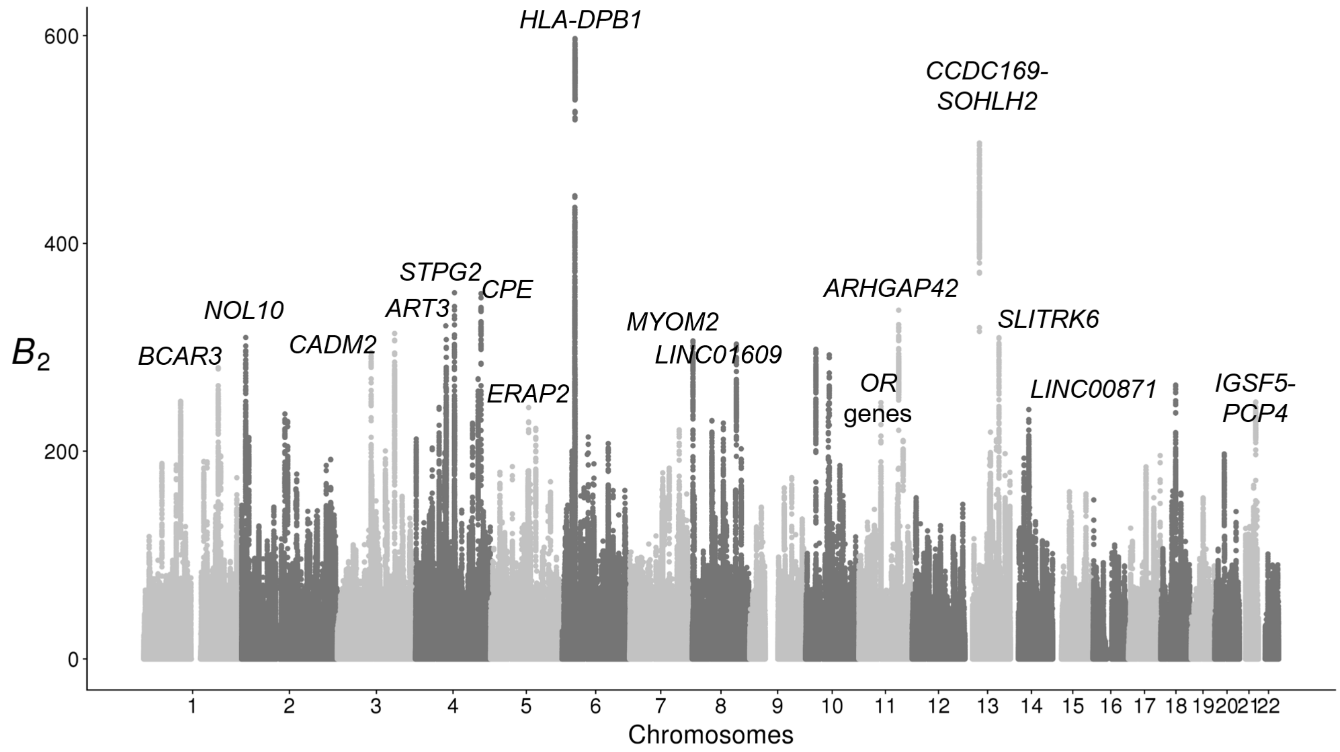

Figure S22: Manhattan plot displaying B_2 scores across the 22 human human autosomes for the CEU population, with top candidates annotated. Outstanding peaks ($B_2 > 241$, top 0.01%) without annotations either fall in regions with abnormal sequencing depths or low sequence uniqueness, or do not neighbor any coding regions within a 100 kb radius.

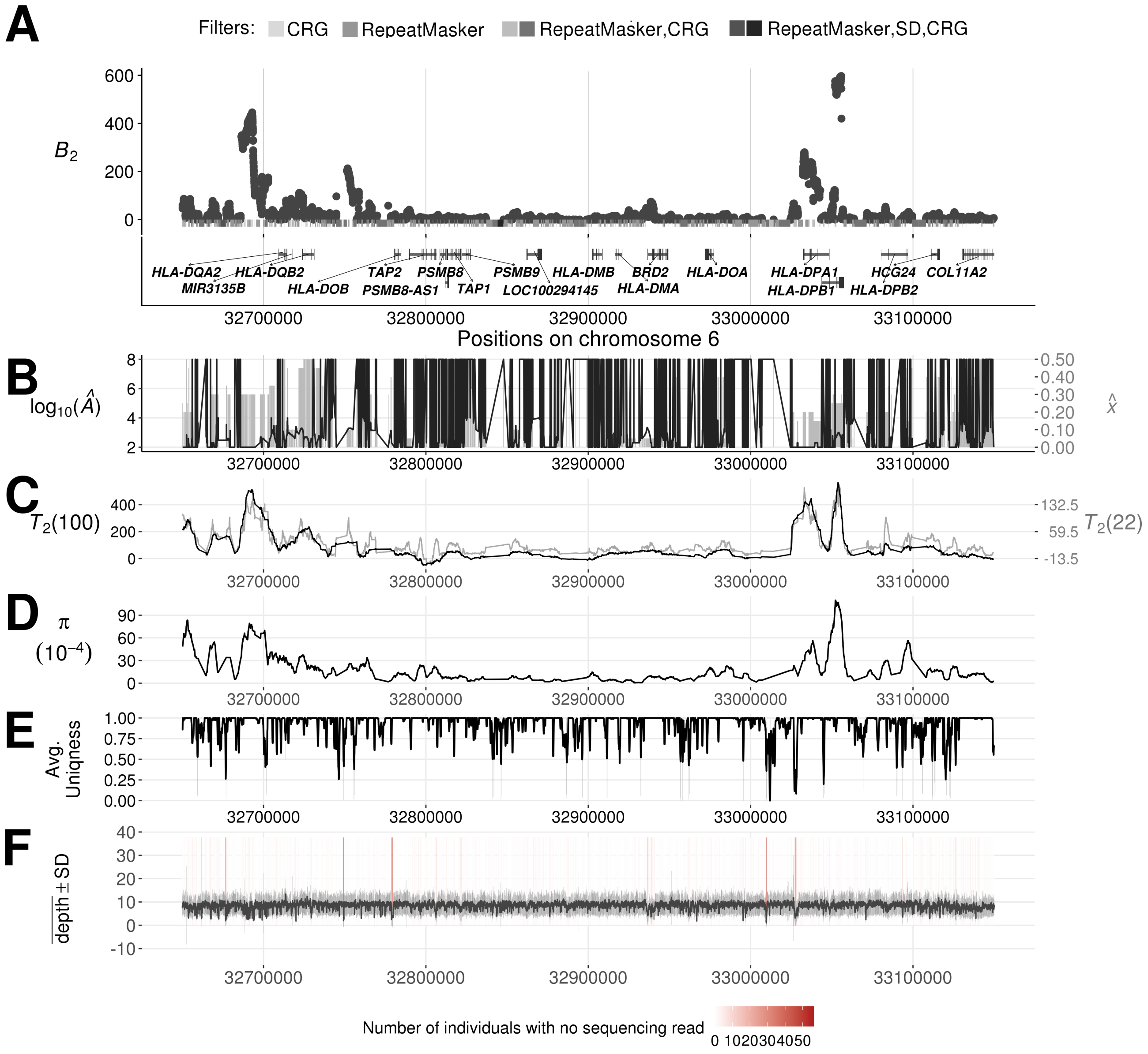

Figure S23: Evidence of balancing selection and sequencing quality around the MHC-D (HLA-D) region in the CEU population. (A) B_2 scores across the region, with gray bars indicating the regions removed by filters. (B) The optimal equilibrium frequency $\widehat{x}$ (gray bars) and $\log_{10}(\widehat{A})$ (black lines) for each maximized likelihood ratio. (C) T_2 scores across the region, with the black and gray lines respectively stand for the scans using 100 and 22 informative sites on either side of each test site. (D) Nucleotide diversity π computed from every window of length five kb across the region. (E) The 35 nucleotide sequence uniqueness, indicating mappability, averaged over windows of length 500 nucleotides across the region.

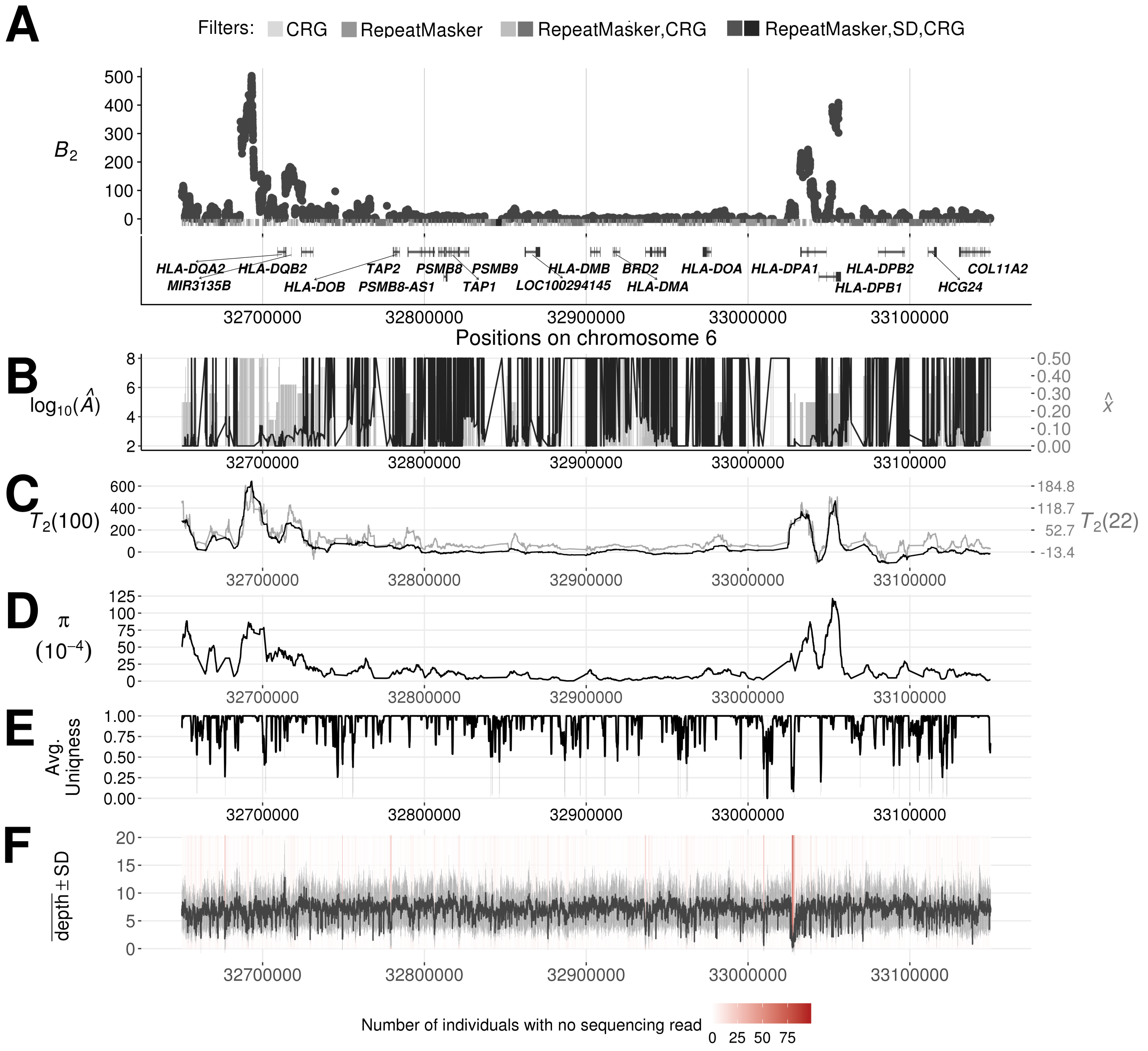

Figure S24: Evidence of balancing selection and sequencing quality around the MHC-D (HLA-D) region in the YRI population. (A) B_2 scores across the region, with gray bars indicating the regions removed by filters. (B) The optimal equilibrium frequency $\widehat{x}$ (gray bars) and $\log_{10}(\widehat{A})$ (black lines) for each maximized likelihood ratio. (C) T_2 scores across the region, with the black and gray lines respectively stand for the scans using 100 and 22 informative sites on either side of each test site. (D) Nucleotide diversity π computed from every window of length five kb across the region. (E) The 35 nucleotide sequence uniqueness, indicating mappability, averaged over windows of length 500 nucleotides across the region.

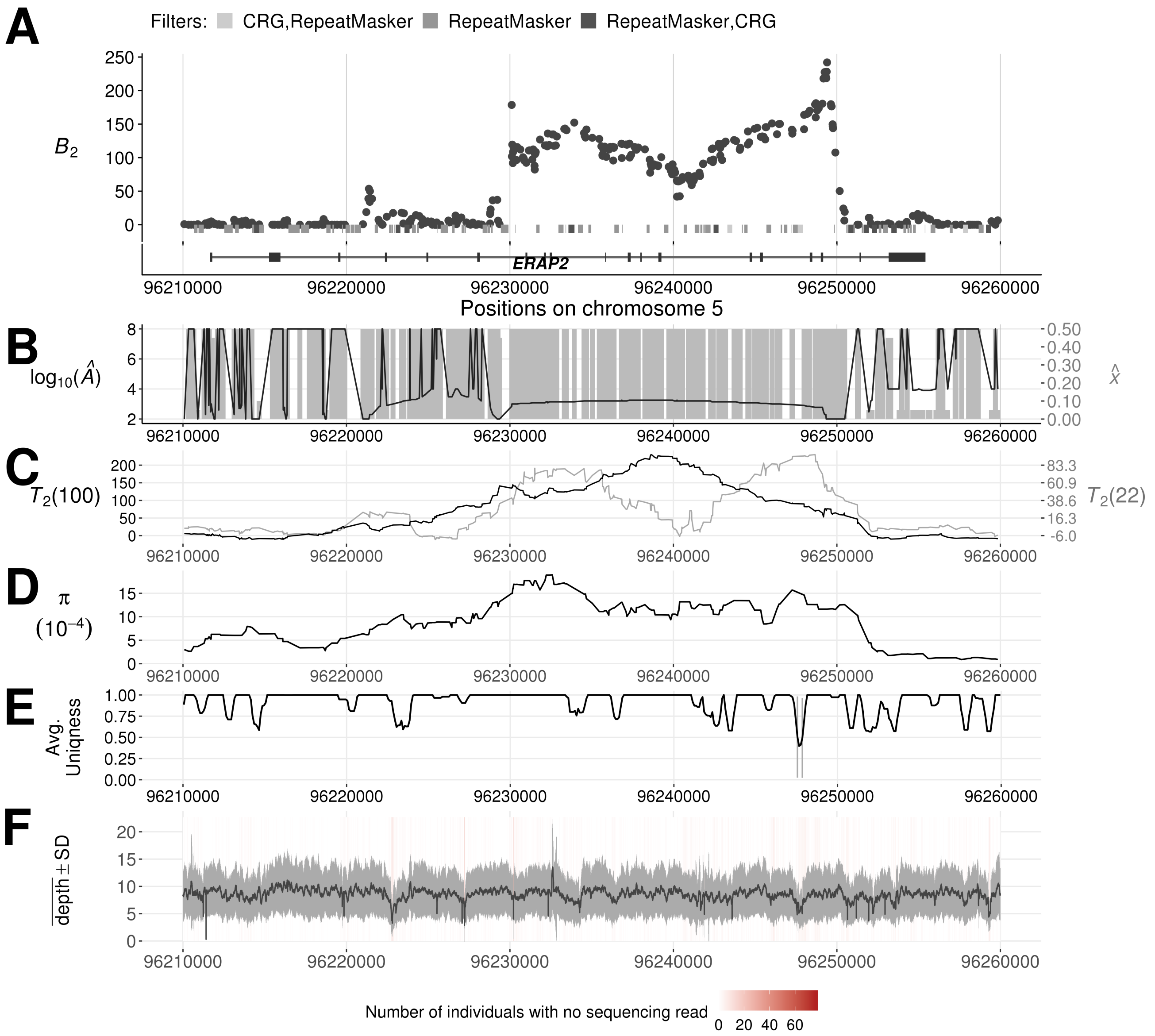

Figure S25: Evidence of balancing selection and sequencing quality across the *ERAP2* gene in the CEU population. (A) B_2 scores across the region, with gray bars indicating the regions removed by filters. (B) The optimal equilibrium frequency $\hat{x}$ (gray bars) and $\log_{10}(\widehat{A})$ (black lines) for each maximized likelihood ratio. (C) T_2 scores across the region, with the black and gray lines respectively stand for the scans using 100 and 22 informative sites on either side of each test site. (D) Nucleotide diversity π computed from every window of length five kb across the region. (E) The 35 nucleotide sequence uniqueness, indicating mappability, averaged over windows of length 500 nucleotides across the region.

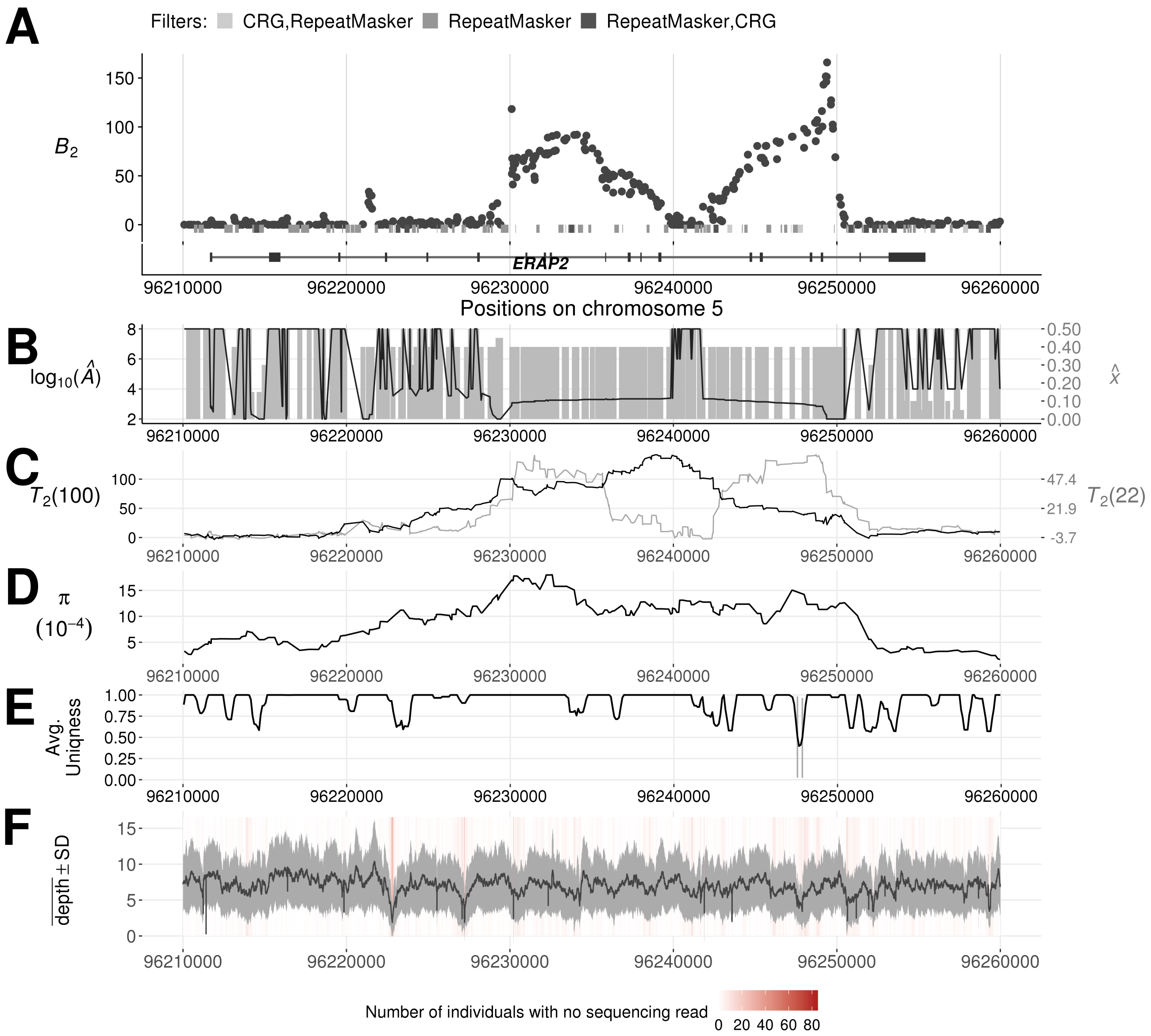

Figure S26: Evidence of balancing selection and sequencing quality across the *ERAP2* gene in the YRI population. (A) B_2 scores across the region, with gray bars indicating the regions removed by filters. (B) The optimal equilibrium frequency $\widehat{x}$ (gray bars) and $\log_{10}(\widehat{A})$ (black lines) for each maximized likelihood ratio. (C) T_2 scores across the region, with the black and gray lines respectively stand for the scans using 100 and 22 informative sites on either side of each test site. (D) Nucleotide diversity π computed from every window of length five kb across the region. (E) The 35 nucleotide sequence uniqueness, indicating mappability, averaged over windows of length 500 nucleotides across the region.

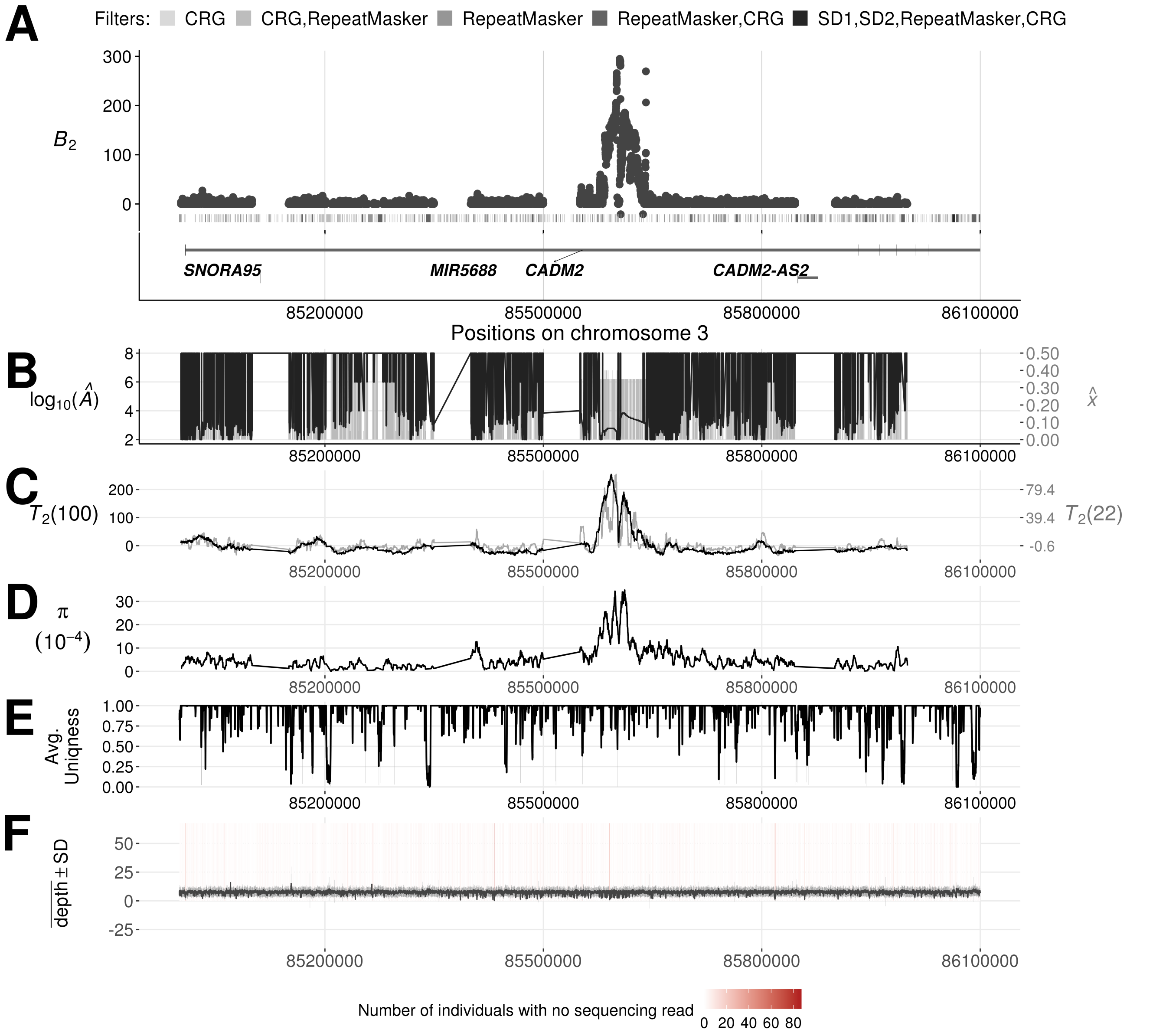

Figure S27: Evidence of balancing selection and sequencing quality across the *CADM2* gene in the CEU population. (A) B_2 scores across the region, with gray bars indicating the regions removed by filters. (B) The optimal equilibrium frequency $\widehat{x}$ (gray bars) and $\log_{10}(\widehat{A})$ (black lines) for each maximized likelihood ratio. (C) T_2 scores across the region, with the black and gray lines respectively stand for the scans using 100 and 22 informative sites on either side of each test site. (D) Nucleotide diversity π computed from every window of length five kb across the region. (E) The 35 nucleotide sequence uniqueness, indicating mappability, averaged over windows of length 500 nucleotides across the region.

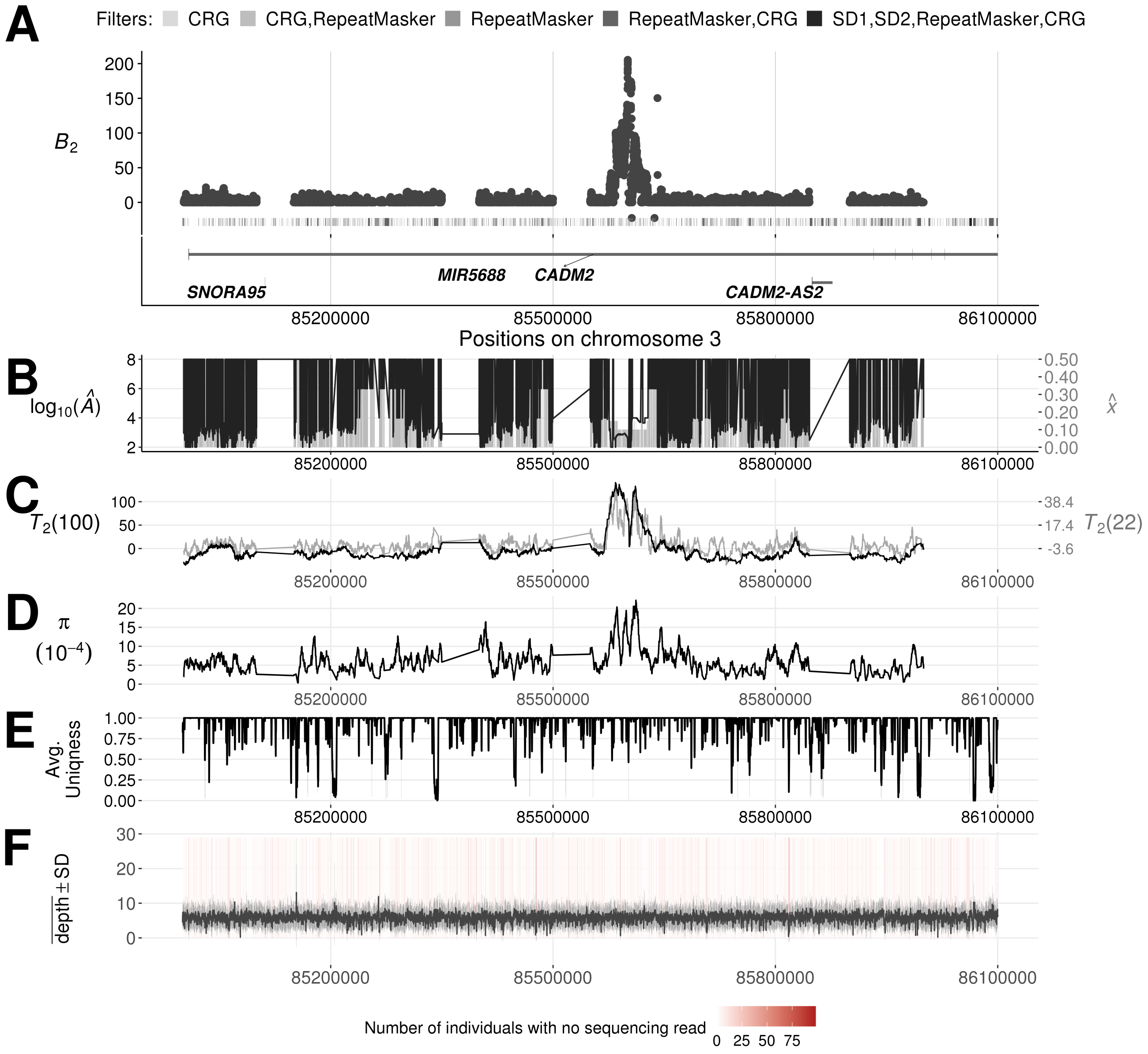

Figure S28: Evidence of balancing selection and sequencing quality across the *CADM2* gene in the YRI population. (A) B_2 scores across the region, with gray bars indicating the regions removed by filters. (B) The optimal equilibrium frequency $\widehat{x}$ (gray bars) and $\log_{10}(\widehat{A})$ (black lines) for each maximized likelihood ratio. (C) T_2 scores across the region, with the black and gray lines respectively stand for the scans using 100 and 22 informative sites on either side of each test site. (D) Nucleotide diversity π computed from every window of length five kb across the region. (E) The 35 nucleotide sequence uniqueness, indicating mappability, averaged over windows of length 500 nucleotides across the region.

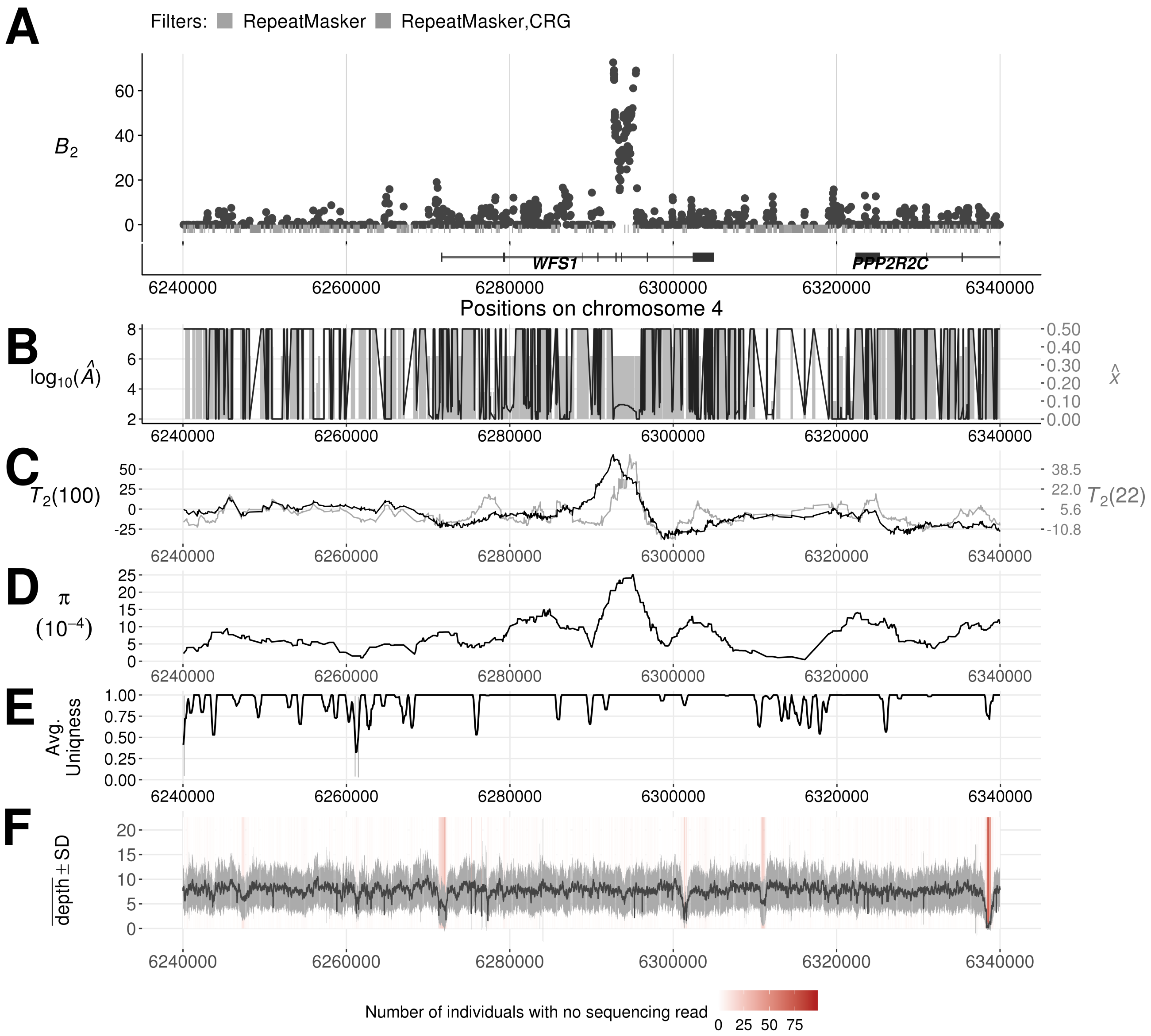

Figure S29: Evidence of balancing selection and sequencing quality around the *WFS1* gene in the CEU population. (A) B_2 scores across the region, with gray bars indicating the regions removed by filters. (B) The optimal equilibrium frequency $\widehat{x}$ (gray bars) and $\log_{10}(\widehat{A})$ (black lines) for each maximized likelihood ratio. (C) T_2 scores across the region, with the black and gray lines respectively stand for the scans using 100 and 22 informative sites on either side of each test site. (D) Nucleotide diversity π computed from every window of length five kb across the region. (E) The 35 nucleotide sequence uniqueness, indicating mappability, averaged over windows of length 500 nucleotides across the region.

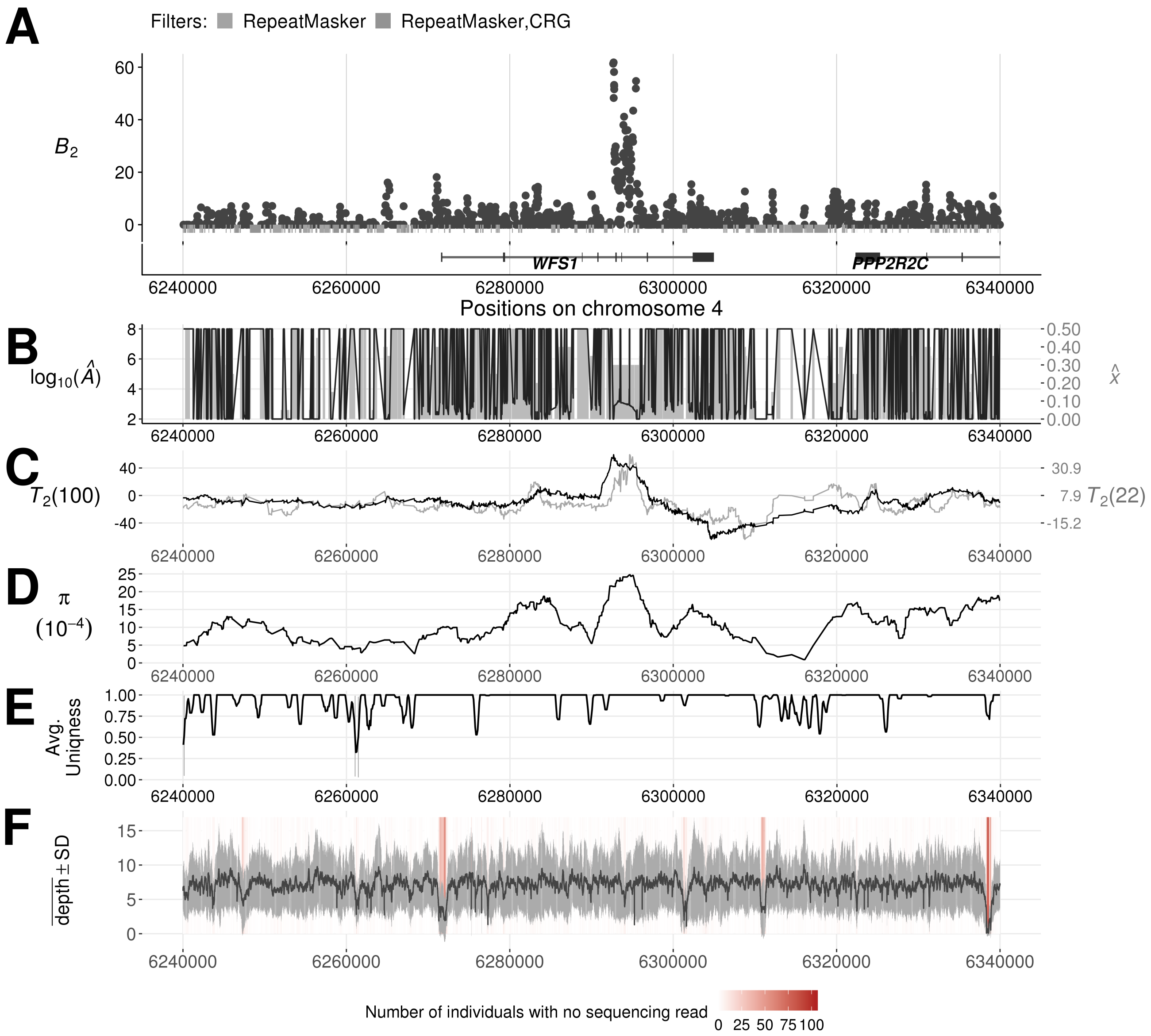

Figure S30: Evidence of balancing selection and sequencing quality around the *WFS1* gene in the YRI population. (A) B_2 scores across the region, with gray bars indicating the regions removed by filters. (B) The optimal equilibrium frequency $\widehat{x}$ (gray bars) and $\log_{10}(\widehat{A})$ (black lines) for each maximized likelihood ratio. (C) T_2 scores across the region, with the black and gray lines respectively stand for the scans using 100 and 22 informative sites on either side of each test site. (D) Nucleotide diversity π computed from every window of length five kb across the region. (E) The 35 nucleotide sequence uniqueness, indicating mappability, averaged over windows of length 500 nucleotides across the region.

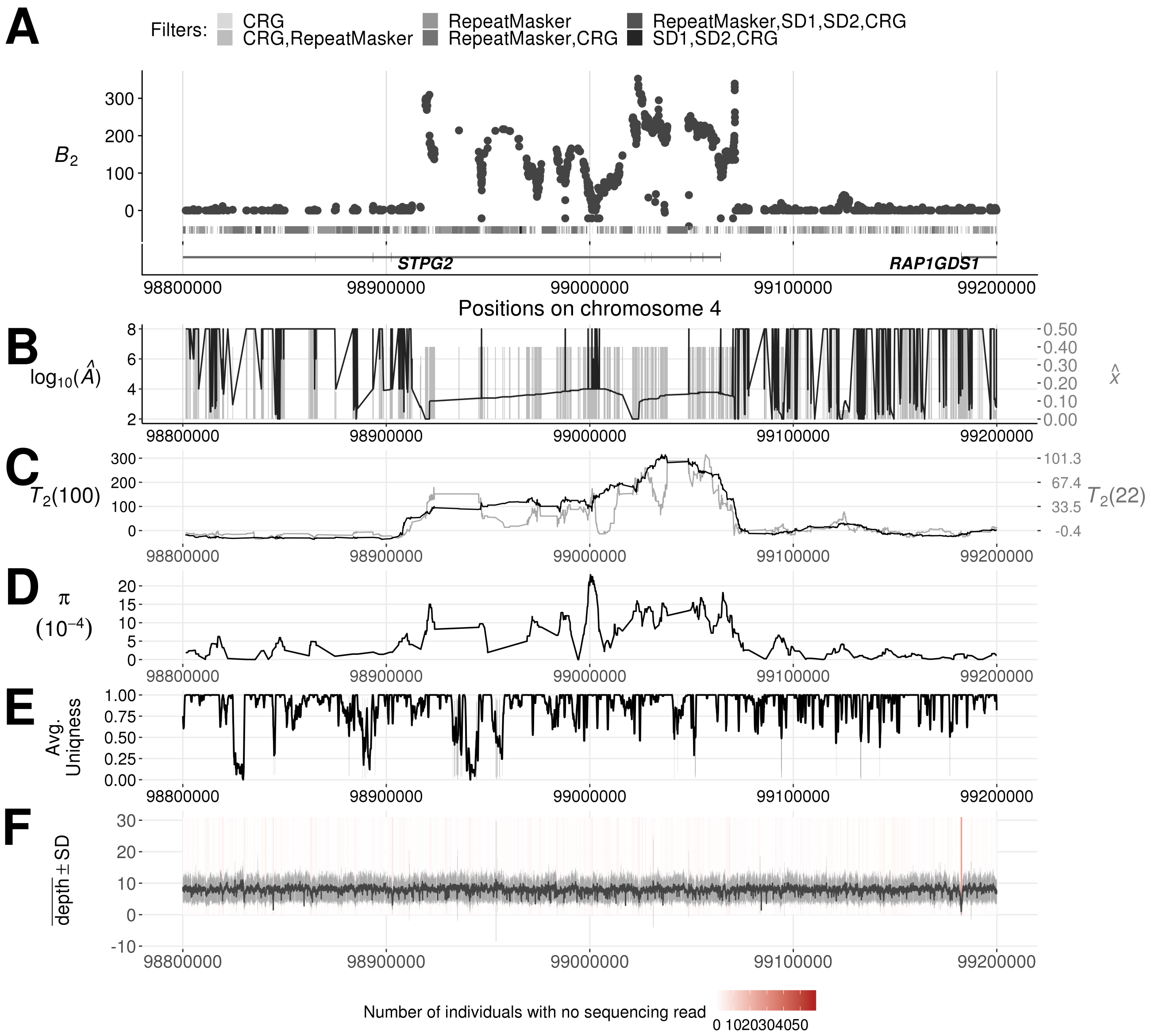

Figure S31: Evidence of balancing selection and sequencing quality around the *STPG2* gene in the CEU population. (A) B_2 scores across the region, with gray bars indicating the regions removed by filters. (B) The optimal equilibrium frequency $\widehat{x}$ (gray bars) and $\log_{10}(\widehat{A})$ (black lines) for each maximized likelihood ratio. (C) T_2 scores across the region, with the black and gray lines respectively stand for the scans using 100 and 22 informative sites on either side of each test site. (D) Nucleotide diversity π computed from every window of length five kb across the region. (E) The 35 nucleotide sequence uniqueness, indicating mappability, averaged over windows of length 500 nucleotides across the region.

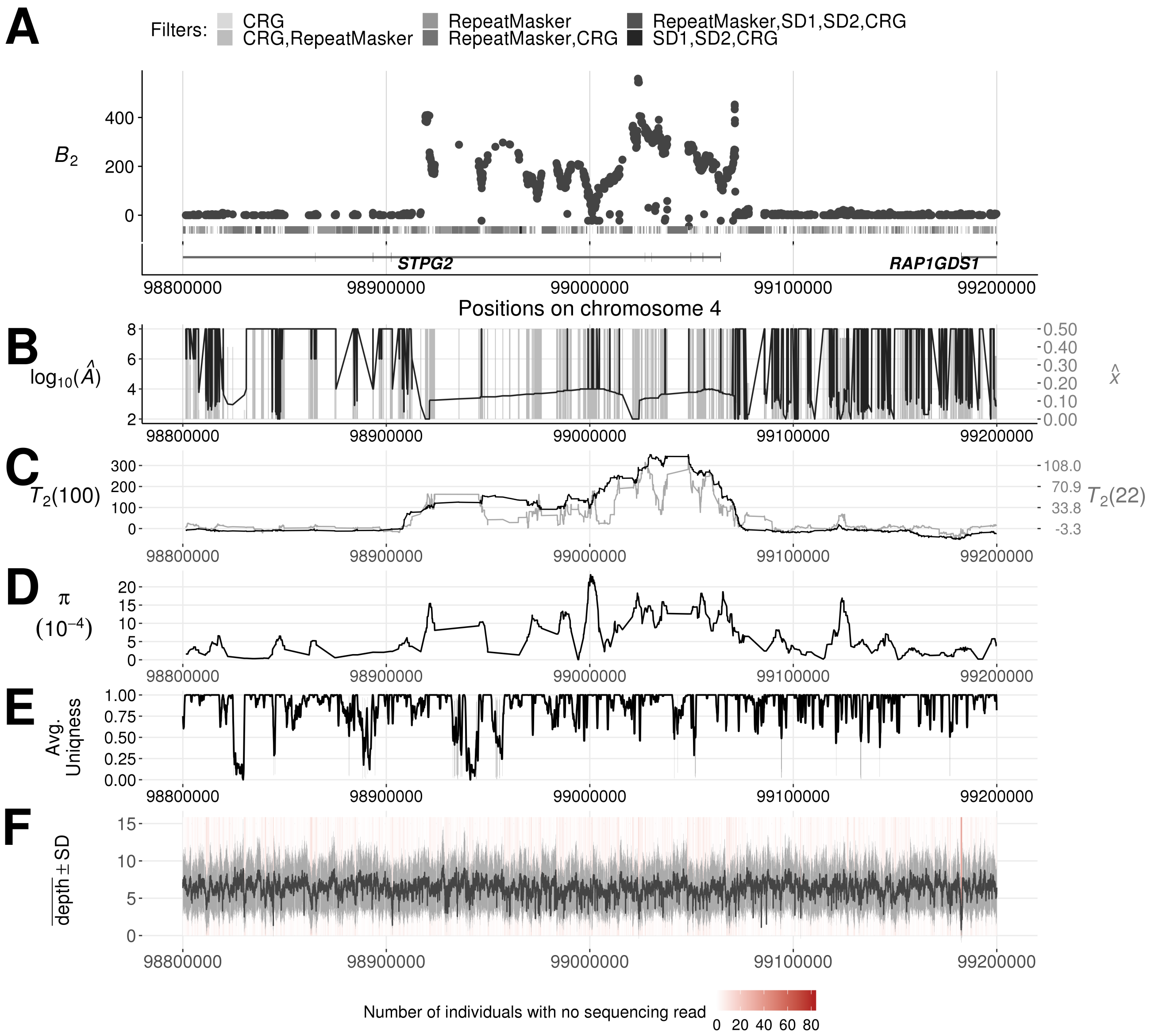

Figure S32: Evidence of balancing selection and sequencing quality around the *STPG2* gene in the YRI population. (A) B_2 scores across the region, with gray bars indicating the regions removed by filters. (B) The optimal equilibrium frequency $\widehat{x}$ (gray bars) and $\log_{10}(\widehat{A})$ (black lines) for each maximized likelihood ratio. (C) T_2 scores across the region, with the black and gray lines respectively stand for the scans using 100 and 22 informative sites on either side of each test site. (D) Nucleotide diversity π computed from every window of length five kb across the region. (E) The 35 nucleotide sequence uniqueness, indicating mappability, averaged over windows of length 500 nucleotides across the region.

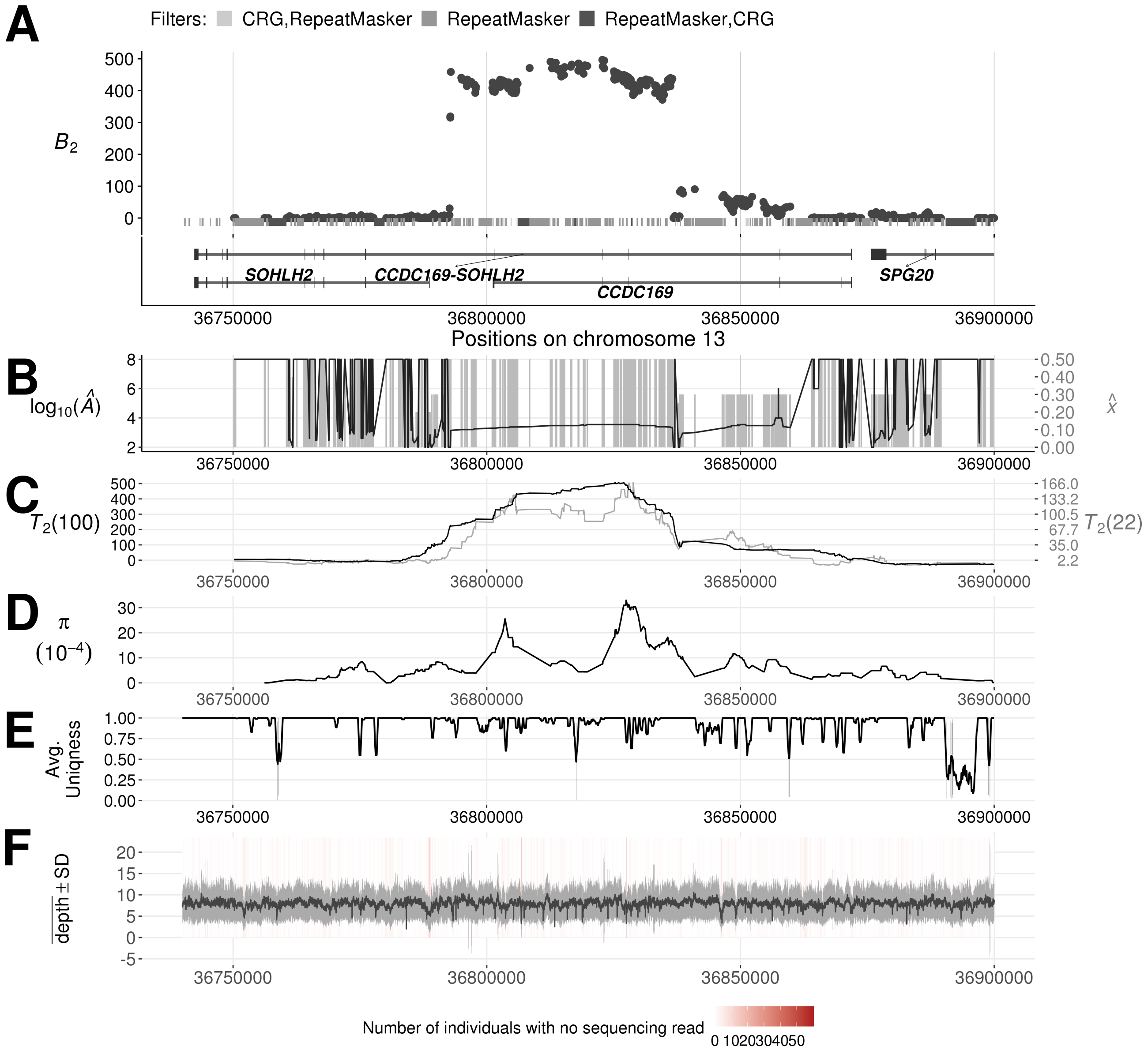

Figure S33: Evidence of balancing selection and sequencing quality across the *CCDC169-SOHLH2* region in the CEU population. (A) B_2 scores across the region, with gray bars indicating the regions removed by filters. (B) The optimal equilibrium frequency $\widehat{x}$ (gray bars) and $\log_{10}(\widehat{A})$ (black lines) for each maximized likelihood ratio. (C) T_2 scores across the region, with the black and gray lines respectively stand for the scans using 100 and 22 informative sites on either side of each test site. (D) Nucleotide diversity π computed from every window of length five kb across the region. (E) The 35 nucleotide sequence uniqueness, indicating mappability, averaged over windows of length 500 nucleotides across the region.

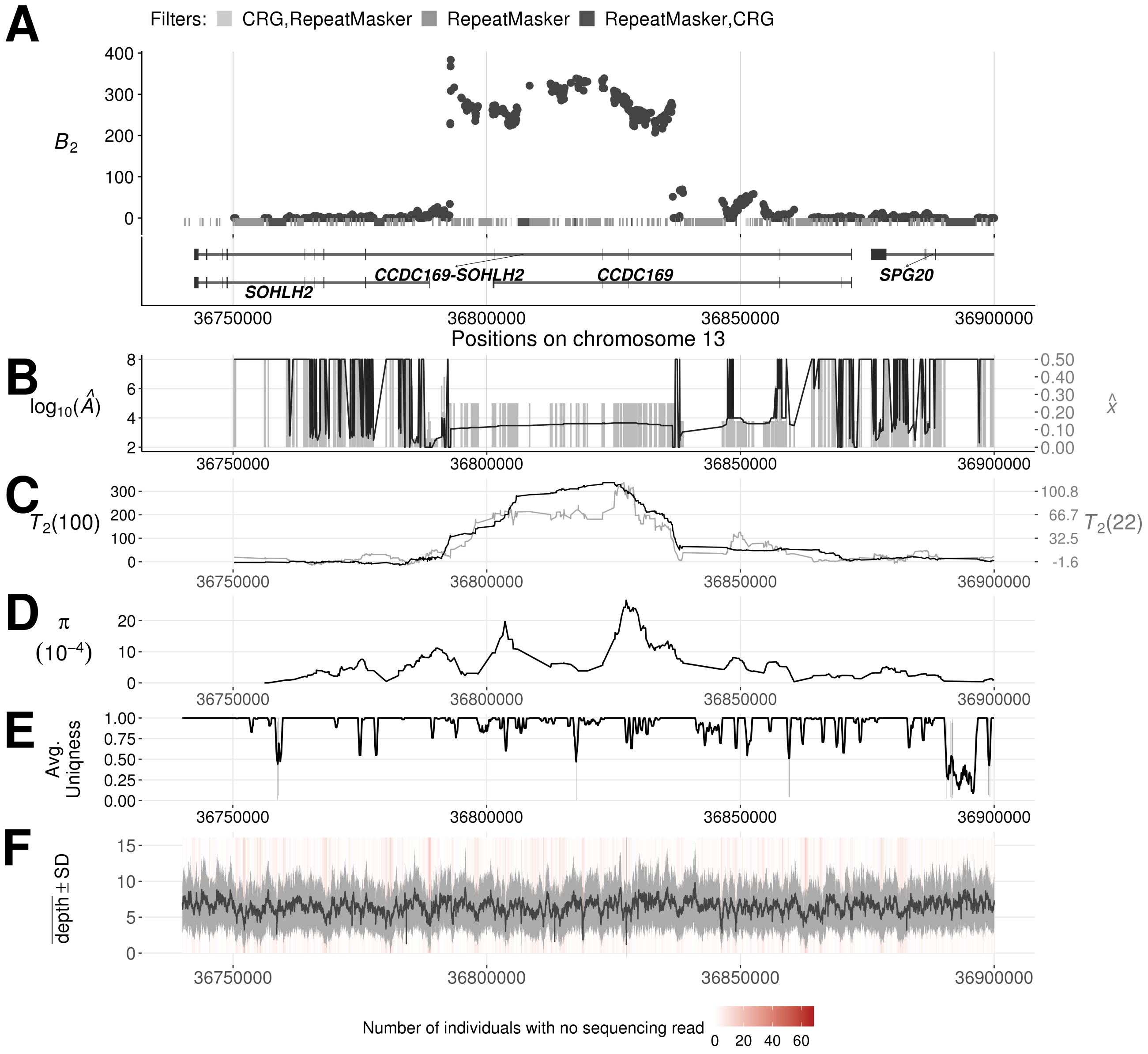

Figure S34: Evidence of balancing selection and sequencing quality across the *CCDC169-SOHLH2* region in the YRI population. (A) B_2 scores across the region, with gray bars indicating the regions removed by filters. (B) The optimal equilibrium frequency $\widehat{x}$ (gray bars) and $\log_{10}(\widehat{A})$ (black lines) for each maximized likelihood ratio. (C) T_2 scores across the region, with the black and gray lines respectively stand for the scans using 100 and 22 informative sites on either side of each test site. (D) Nucleotide diversity π computed from every window of length five kb across the region. (E) The 35 nucleotide sequence uniqueness, indicating mappability, averaged over windows of length 500 nucleotides across the region.

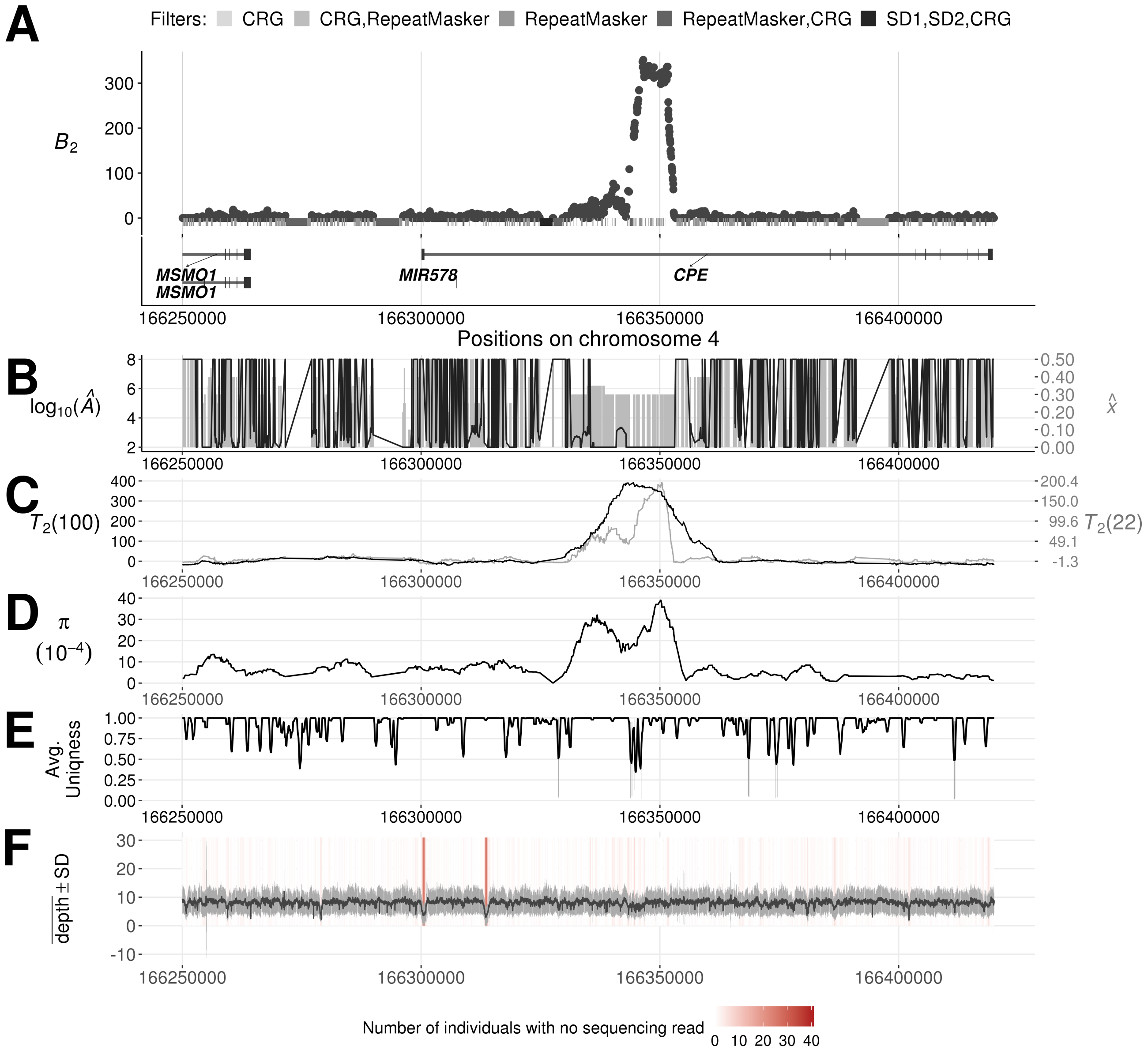

Figure S35: Evidence of balancing selection and sequencing quality around the *MYOM2* gene in the CEU population. (A) B_2 scores across the region, with gray bars indicating the regions removed by filters. (B) The optimal equilibrium frequency $\widehat{x}$ (gray bars) and $\log_{10}(\widehat{A})$ (black lines) for each maximized likelihood ratio. (C) T_2 scores across the region, with the black and gray lines respectively stand for the scans using 100 and 22 informative sites on either side of each test site. (D) Nucleotide diversity π computed from every window of length five kb across the region. (E) The 35 nucleotide sequence uniqueness, indicating mappability, averaged over windows of length 500 nucleotides across the region.

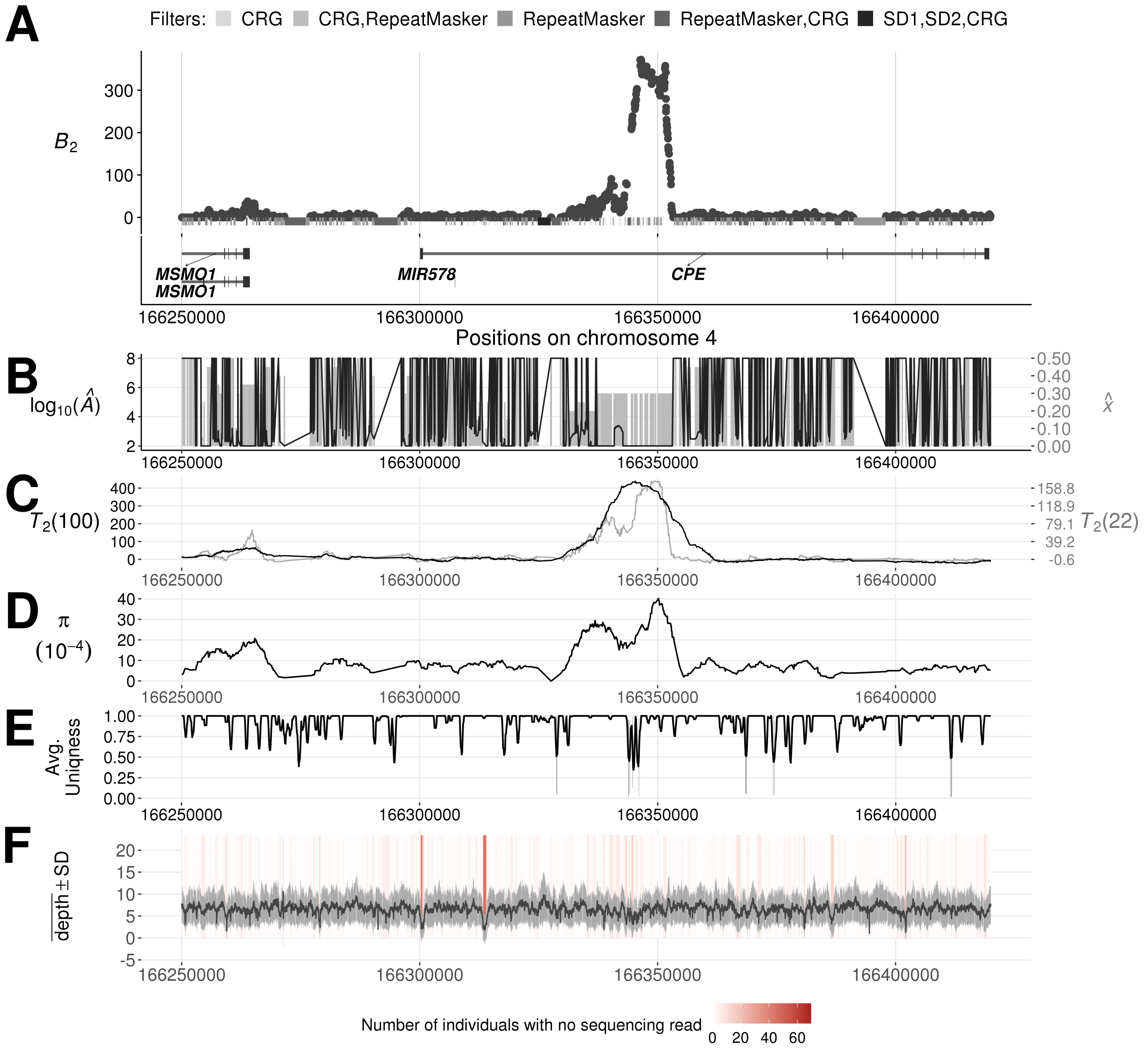

Figure S36: Evidence of balancing selection and sequencing quality around the *CPE* gene in the YRI population. (A) B_2 scores across the region, with gray bars indicating the regions removed by filters. (B) The optimal equilibrium frequency $\widehat{x}$ (gray bars) and $\log_{10}(\widehat{A})$ (black lines) for each maximized likelihood ratio. (C) T_2 scores across the region, with the black and gray lines respectively stand for the scans using 100 and 22 informative sites on either side of each test site. (D) Nucleotide diversity π computed from every window of length five kb across the region. (E) The 35 nucleotide sequence uniqueness, indicating mappability, averaged over windows of length 500 nucleotides across the region.

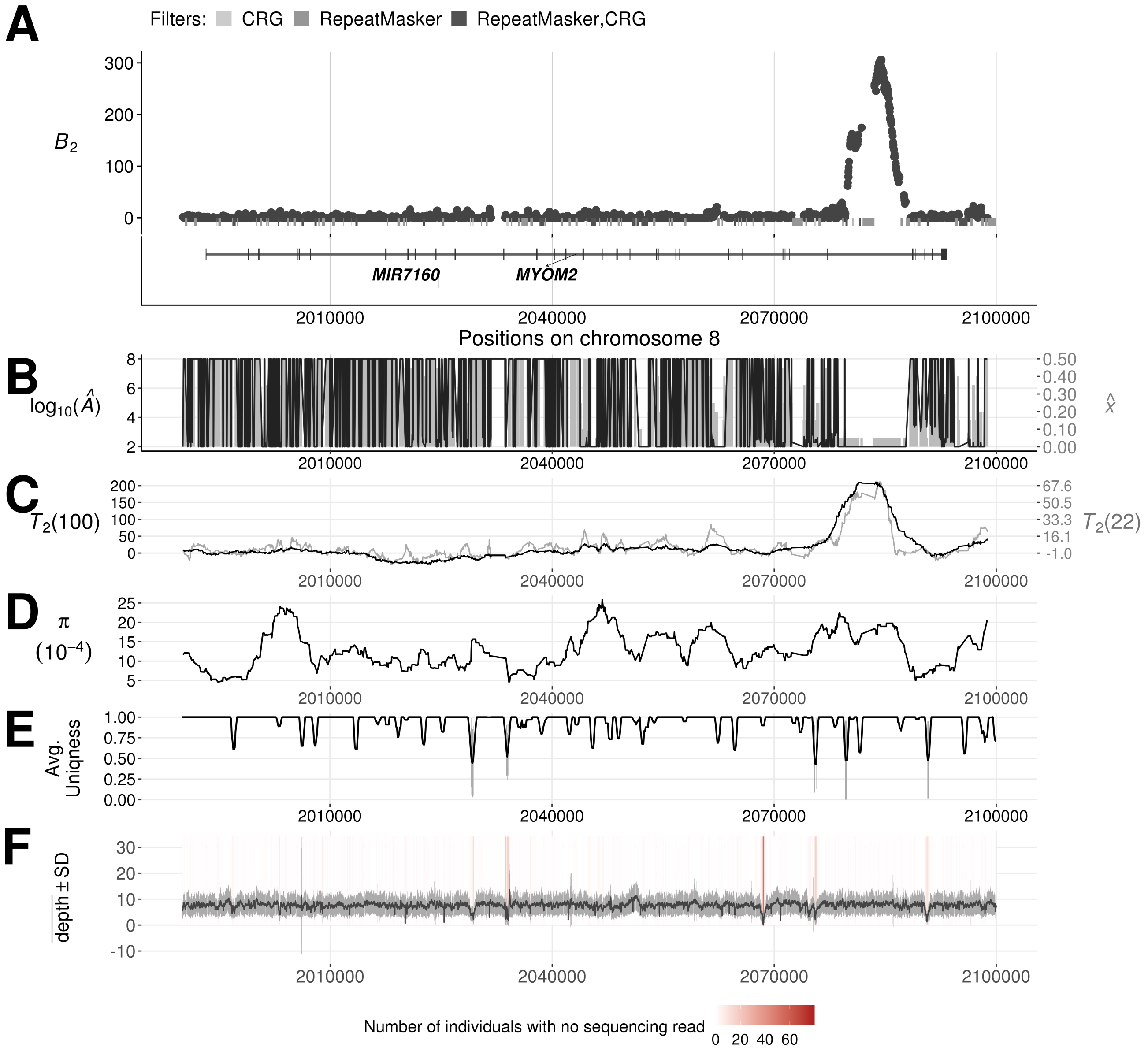

Figure S37: Evidence of balancing selection and sequencing quality around the *CPE* gene in the CEU population. (A) B_2 scores across the region, with gray bars indicating the regions removed by filters. (B) The optimal equilibrium frequency $\widehat{x}$ (gray bars) and $\log_{10}(\widehat{A})$ (black lines) for each maximized likelihood ratio. (C) T_2 scores across the region, with the black and gray lines respectively stand for the scans using 100 and 22 informative sites on either side of each test site. (D) Nucleotide diversity π computed from every window of length five kb across the region. (E) The 35 nucleotide sequence uniqueness, indicating mappability, averaged over windows of length 500 nucleotides across the region.

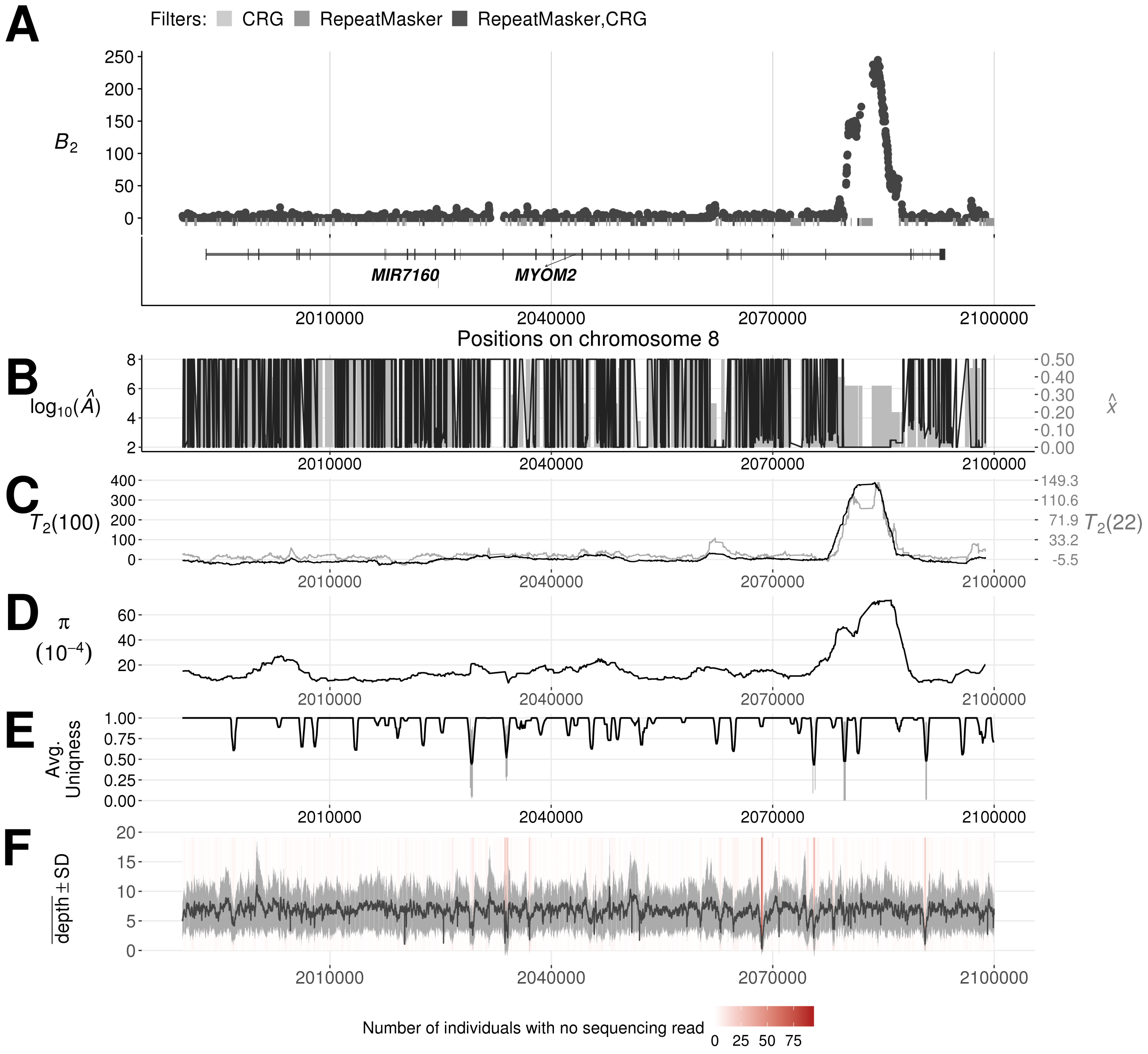

Figure S38: Evidence of balancing selection and sequencing quality around the *MYOM2* gene in the YRI population. (A) B_2 scores across the region, with gray bars indicating the regions removed by filters. (B) The optimal equilibrium frequency $\widehat{x}$ (gray bars) and $\log_{10}(\widehat{A})$ (black lines) for each maximized likelihood ratio. (C) T_2 scores across the region, with the black and gray lines respectively stand for the scans using 100 and 22 informative sites on either side of each test site. (D) Nucleotide diversity π computed from every window of length five kb across the region. (E) The 35 nucleotide sequence uniqueness, indicating mappability, averaged over windows of length 500 nucleotides across the region.

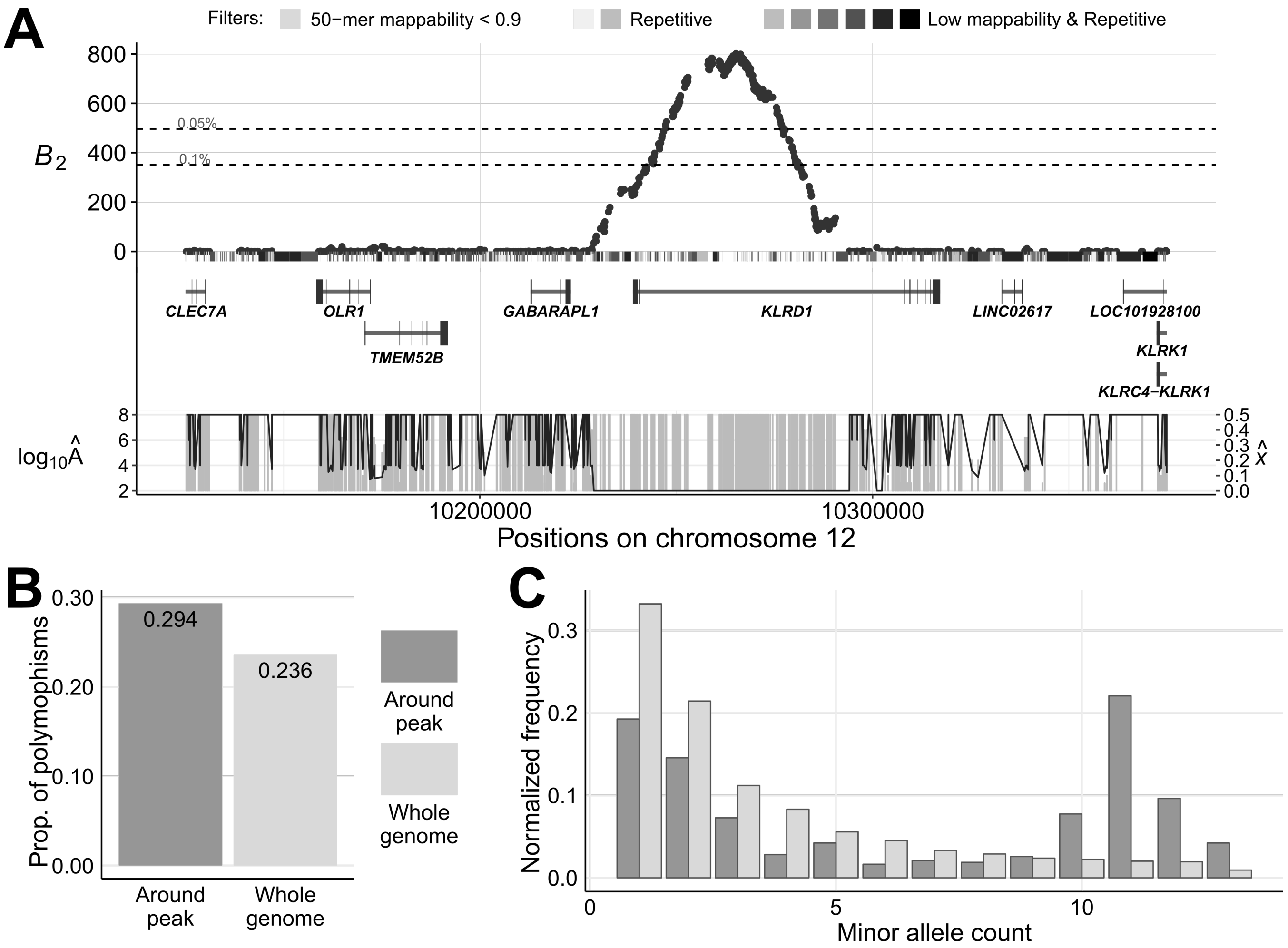

Figure S39: Evidence of balancing selection on *KLRD1*. (A) B_2 scores across the 250 kb genomic region on chromosome 12 centered on the peak. The gray bars directly under the B_2 scores represent the masked regions, as well as the features in these regions. The darker the shade, the greater number of types of repetitive sequences (*e.g.*, RepeatMasker mask, segmental duplication, simple repeats, or interrupted repeats) overlapping the region. Vertical gray bars below display the estimated equilibrium minor allele frequency $\widehat{x}$ for each maximum likelihood ratio B_2, and the black line traces the value for the respective inferred footprint size $\log_{10}(\widehat{A})$. (B) Proportion of informative sites that are polymorphic in the 250 kb region centered on the peak compared with the whole-genome average. (C) Minor allele frequency distribution in the 250 kb region centered on the peak compared with the whole-genome average.

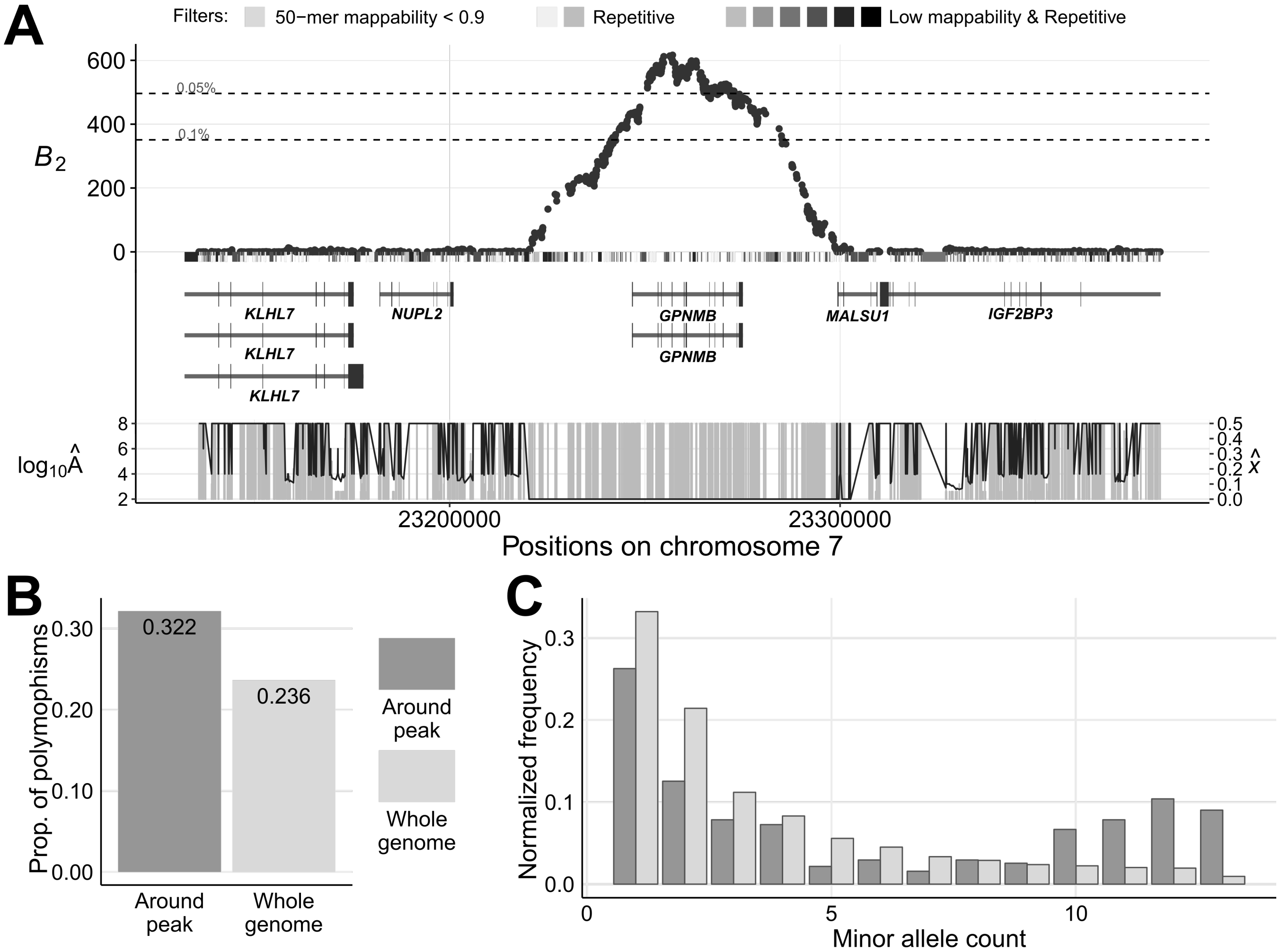

Figure S40: Evidence of balancing selection on *GPNMB*. (A) B_2 scores across the 250 kb genomic region on chromosome 7 centered on the peak. The gray bars directly under the B_2 scores represent the masked regions, as well as the features in these regions. The darker the shade, the greater number of types of repetitive sequences (*e.g.*, RepeatMasker mask, segmental duplication, simple repeats, or interrupted repeats) overlapping the region. Vertical gray bars below display the estimated equilibrium minor allele frequency $\widehat{x}$ for each maximum likelihood ratio B_2, and the black line traces the value for the respective inferred footprint size $\log_{10}(\widehat{A})$. (B) Proportion of informative sites that are polymorphic in the 250 kb region centered on the peak compared with the whole-genome average. (C) Minor allele frequency distribution in the 250 kb region centered on the peak compared with the whole-genome average.

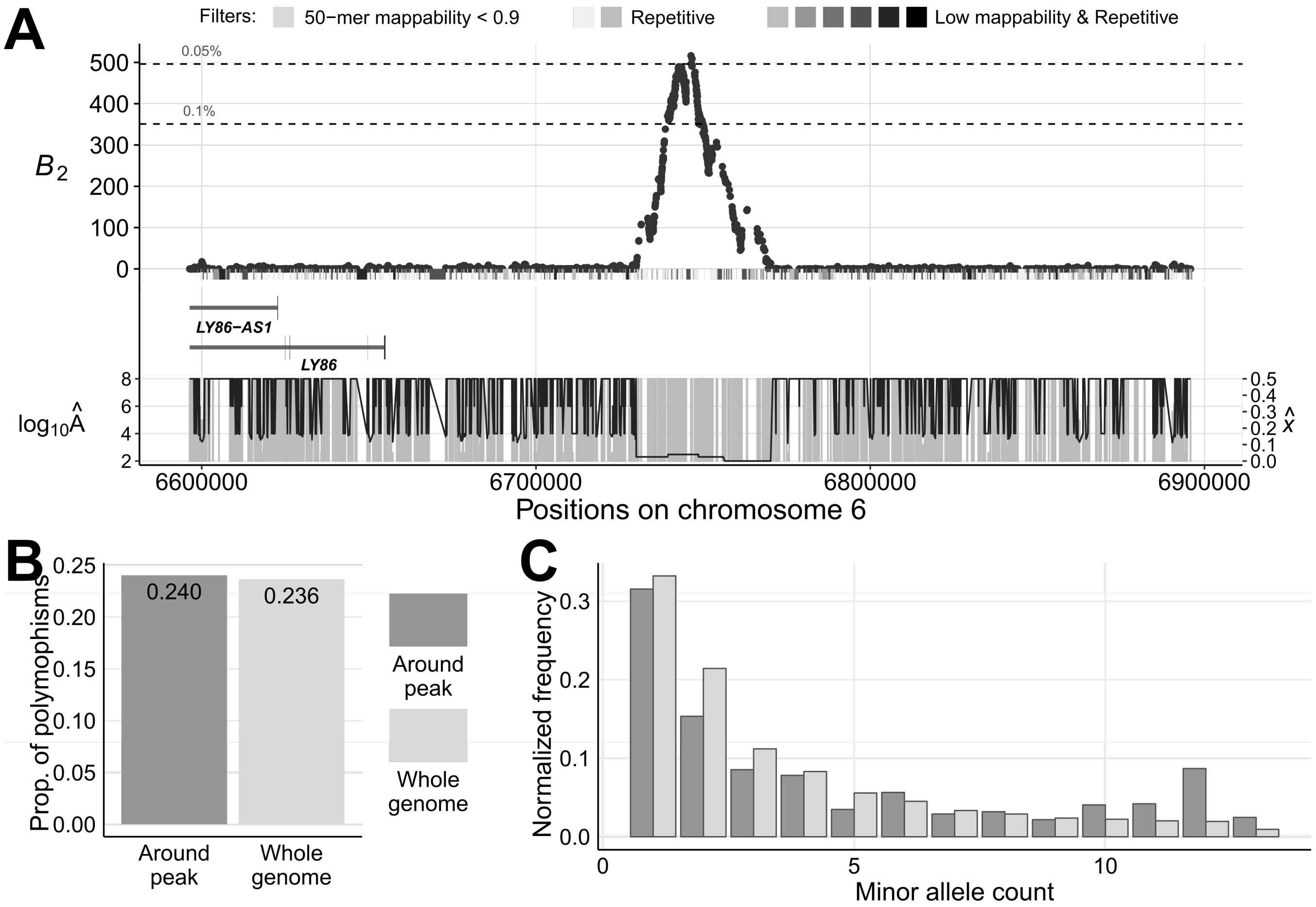

Figure S41: Evidence of balancing selection near *LY86*. (A) B_2 scores across the 300 kb genomic region on chromosome 6 centered on the peak. The gray bars directly under the B_2 scores represent the masked regions, as well as the features in these regions. The darker the shade, the greater number of types of repetitive sequences (*e.g.*, RepeatMasker mask, segmental duplication, simple repeats, or interrupted repeats) overlapping the region. Vertical gray bars below display the estimated equilibrium minor allele frequency $\widehat{x}$ for each maximum likelihood ratio B_2, and the black line traces the value for the respective inferred footprint size $\log_{10}(\widehat{A})$. (B) Proportion of informative sites that are polymorphic in the 300 kb region centered on the peak compared with the whole-genome average. (C) Minor allele frequency distribution in the 300 kb region centered on the peak compared with the whole-genome average.

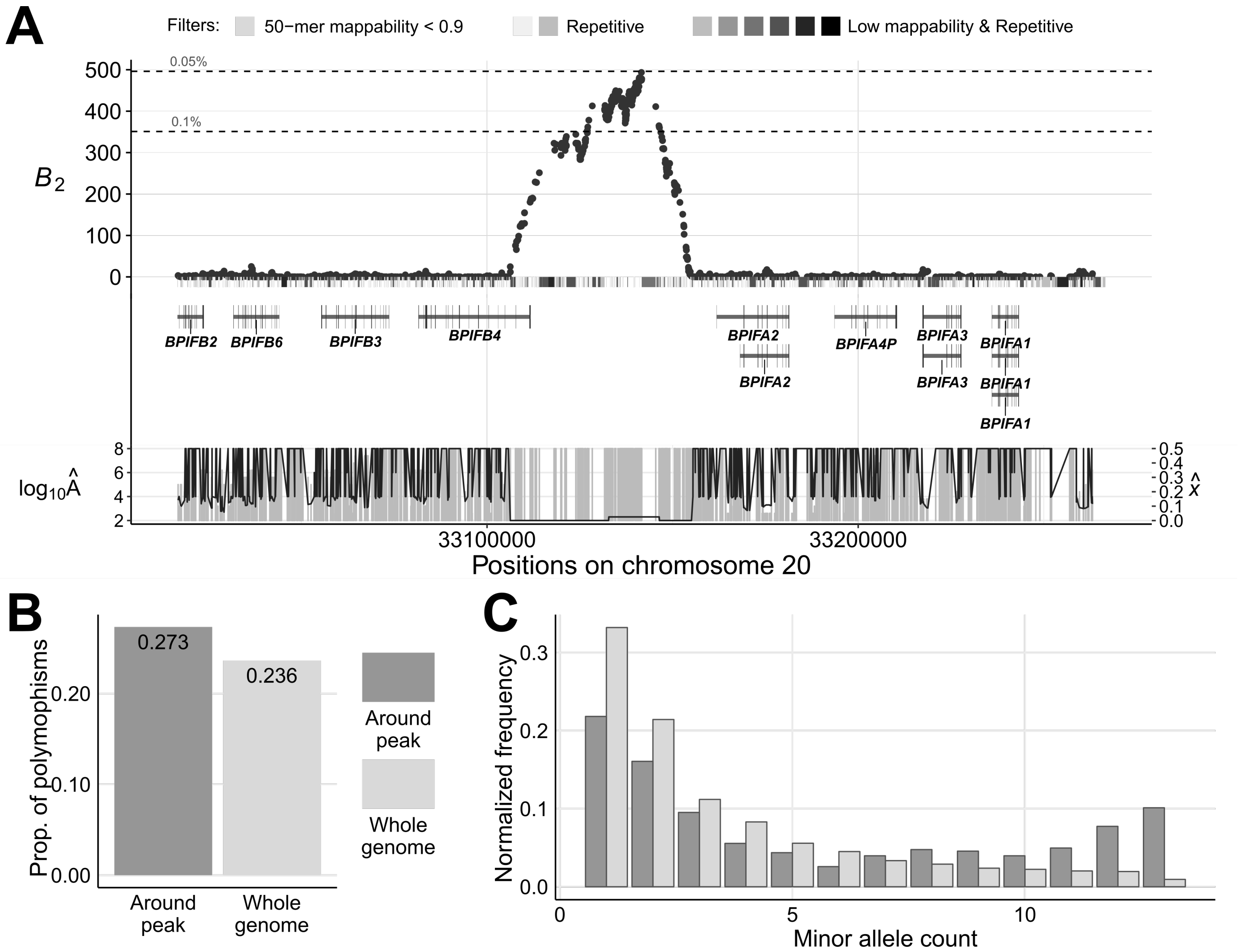

Figure S42: Evidence of balancing selection on the *BPIFB2/BPIFA4* intergenic region. (A) B_2 scores across the 250 kb genomic region on chromosome 20 centered on the peak. The gray bars directly under the B_2 scores represent the masked regions, as well as the features in these regions. The darker the shade, the greater number of types of repetitive sequences (*e.g.*, RepeatMasker mask, segmental duplication, simple repeats, or interrupted repeats) overlapping the region. Vertical gray bars below display the estimated equilibrium minor allele frequency $\widehat{x}$ for each maximum likelihood ratio B_2, and the black line traces the value for the respective inferred footprint size $\log_{10}(\widehat{A})$. (B) Proportion of informative sites that are polymorphic in the 250 kb region centered on the peak compared with the whole-genome average. (C) Minor allele frequency distribution in the 250 kb region centered on the peak compared with the whole-genome average.

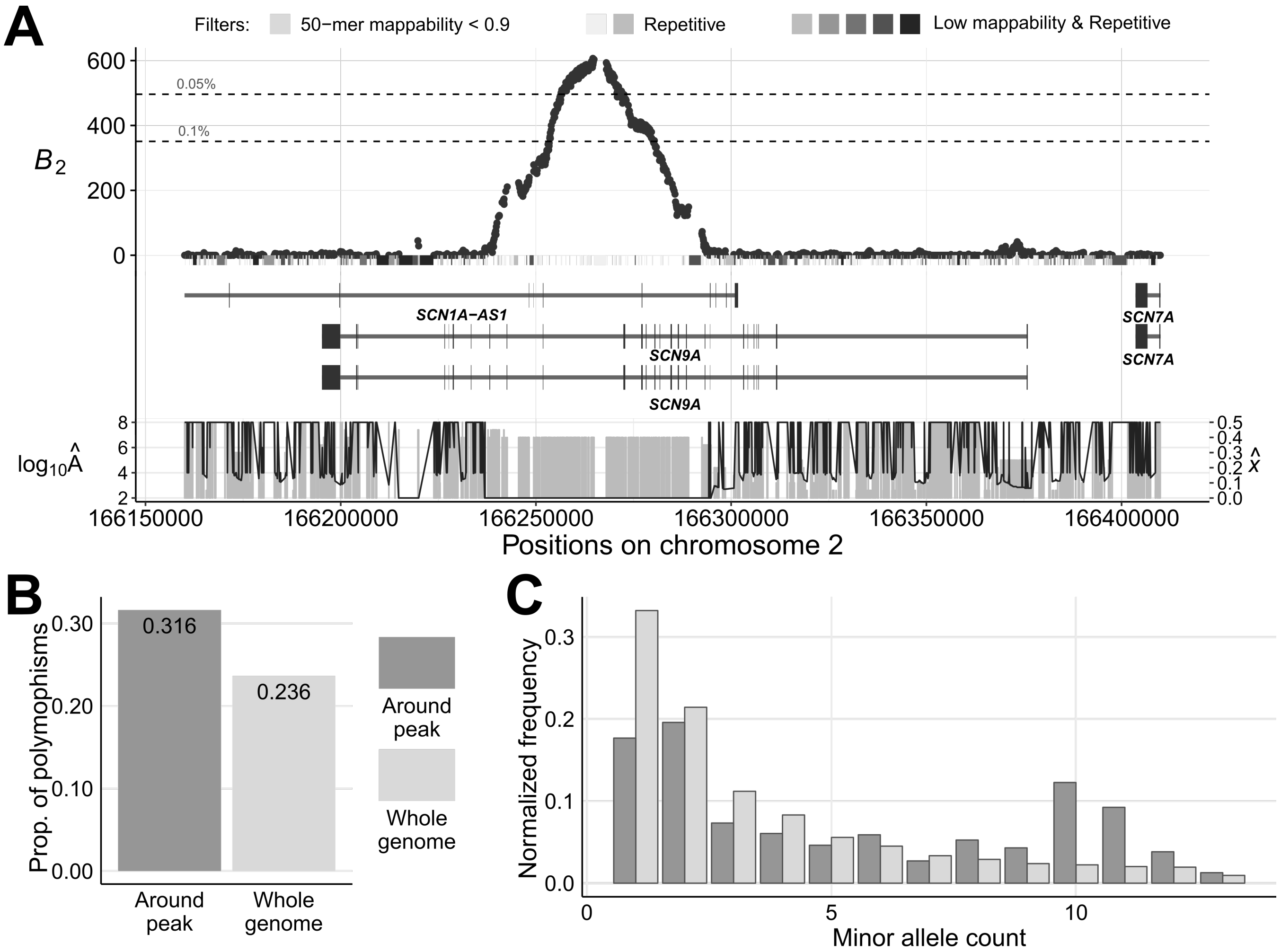

Figure S43: Evidence of balancing selection on *SCN9A*. (A) B_2 scores across the 250 kb genomic region on chromosome 2 centered on the peak. The gray bars directly under the B_2 scores represent the masked regions, as well as the features in these regions. The darker the shade, the greater number of types of repetitive sequences (*e.g.*, RepeatMasker mask, segmental duplication, simple repeats, or interrupted repeats) overlapping the region. Vertical gray bars below display the estimated equilibrium minor allele frequency $\widehat{x}$ for each maximum likelihood ratio B_2, and the black line traces the value for the respective inferred footprint size $\log_{10}(\widehat{A})$. (B) Proportion of informative sites that are polymorphic in the 250 kb region centered on the peak compared with the whole-genome average. (C) Minor allele frequency distribution in the 250 kb region centered on the peak compared with the whole-genome average.

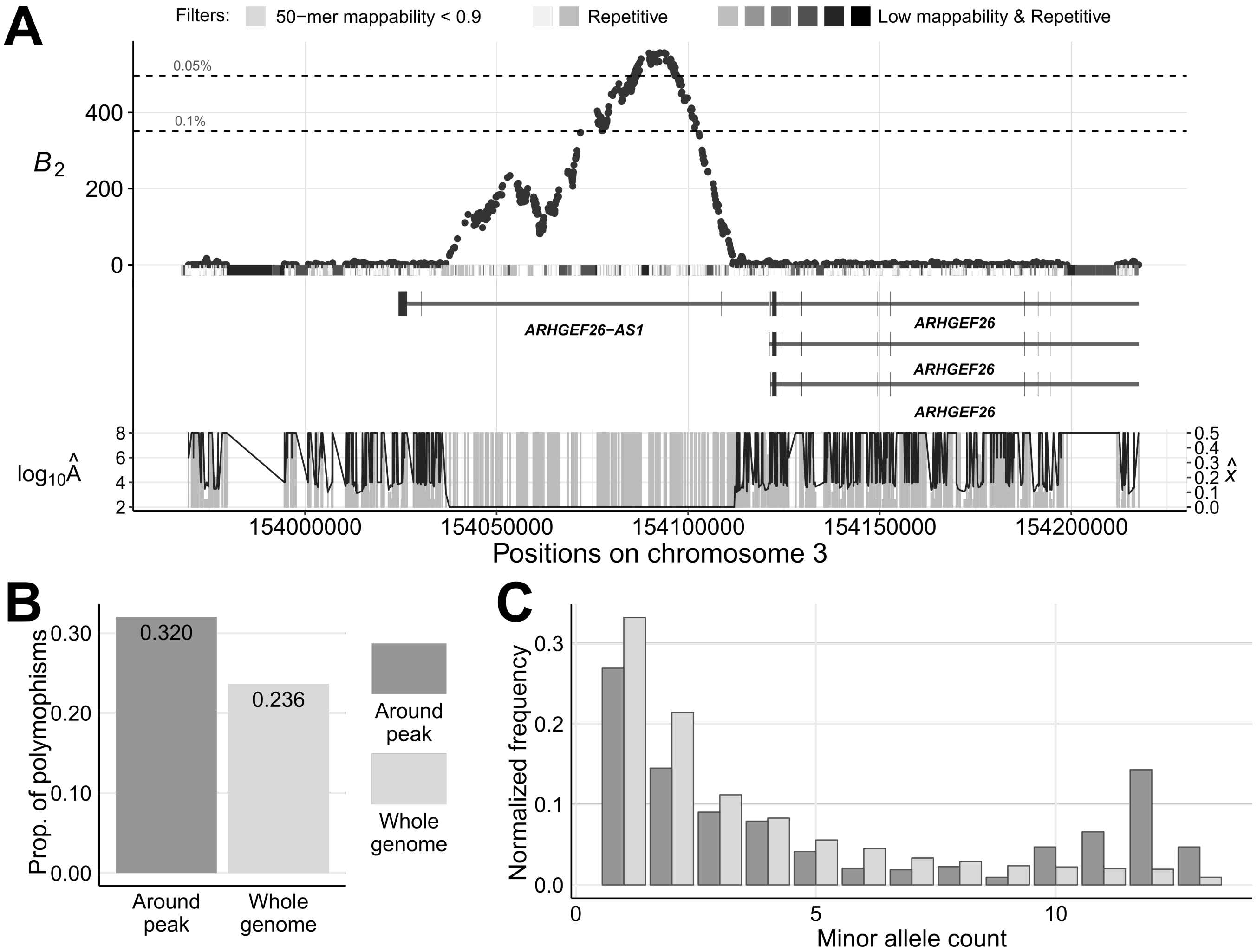

Figure S44: Evidence of balancing selection on *ARHGEF26*. (A) B_2 scores across the 250 kb genomic region on chromosome 3 centered on the peak. The gray bars directly under the B_2 scores represent the masked regions, as well as the features in these regions. The darker the shade, the greater number of types of repetitive sequences (*e.g.*, RepeatMasker mask, segmental duplication, simple repeats, or interrupted repeats) overlapping the region. Vertical gray bars below display the estimated equilibrium minor allele frequency $\widehat{x}$ for each maximum likelihood ratio B_2, and the black line traces the value for the respective inferred footprint size $\log_{10}(\widehat{A})$. (B) Proportion of informative sites that are polymorphic in the 250 kb region centered on the peak compared with the whole-genome average. (C) Minor allele frequency distribution in the 250 kb region centered on the peak compared with the whole-genome average.

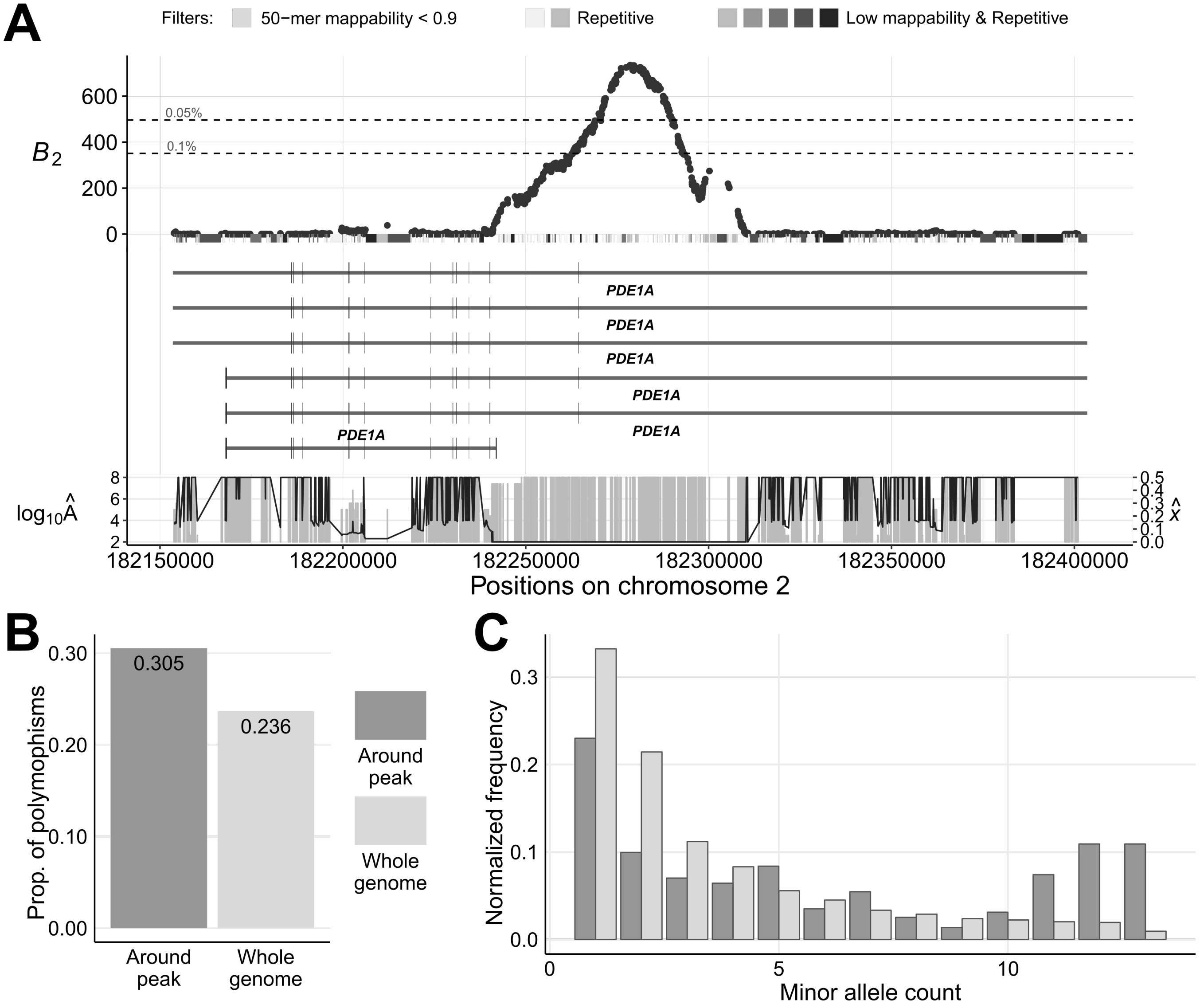

Figure S45: Evidence of balancing selection on *PDE1A*. (A) B_2 scores across the 250 kb genomic region on chromosome 2 centered on the peak. The gray bars directly under the B_2 scores represent the masked regions, as well as the features in these regions. The darker the shade, the greater number of types of repetitive sequences (*e.g.*, RepeatMasker mask, segmental duplication, simple repeats, or interrupted repeats) overlapping the region. Vertical gray bars below display the estimated equilibrium minor allele frequency $\widehat{x}$ for each maximum likelihood ratio B_2, and the black line traces the value for the respective inferred footprint size $\log_{10}(\widehat{A})$. (B) Proportion of informative sites that are polymorphic in the 500 kb region centered on the peak compared with the whole-genome average. (C) Minor allele frequency distribution in the 250 kb region centered on the peak compared with the whole-genome average.

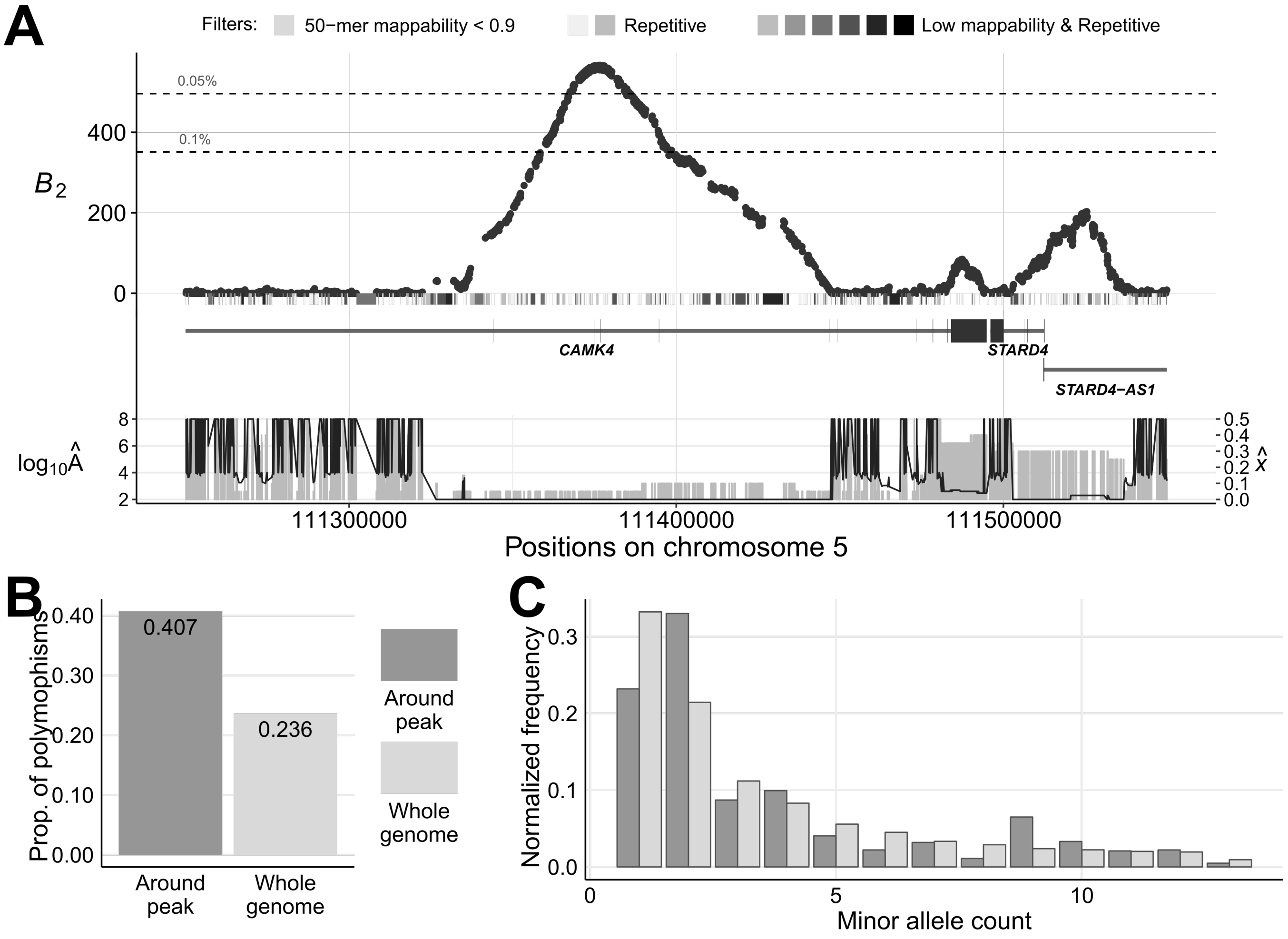

Figure S46: Evidence of balancing selection on *CAMK4*. (A) B_2 scores across the 250 kb genomic region on chromosome 5 centered on the peak. The gray bars directly under the B_2 scores represent the masked regions, as well as the features in these regions. Vertical gray bars below display the estimated equilibrium minor allele frequency $\widehat{x}$ for each maximum likelihood ratio B_2, and the black line traces the value for the respective inferred $\log_{10}(\widehat{A})$. (B) Proportion of informative sites that are polymorphic in the 250 kb region centered on the peak compared with the whole-genome average. (C) Minor allele frequency distribution in the 250 kb region centered on the peak compared with the whole-genome average.

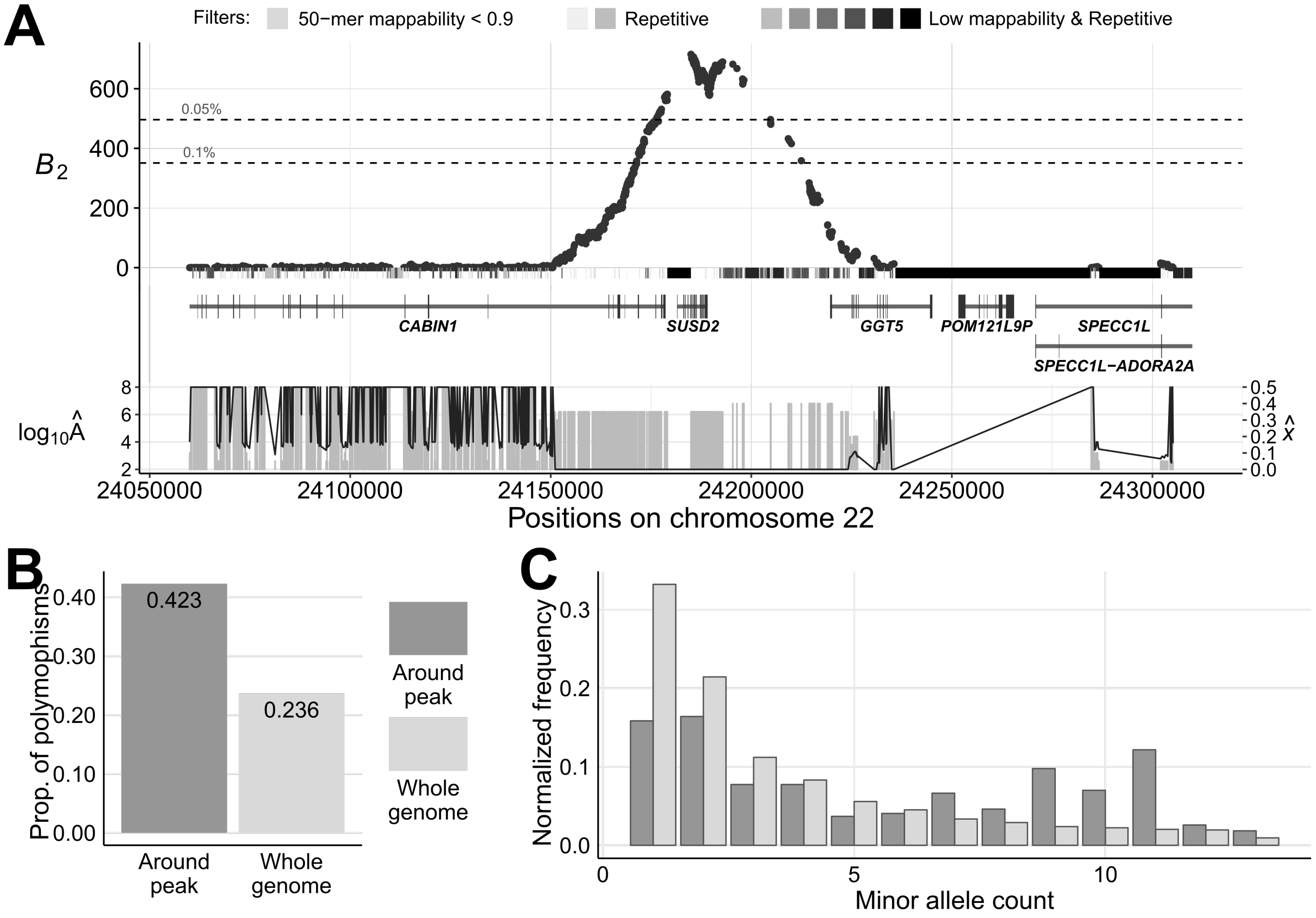

Figure S47: Evidence of balancing selection on *SUSD2*. (A) B_2 scores across the 250 kb genomic region on chromosome 22 centered on the peak. The gray bars directly under the B_2 scores represent the masked regions, as well as the features in these regions. The darker the shade, the greater number of types of repetitive sequences (*e.g.*, RepeatMasker mask, segmental duplication, simple repeats, or interrupted repeats) overlapping the region. Vertical gray bars below display the estimated equilibrium minor allele frequency $\widehat{x}$ for each maximum likelihood ratio B_2, and the black line traces the value for the respective inferred footprint size $\log_{10}(\widehat{A})$. (B) Proportion of informative sites that are polymorphic in the 250 kb region centered on the peak compared with the whole-genome average. (C) Minor allele frequency distribution in the 250 kb region centered on the peak compared with the whole-genome average.

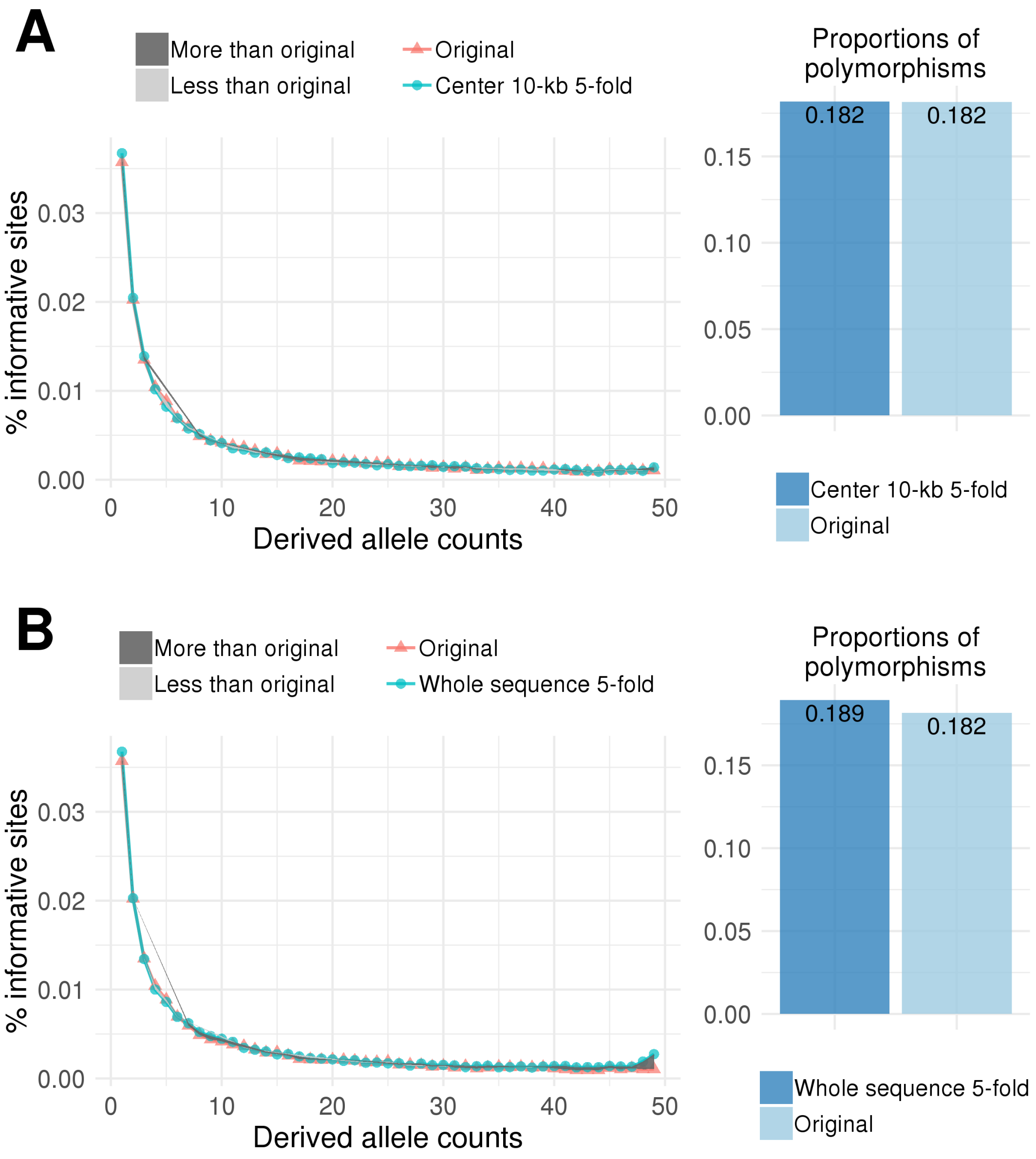

Figure S48: Derived site frequency spectra and proportions of polymorphic sites for (A) sequences with a central 10 kb mutational hotspot of rate 5μ, and for (B) sequences with an elevated mutation rate of 5μ across the entire sequence, each compared to sequences with the original mutation rate μ.

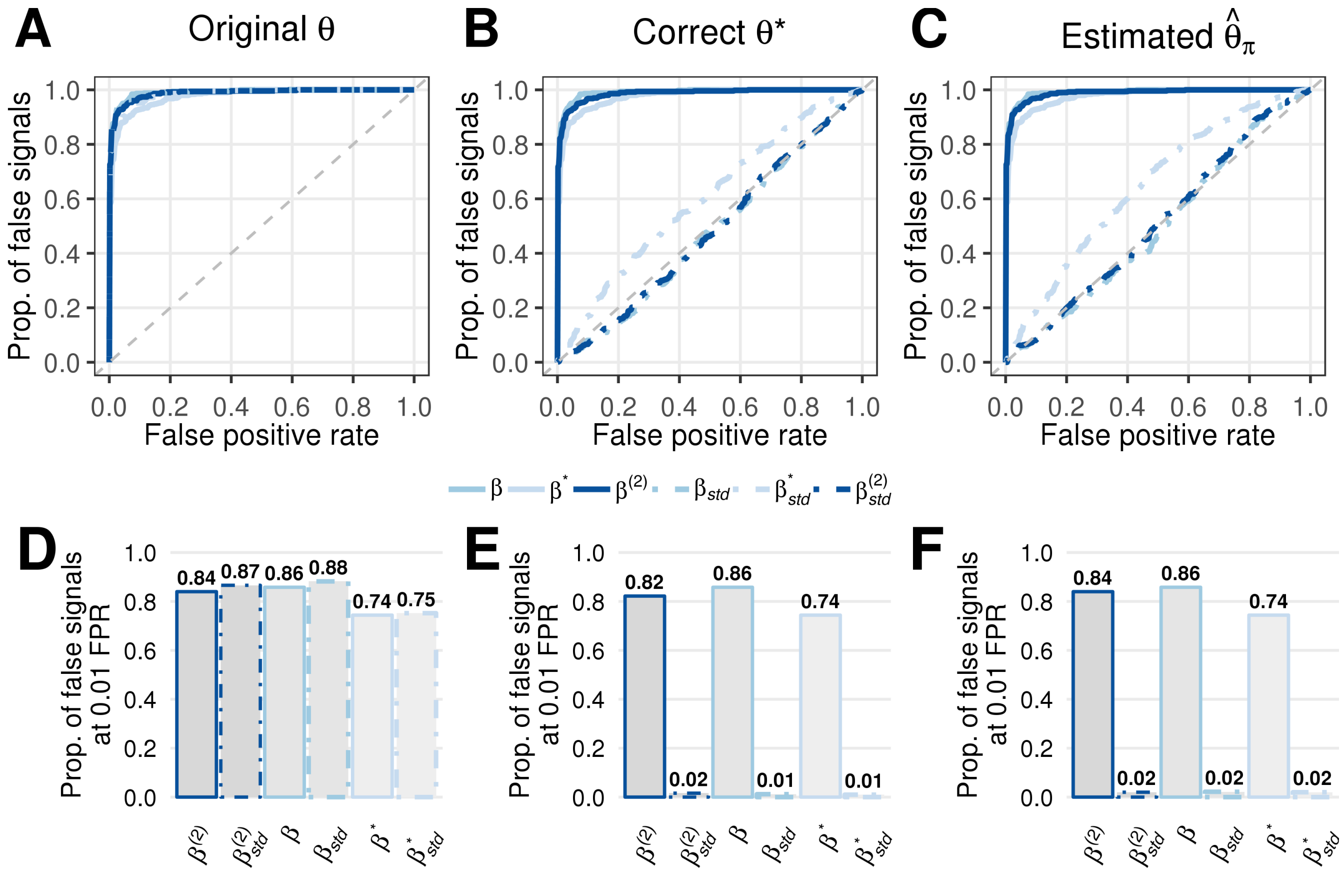

Figure S49: Robustness of β statistics on sequences with five-fold mutation rate when provided with different θ parameters. (A-C) Proportions of false signals reported by each β statistic variant as a function of the false positive rate (FPR), and (D-F) proportions of false signals at a 1% FPR when the population-scaled mutation rate $\theta = 4N\mu$ provided to the β statistics is (A, D) the original un-elevated rate θ, (B, E) the true mutation rate $\theta^\star = 5\theta$, or (C, F) the mean pairwise sequence difference $\widehat{\theta}_\pi$ estimated from the given simulated replicate. In all panels, the false positive rate was based on neutral simulations with the original mutation rate $\mu = 2.5 \times 10^{-8}$ per site per generation, and the proportion of false signals was based on neutral simulations with the elevated mutation rate of 5μ. We define a false signal as a simulated replicate that has a higher score than the score associated with a given false positive rate cutoff.

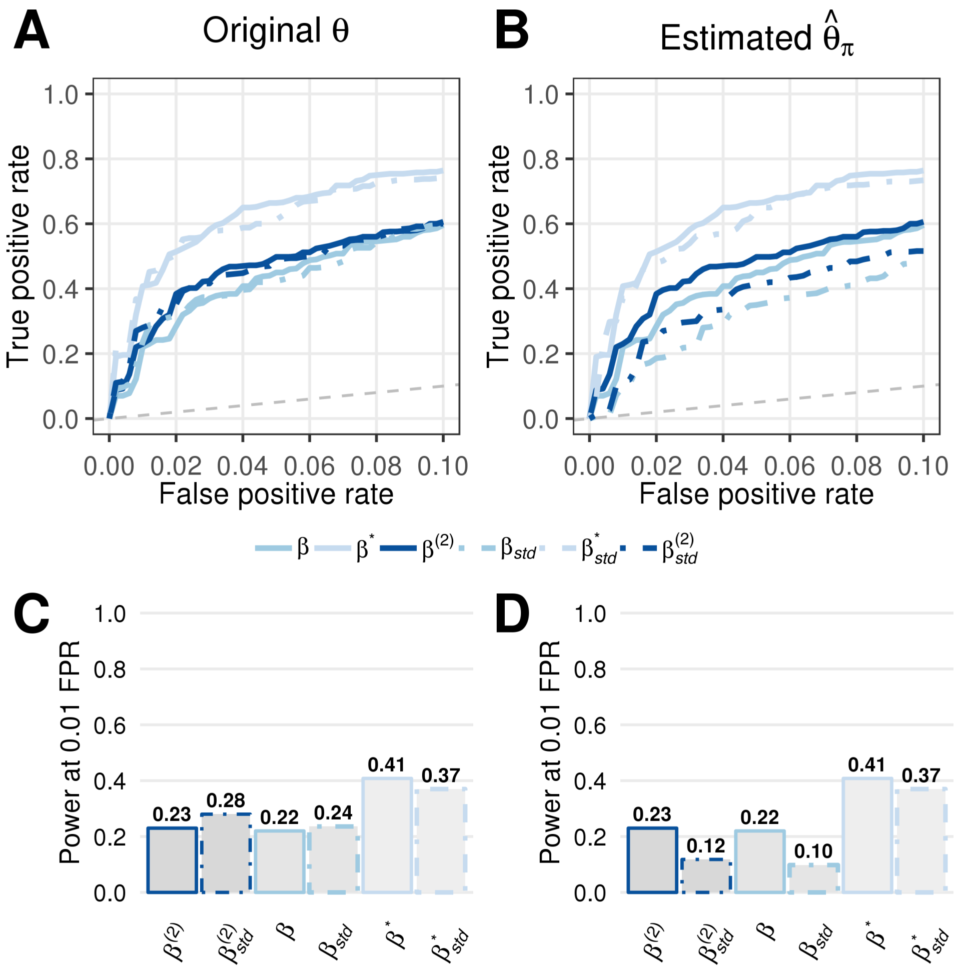

Figure S50: Power of β statistics to detect balancing selection that lasted 2.5×10^5 generations when the population-scaled mutation rate θ provided to the β statistics is either (A, C) the correct rate $\theta = 4N\mu$ or (B, D) the mean pairwise sequence difference $\widehat{\theta}_\pi$ estimated from the given simulated replicate. (A, B) Receiver operating characteristic curves for each β statistic when the false positive rate (FPR) is below 10%. (C, D) True positive rate (power) at a 1% FPR for each β statistic. Dashed lines represent standardized β statistics, and solid lines represent unstandardized β statistics.

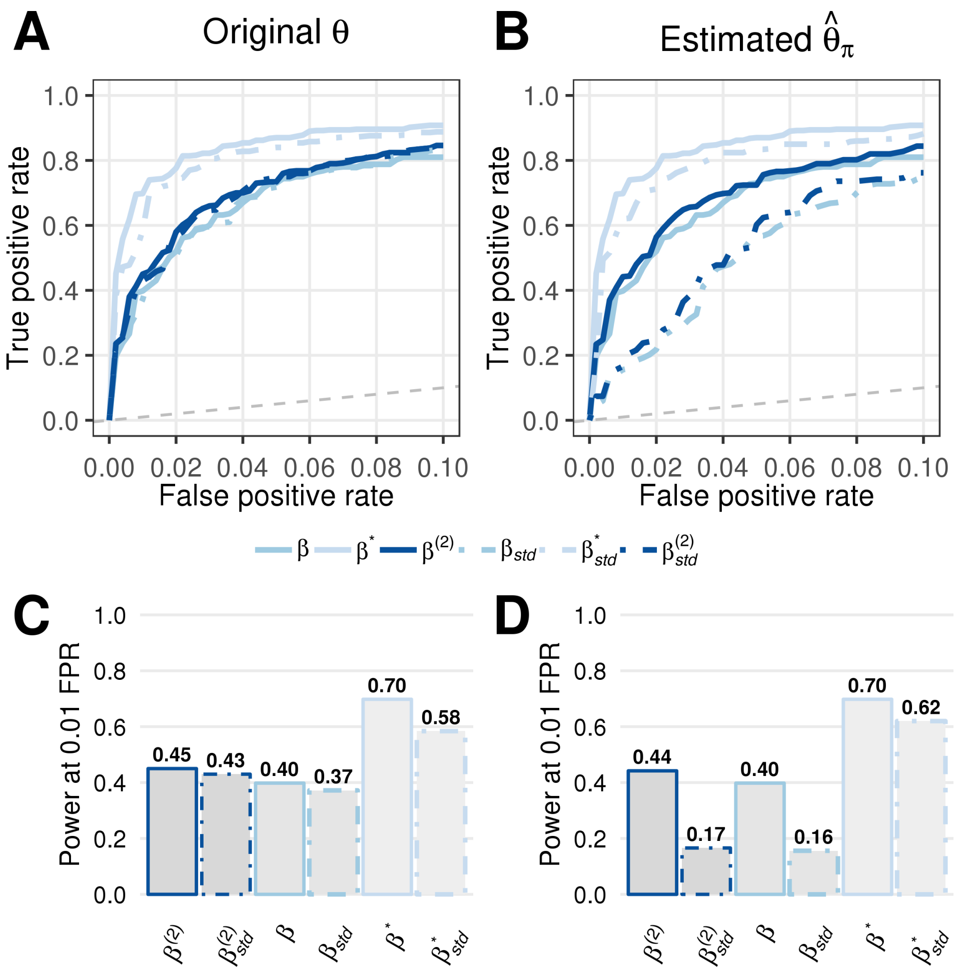

Figure S51: Power of β statistics to detect balancing selection that lasted 2.5×10^5 generations on sequences with an elevated mutation rate of 5μ, when the population-scaled mutation rate θ provided to the β statistics is either (A, C) the correct rate $\theta = 4N \cdot 5\mu$ or (B, D) the mean pairwise sequence difference $\widehat{\theta}_\pi$ estimated from the given simulated replicate. (A, B) Receiver operating characteristic curves for each β statistic when the false positive rate (FPR) is below 10%. (C, D) True positive rate (power) at a 1% FPR for each β statistic. Dashed lines represent standardized β statistics, and solid lines represent unstandardized β statistics.

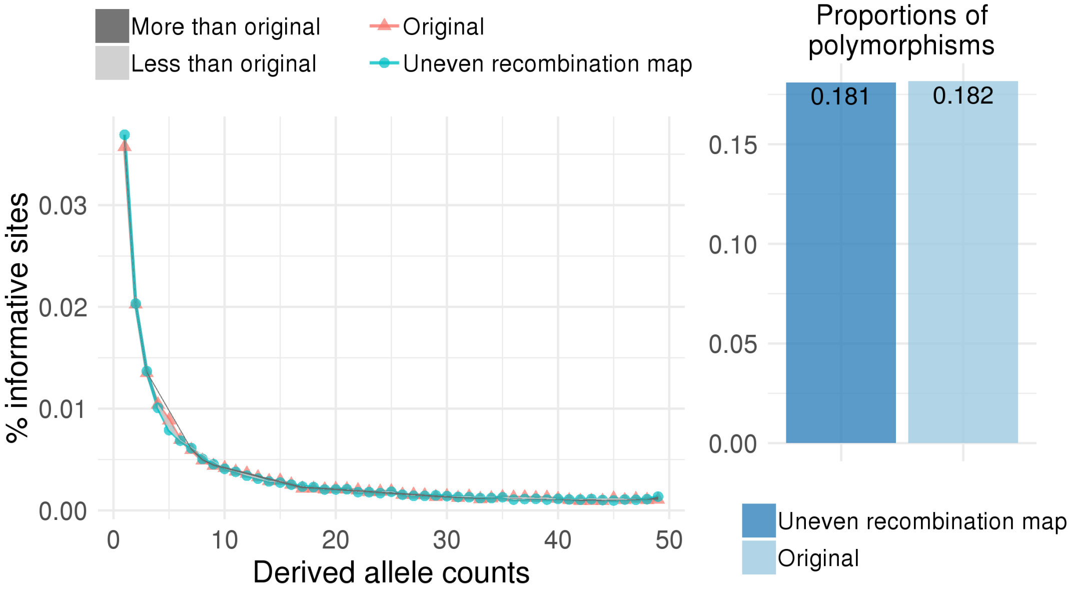

Figure S52: Derived allele frequency spectra and proportions of polymorphic sites for sequences evolving with an uneven recombination map relative to those evolving with a uniform recombination map.

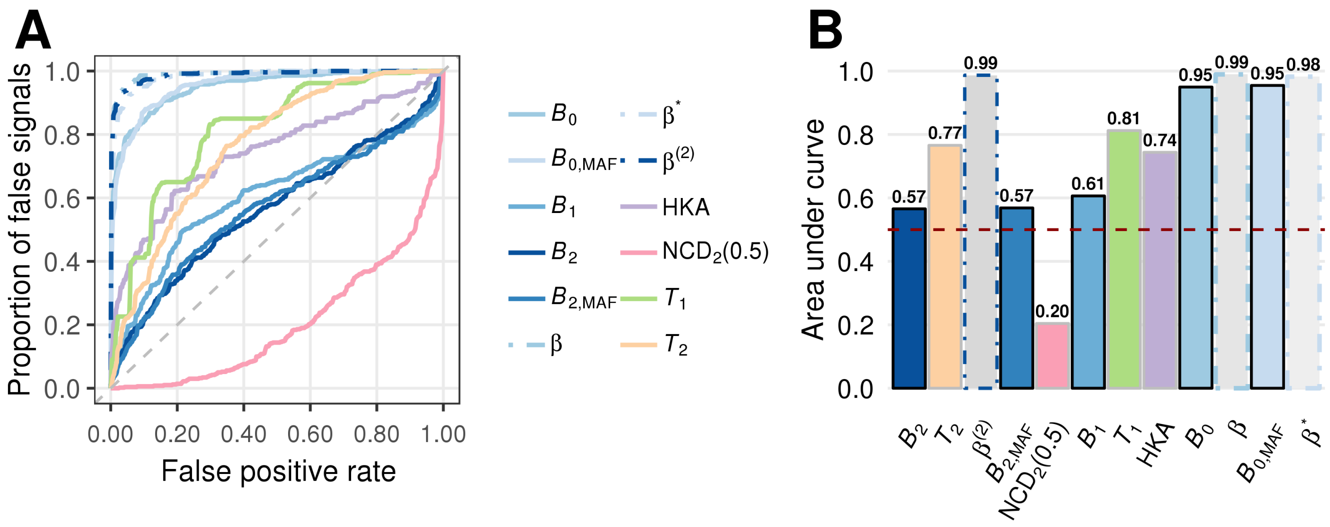

Figure S53: Robustness of B statistics evaluated when the mutation rate is elevated five-fold across the entire genomic region, with B_1, B_2, and $B_{2,\mathrm{MAF}}$ variants adopting identical numbers of informative sites as T_1 and T_2 for each test, and B_0 and $B_{0,\mathrm{MAF}}$ variants adopting identical length-based windows as β statistics. (A) Proportions of false signals as a function of false positive rates (FPR) for each statistic. (B) Area under curve for each statistic. The false positive rate was based on neutral simulations with the original mutation rate $\mu = 2.5 \times 10^{-8}$ per site per generation, and the proportion of false signals was based on neutral simulations with the elevated mutation rate of 5μ. We define a false signal as a simulated replicate that has a higher score than the score associated with a given false positive rate cutoff.

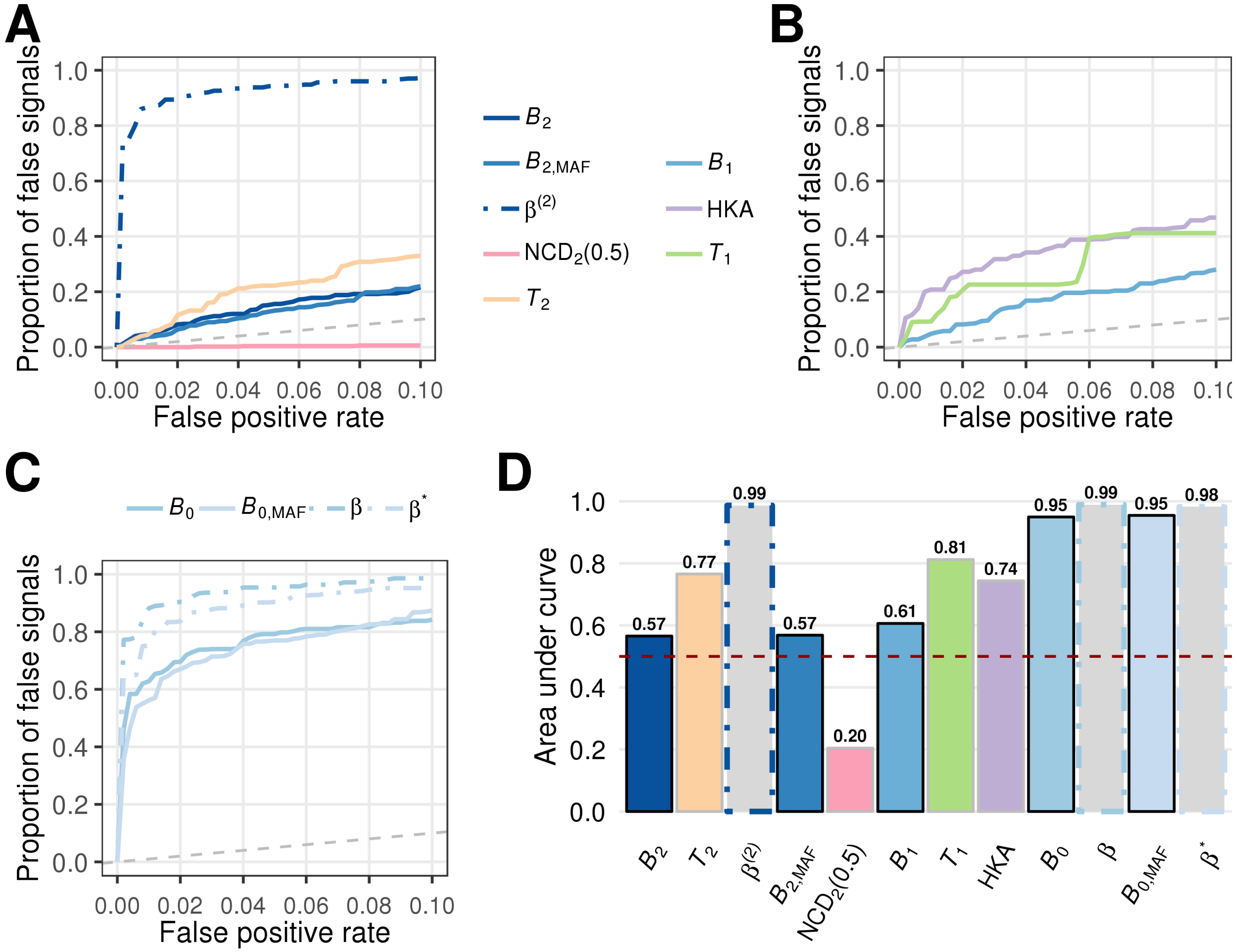

Figure S54: Split view for performance of three groups of analogous statistics on neutral sequences with an elevated mutation rate of 5μ, with B_1, B_2, and $B_{2,\mathrm{MAF}}$ variants adopting identical numbers of informative sites as T_1 and T_2 for each test, and B_0 and $B_{0,\mathrm{MAF}}$ adopting identical length-based windows as β statistics. (A-C) Proportions of false signals as a function of false positive rate (FPR) for (A) statistics that use both substitutions and allele frequencies, namely B_2, T_2, $B_{2,\mathrm{MAF}}$, $\beta^{(2)}$, and NCD, (B) statistics that only use substitution and polymorphism calls, *i.e.*, B_1, T_1, and HKA, and (C) statistics that only use allele frequencies, which are B_0, β, $B_{0,\mathrm{MAF}}$, and β^*. (D) Areas under curve for all statistics. Bars for all B statistics are bordered by black lines, and all β statistics are filled in gray. In all panels, the false positive rate was based on neutral simulations with the original mutation rate $\mu = 2.5 \times 10^{-8}$ per site per generation, and the proportion of false signals was based on neutral simulations with the elevated mutation rate of 5μ. We define a false signal as a simulated replicate that has a higher score than the score associated with a given false positive rate cutoff.

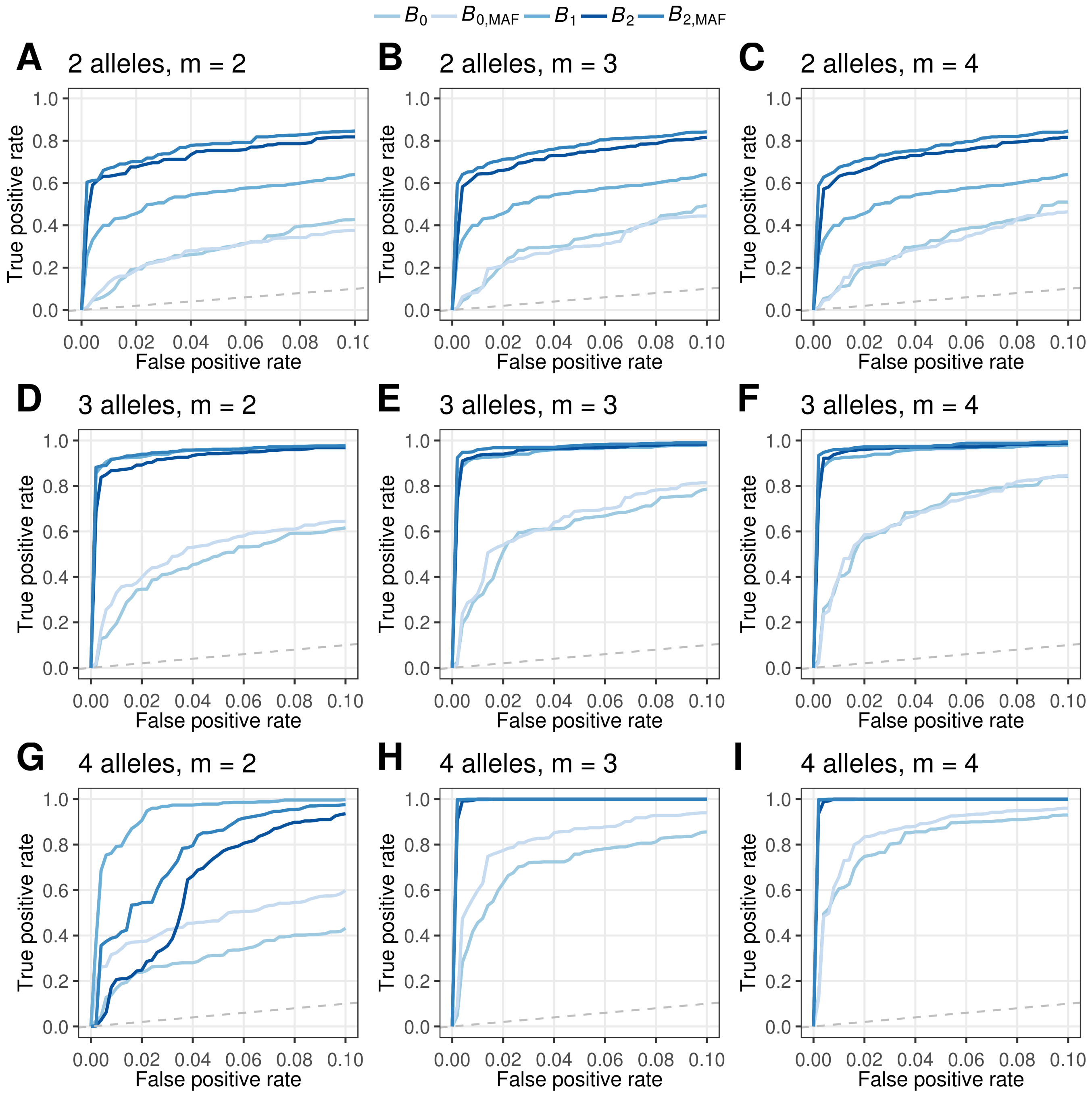

Figure S55: Receiver operating characteristic curves for multi-allelic B statistics when adopting different m, the presumed number of balanced alleles. Mutations with selection coefficients $s = 0.001$ with $h = 20$ were introduced 500,000 generations before sampling, with (A-C) two, (D-F) three, or (G-I) four distinct alleles balanced in the population.

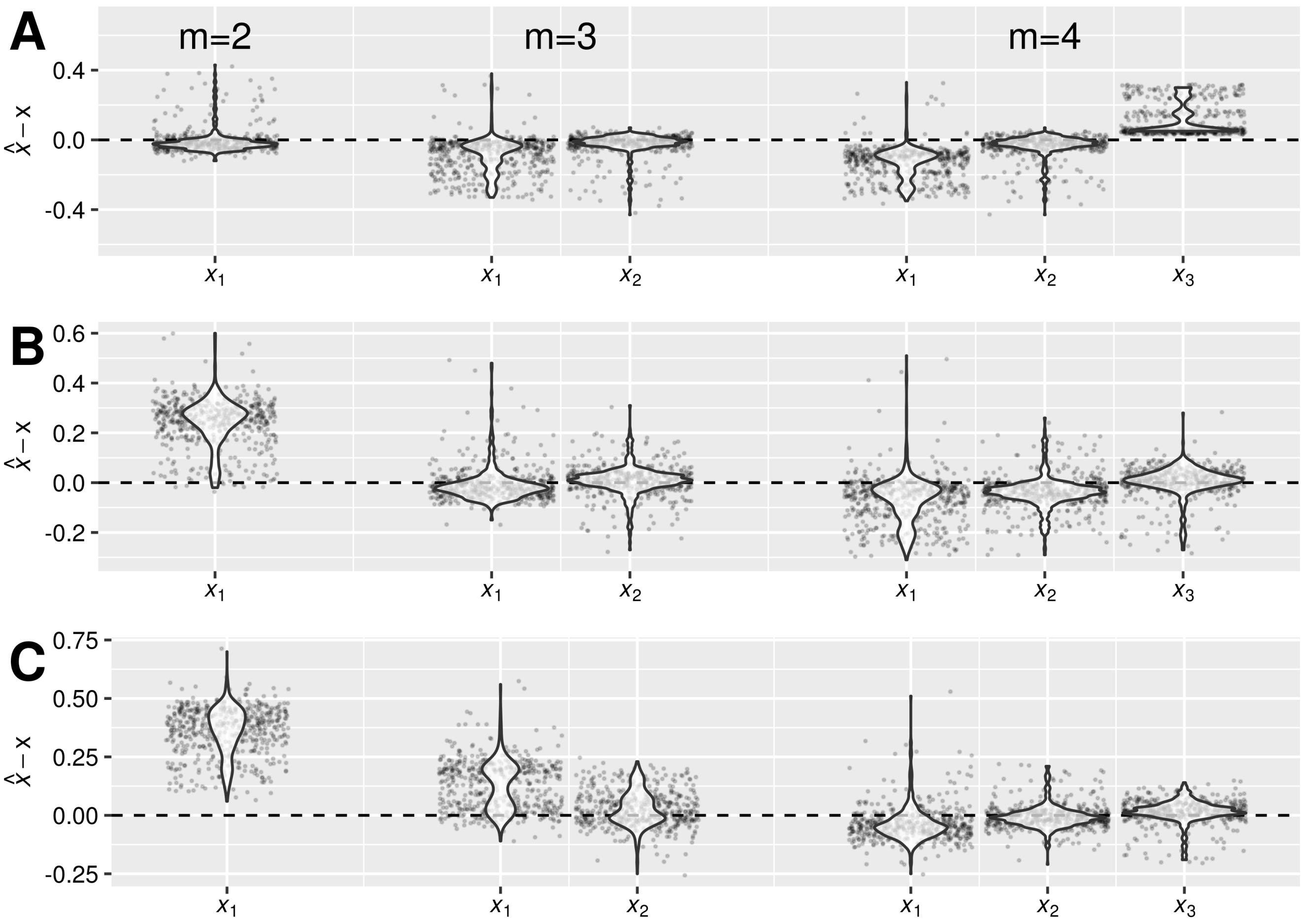

Figure S56: Differences between the optimal equilibrium frequencies ($\widehat{x}$) and true allele frequencies of the site under selection reported by B_2 statistic when the number of balanced alleles m is assumed to be two, three, or four. Panels represent simulations with (A) two, (B) three, or (C) four distinct alleles balancing at the selected locus.

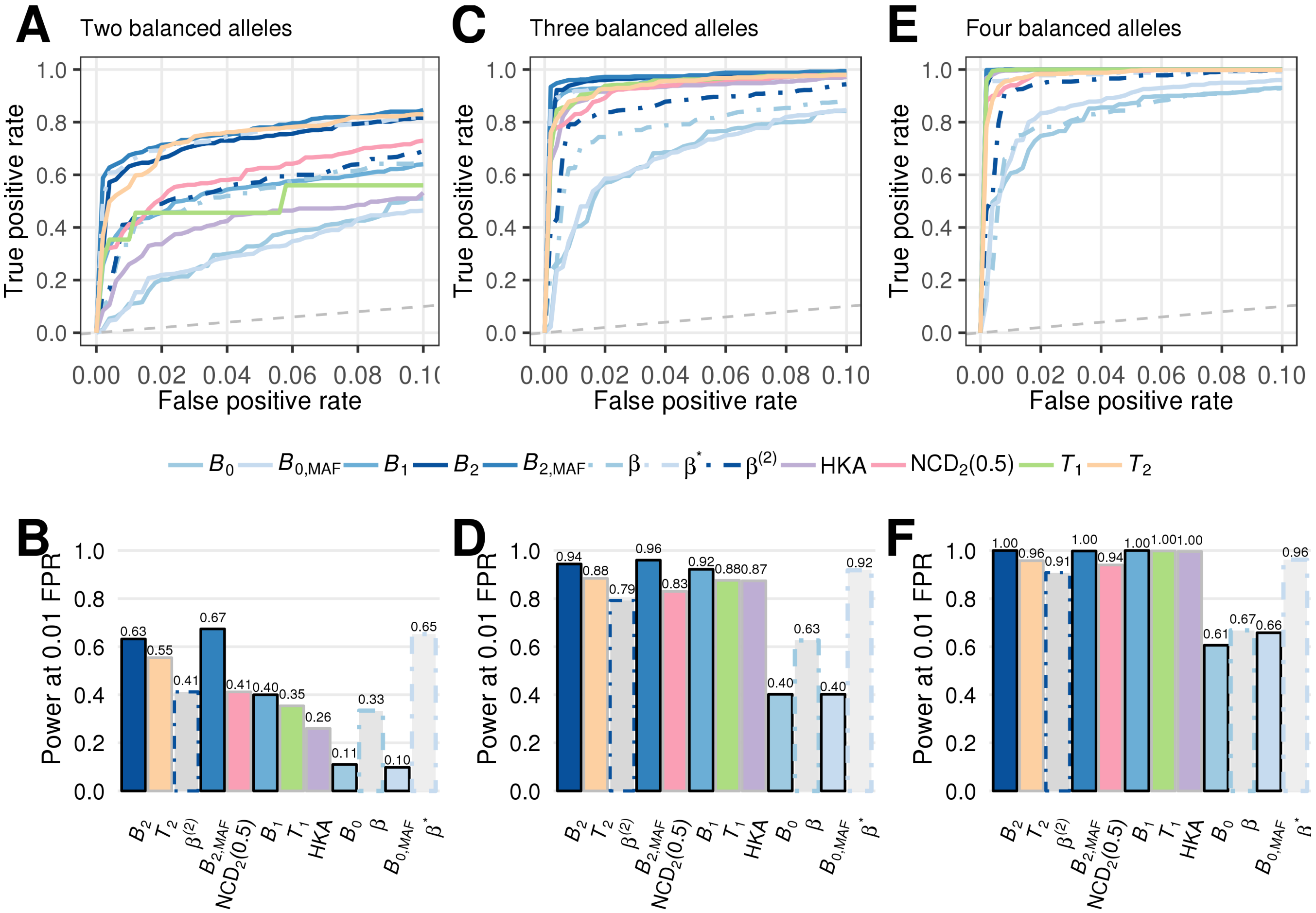

Figure S57: Powers of each statistic to discover genomic regions carrying (A, B) two, (C, D) three, or (E, F) four distinct balanced alleles with $s = 0.001$ and $h = 20$. The B statistics assume $m = 4$. (A, C, E) Receiver operating characteristic curves for each statistic under each scenario. (B, D, F) Powers at a 1% false positive rate (FPR) for each statistic under each of the three scenarios.

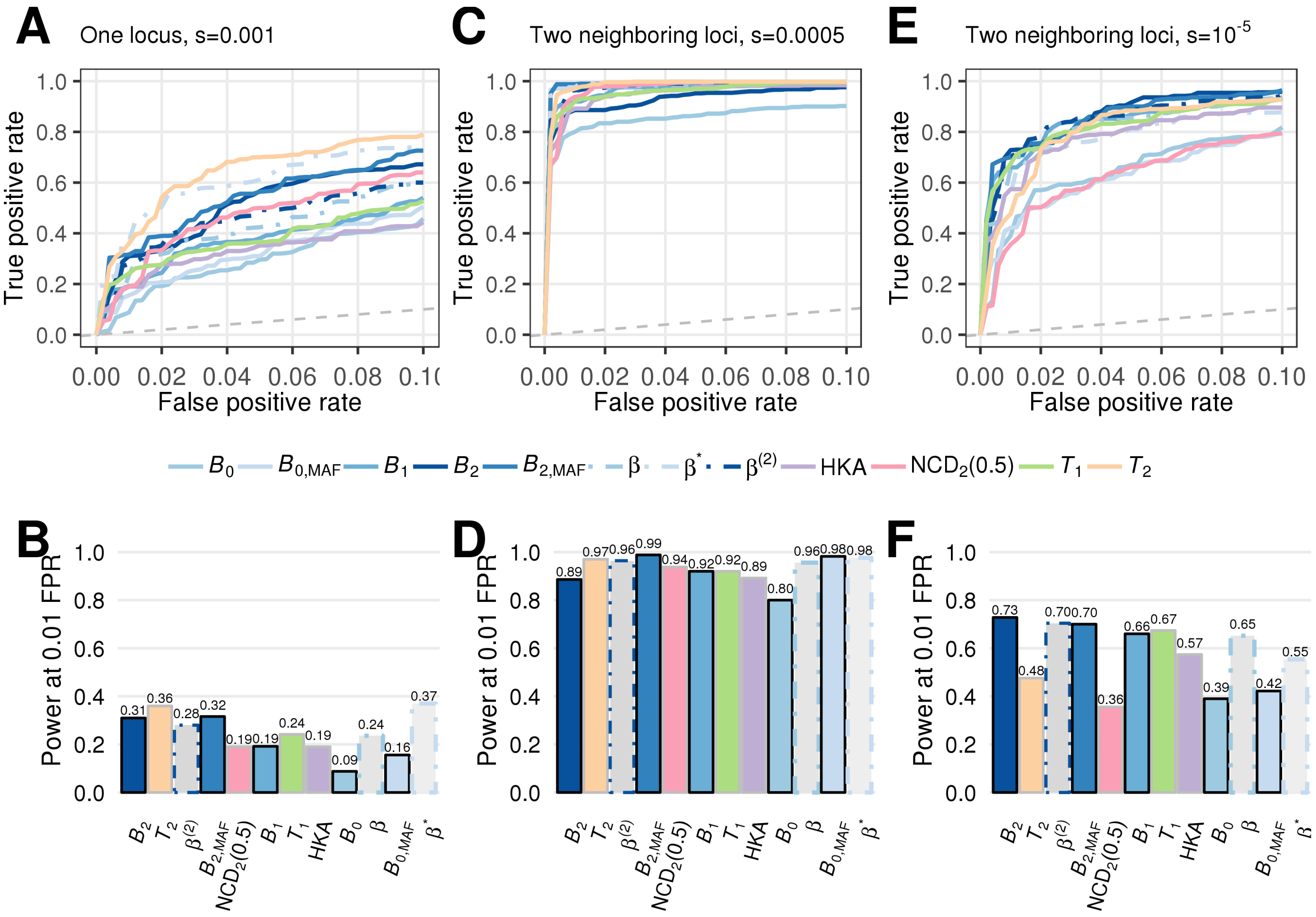

Figure S58: Powers of each statistic to discover genomic regions carrying balanced alleles at (A, B) one locus with $s = 0.001$ and $h = 20$, at (C, D) two neighboring loci 10 kb apart with $s = 0.0005$ and $h = 20$, and at (E, F) two loci 10 kb apart with $s = 10^{-5}$ and $h = 20$. (A, C, E) Receiver operating characteristic curves for each statistic under each scenario. (B, D, F) Powers at a 1% false positive rate (FPR) for each statistic under each of the three scenarios.

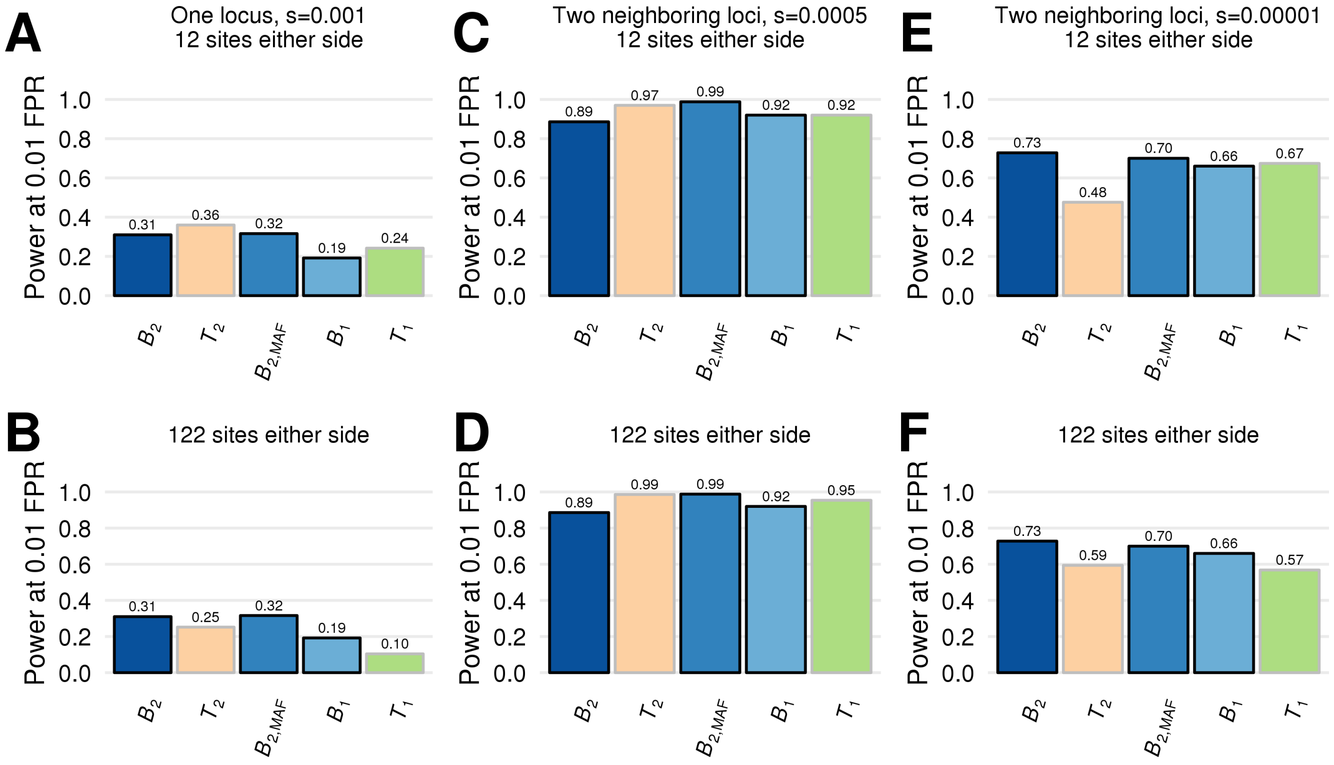

Figure S59: Power at a 1% false positive rate (FPR) of T statistics and their analogous B statistics to discover genomic regions carrying balanced alleles at (A, B) one locus with $s = 0.001$ and $h = 20$, at (C, D) two neighboring loci 10 kb apart with $s = 0.0005$ and $h = 20$, and at (E, F) two loci 10 kb apart with $s = 10^{-5}$ and $h = 20$. B statistics, and T statistics consider (A, C, E) 12 informative sites or (B, D, F) informative sites on either side of each test site.
