# Supplementary figures and images for: Flexible Mixture Model Approaches That Accommodate Footprint Size Variability for Robust Detection of Balancing Selection
Source: Mol Biol Evol. 2020 Oct 4;37(11):3267–91. doi: 10.1093/molbev/msaa134 (PMC7820363; doi:10.1093/molbev/msaa134)

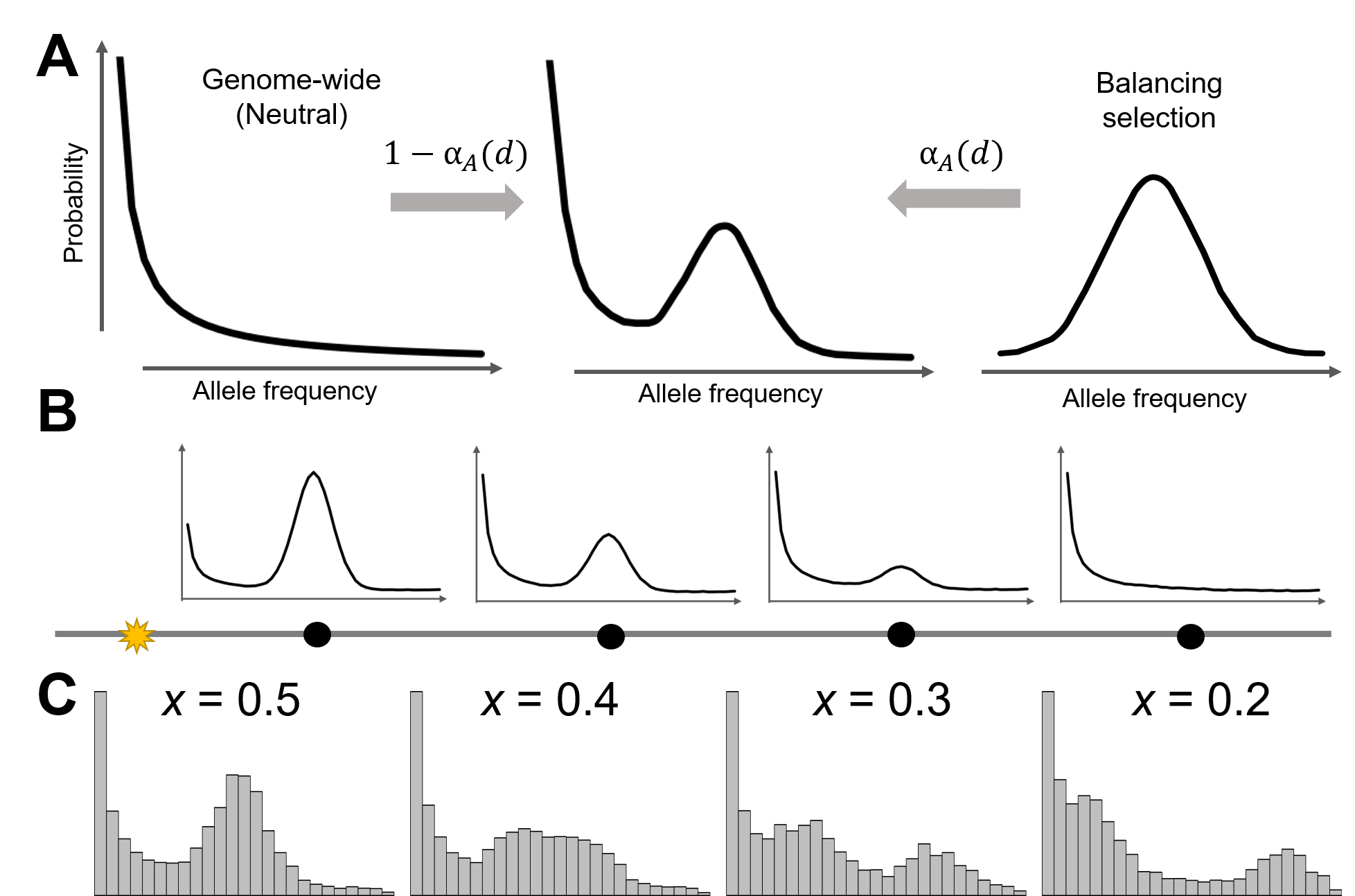

Supplement: msaa134_supplementary_data [file msaa134_supplementary_data.zip › BallerMix_final/figures/Fig1_schematic_3panel.png]

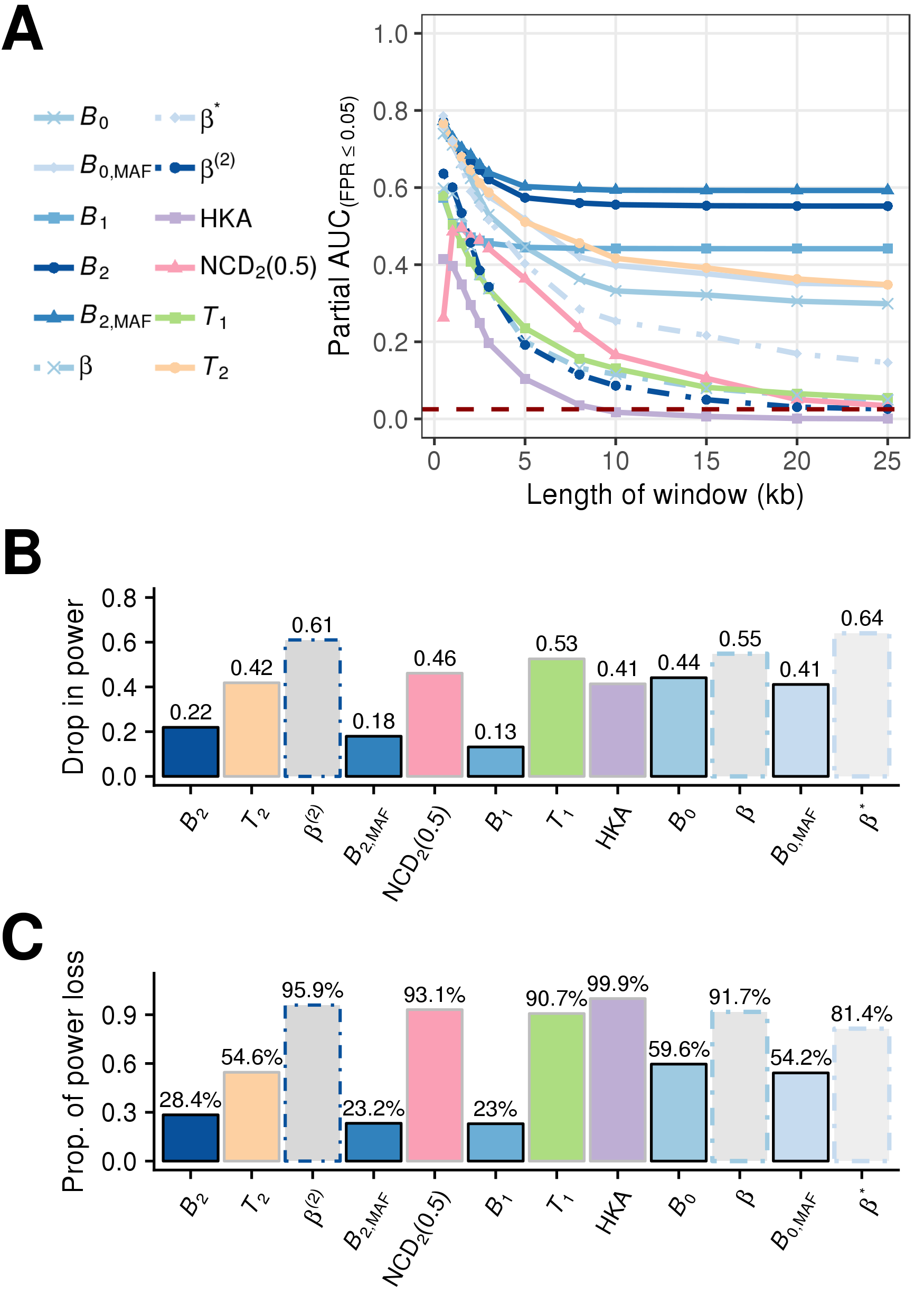

Supplement: msaa134_supplementary_data [file msaa134_supplementary_data.zip › BallerMix_final/figures/Fig2_PropLoss-05AUC_Bs_v_T12+stats_HCG_15MYA_s01_h20.png]

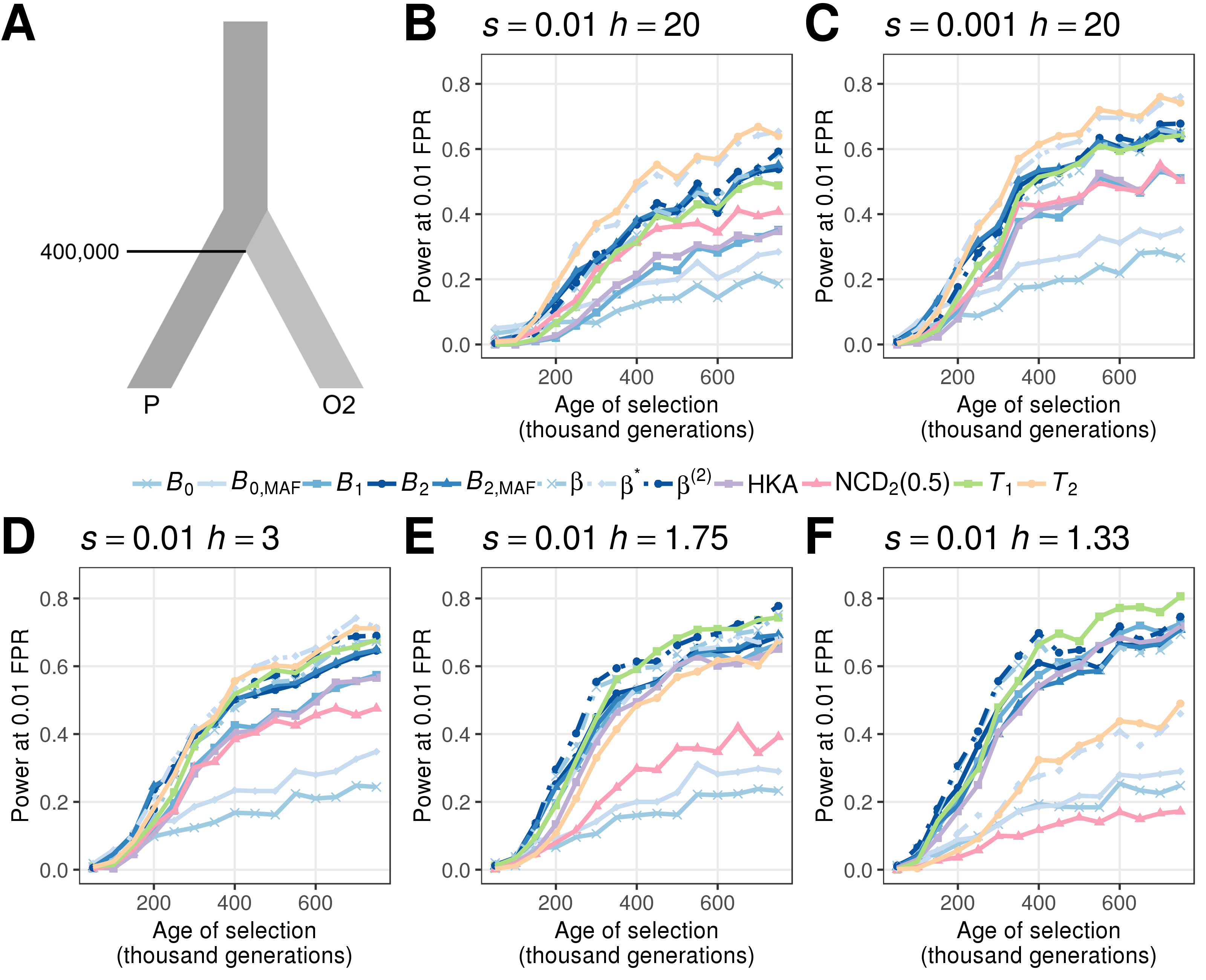

Supplement: msaa134_supplementary_data [file msaa134_supplementary_data.zip › BallerMix_final/figures/Fig3_Time-power_1-15mya_bimodalBs_v_T12+stats_6panel.png]

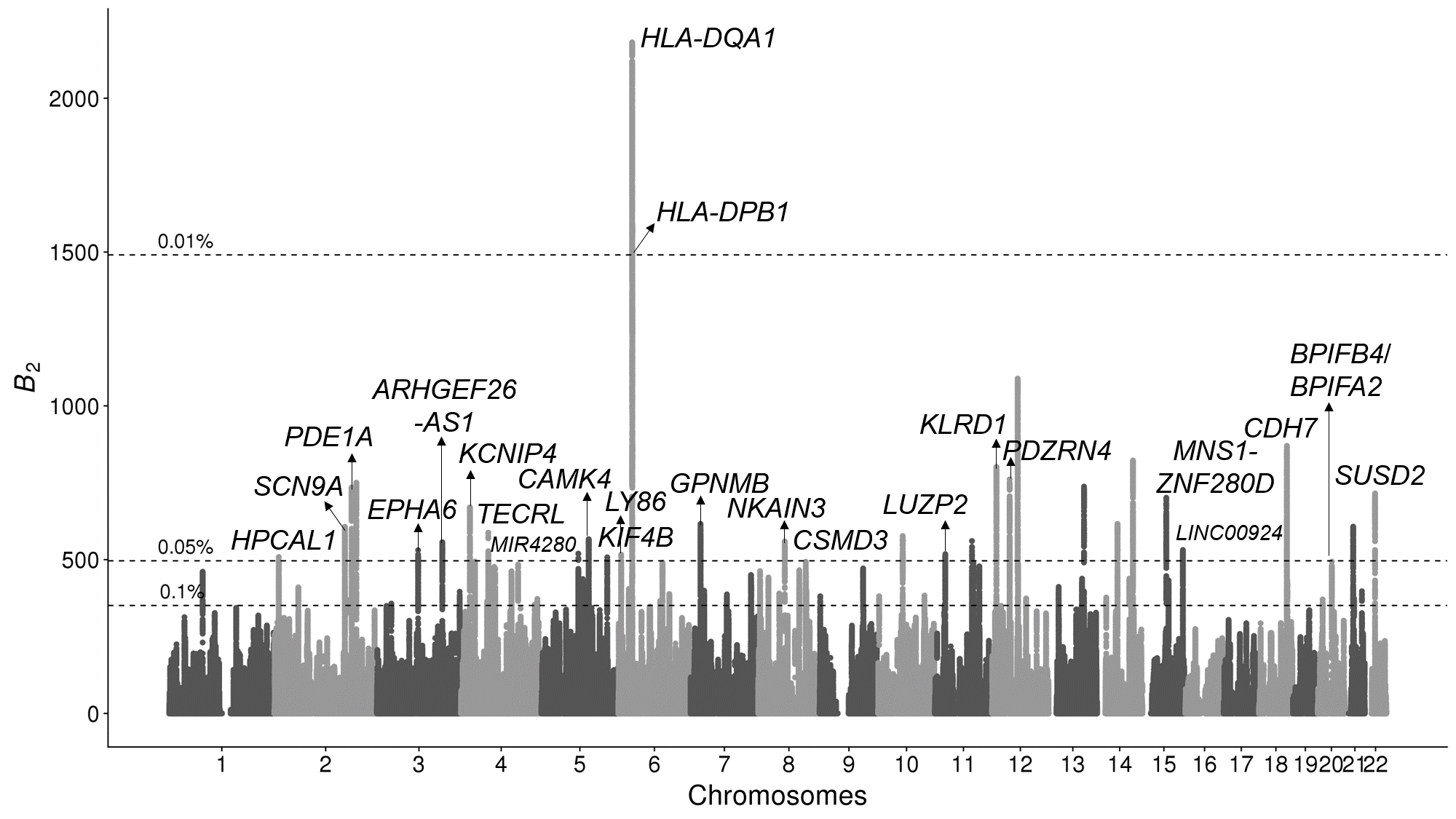

Supplement: msaa134_supplementary_data [file msaa134_supplementary_data.zip › BallerMix_final/figures/Fig4_anntManhattan_hg38_B2_all_WG_crg50m0-6filters_LR.png]

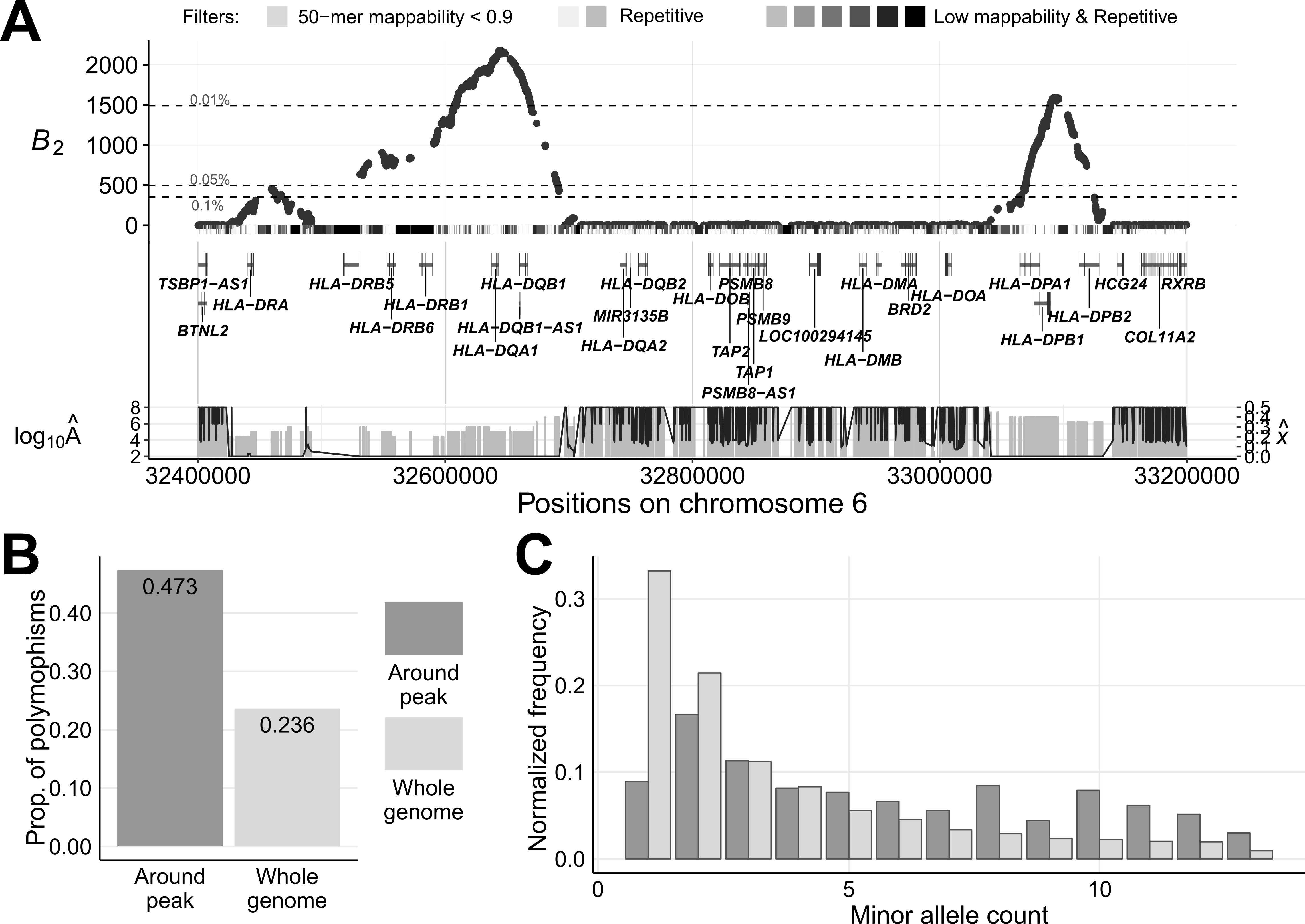

Supplement: msaa134_supplementary_data [file msaa134_supplementary_data.zip › BallerMix_final/figures/Fig5_hg38_B2_Chr6_HLA-D_800kb_LR-fancyGene-xA_spect.png]

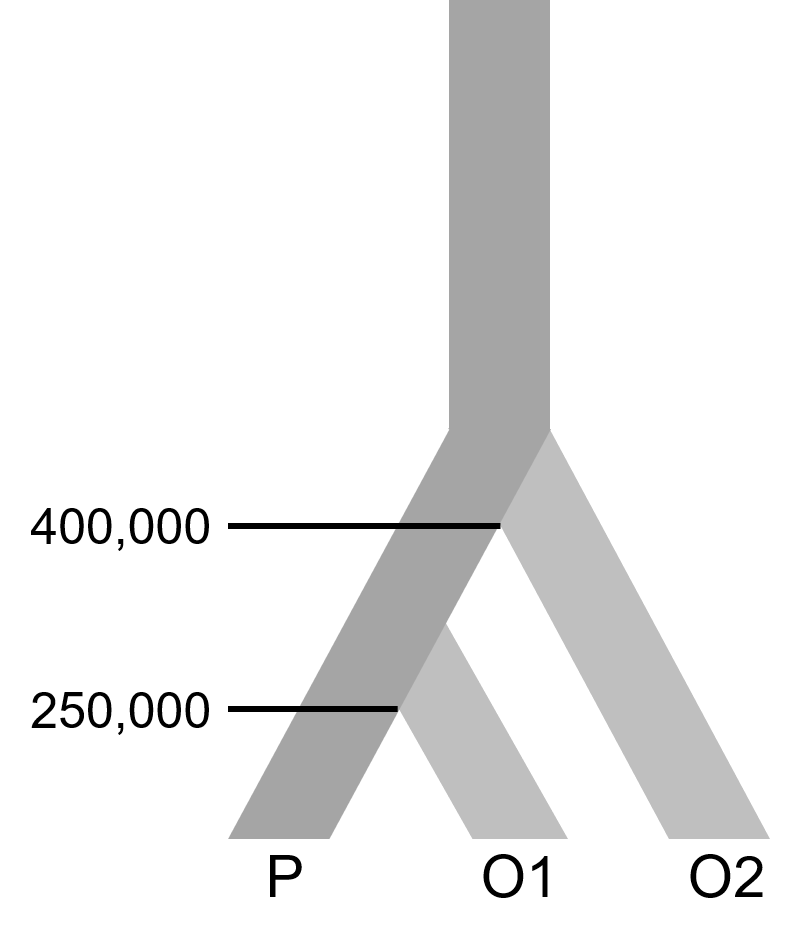

Supplement: msaa134_supplementary_data [file msaa134_supplementary_data.zip › BallerMix_final/figures/FigS1_HCG_tree.png]

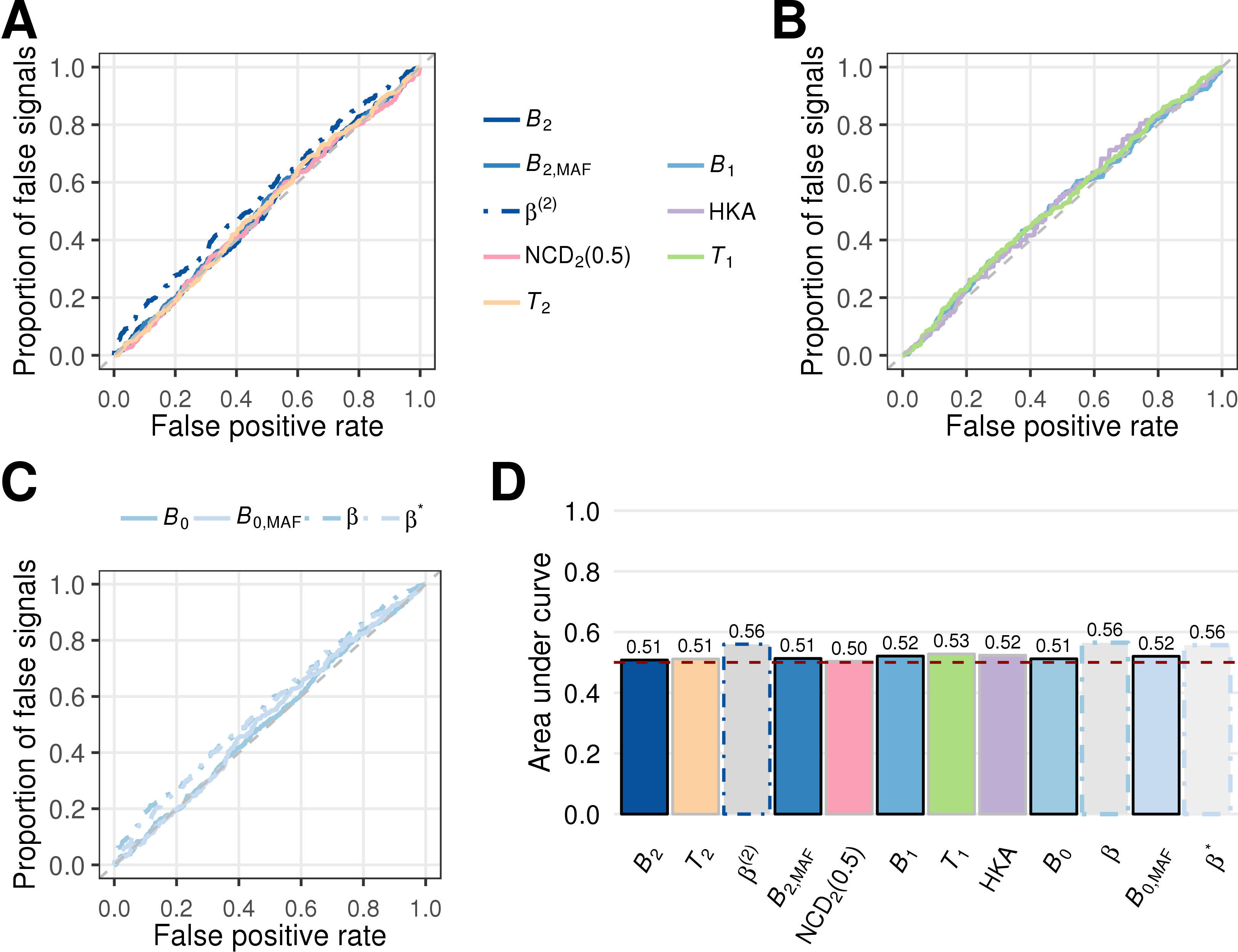

Supplement: msaa134_supplementary_data [file msaa134_supplementary_data.zip › BallerMix_final/figures/FigS10_splitView_HCG_localMut_ROC+powerbar_alphaB+stats.png]

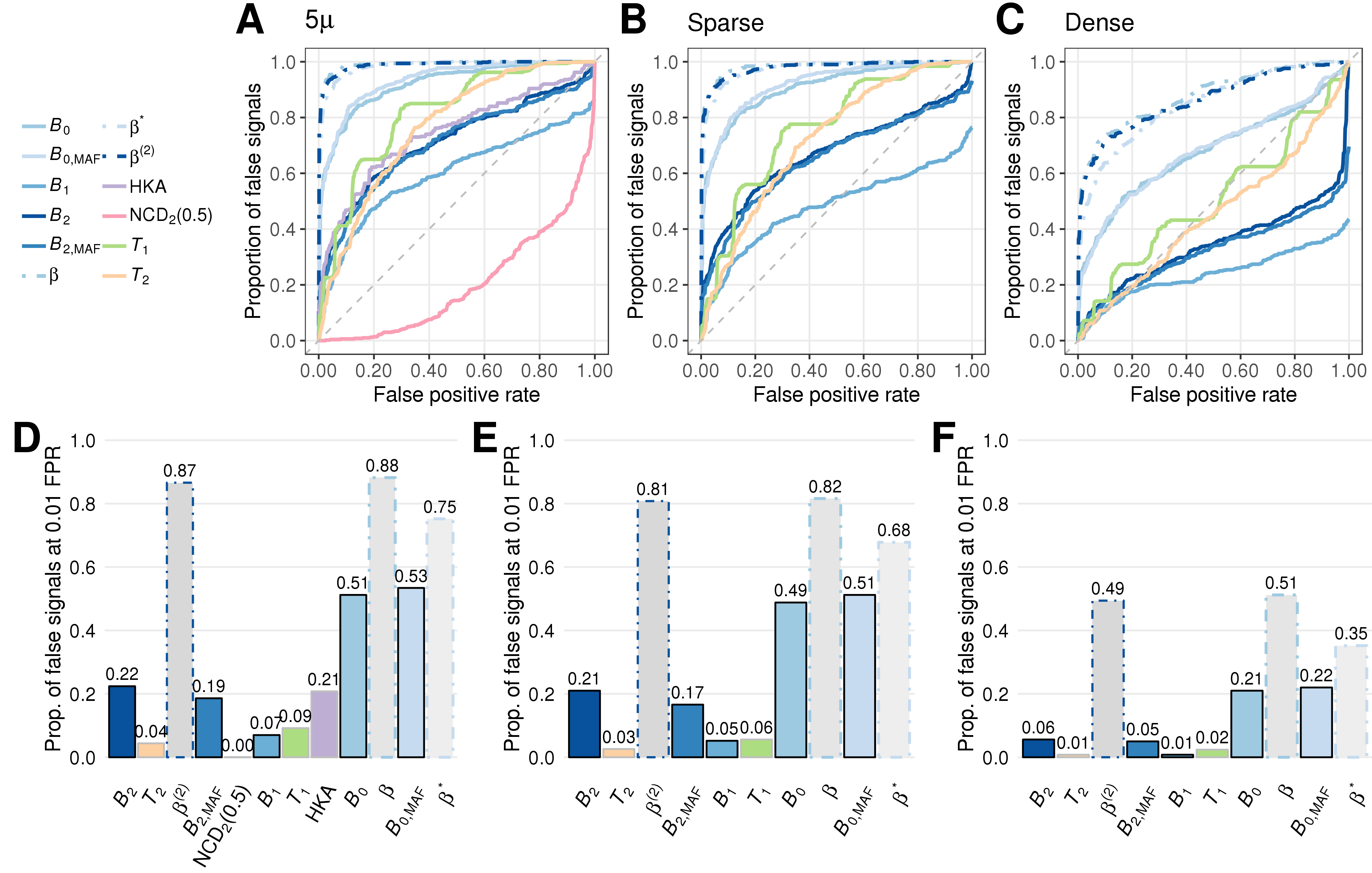

Supplement: msaa134_supplementary_data [file msaa134_supplementary_data.zip › BallerMix_final/figures/FigS11_newHCG_5Mut_ROC+bar_allStats_biBs-stdBetas_ori+DSs.png]

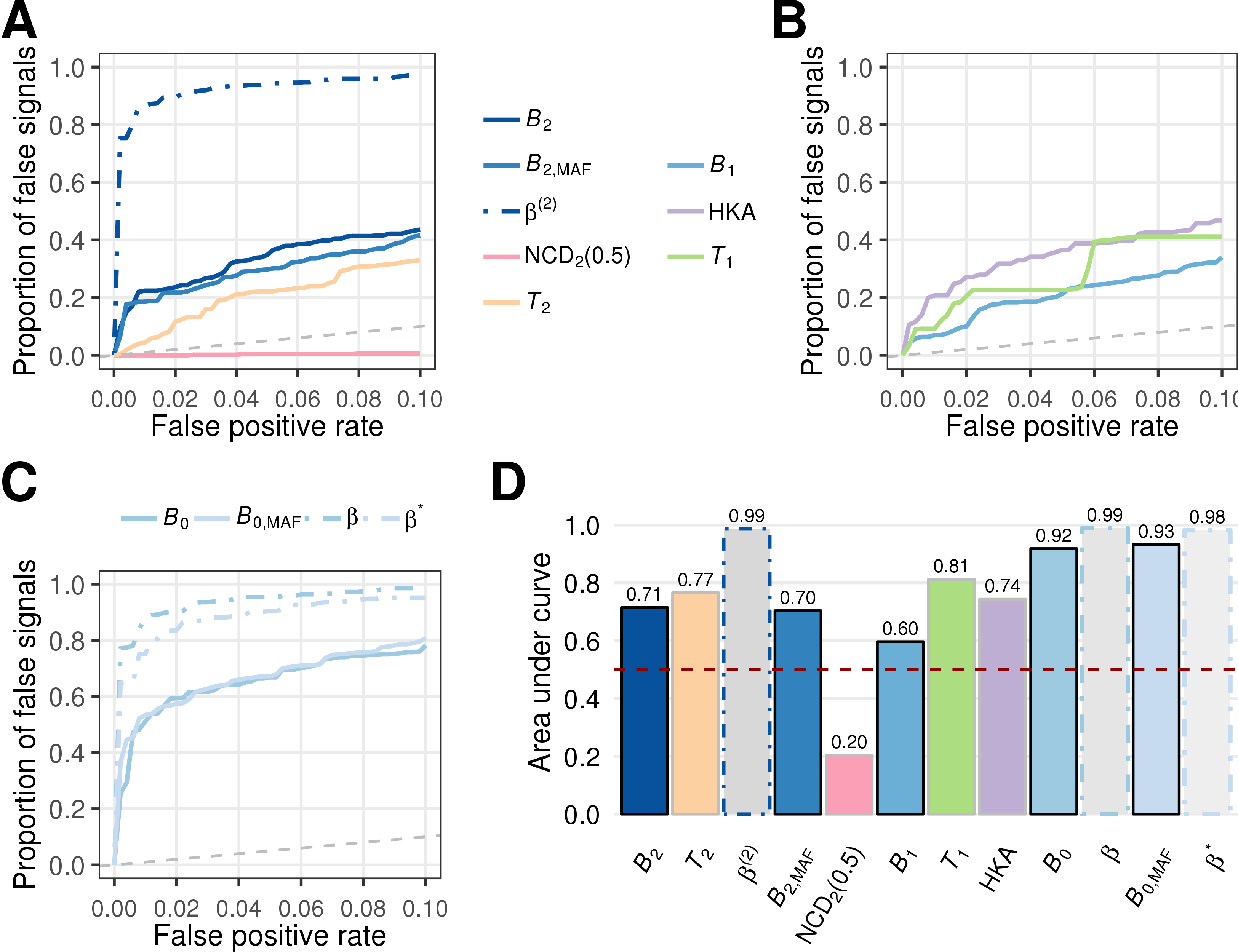

Supplement: msaa134_supplementary_data [file msaa134_supplementary_data.zip › BallerMix_final/figures/FigS12_splitView_HCG_5Mut_01ROC+powerbar_alphaB+stats.png]

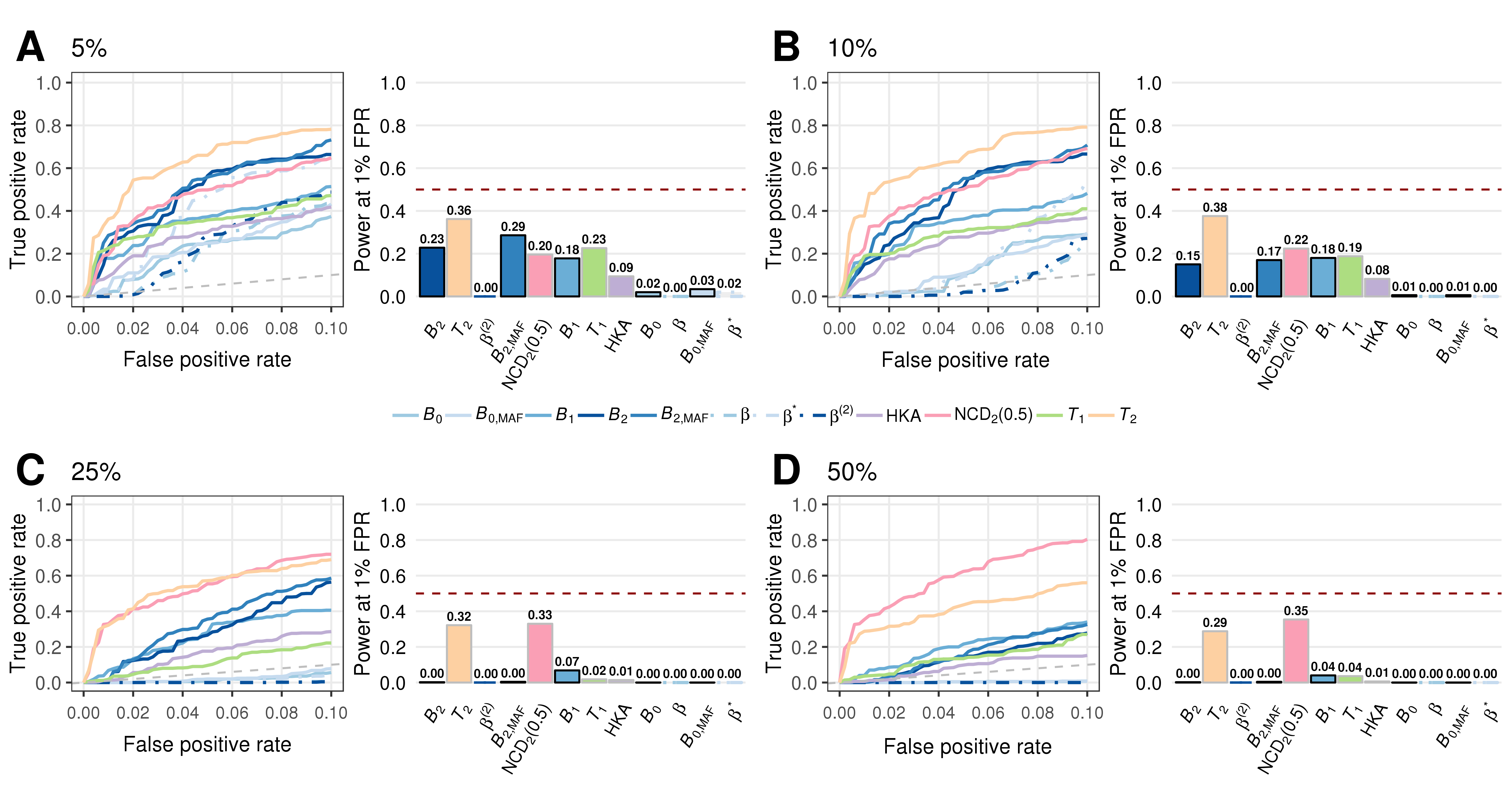

Supplement: msaa134_supplementary_data [file msaa134_supplementary_data.zip › BallerMix_final/figures/FigS13_mixNeut_rerun_ROC+bars.png]

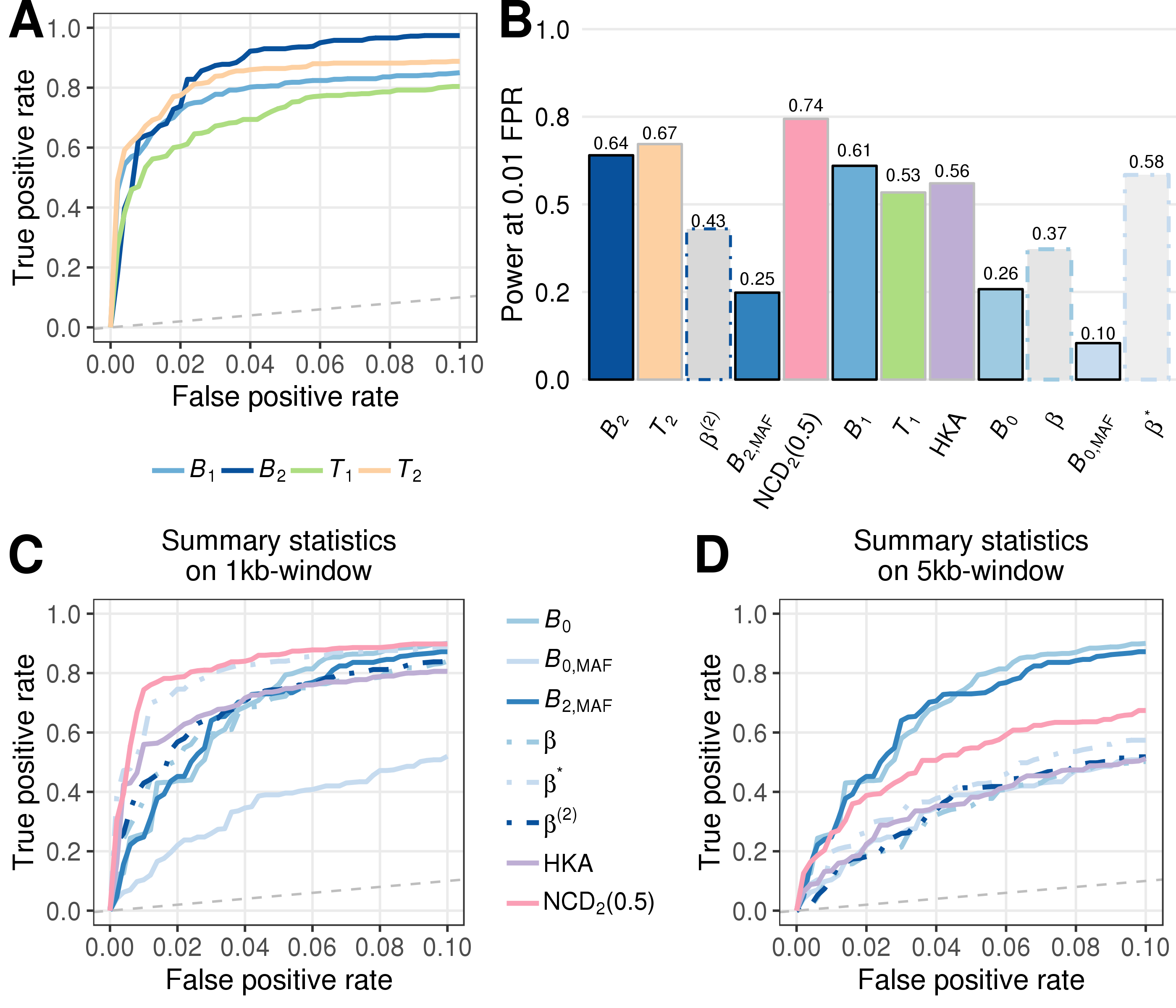

Supplement: msaa134_supplementary_data [file msaa134_supplementary_data.zip › BallerMix_final/figures/FigS14_newHCG_5Mut_5MYA_s001_h20_01ROC+bar_60snp-1v5kb.png]

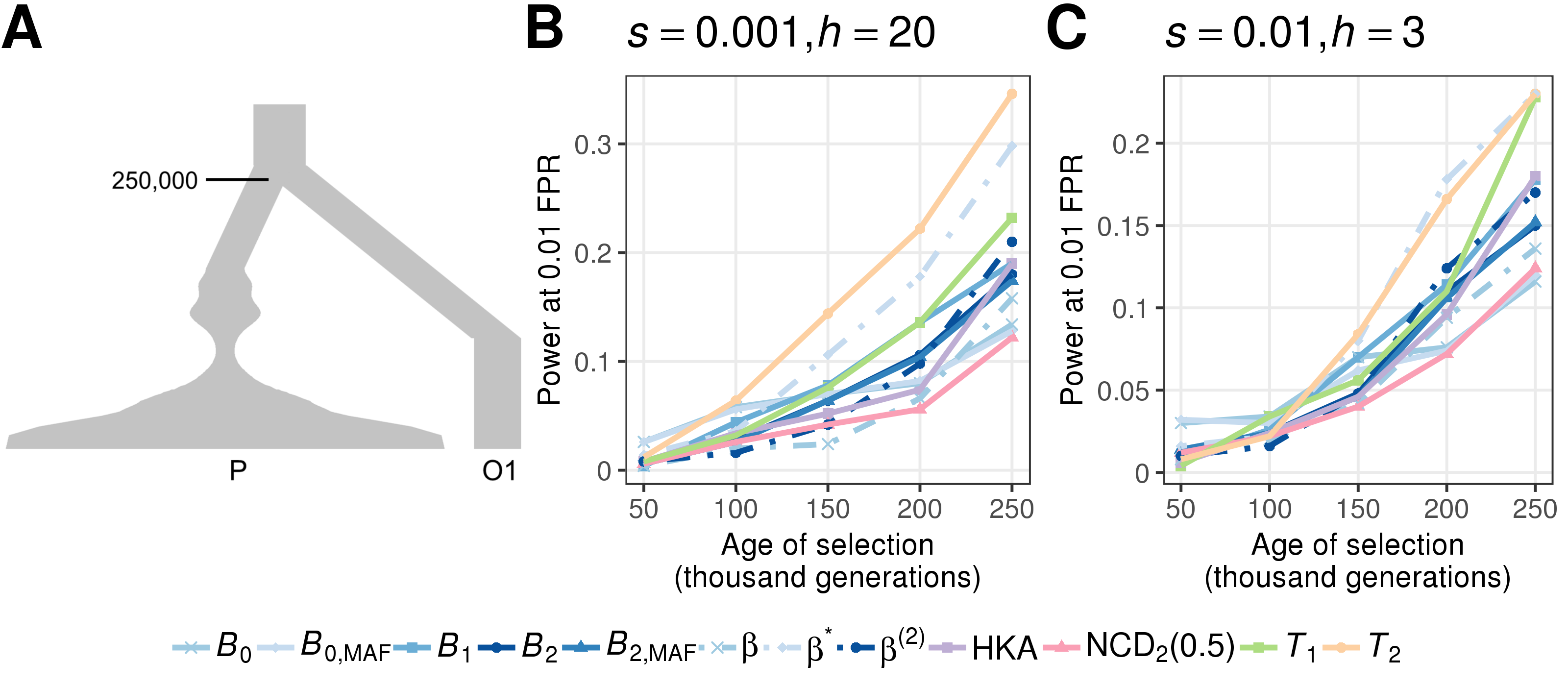

Supplement: msaa134_supplementary_data [file msaa134_supplementary_data.zip › BallerMix_final/figures/FigS15_Time-power_HC_CEU2_1-5mya_alphaB+Stats_3panel.png]

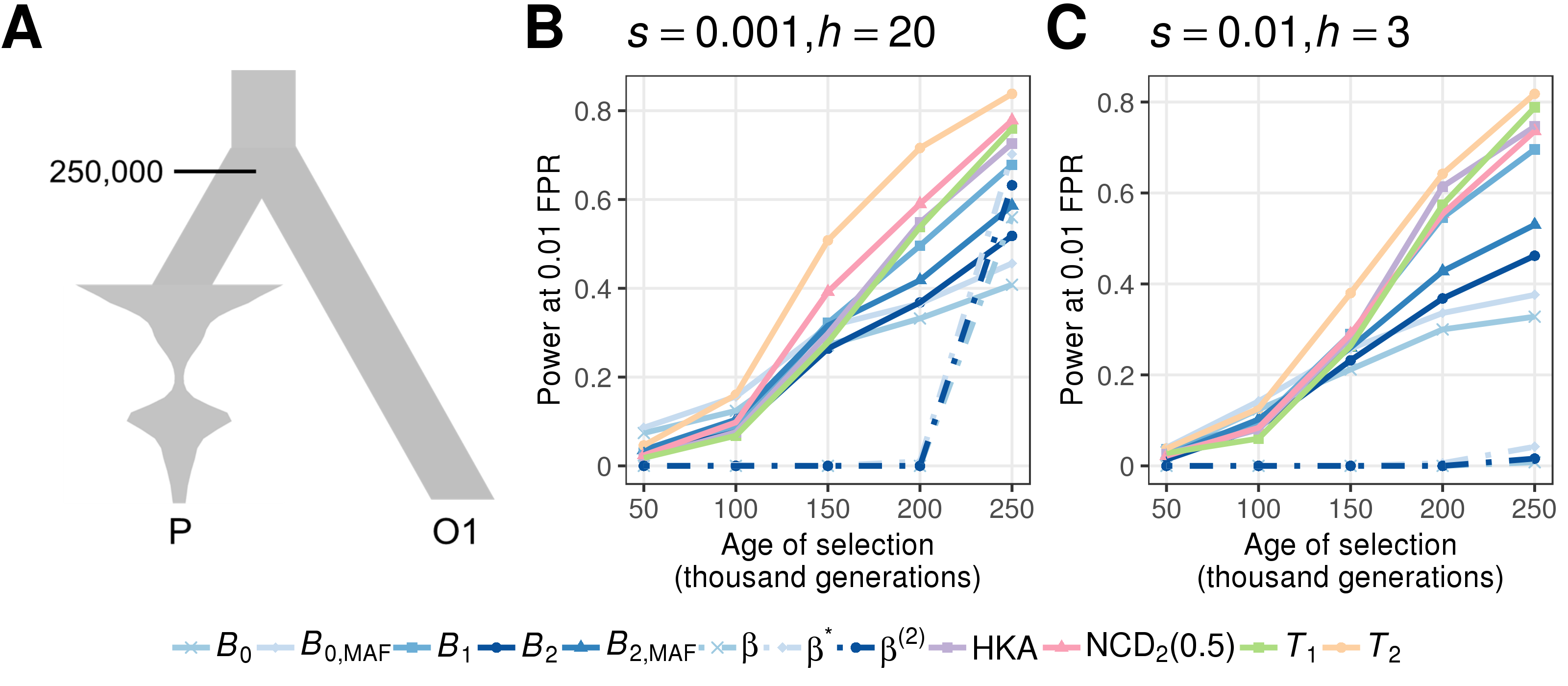

Supplement: msaa134_supplementary_data [file msaa134_supplementary_data.zip › BallerMix_final/figures/FigS16_Time-power_HC_Dzeeta_psmc_1-5mya_alphaB+Stats_3panel.png]

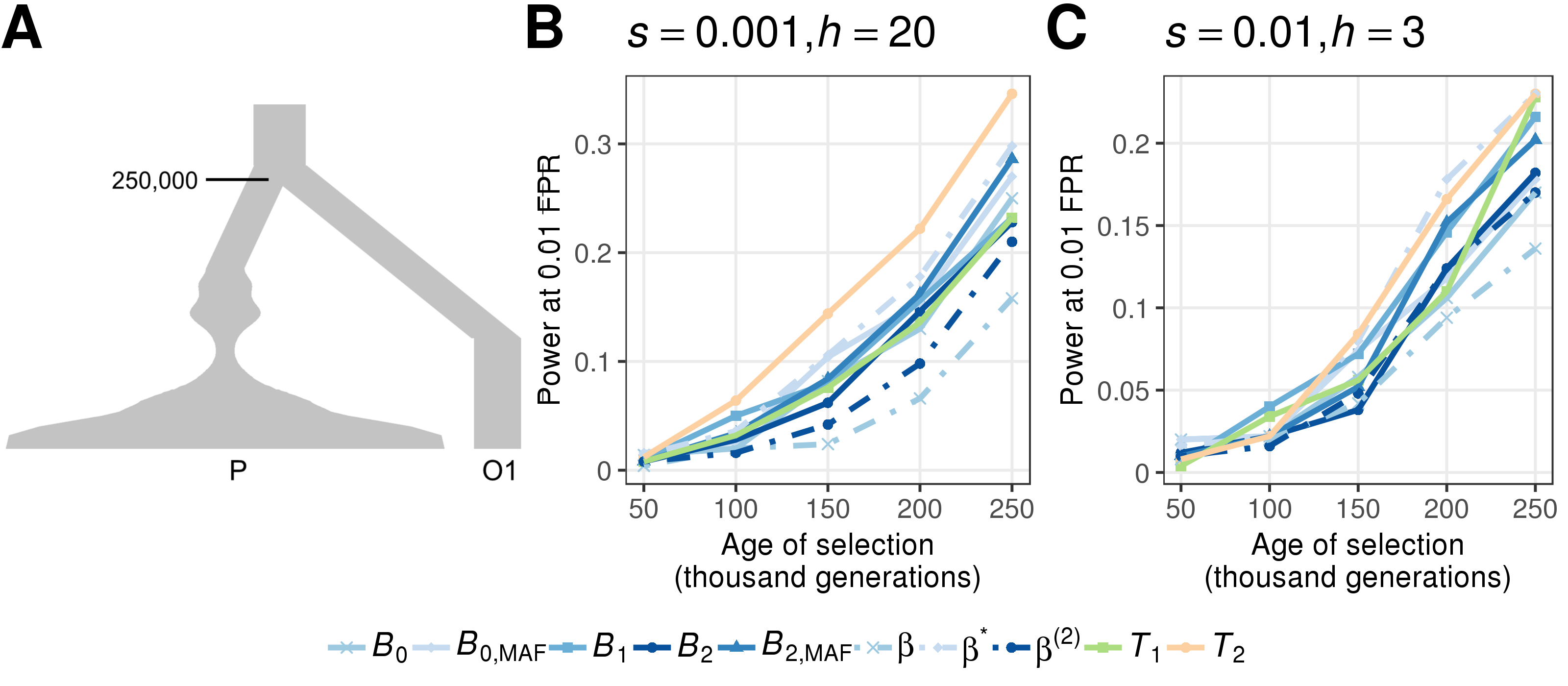

Supplement: msaa134_supplementary_data [file msaa134_supplementary_data.zip › BallerMix_final/figures/FigS17_Time-power_HC_CEU2_1-5mya_matchB+Stats_3panel.png]

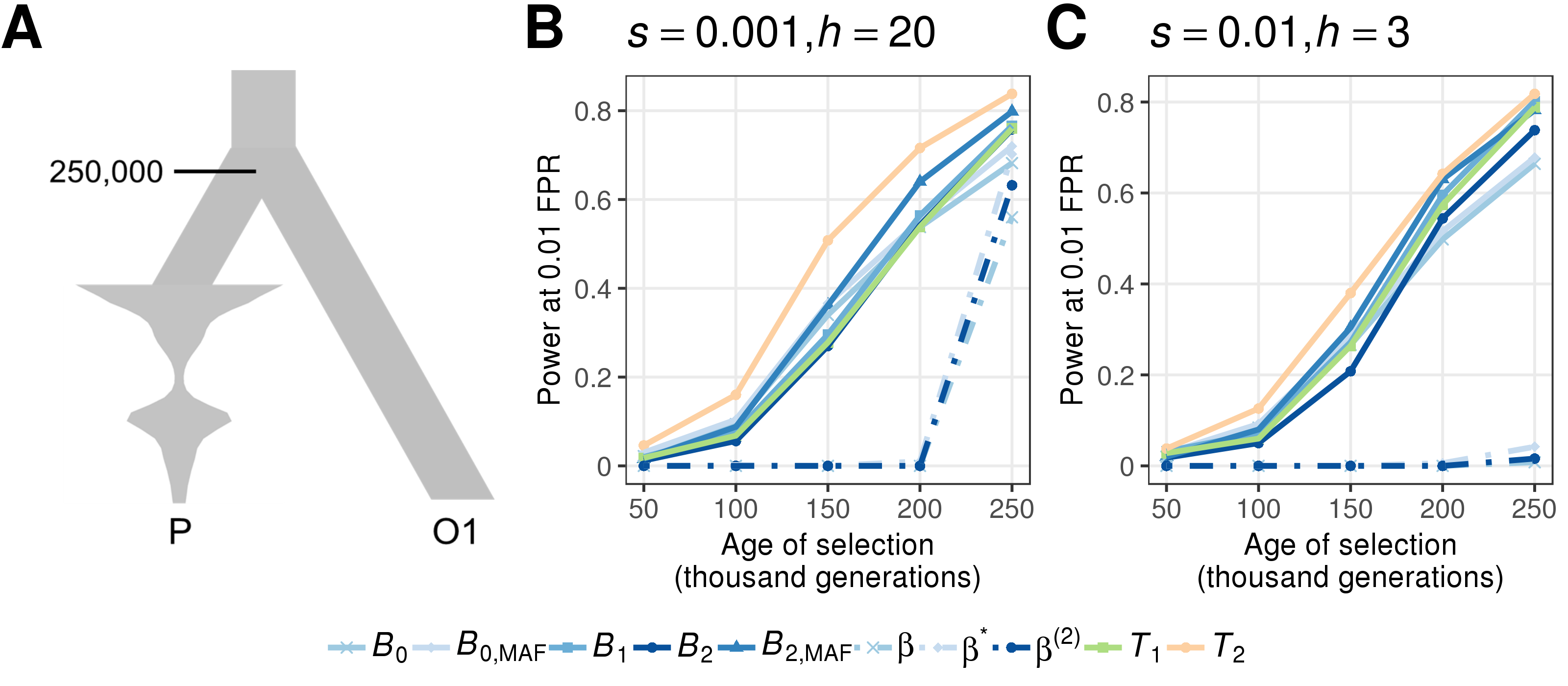

Supplement: msaa134_supplementary_data [file msaa134_supplementary_data.zip › BallerMix_final/figures/FigS18_Time-power_HC_Dzeeta_psmc_1-5mya_matchB+Stats_3panel.png]

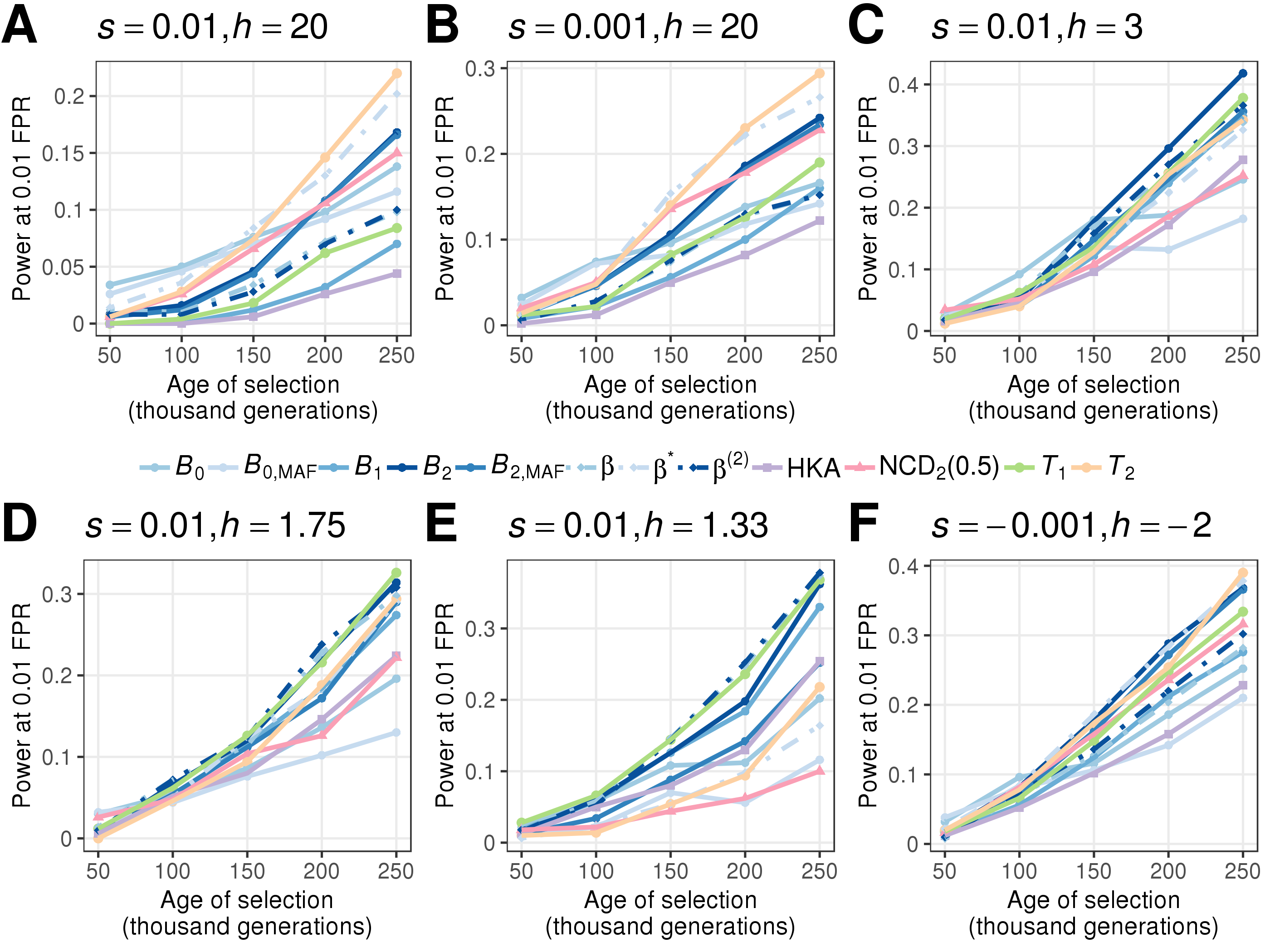

Supplement: msaa134_supplementary_data [file msaa134_supplementary_data.zip › BallerMix_final/figures/FigS19_Time-power_HCB_splitMut_1-5mya_B+Stats_6panel.png]

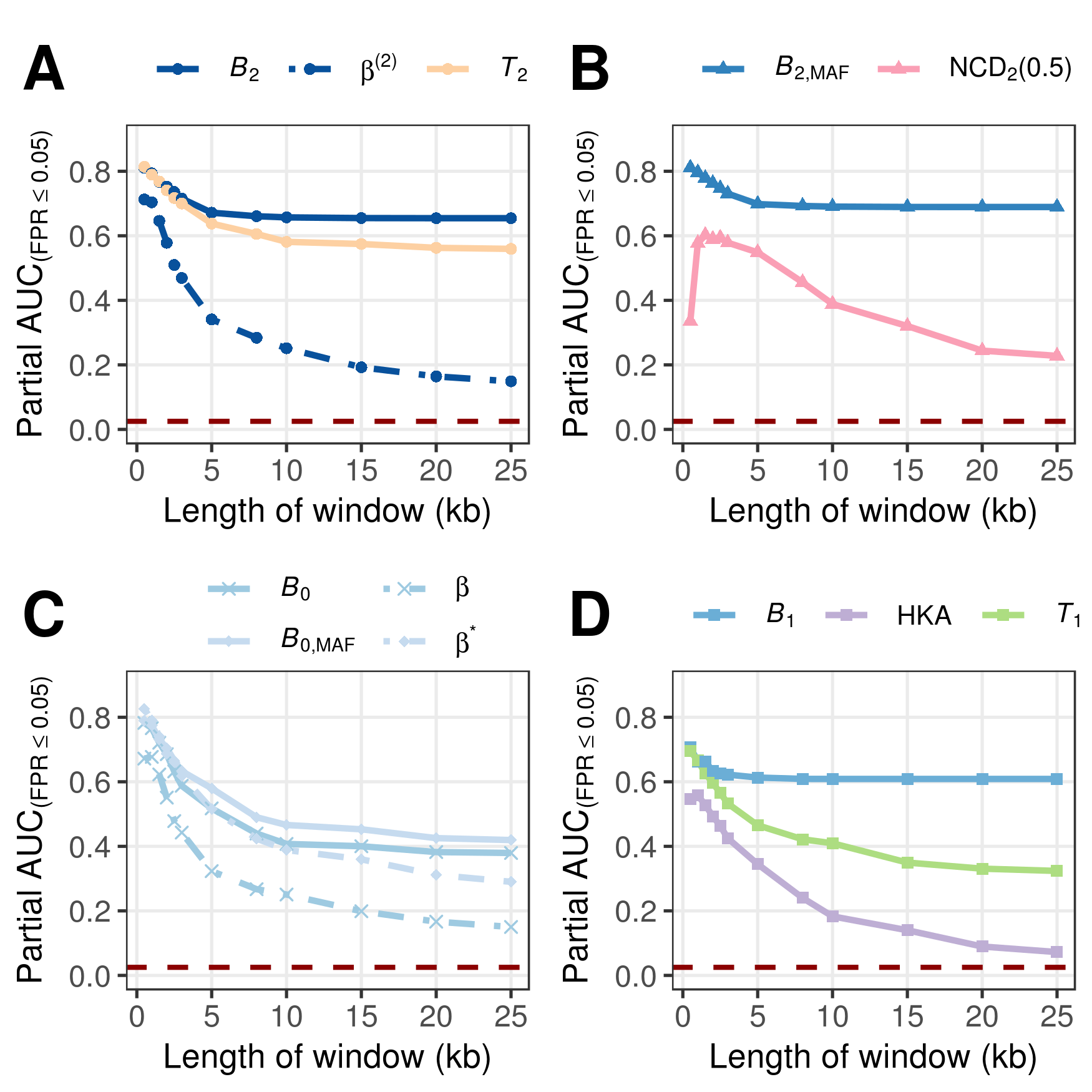

Supplement: msaa134_supplementary_data [file msaa134_supplementary_data.zip › BallerMix_final/figures/FigS2_splitPanel-winSize-05AUC_HCG_15MYA_s001_h20.png]

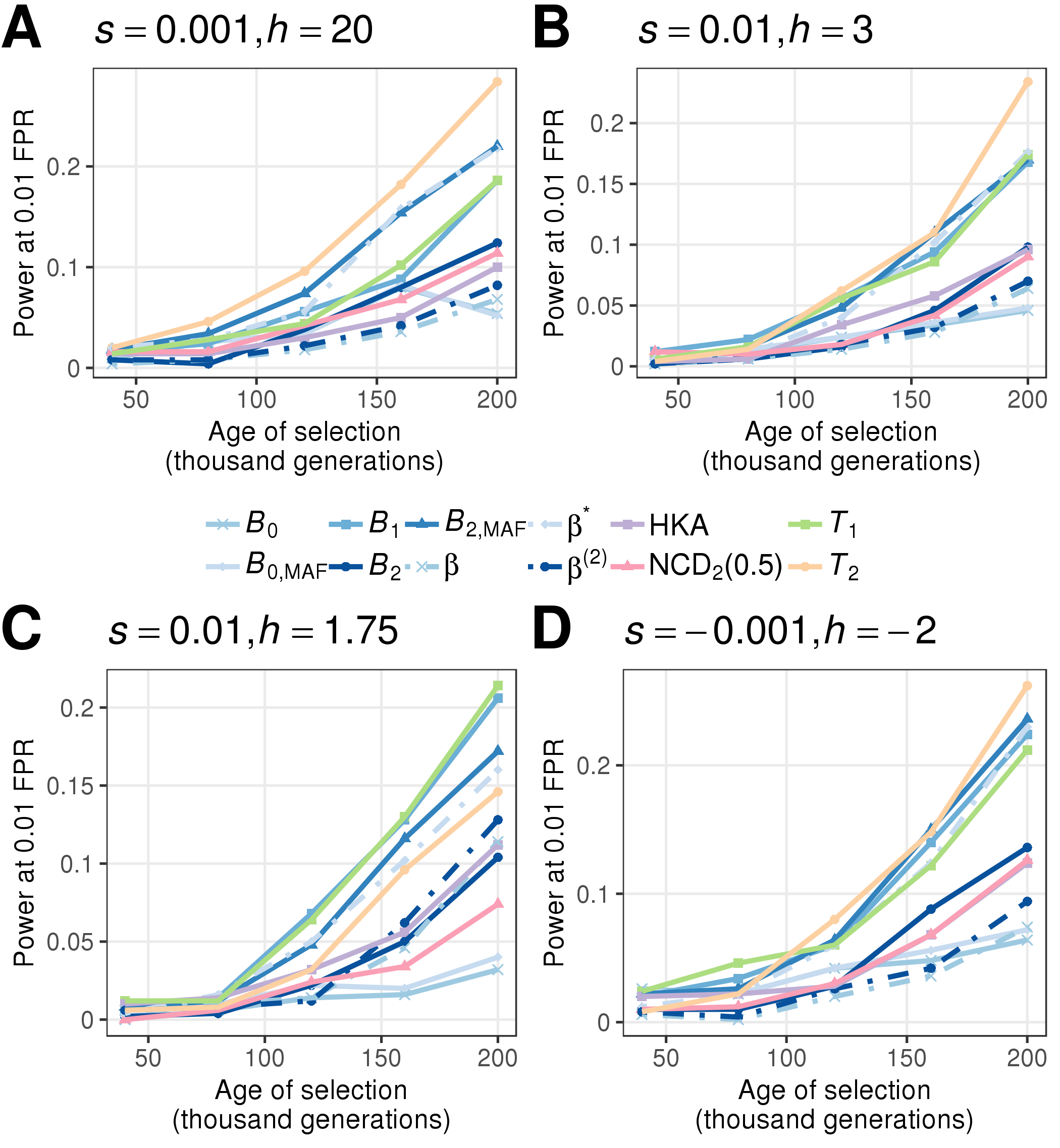

Supplement: msaa134_supplementary_data [file msaa134_supplementary_data.zip › BallerMix_final/figures/FigS20_Time-power_HCB_splitMut_CEU_1-5mya_B+Stats_4panel.png]

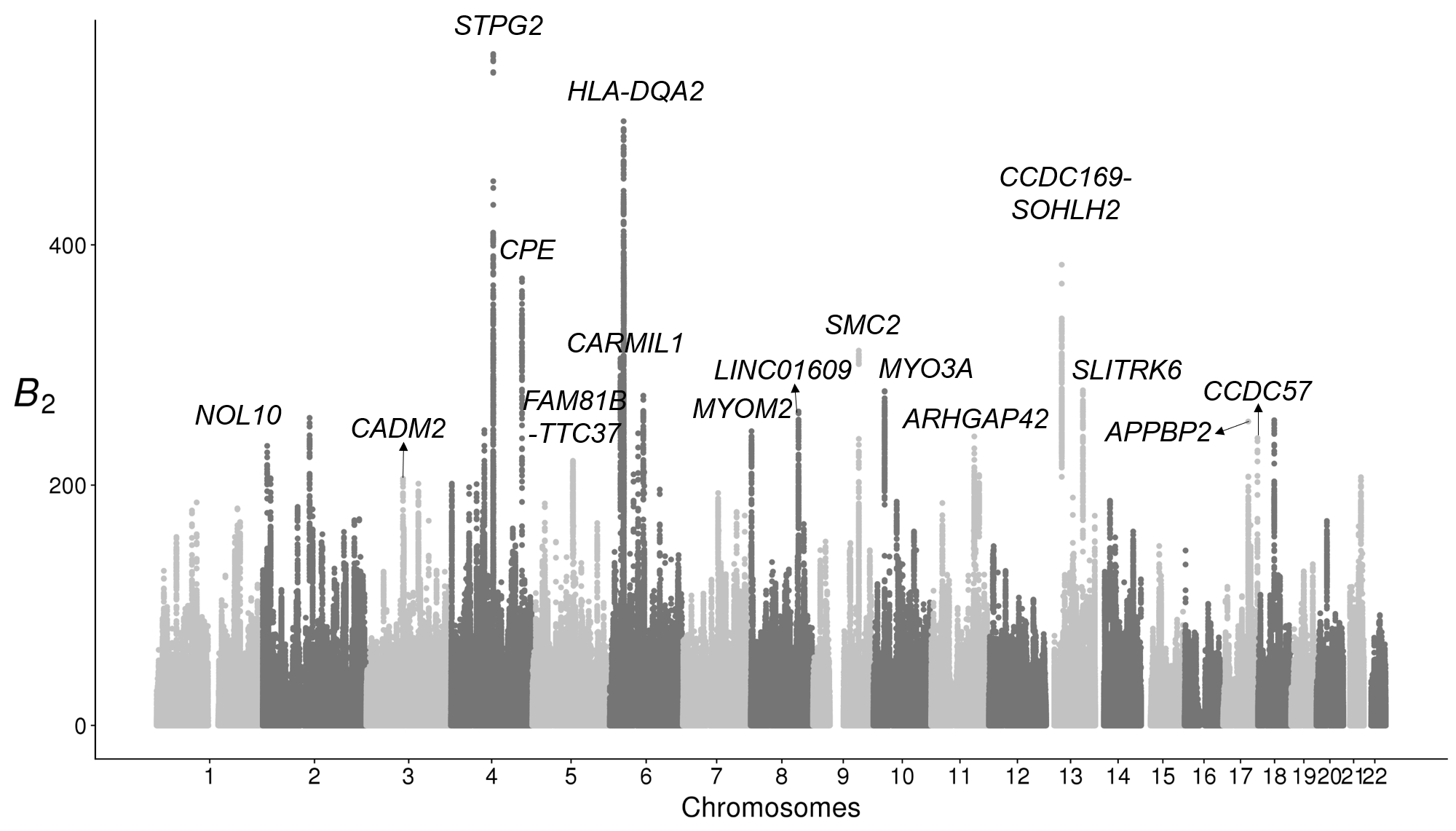

Supplement: msaa134_supplementary_data [file msaa134_supplementary_data.zip › BallerMix_final/figures/FigS21_newAnnt-B2_wg_allYRI_HW_CRG+RepeatMasker+SD1+SD2.png]

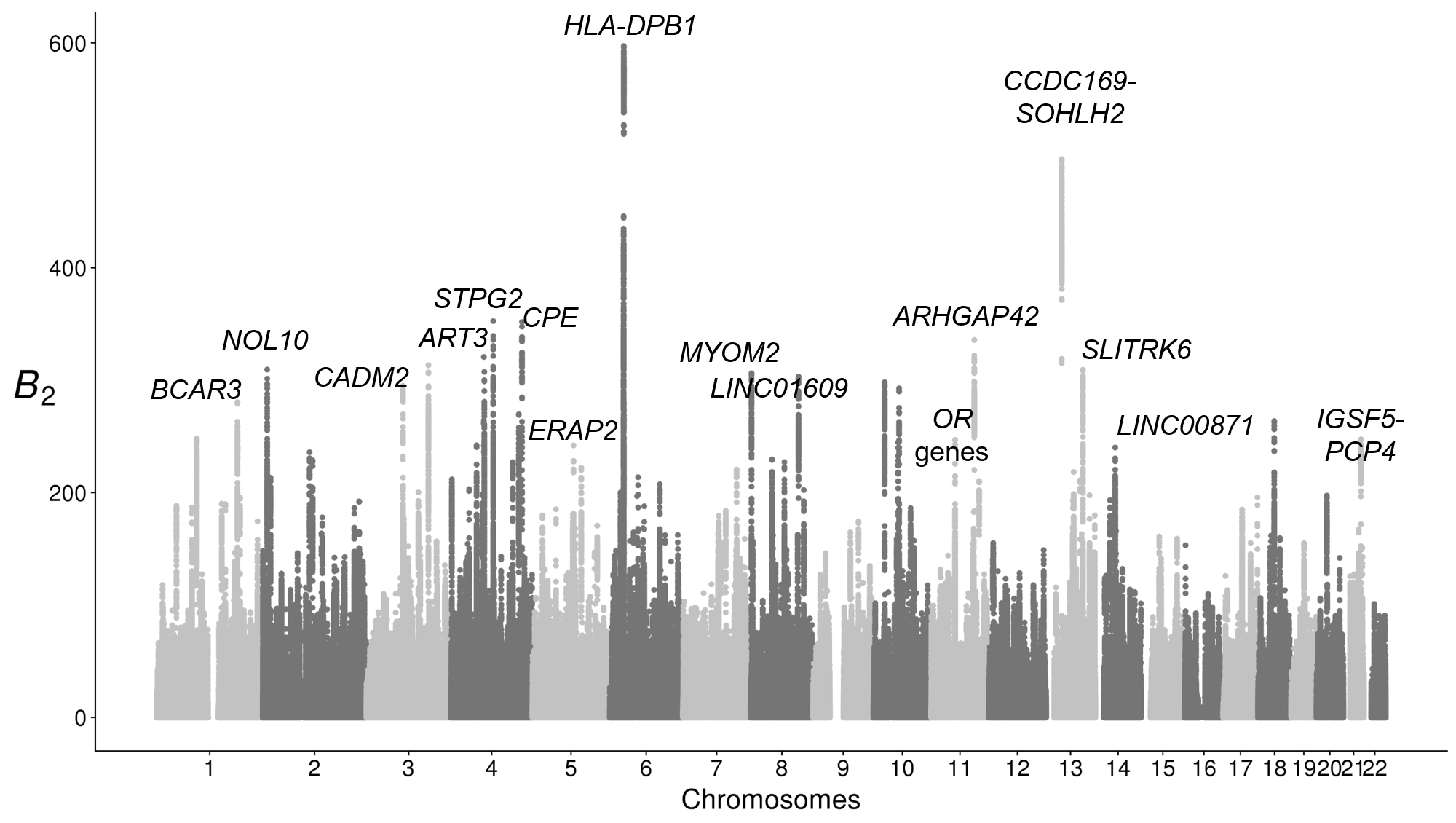

Supplement: msaa134_supplementary_data [file msaa134_supplementary_data.zip › BallerMix_final/figures/FigS22_newAnnt-B2_wg_allCEU_HW_CRG+RepeatMasker+SD1+SD2.png]

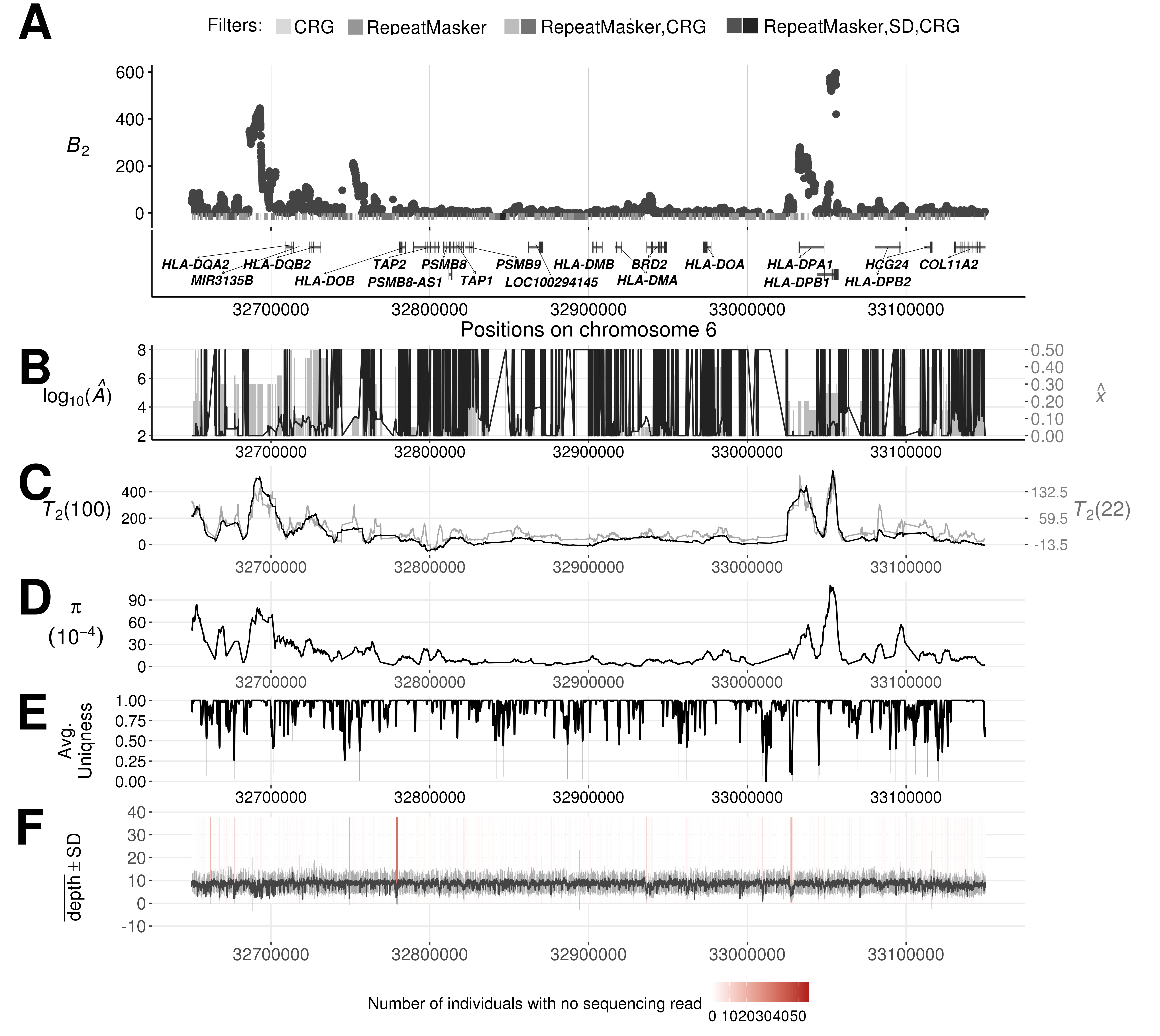

Supplement: msaa134_supplementary_data [file msaa134_supplementary_data.zip › BallerMix_final/figures/FigS23_CEU_HLA-D_Chr6_326-332e5_LR-fancyGene-aD_T2+pi+avgUniq+Depth.png]

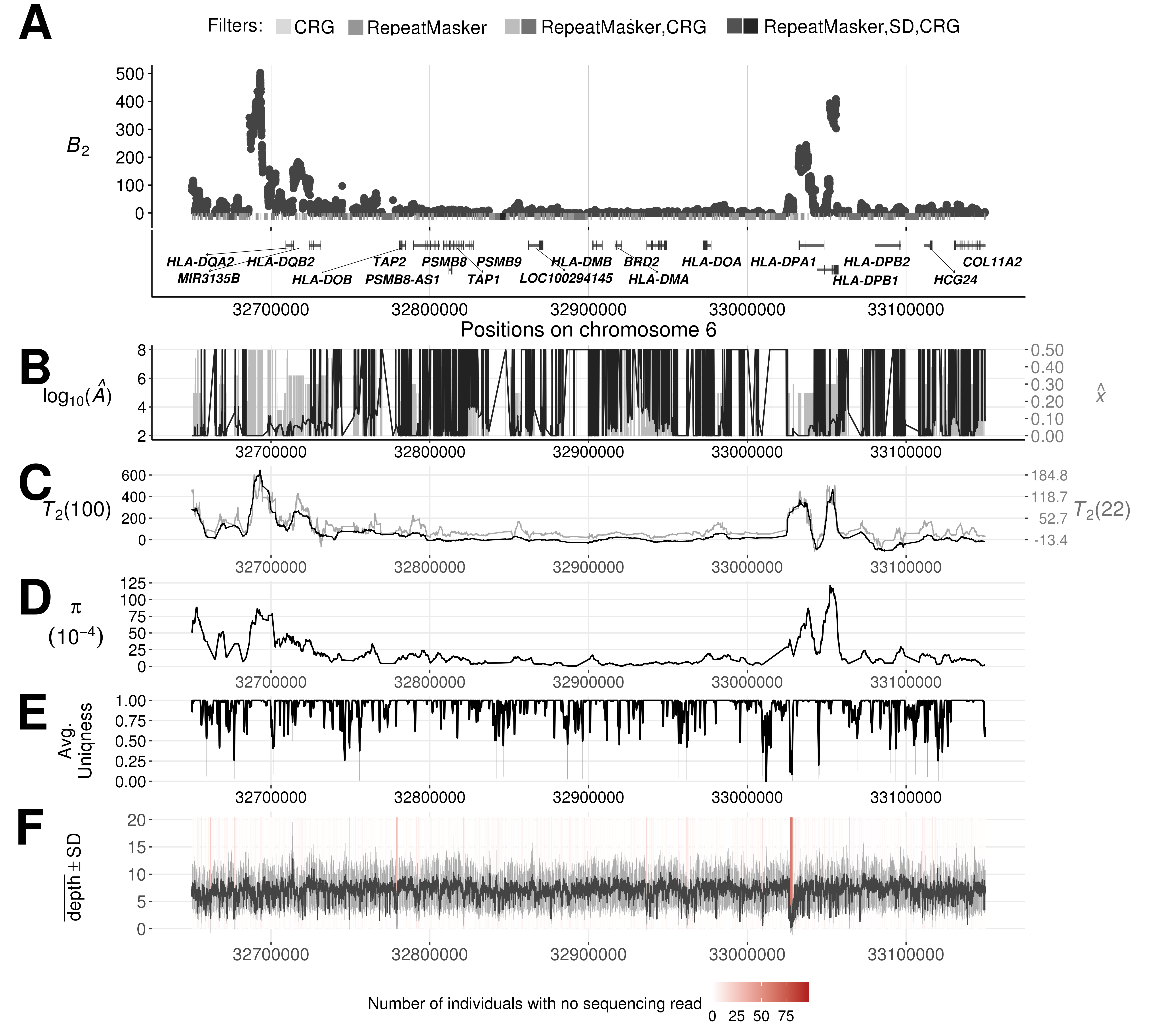

Supplement: msaa134_supplementary_data [file msaa134_supplementary_data.zip › BallerMix_final/figures/FigS24_YRI_HLA-D_Chr6_326-332e5_LR-fancyGene-aD_T2+pi+avgUniq+Depth.png]

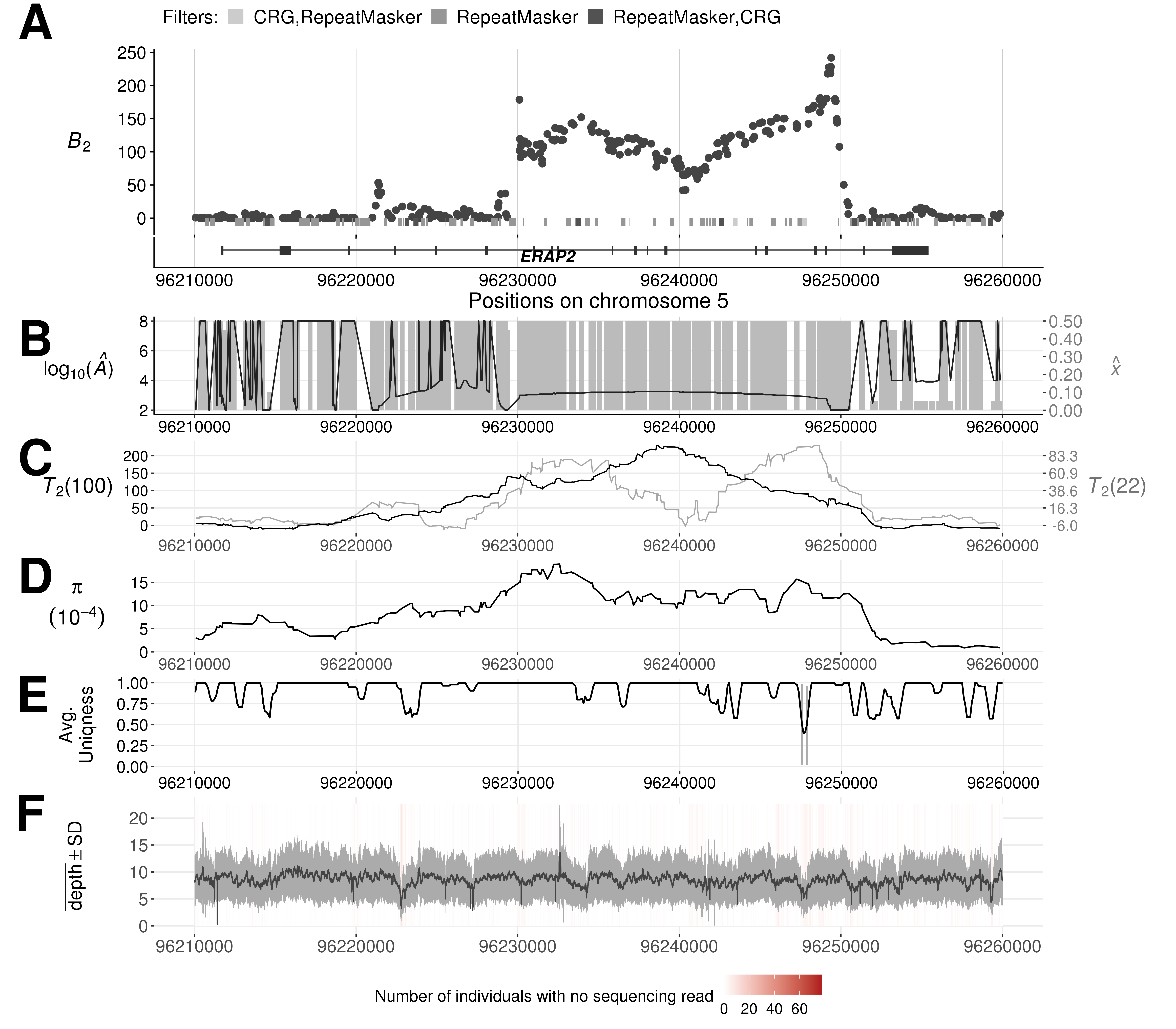

Supplement: msaa134_supplementary_data [file msaa134_supplementary_data.zip › BallerMix_final/figures/FigS25_CEU_B2_alpha1e-8_ERAP2_Chr5_962-963e5_LR-fancyGene-aD_T2+pi+avgUniq+Depth.png]

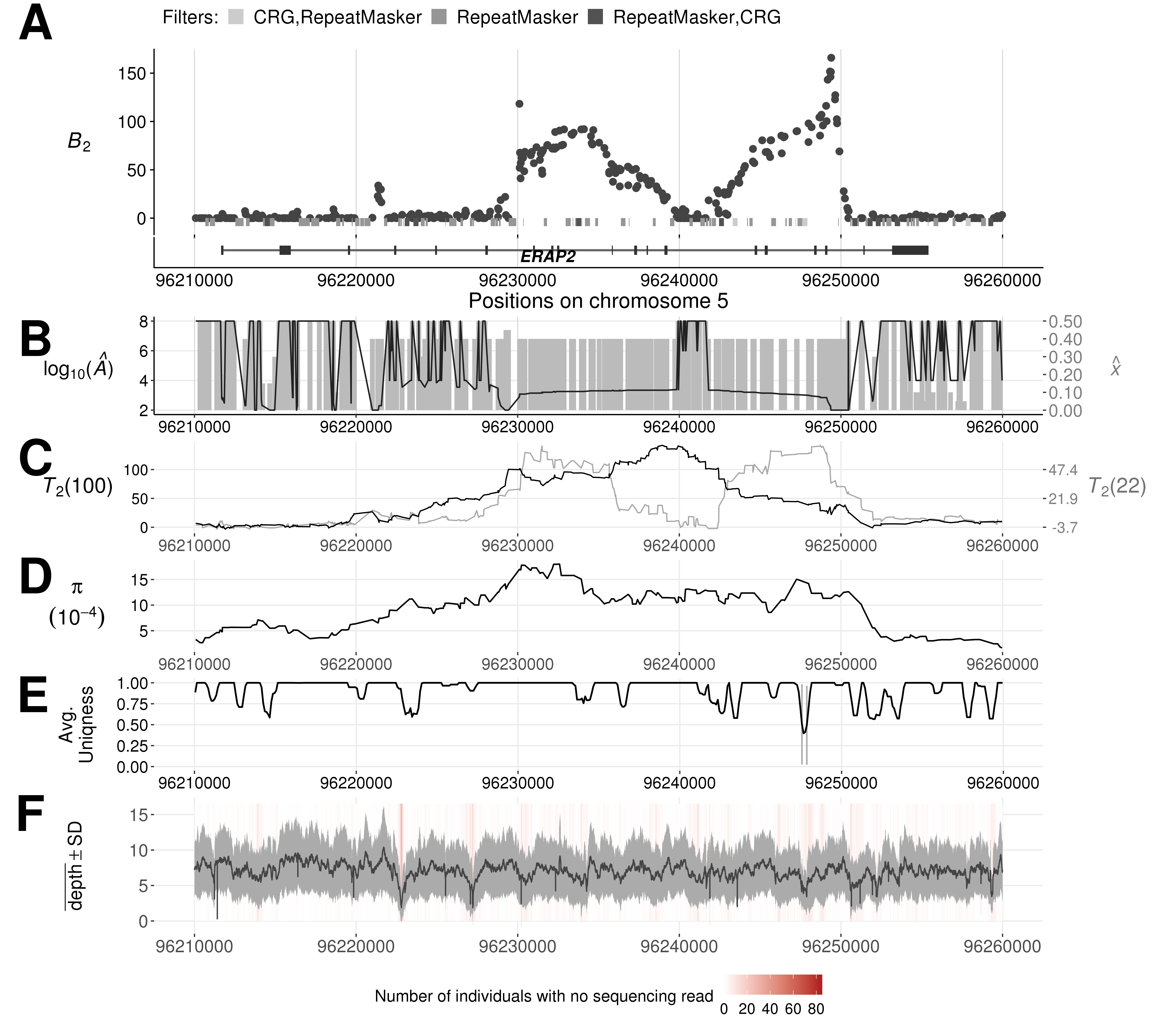

Supplement: msaa134_supplementary_data [file msaa134_supplementary_data.zip › BallerMix_final/figures/FigS26_YRI_B2_alpha1e-8_ERAP2_Chr5_962-963e5_LR-fancyGene-aD_T2+pi+avgUniq+Depth.png]

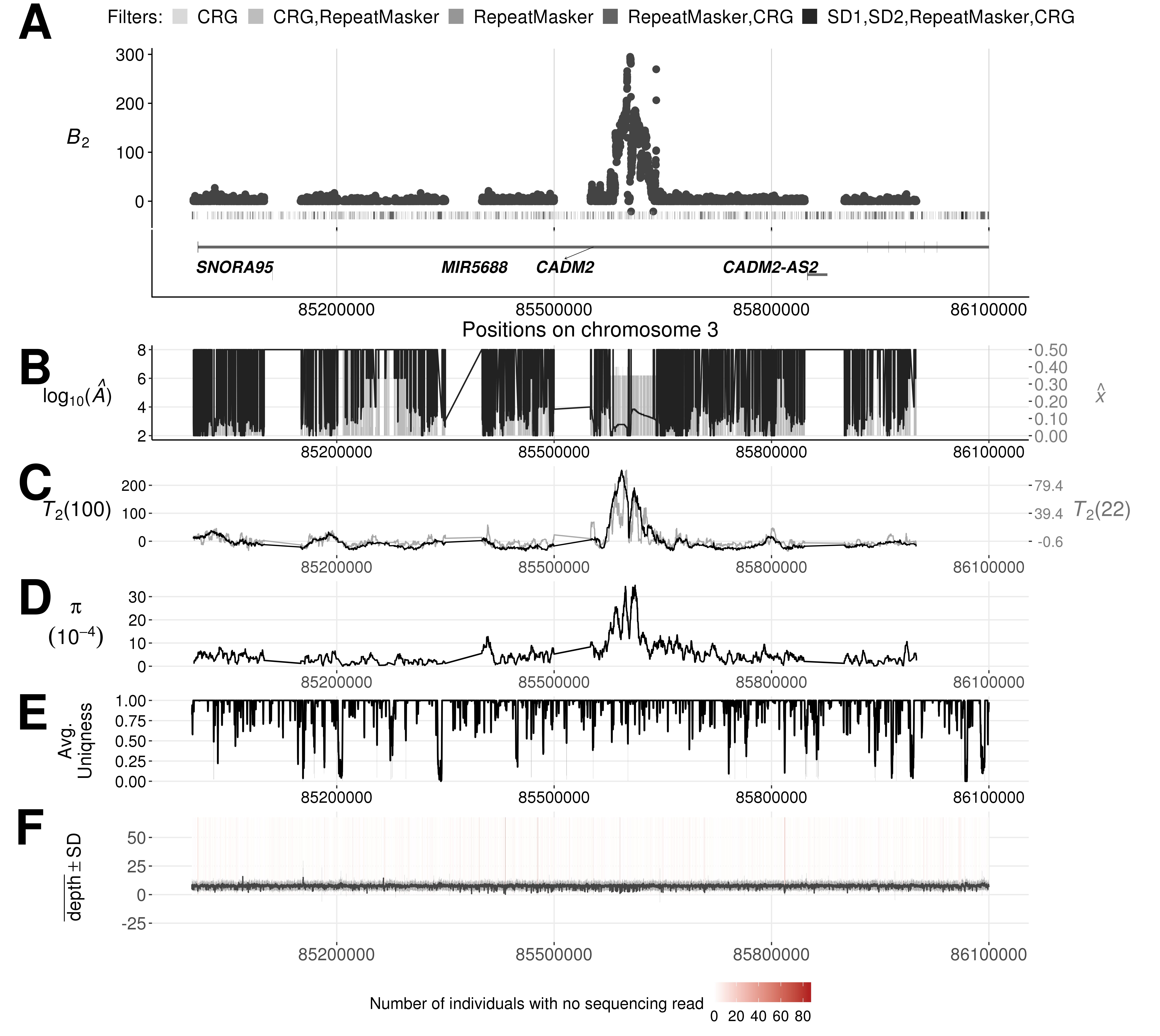

Supplement: msaa134_supplementary_data [file msaa134_supplementary_data.zip › BallerMix_final/figures/FigS27_CEU_B2_alpha1e-8_CADM2_Chr3_850-861e5_LR-fancyGene-aD_T2+pi+avgUniq+Depth.png]

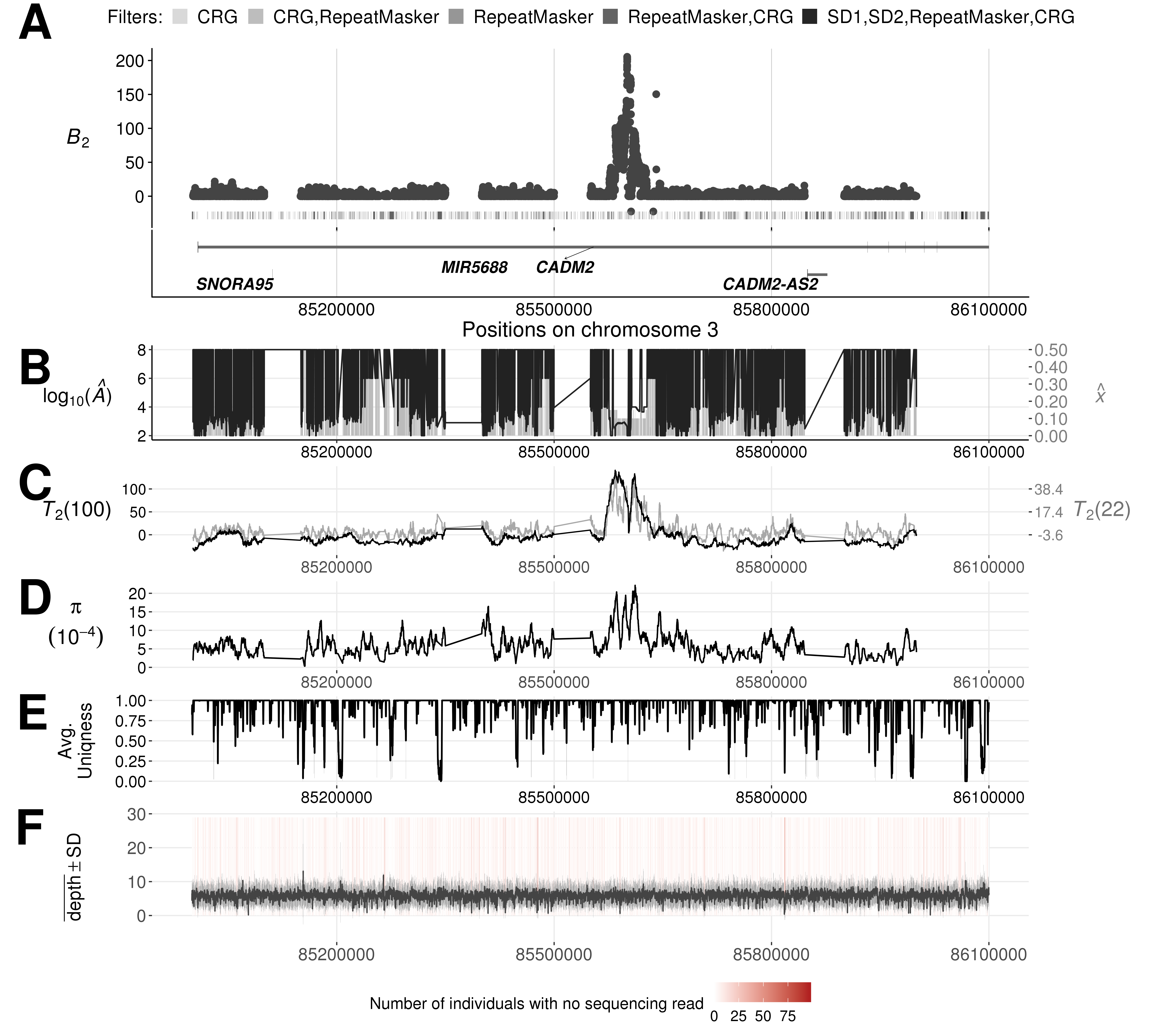

Supplement: msaa134_supplementary_data [file msaa134_supplementary_data.zip › BallerMix_final/figures/FigS28_YRI_B2_alpha1e-8_CADM2_Chr3_850-861e5_LR-fancyGene-aD_T2+pi+avgUniq+Depth.png]

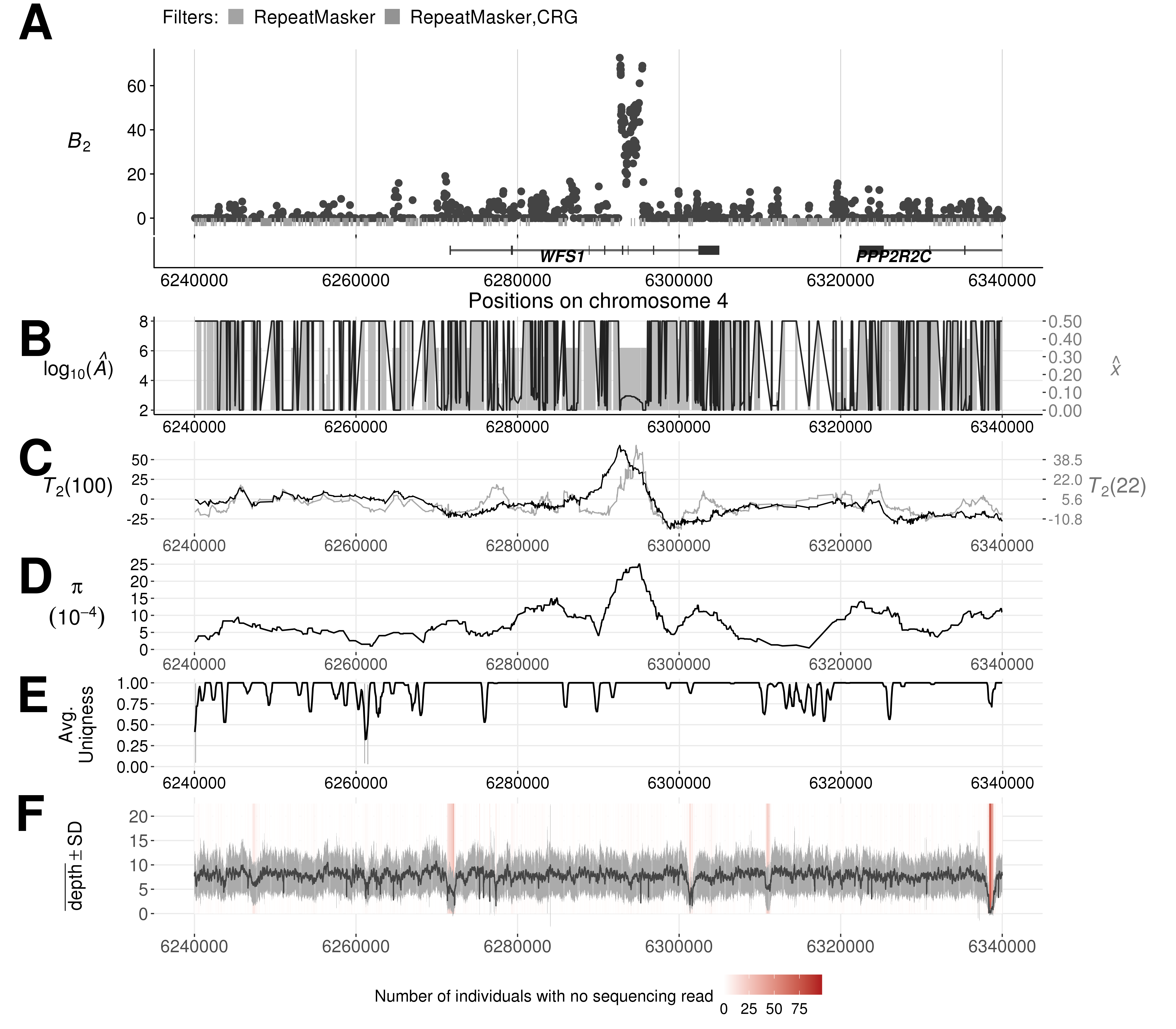

Supplement: msaa134_supplementary_data [file msaa134_supplementary_data.zip › BallerMix_final/figures/FigS29_CEU_B2_alpha1e-8_WFS1_Chr4_62-63e5_LR-fancyGene-aD_T2+pi+avgUniq+Depth.png]

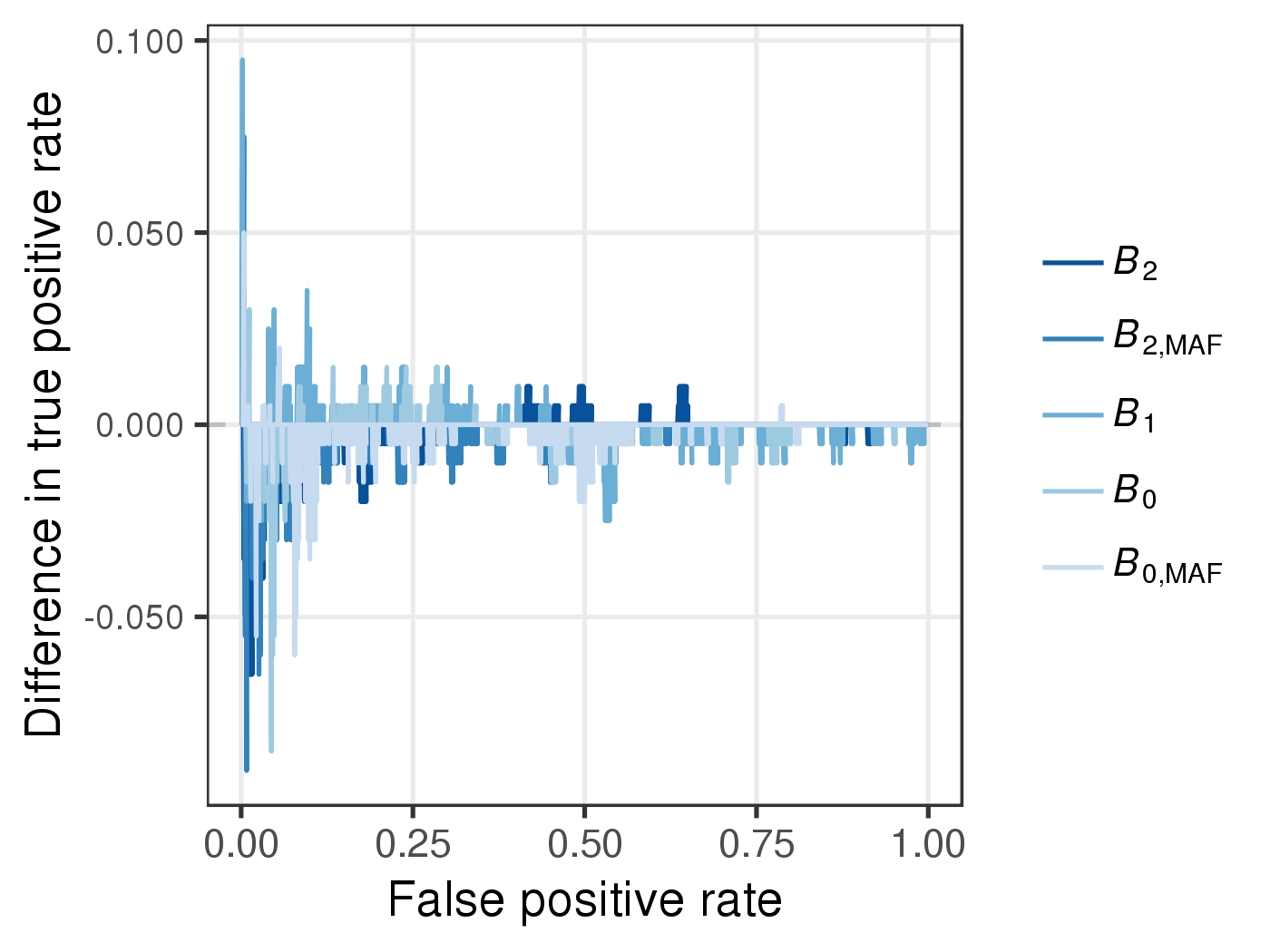

Supplement: msaa134_supplementary_data [file msaa134_supplementary_data.zip › BallerMix_final/figures/FigS3_TPRdiffs-noROC_B-5var_v_T12+stats_HCG_15MYA_s01_h100_all-v-alpha.png]

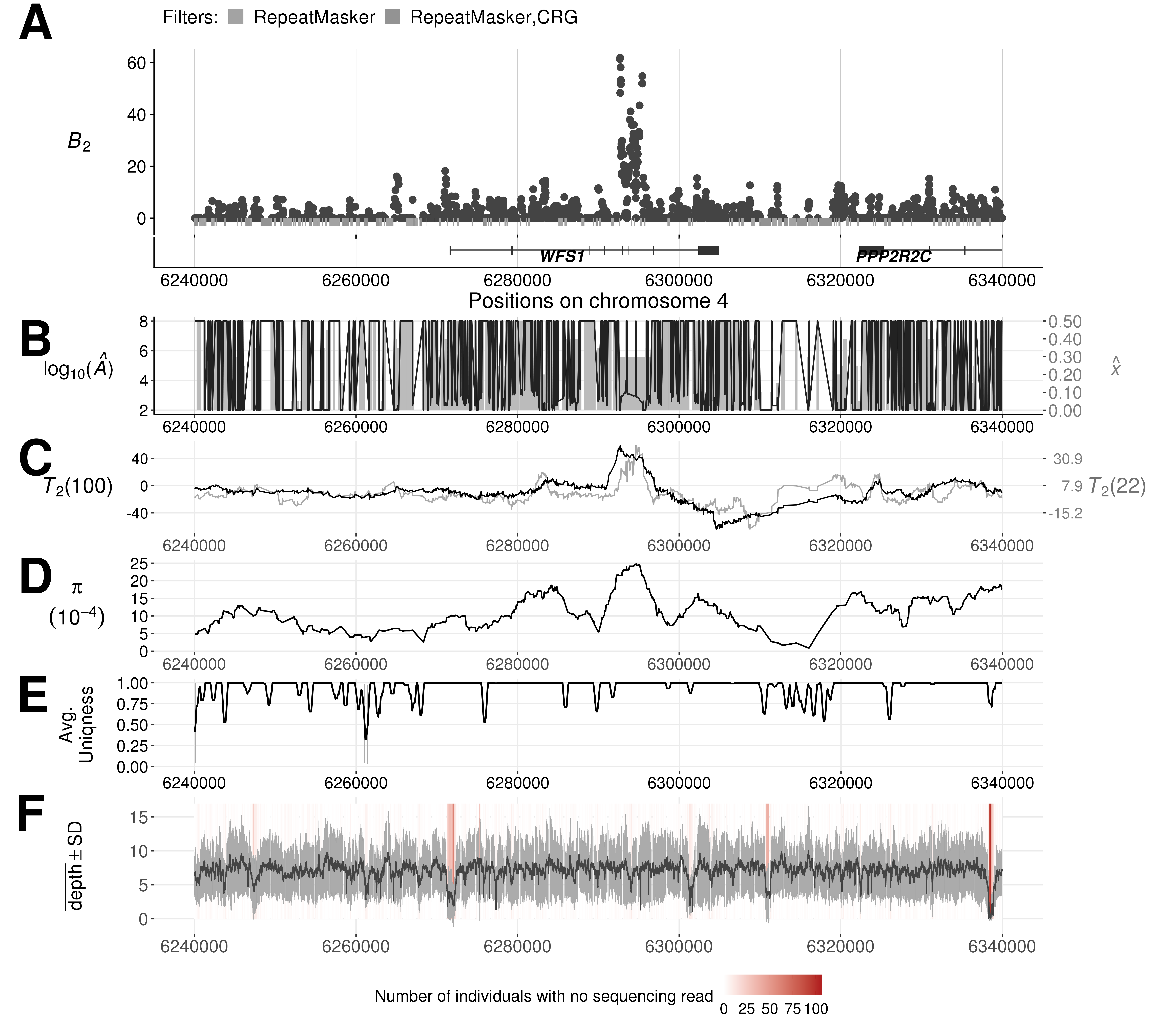

Supplement: msaa134_supplementary_data [file msaa134_supplementary_data.zip › BallerMix_final/figures/FigS30_YRI_B2_alpha1e-8_WFS1_Chr4_62-63e5_LR-fancyGene-aD_T2+pi+avgUniq+Depth.png]

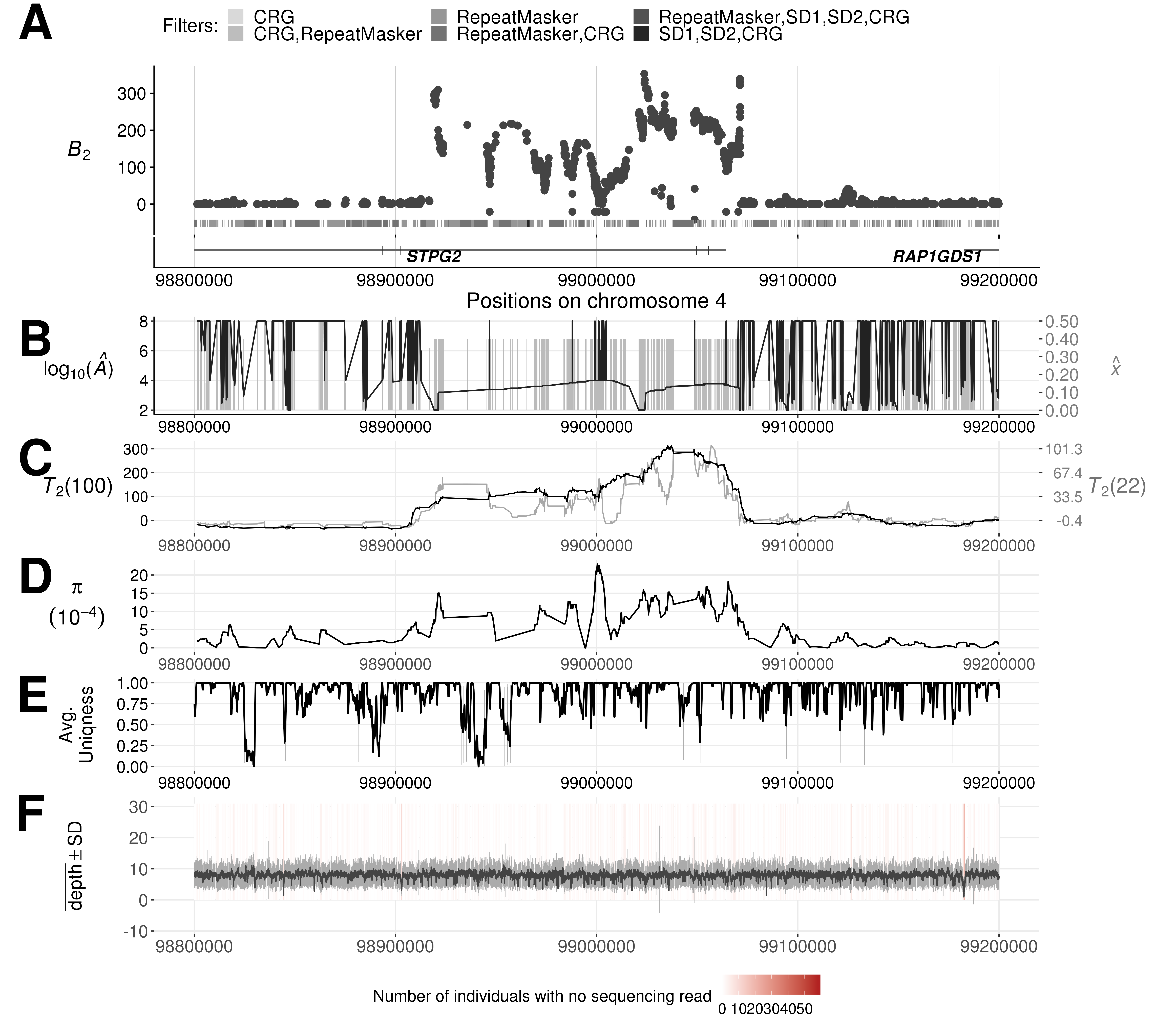

Supplement: msaa134_supplementary_data [file msaa134_supplementary_data.zip › BallerMix_final/figures/FigS31_CEU_B2_alpha1e-8_STPG2_Chr4_988-992e5_LR-fancyGene-aD_T2+pi+avgUniq+Depth.png]

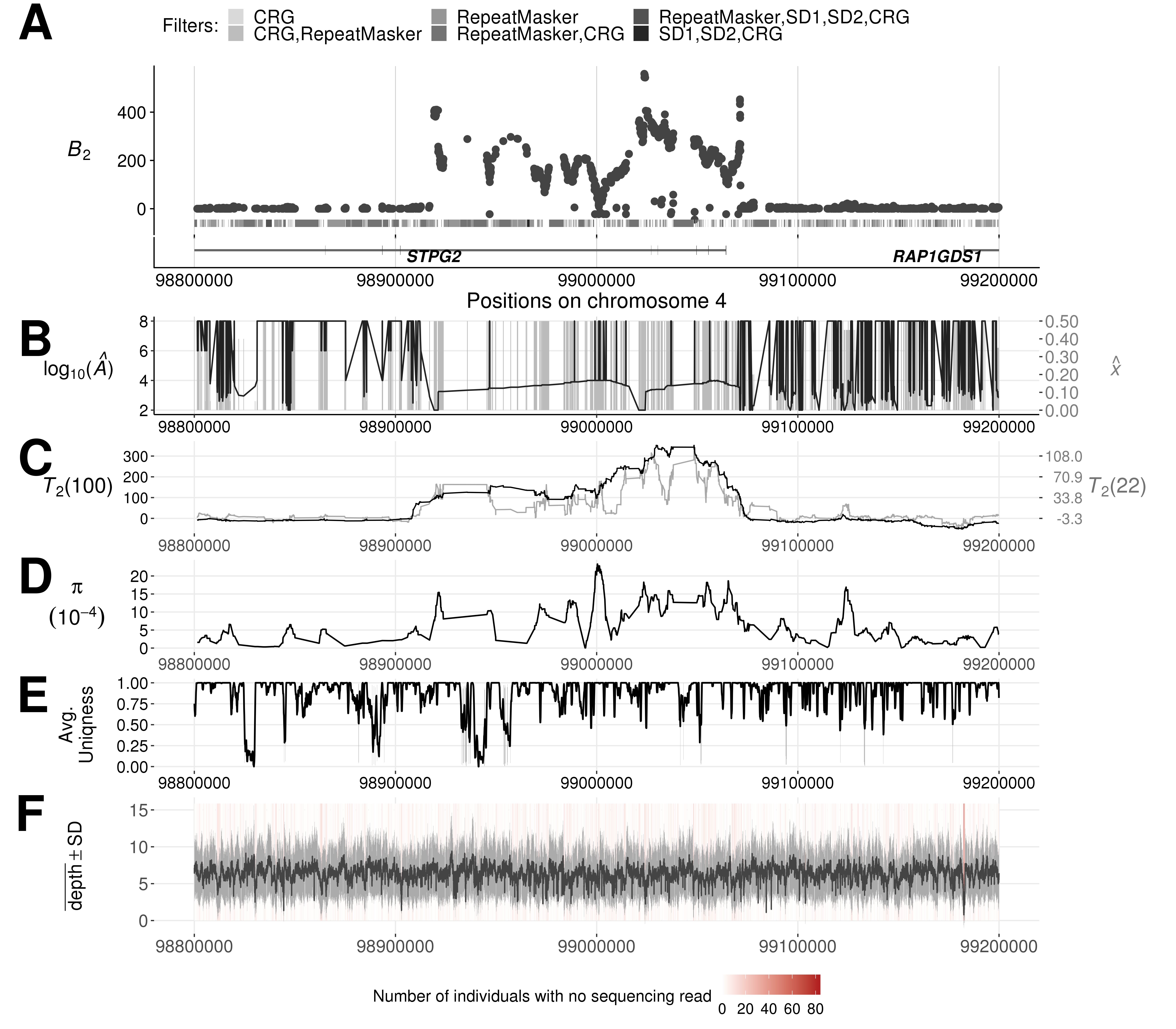

Supplement: msaa134_supplementary_data [file msaa134_supplementary_data.zip › BallerMix_final/figures/FigS32_YRI_B2_alpha1e-8_STPG2_Chr4_988-992e5_LR-fancyGene-aD_T2+pi+avgUniq+Depth.png]

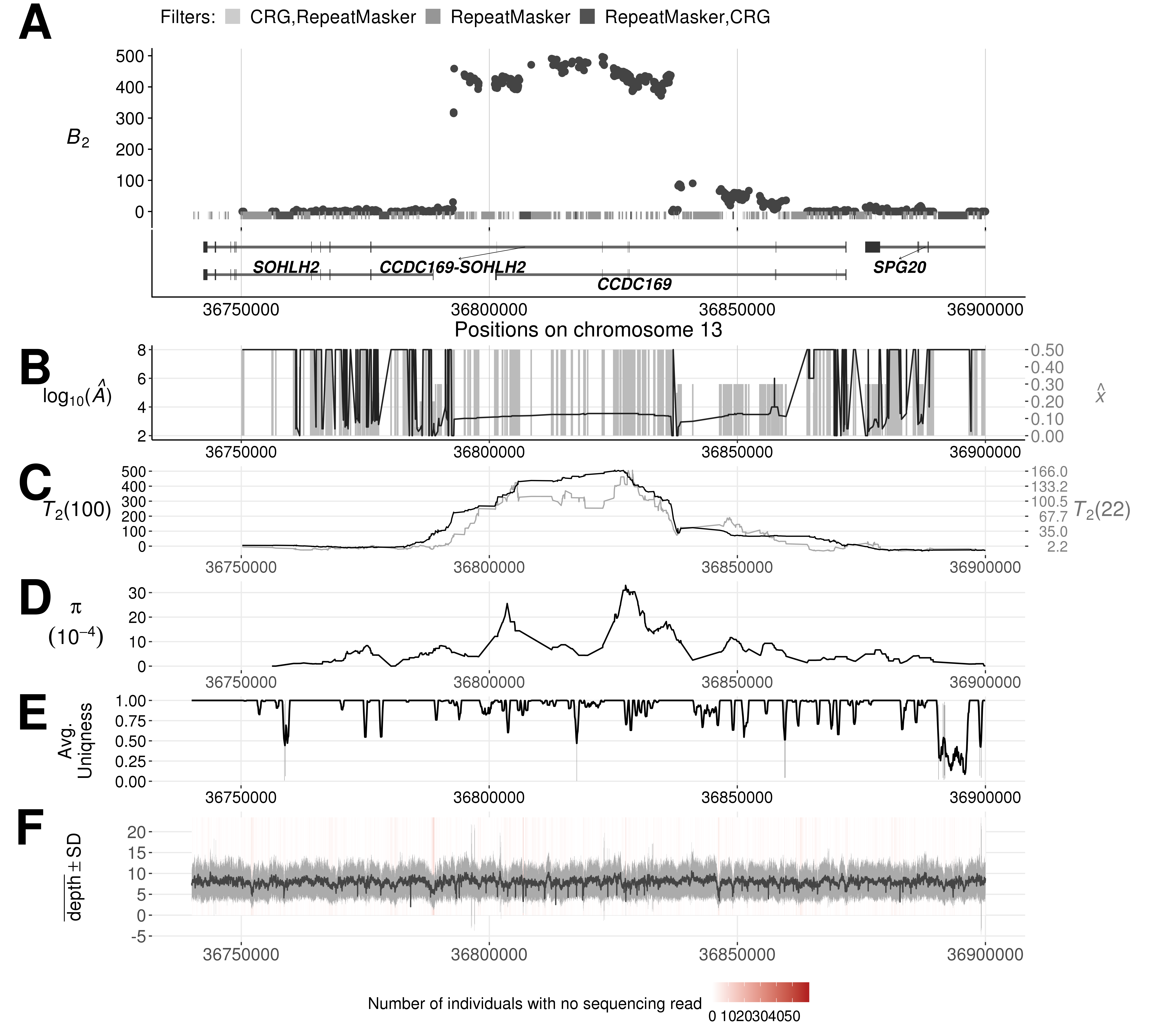

Supplement: msaa134_supplementary_data [file msaa134_supplementary_data.zip › BallerMix_final/figures/FigS33_CEU_B2_alpha1e-8_CCDC169_Chr13_367-369e5_LR-fancyGene-aD_T2+pi+avgUniq+Depth.png]

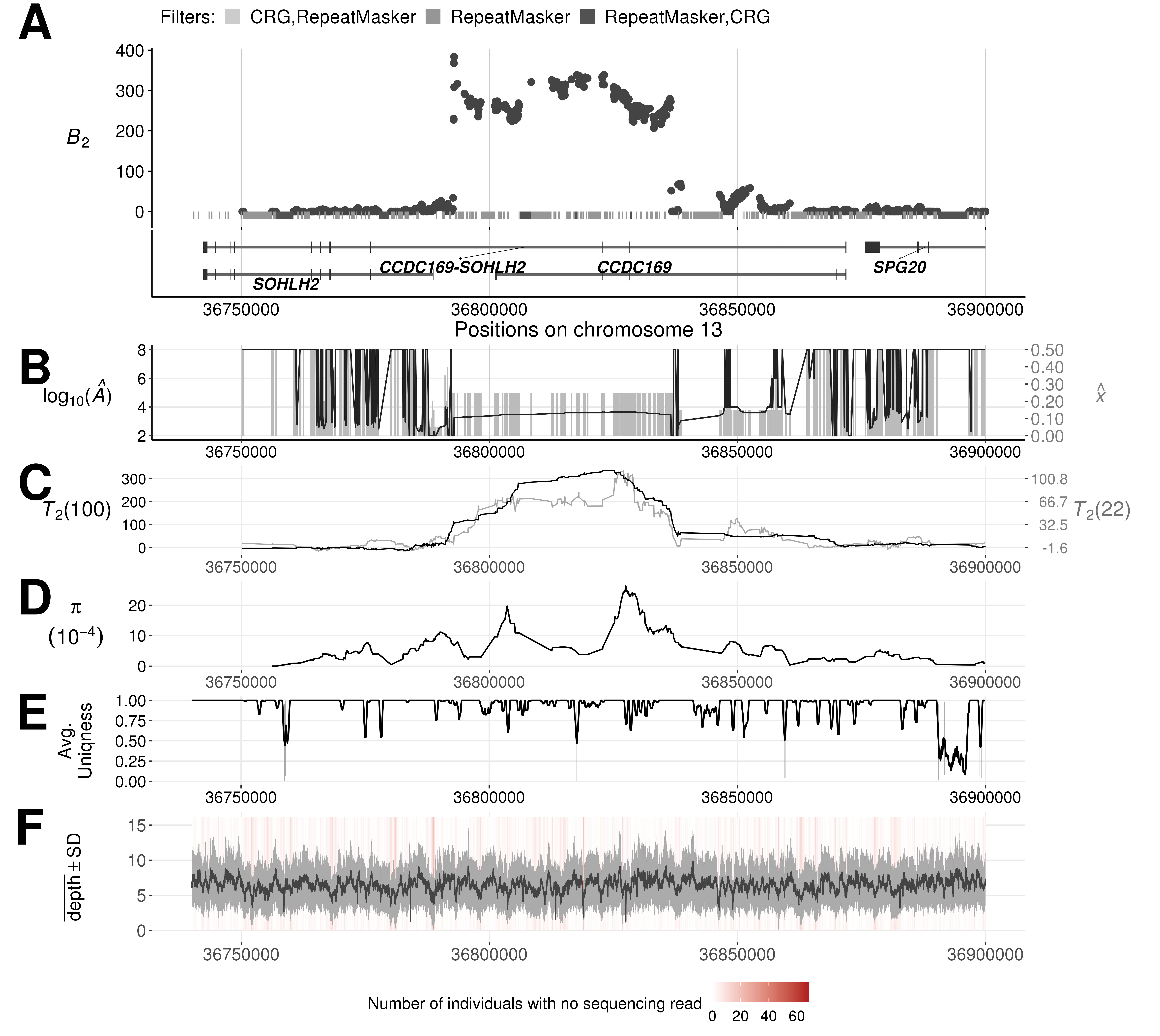

Supplement: msaa134_supplementary_data [file msaa134_supplementary_data.zip › BallerMix_final/figures/FigS34_YRI_B2_alpha1e-8_CCDC169_Chr13_367-369e5_LR-fancyGene-aD_T2+pi+avgUniq+Depth.png]

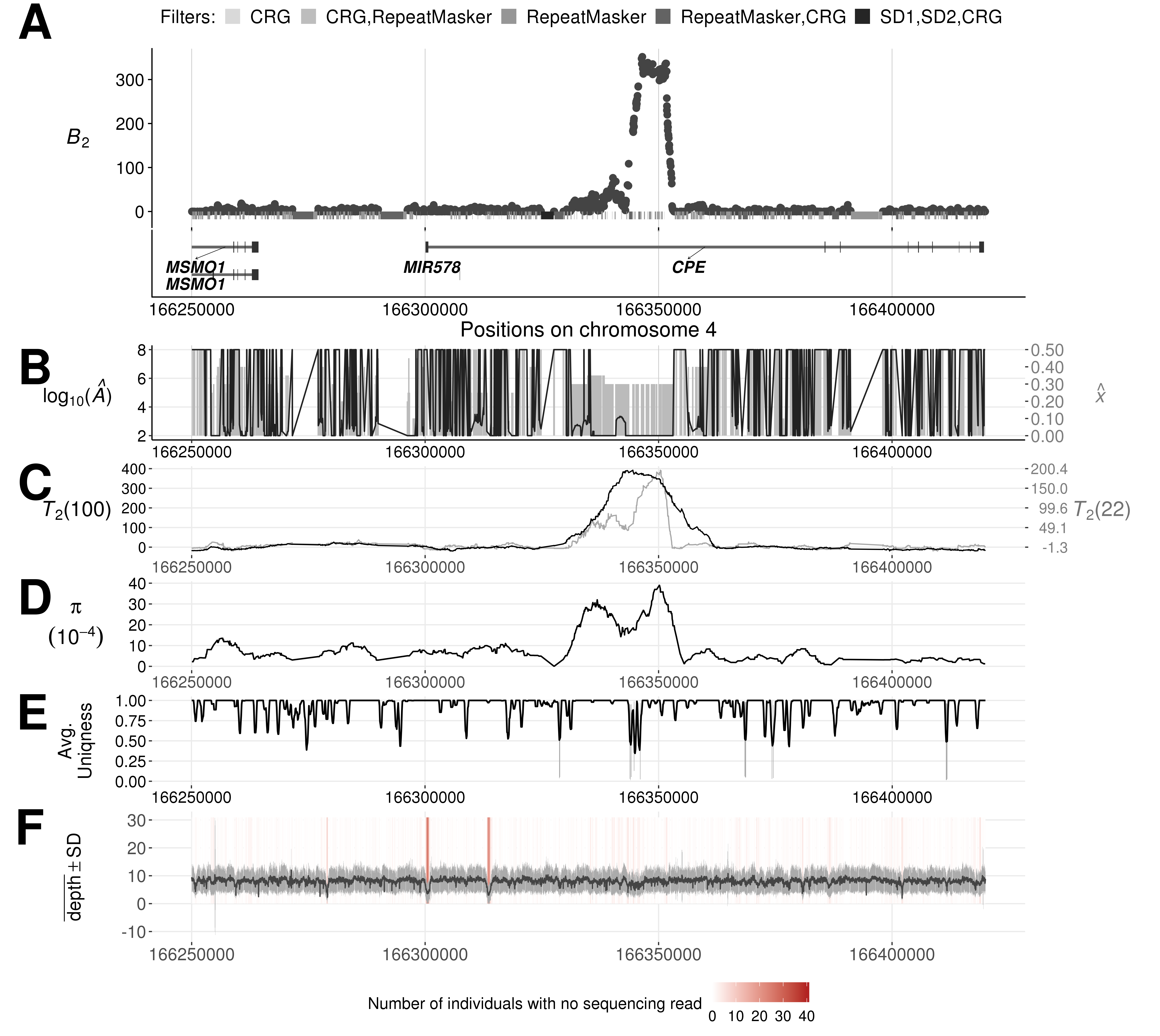

Supplement: msaa134_supplementary_data [file msaa134_supplementary_data.zip › BallerMix_final/figures/FigS35_CEU_B2_alpha1e-8_CPE_Chr4_1662-1664e5_LR-fancyGene-aD_T2+pi+avgUniq+Depth.png]

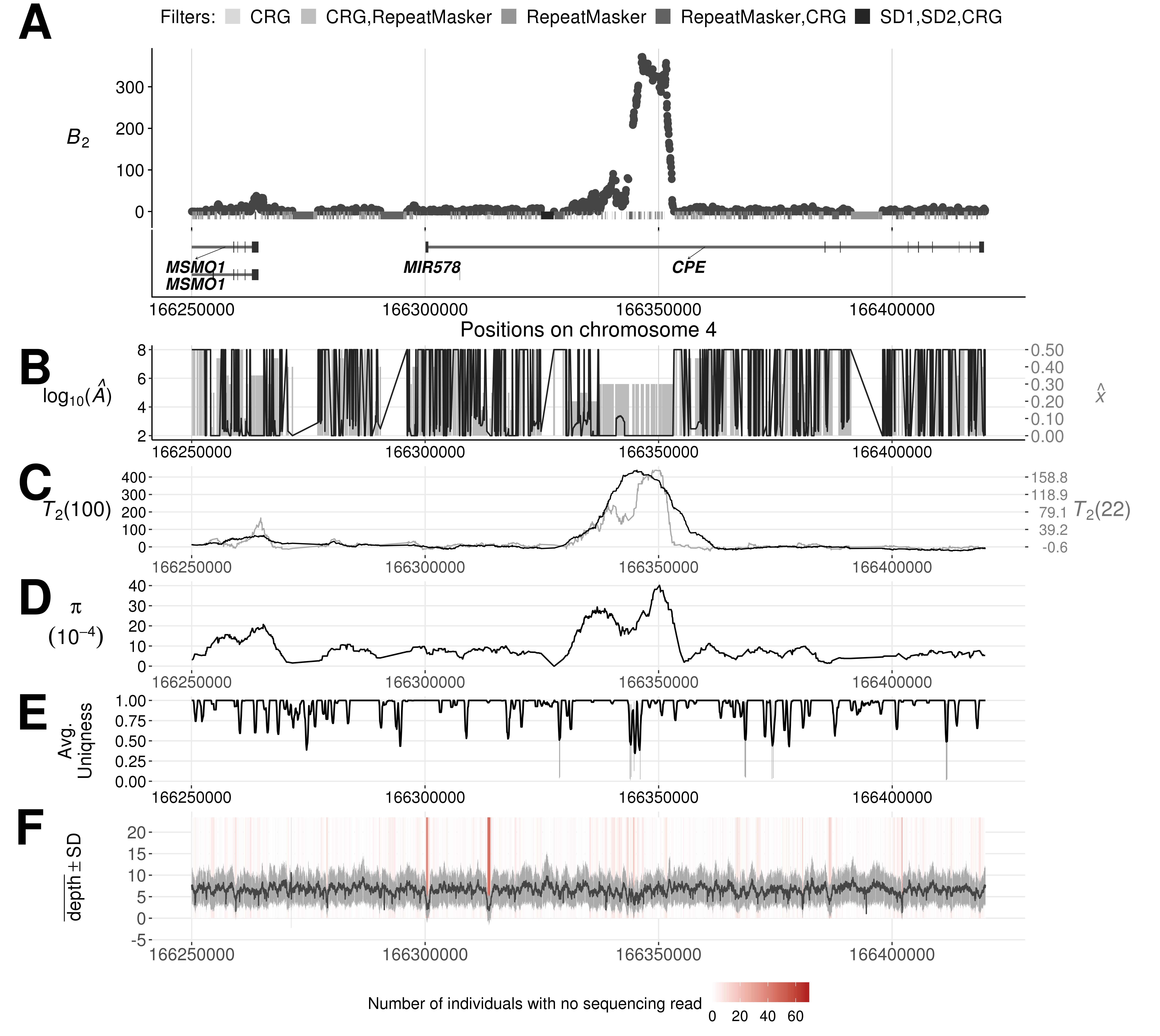

Supplement: msaa134_supplementary_data [file msaa134_supplementary_data.zip › BallerMix_final/figures/FigS36_YRI_B2_alpha1e-8_CPE_Chr4_1662-1664e5_LR-fancyGene-aD_T2+pi+avgUniq+Depth.png]

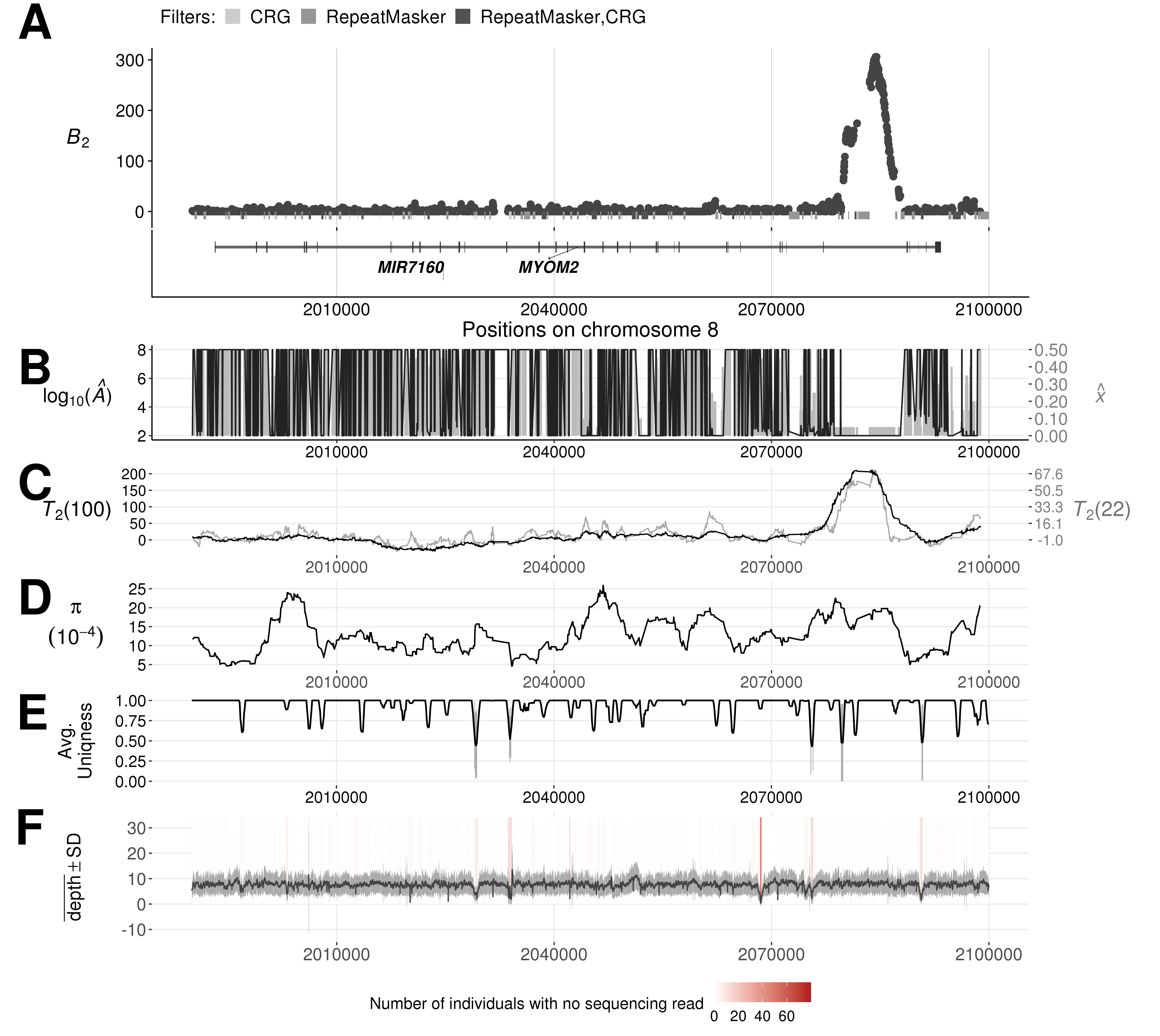

Supplement: msaa134_supplementary_data [file msaa134_supplementary_data.zip › BallerMix_final/figures/FigS37_CEU_B2_alpha1e-8_MYOM2_Chr8_20-21e5_LR-fancyGene-aD_T2+pi+avgUniq+Depth.png]

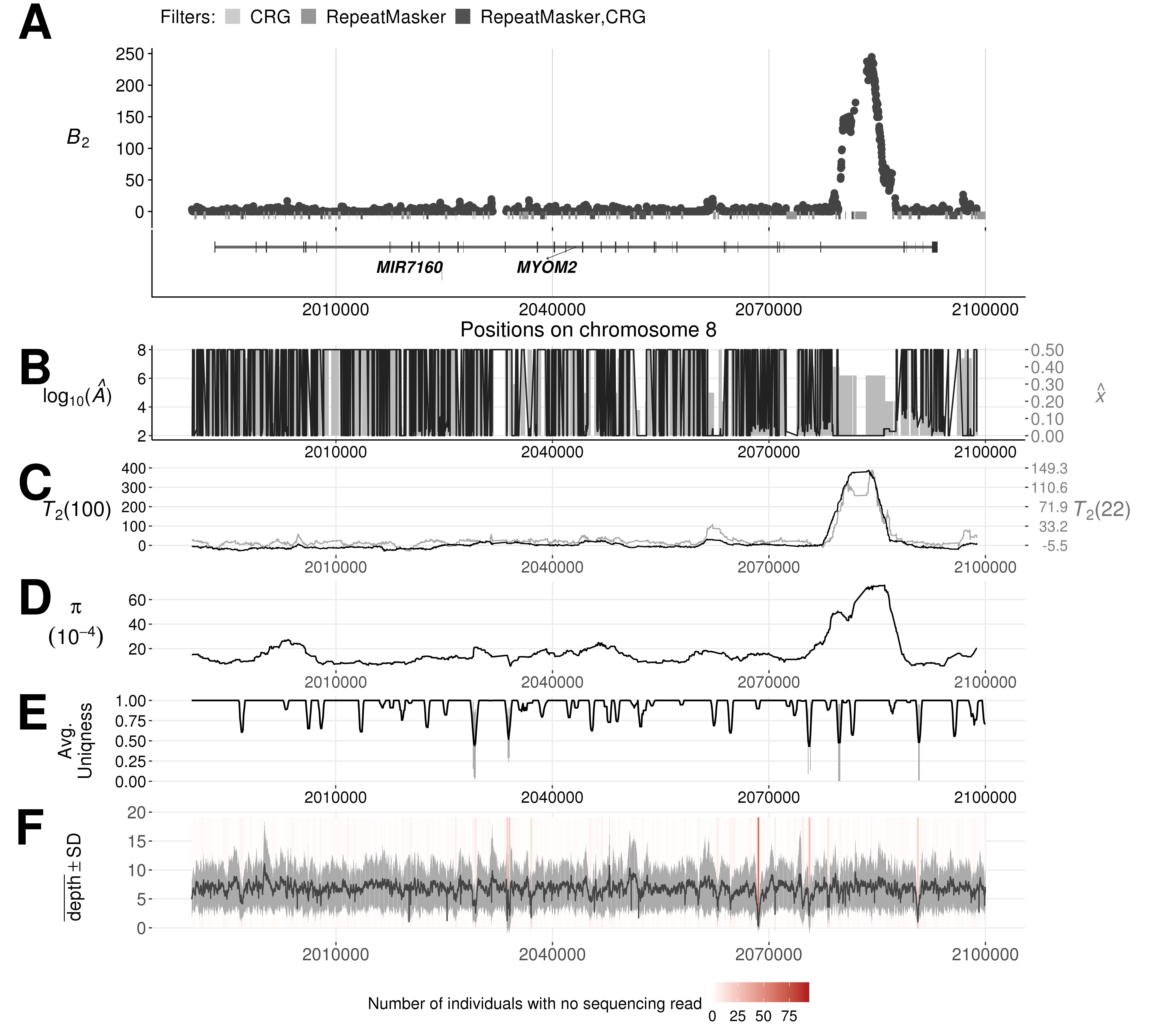

Supplement: msaa134_supplementary_data [file msaa134_supplementary_data.zip › BallerMix_final/figures/FigS38_YRI_B2_alpha1e-8_MYOM2_Chr8_20-21e5_LR-fancyGene-aD_T2+pi+avgUniq+Depth.png]

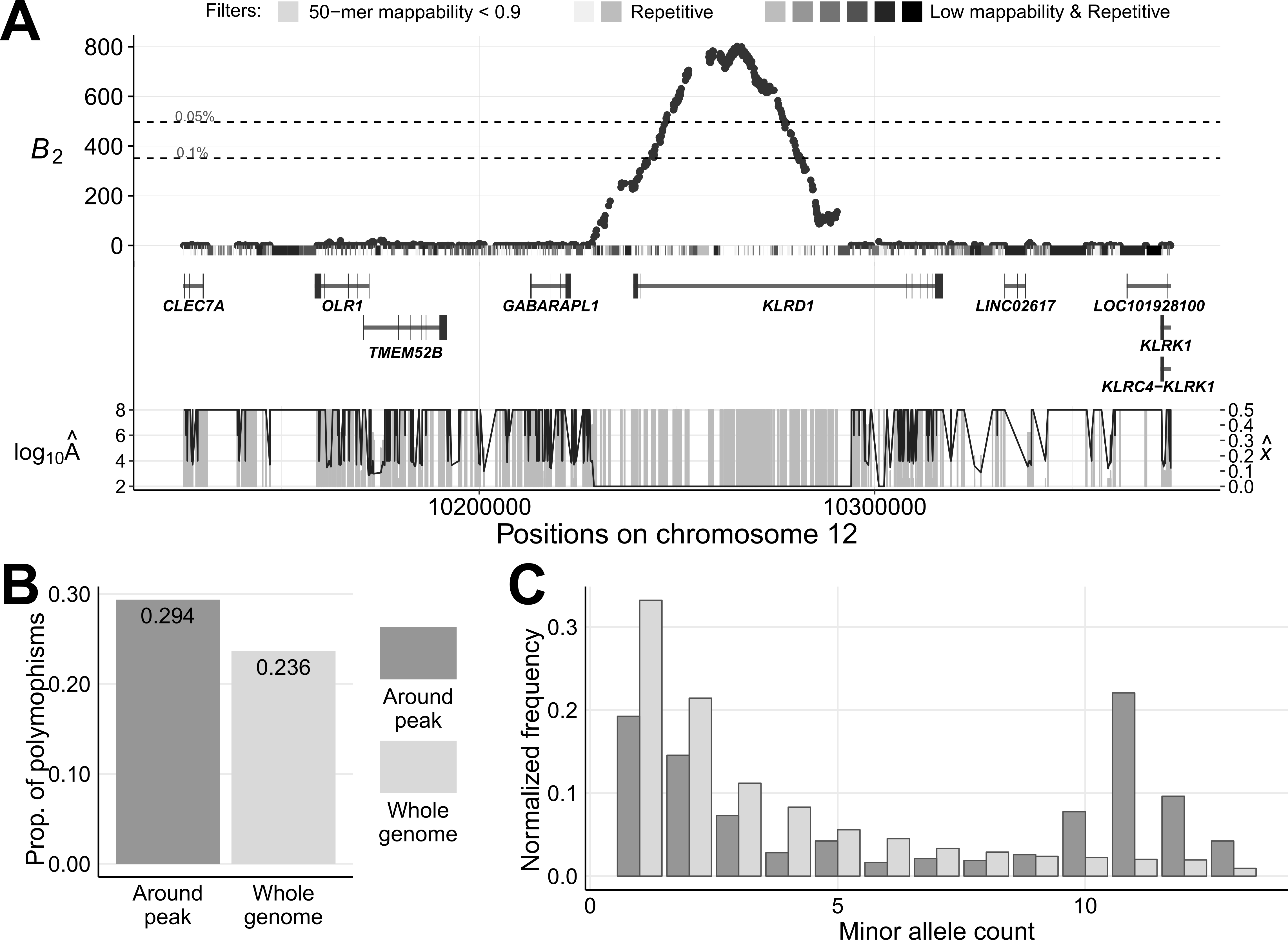

Supplement: msaa134_supplementary_data [file msaa134_supplementary_data.zip › BallerMix_final/figures/FigS39_hg38_B2_alpha1e-8_Chr12-KLRD1_250kb_LR-fancyGene-xA_spect.png]

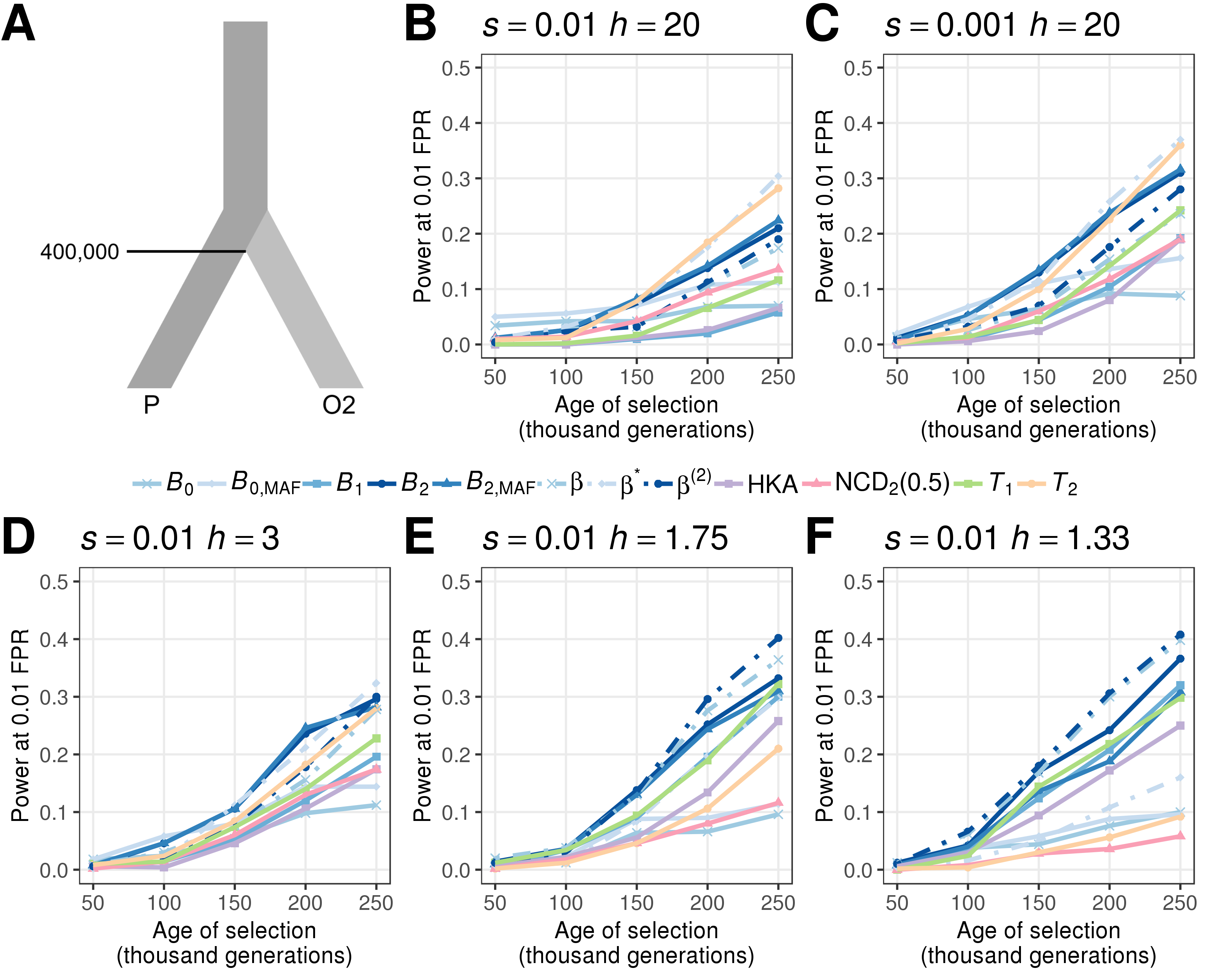

Supplement: msaa134_supplementary_data [file msaa134_supplementary_data.zip › BallerMix_final/figures/FigS4_Time-power_1-5mya_bimodalBs_v_T12+stats_6panel.png]

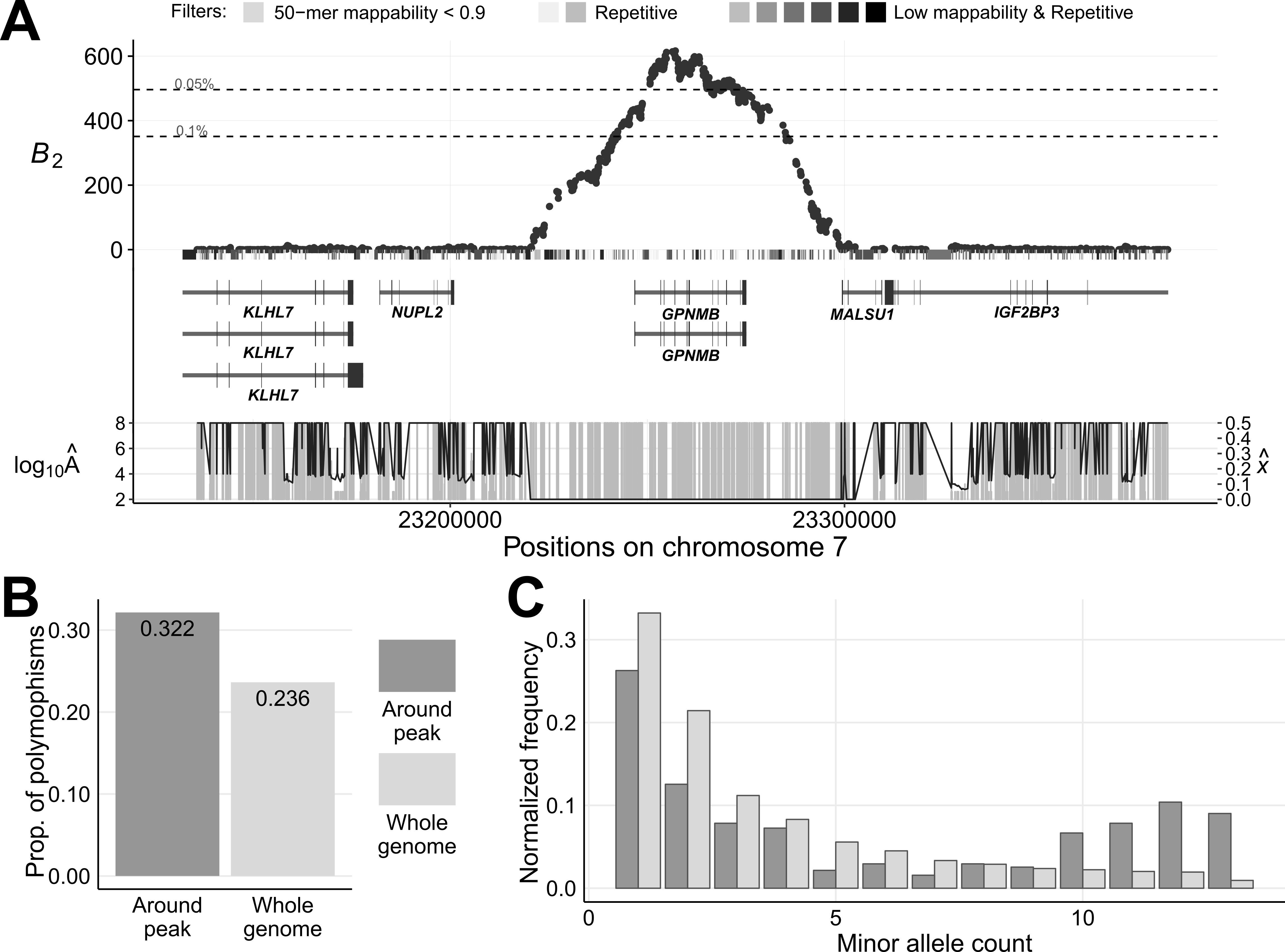

Supplement: msaa134_supplementary_data [file msaa134_supplementary_data.zip › BallerMix_final/figures/FigS40_hg38_B2_alpha1e-8_Chr7-GPNMB_250kb_LR-fancyGene-xA_spect.png]

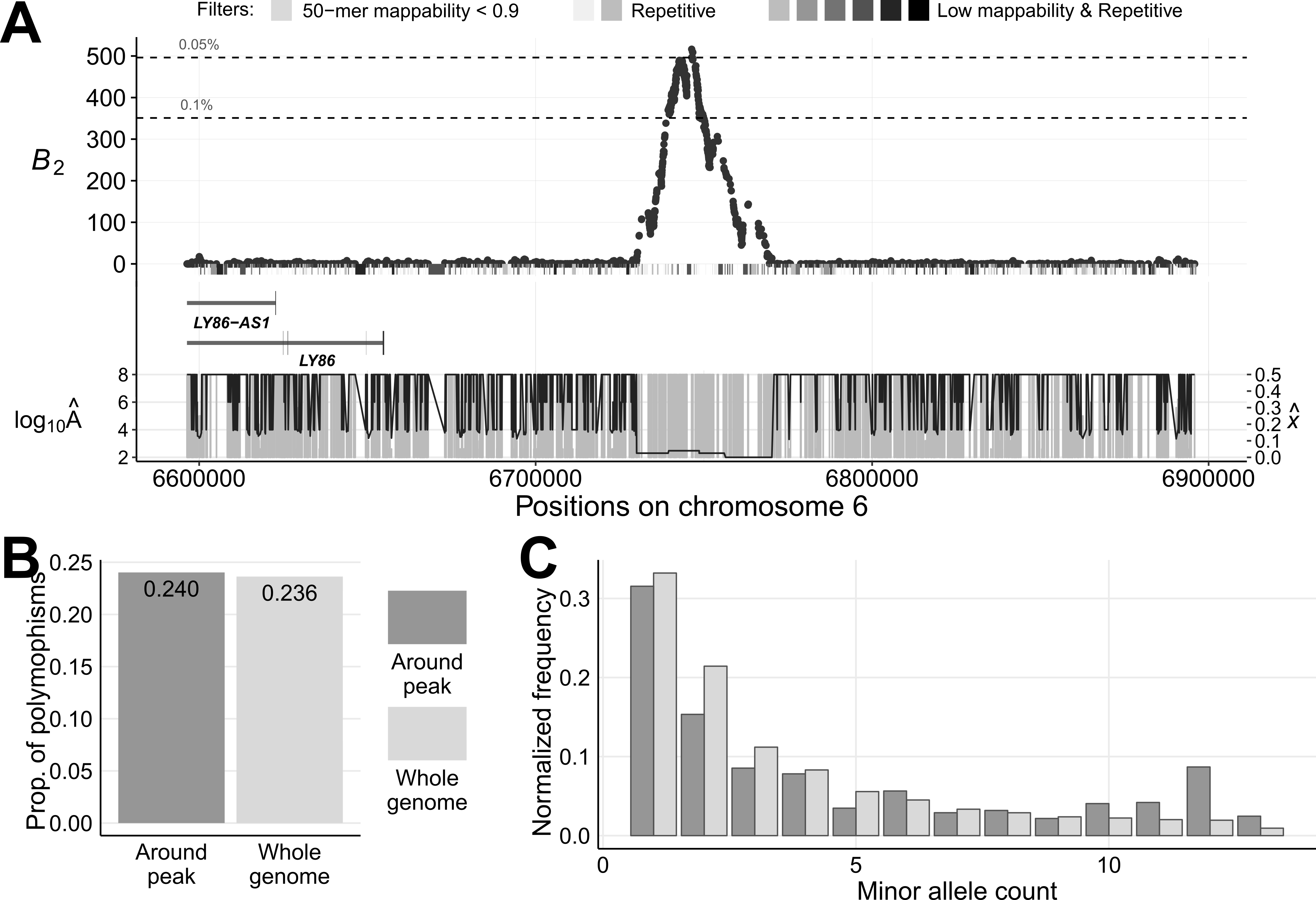

Supplement: msaa134_supplementary_data [file msaa134_supplementary_data.zip › BallerMix_final/figures/FigS41_hg38_B2_alpha1e-8_Chr6-LY86_250kb_LR-fancyGene-xA_spect.png]

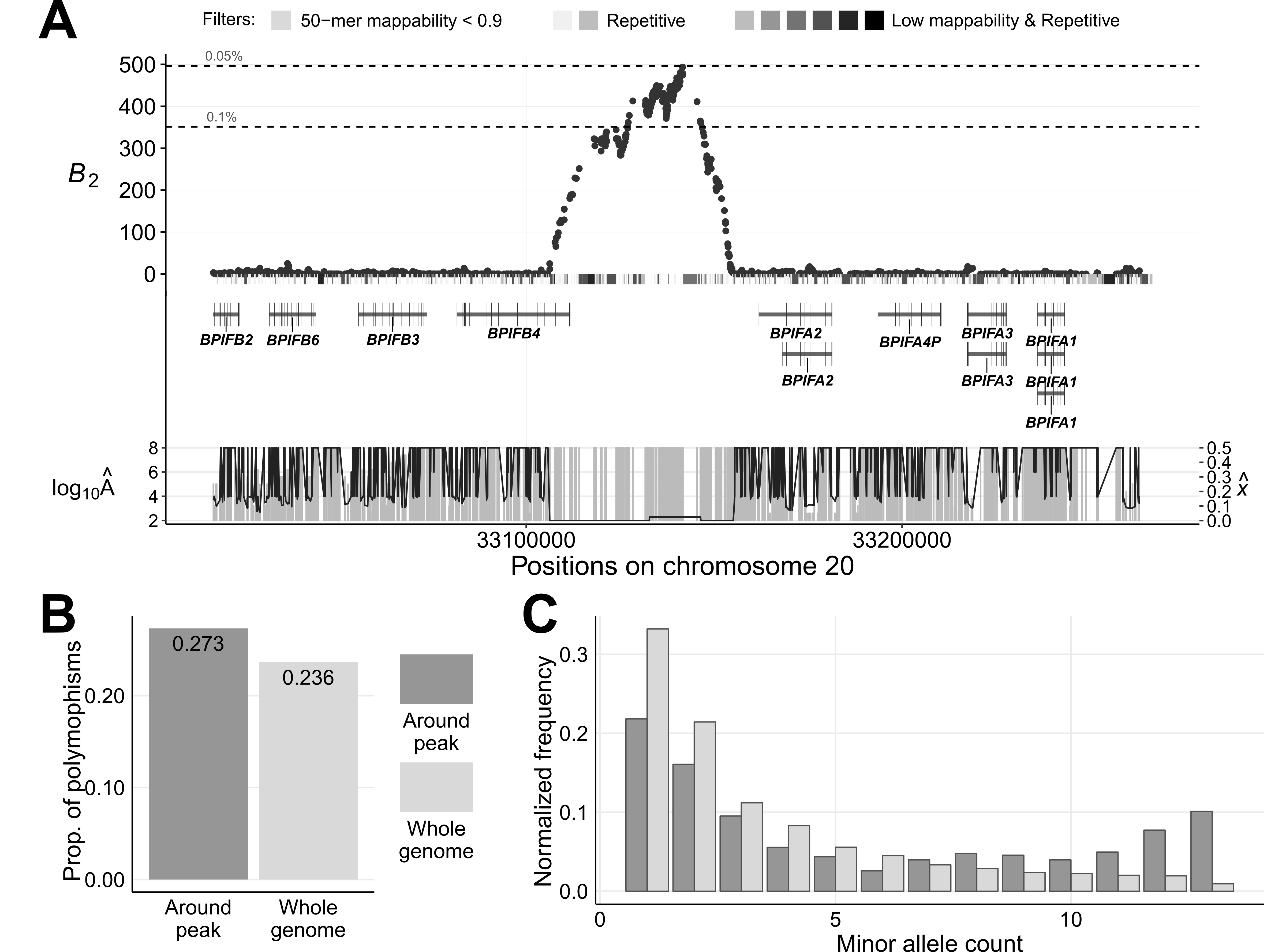

Supplement: msaa134_supplementary_data [file msaa134_supplementary_data.zip › BallerMix_final/figures/FigS42_hg38_B2_alpha1e-8_Ch20-BPIF_250kb_LR-fancyGene-xA_spect.png]

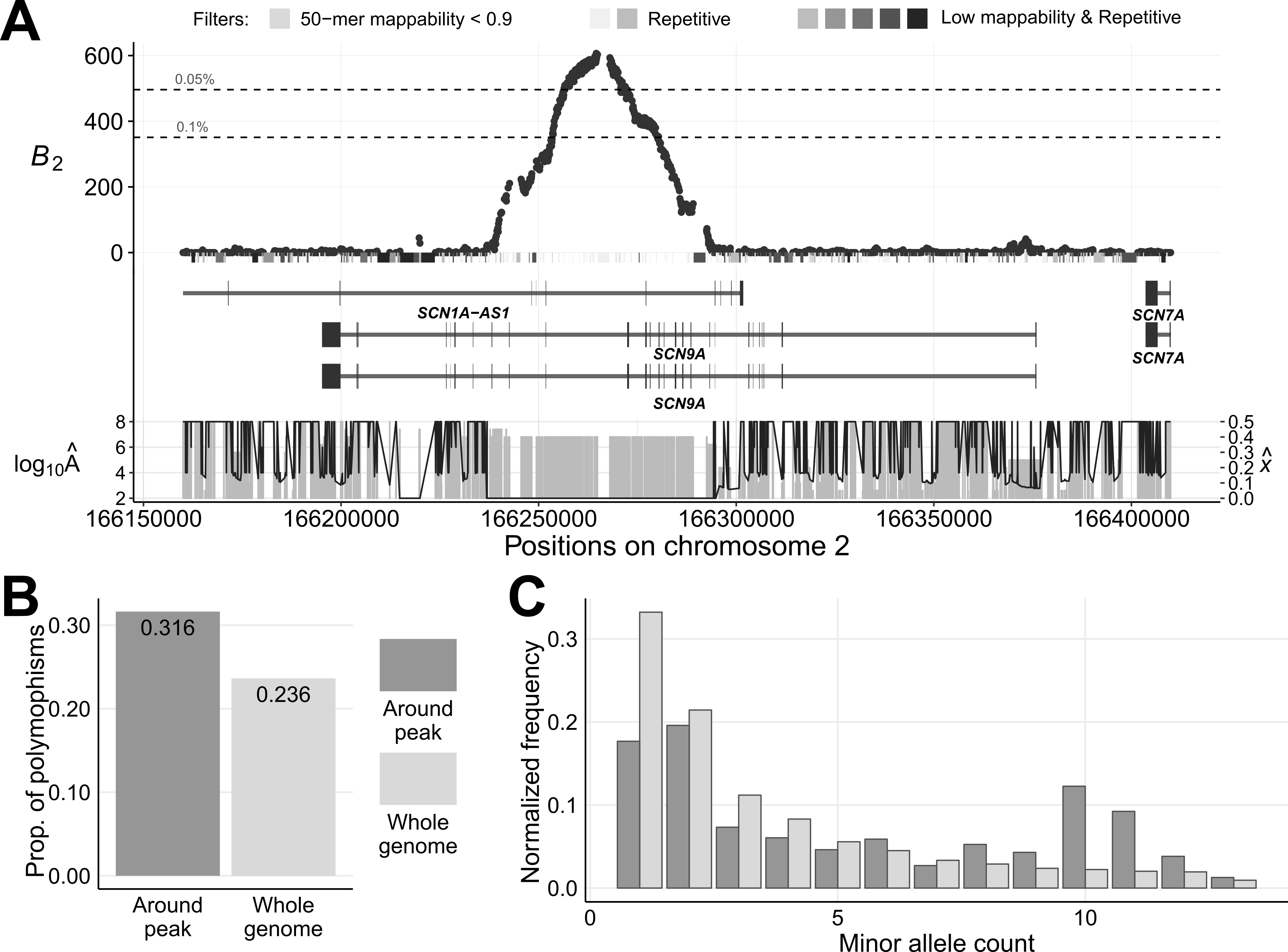

Supplement: msaa134_supplementary_data [file msaa134_supplementary_data.zip › BallerMix_final/figures/FigS43_hg38_B2_alpha1e-8_Ch2-SCN9A_250kb_LR-fancyGene-xA_spect.png]

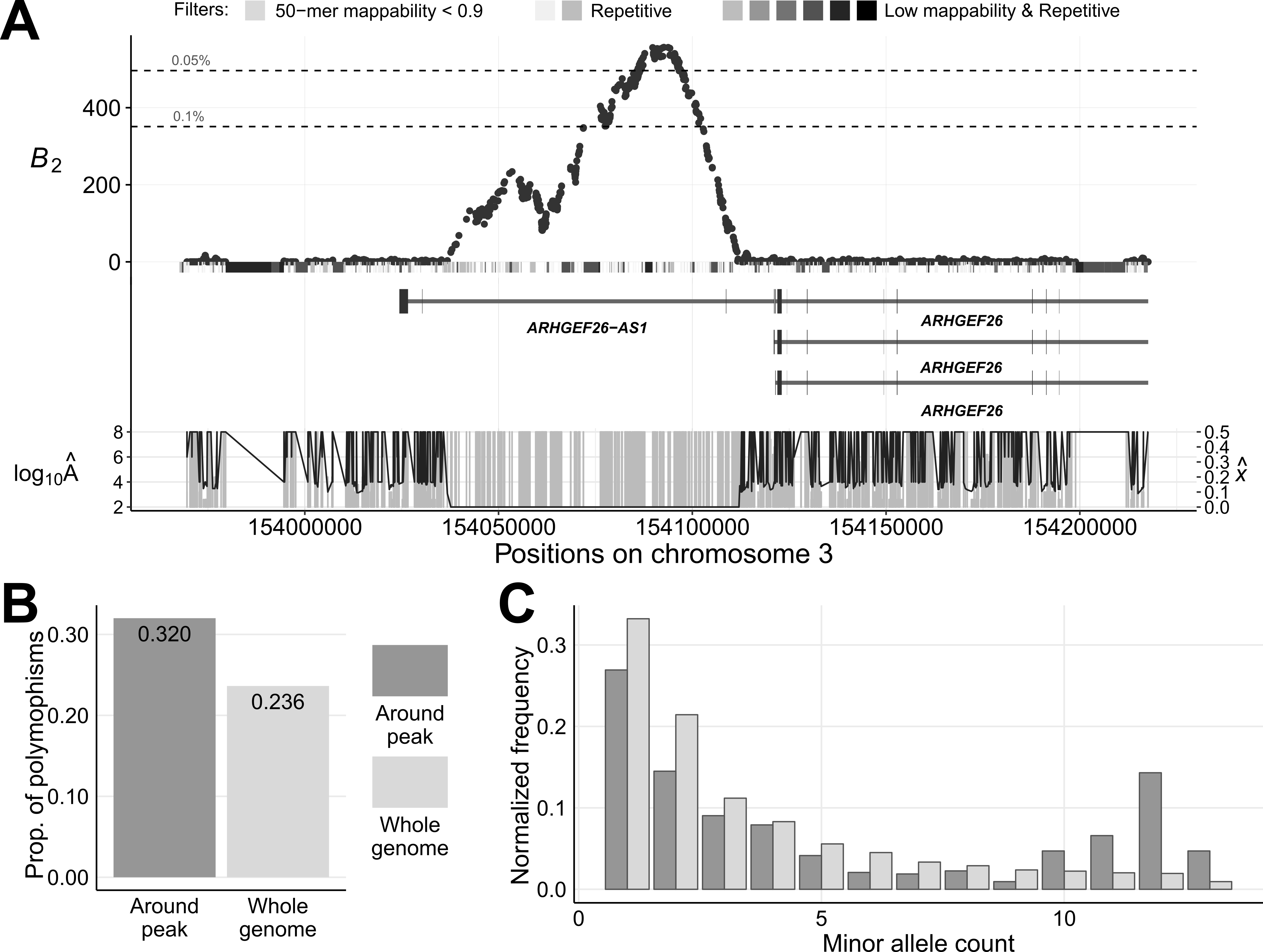

Supplement: msaa134_supplementary_data [file msaa134_supplementary_data.zip › BallerMix_final/figures/FigS44_hg38_B2_alpha1e-8_Ch3-ARHGEF26_250kb_LR-fancyGene-xA_spect.png]

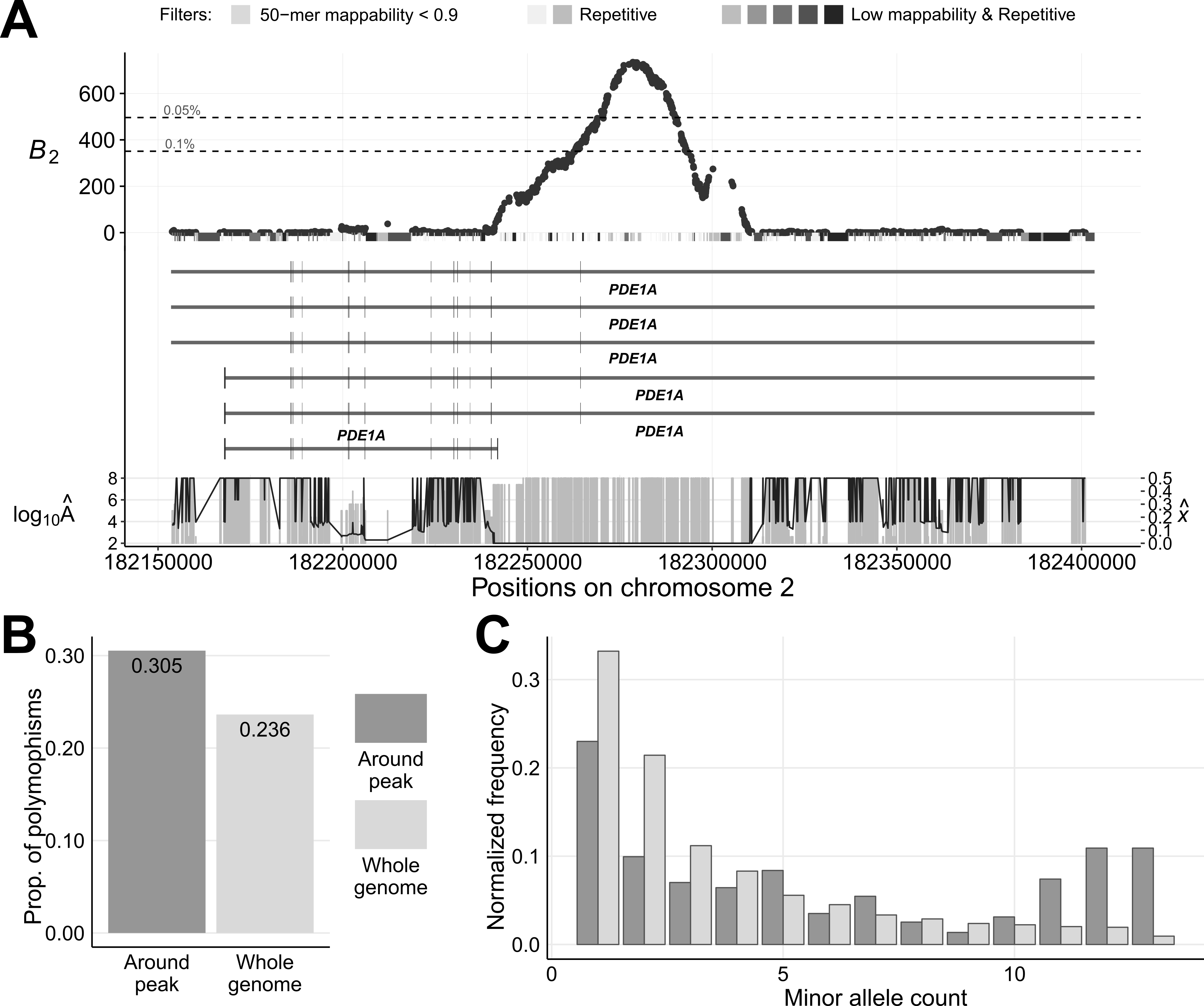

Supplement: msaa134_supplementary_data [file msaa134_supplementary_data.zip › BallerMix_final/figures/FigS45_hg38_B2_Chr2-PDE1A_250kb_LR-fancyGene-xA_spect.png]

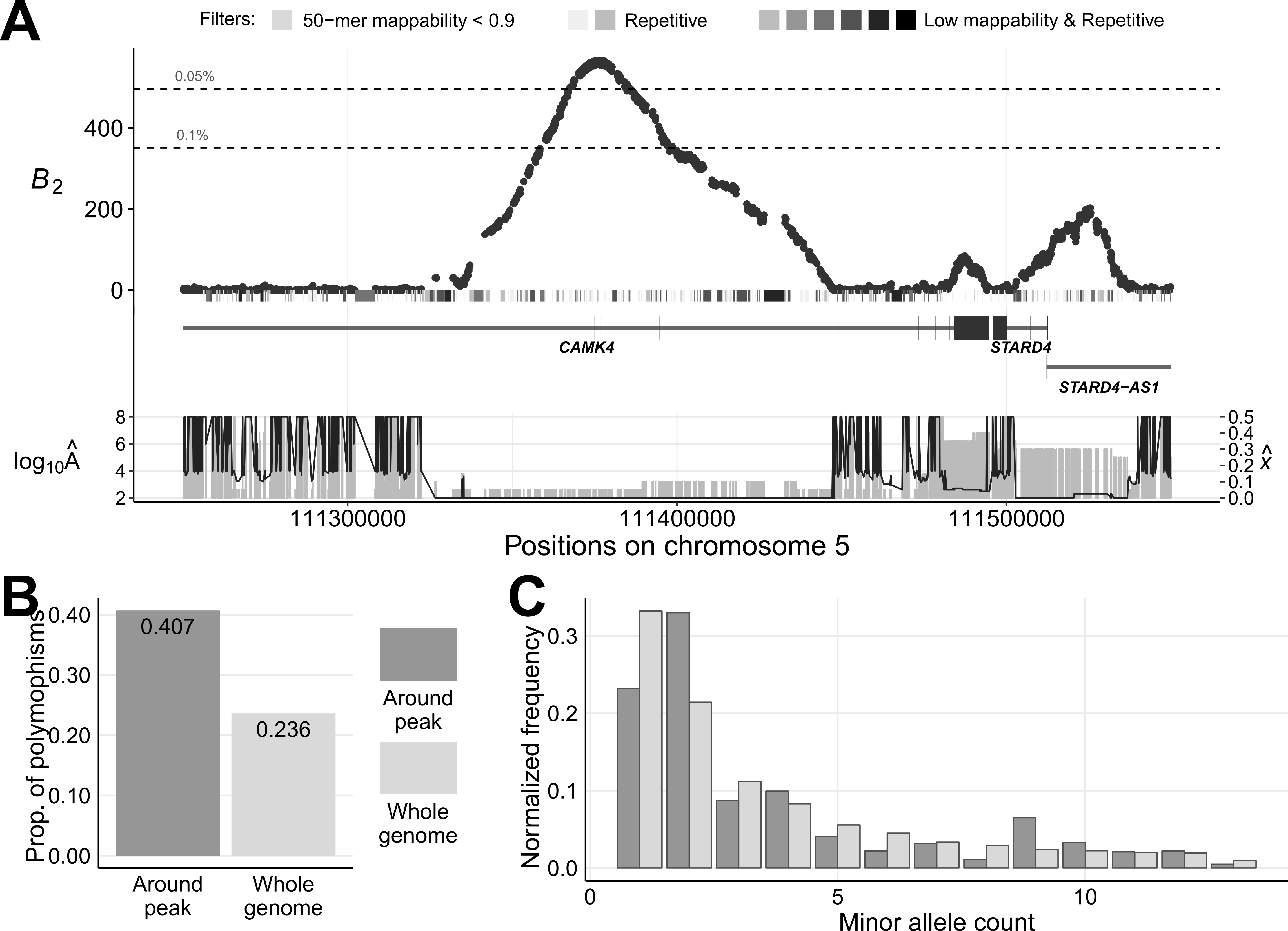

Supplement: msaa134_supplementary_data [file msaa134_supplementary_data.zip › BallerMix_final/figures/FigS46_hg38_B2_alpha1e-8_Ch5-CAMK4_250kb_LR-fancyGene-xA_spect.png]

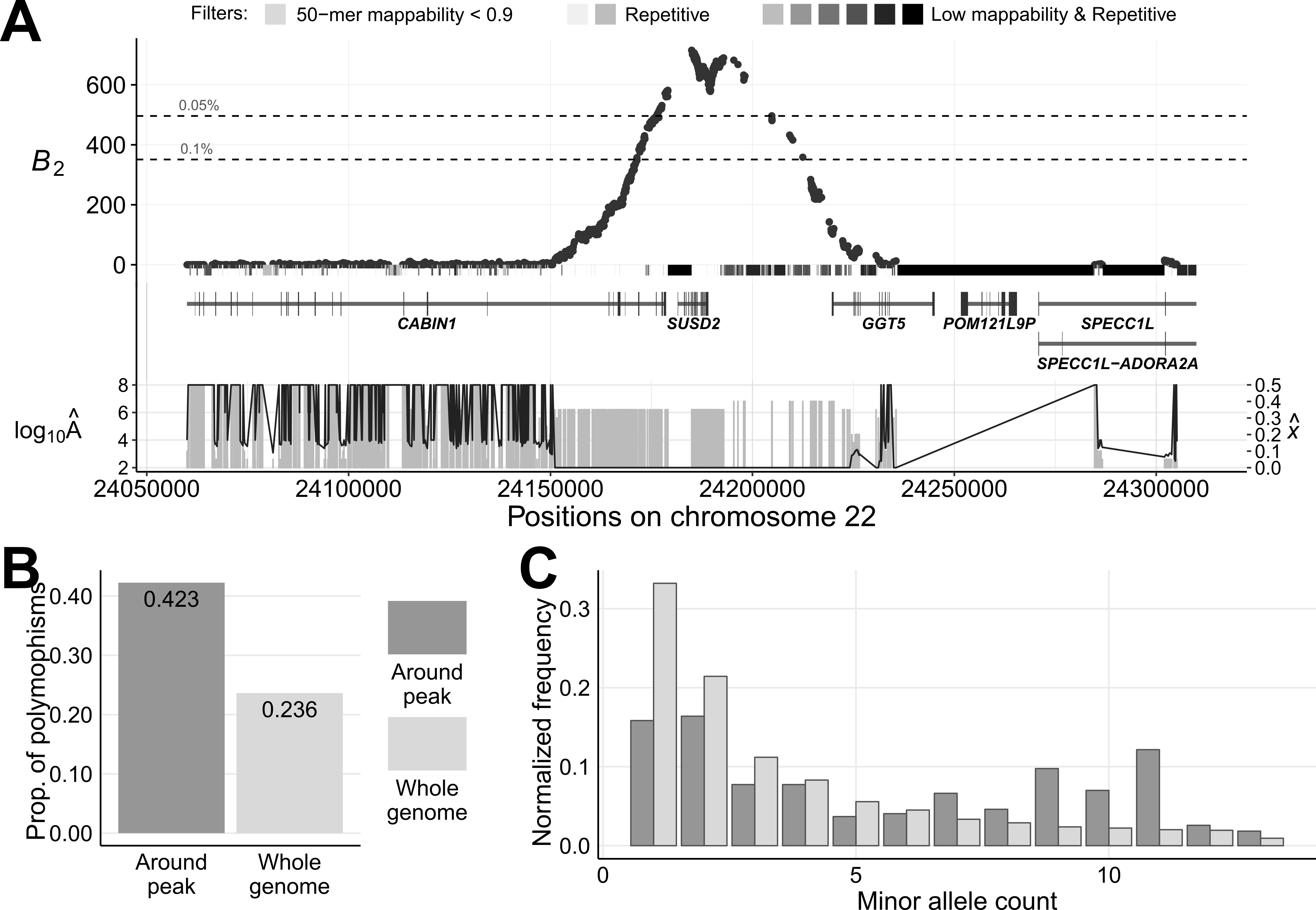

Supplement: msaa134_supplementary_data [file msaa134_supplementary_data.zip › BallerMix_final/figures/FigS47_hg38_B2_alpha1e-8_Ch22-SUSD2_250kb_LR-fancyGene-xA_spect.png]

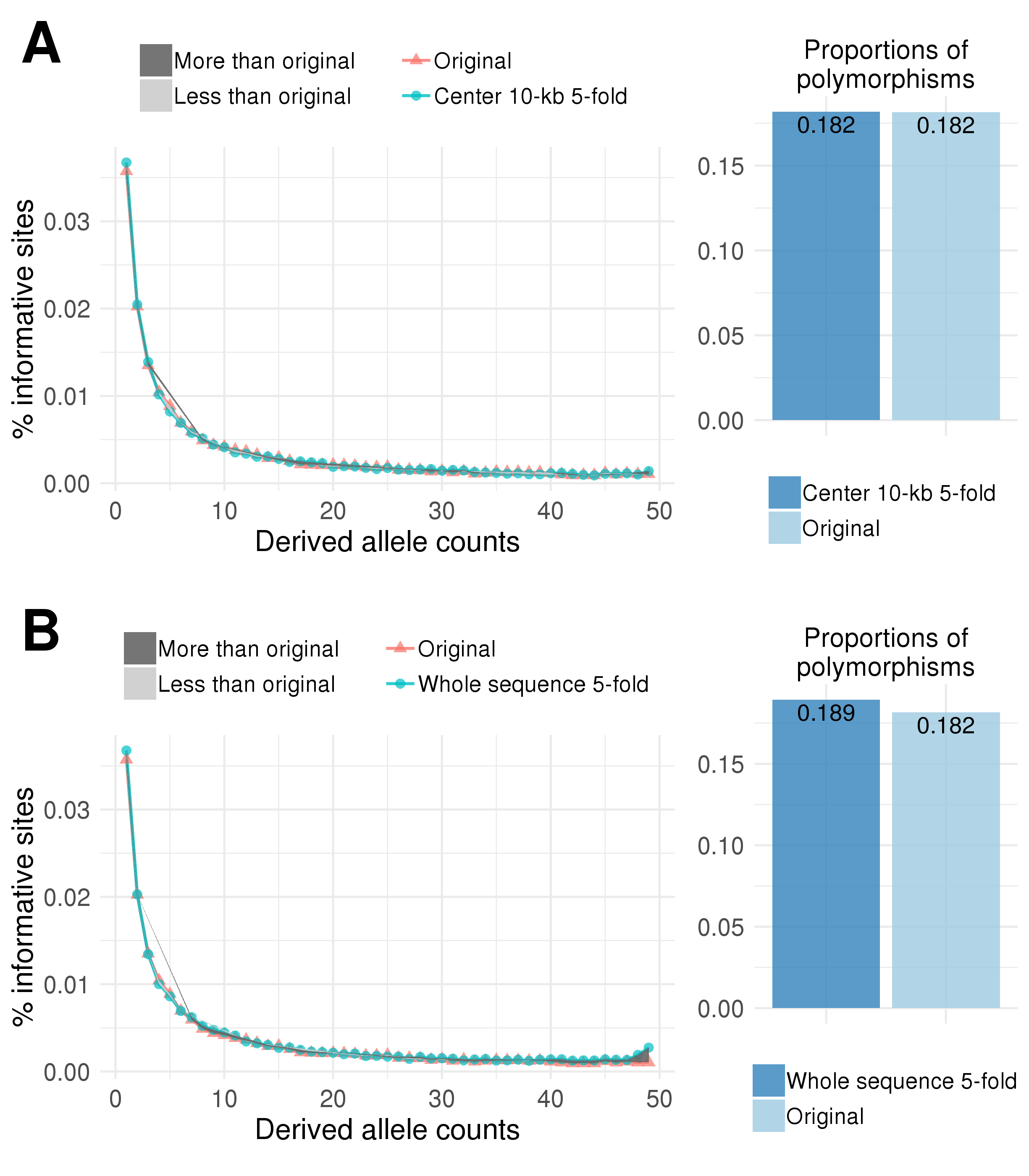

Supplement: msaa134_supplementary_data [file msaa134_supplementary_data.zip › BallerMix_final/figures/FigS48_Compare_spectra_localMut-5Mut.png]

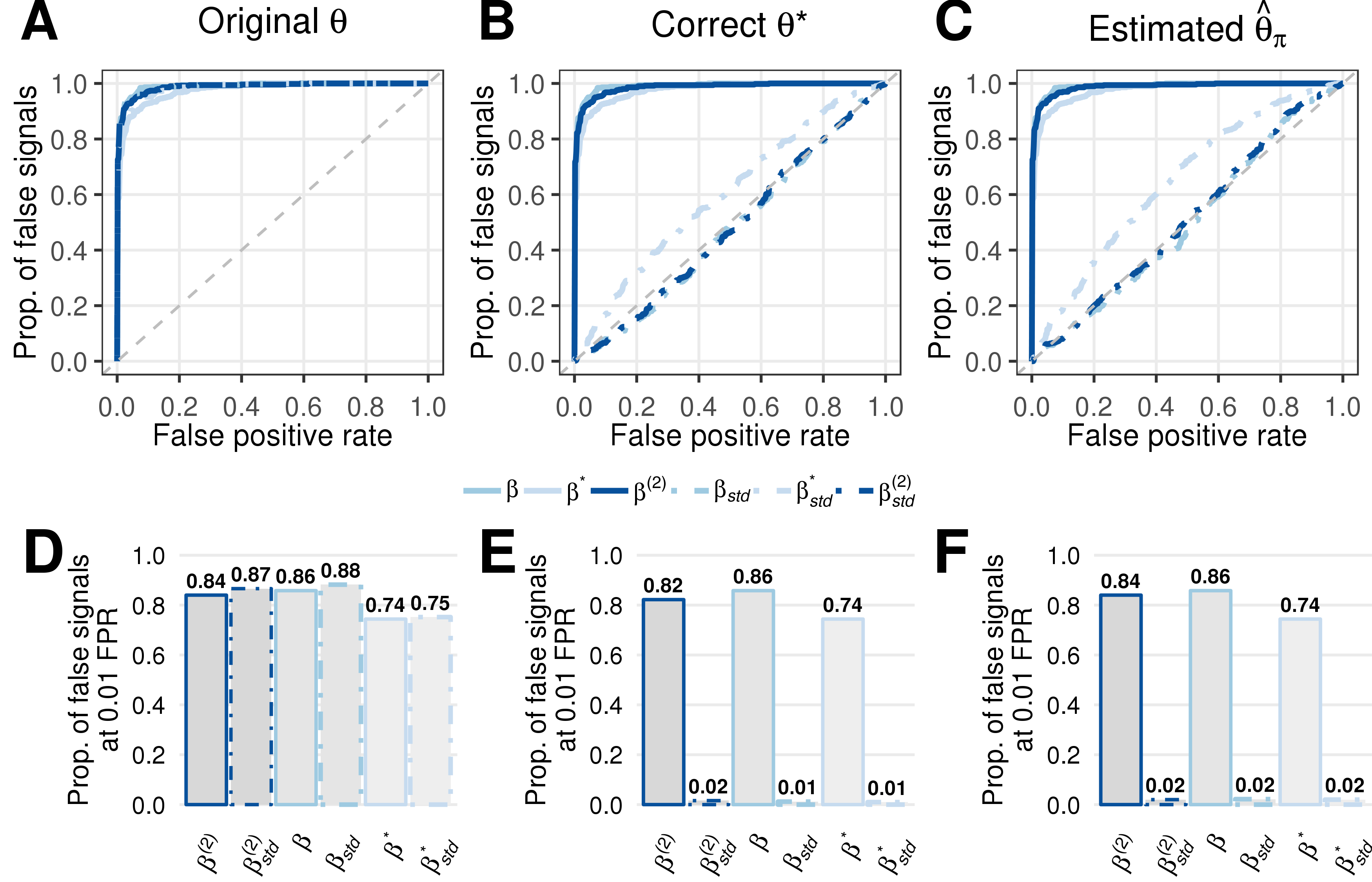

Supplement: msaa134_supplementary_data [file msaa134_supplementary_data.zip › BallerMix_final/figures/FigS49_HCG_5Mut_ROC-powerBar_5Mut_Betas-6panel.png]

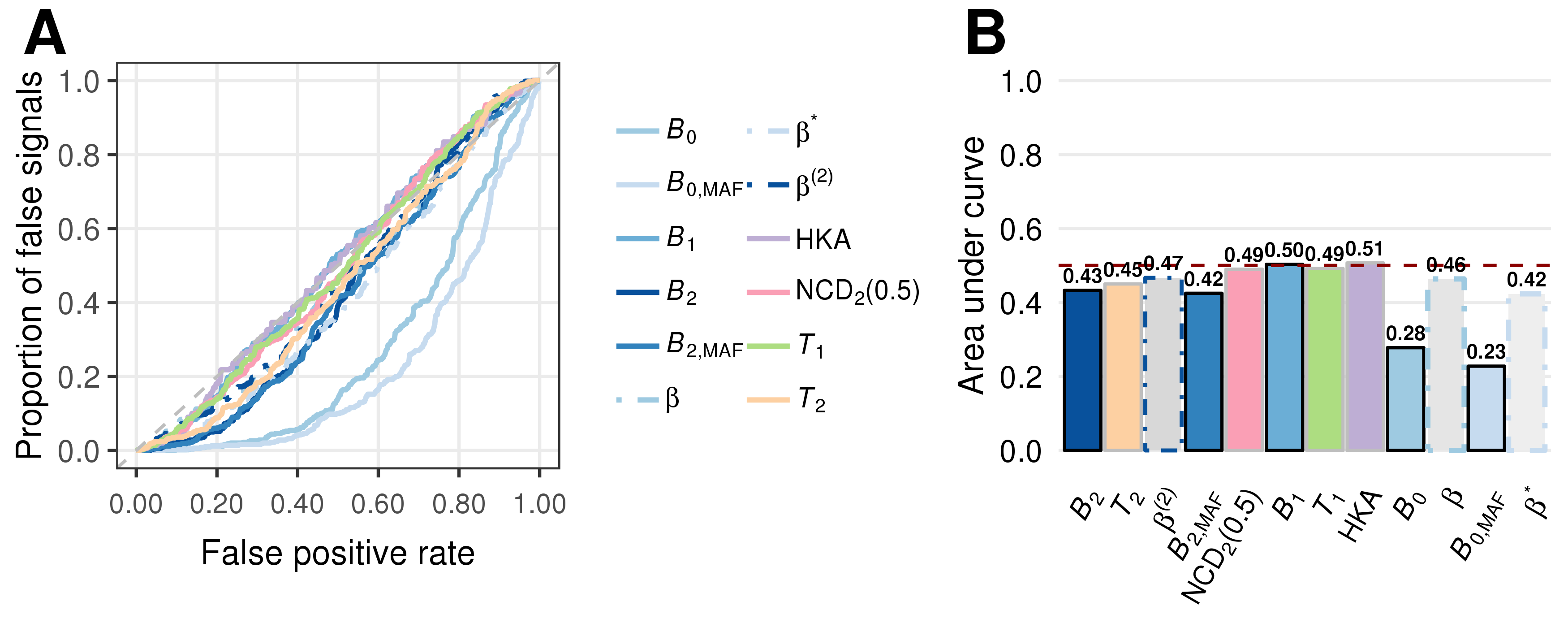

Supplement: msaa134_supplementary_data [file msaa134_supplementary_data.zip › BallerMix_final/figures/FigS5_HCG_10rec_Neut_ROC+powerbar_alphaB+stats.png]

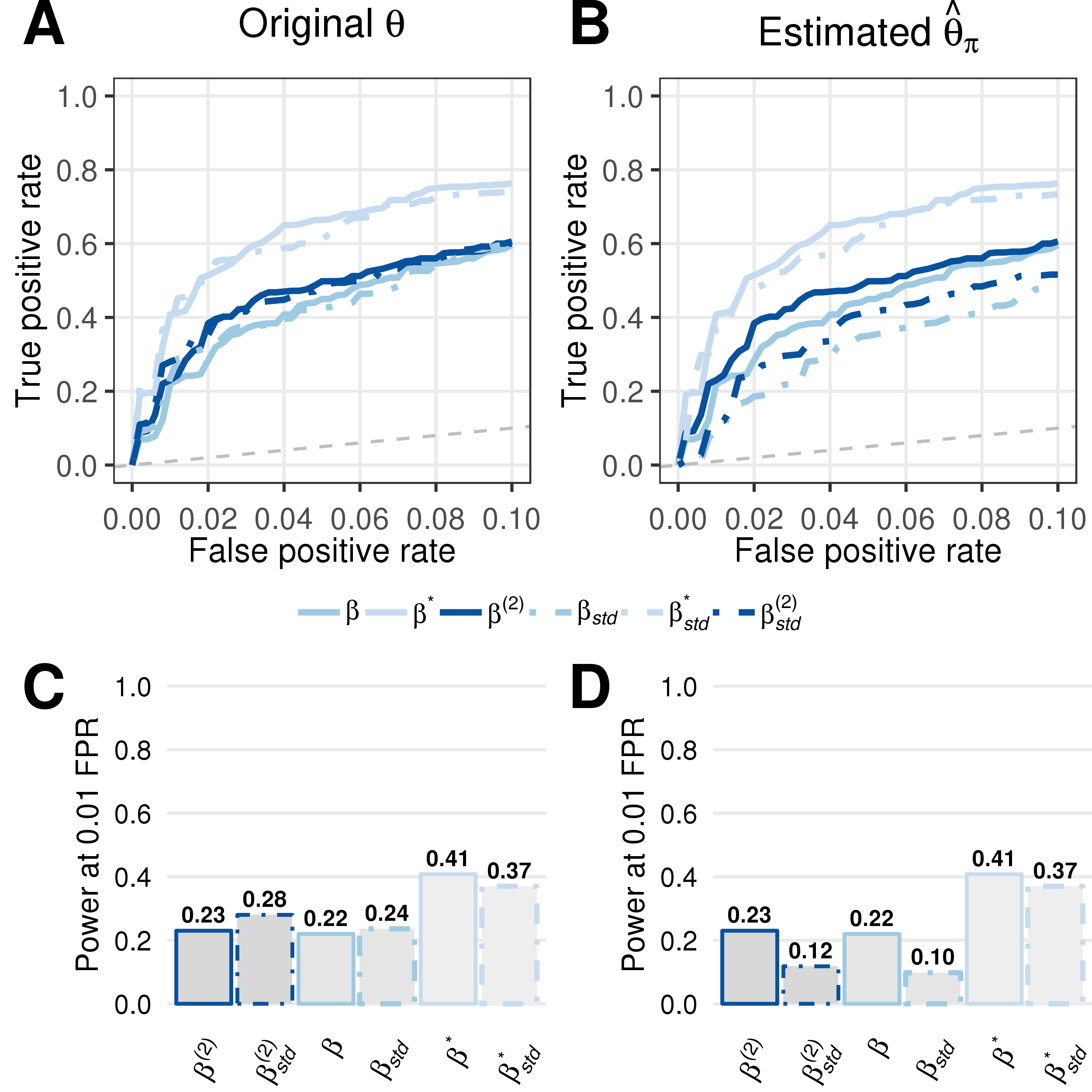

Supplement: msaa134_supplementary_data [file msaa134_supplementary_data.zip › BallerMix_final/figures/FigS50_HCG_5MYA_s001_h20_ROC-powerBar_s001h20_Betas-4panel.png]

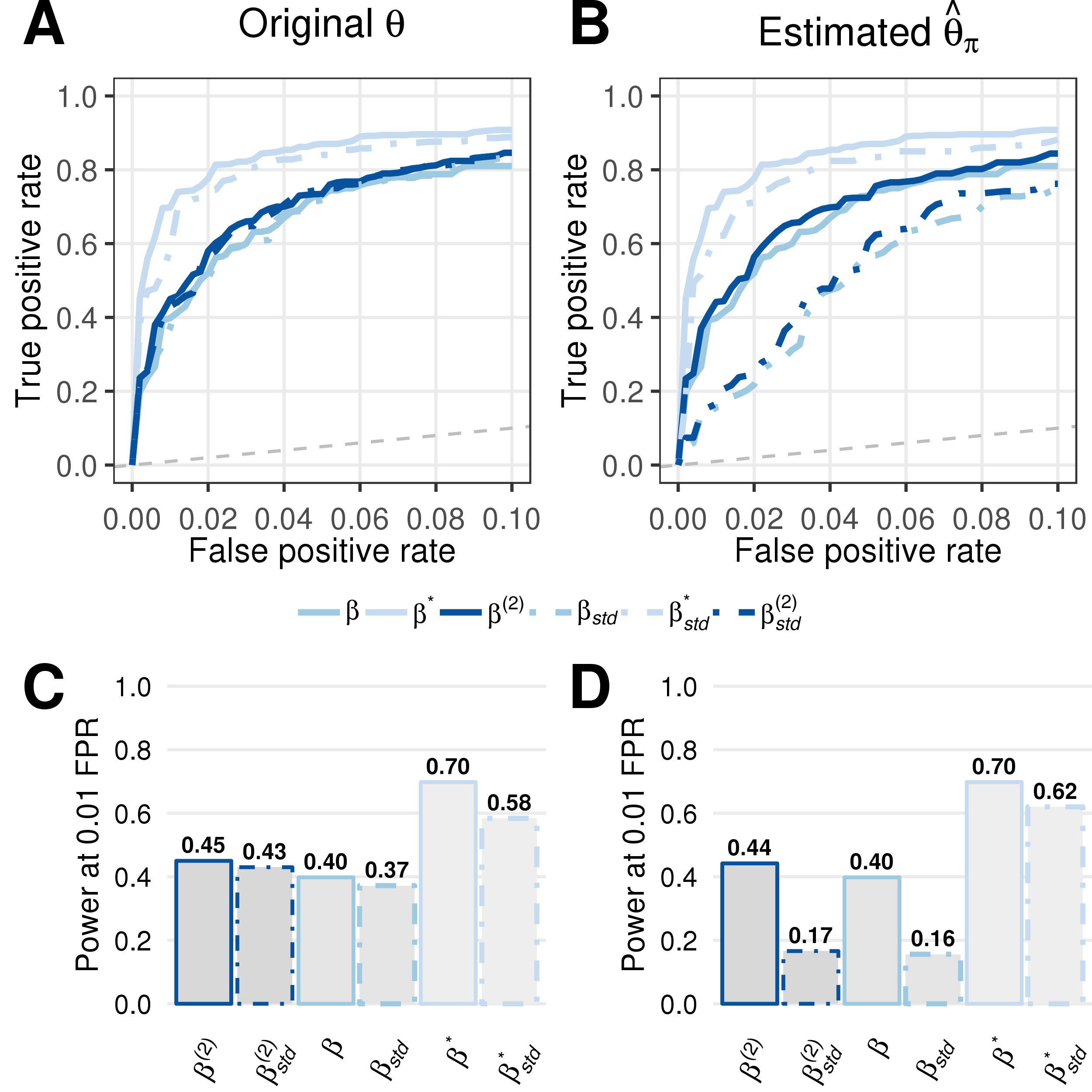

Supplement: msaa134_supplementary_data [file msaa134_supplementary_data.zip › BallerMix_final/figures/FigS51_HCG_5Mut_5MYA_s001_h20_ROC-powerBar_s001h20_Betas-4panel.png]

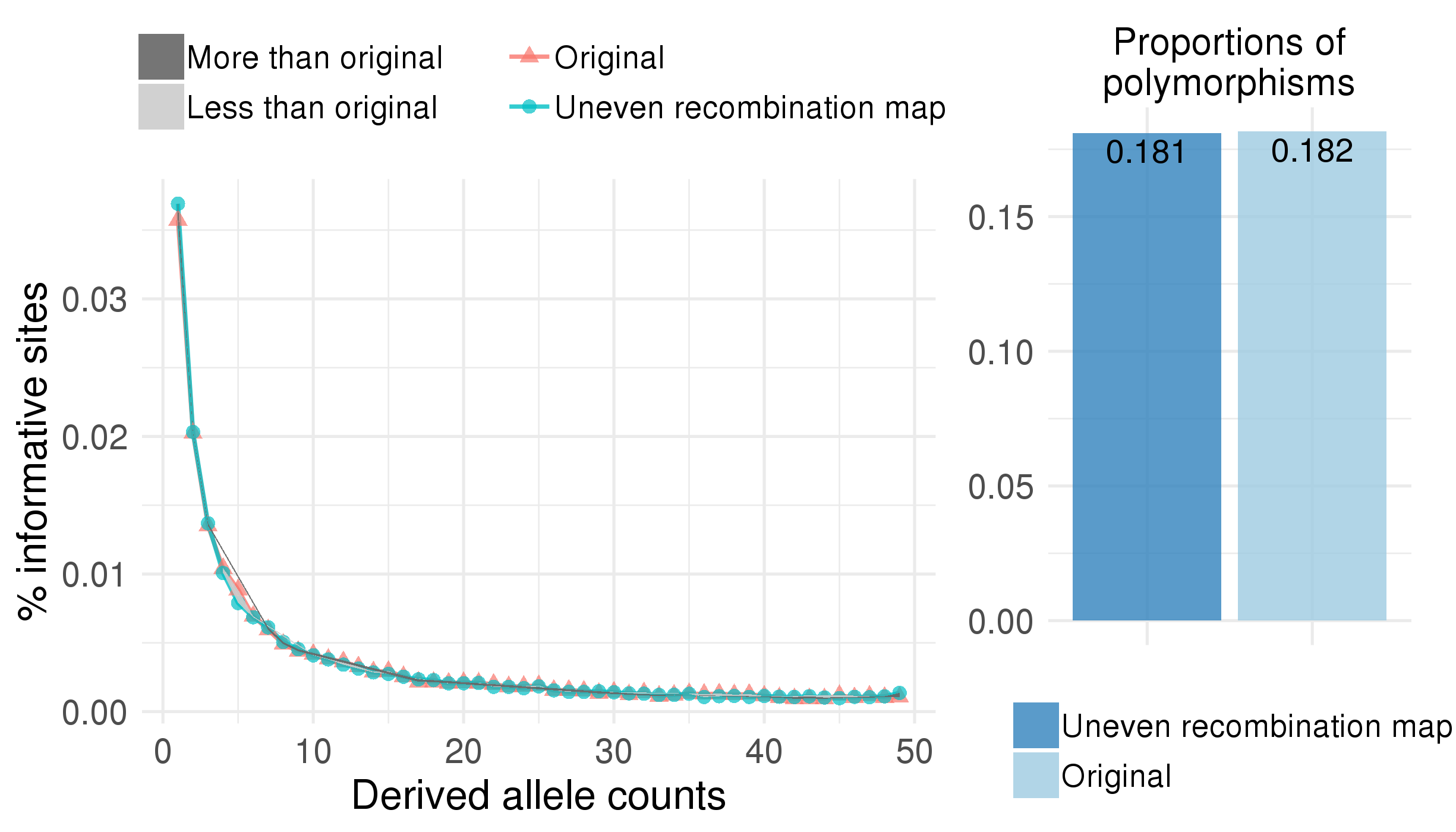

Supplement: msaa134_supplementary_data [file msaa134_supplementary_data.zip › BallerMix_final/figures/FigS52_Compare_spectra_10rec_Neut.png]

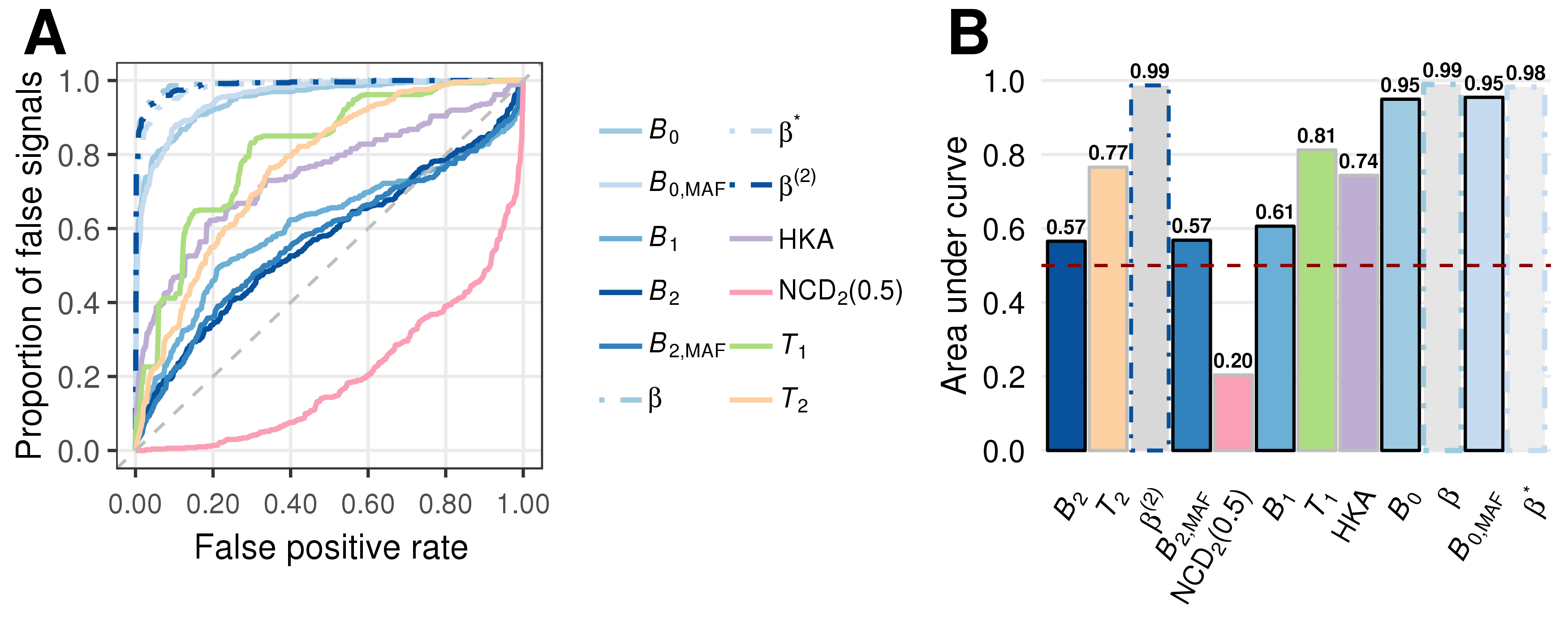

Supplement: msaa134_supplementary_data [file msaa134_supplementary_data.zip › BallerMix_final/figures/FigS53_HCG_5Mut_ROC+powerbar_matchB+stats.png]

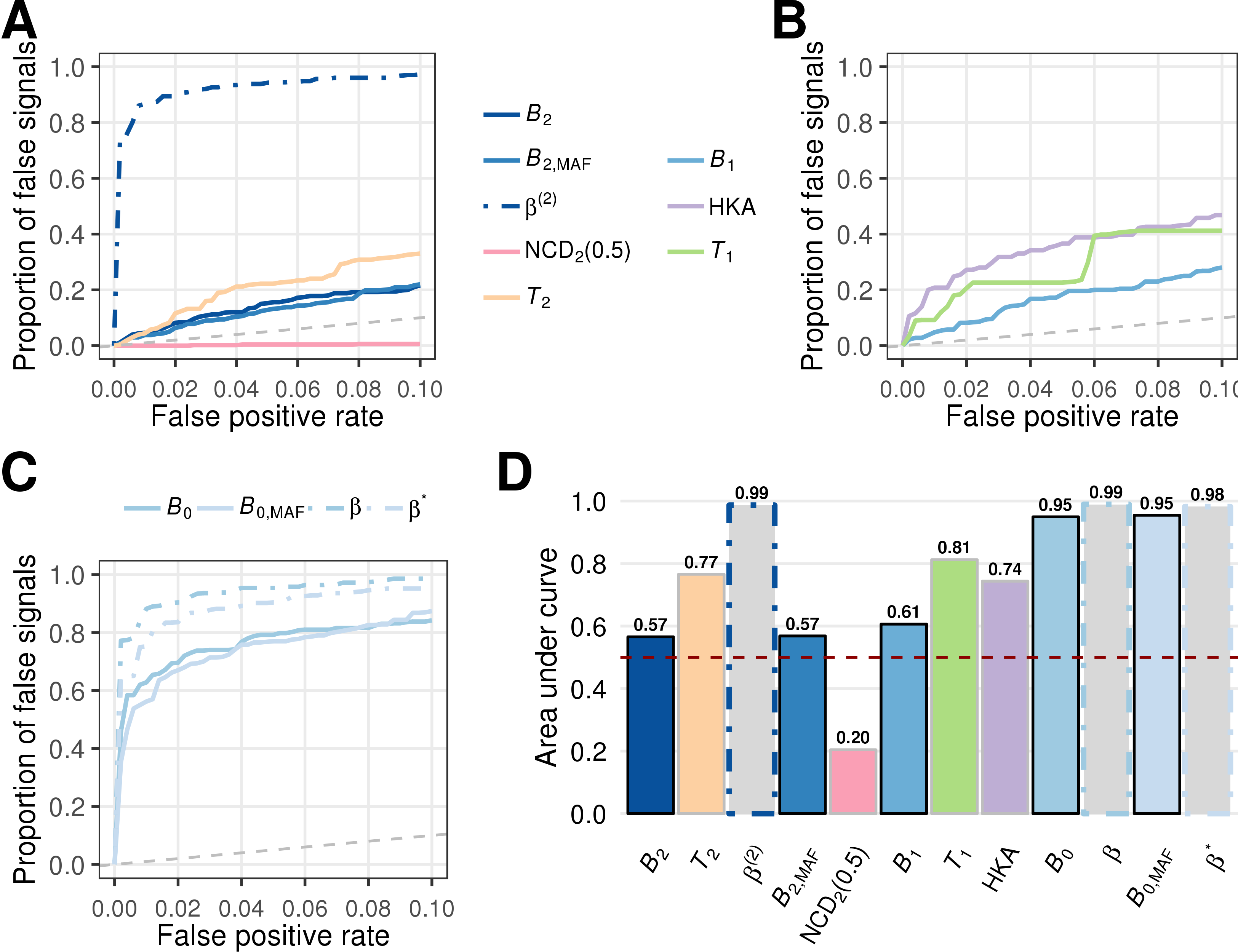

Supplement: msaa134_supplementary_data [file msaa134_supplementary_data.zip › BallerMix_final/figures/FigS54_splitView_HCG_5Mut_ROC+powerbar_matchB+stats.png]

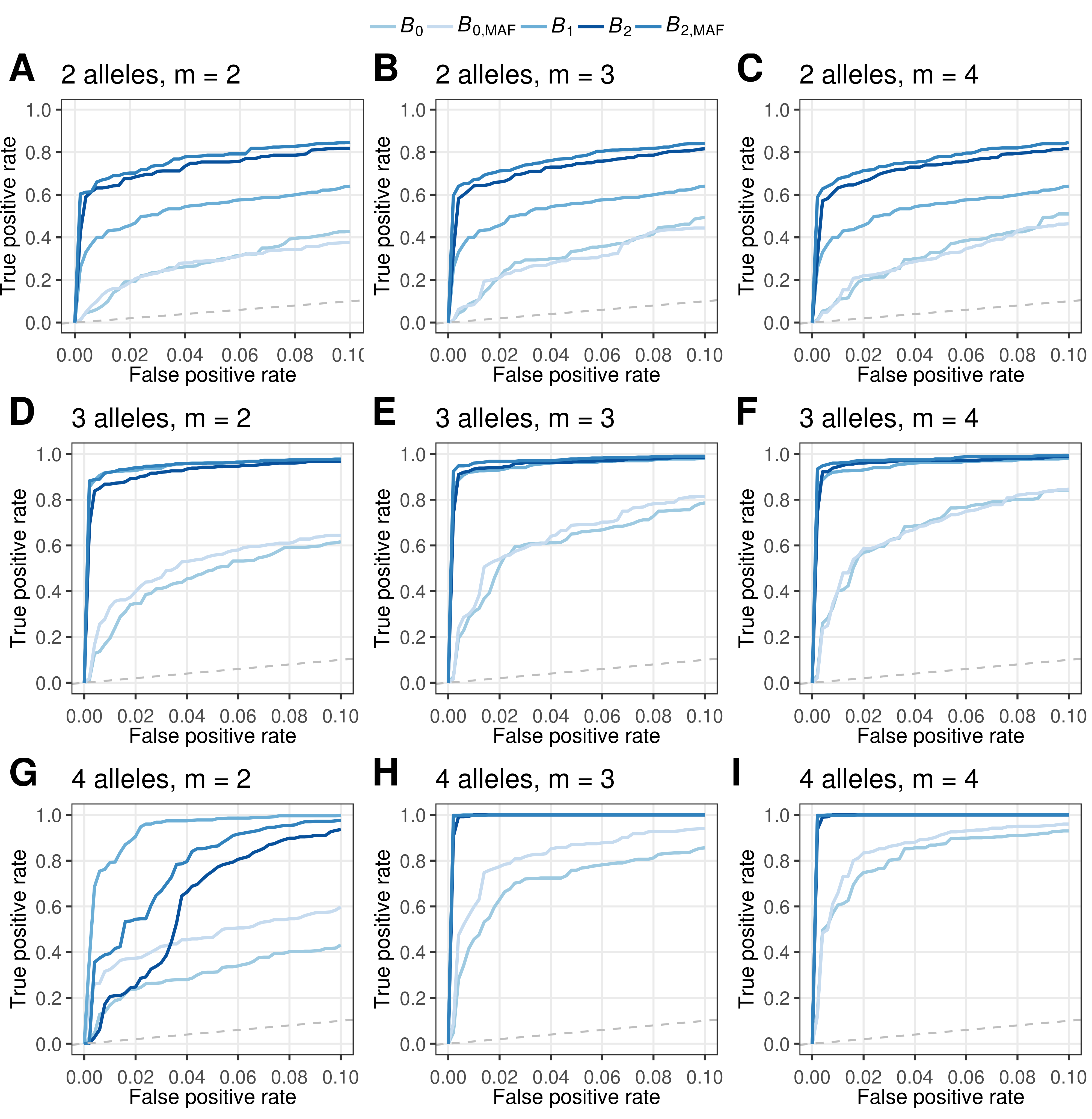

Supplement: msaa134_supplementary_data [file msaa134_supplementary_data.zip › BallerMix_final/figures/FigS55_newMultialleles_01ROCs_multiB_m2-real4.png]

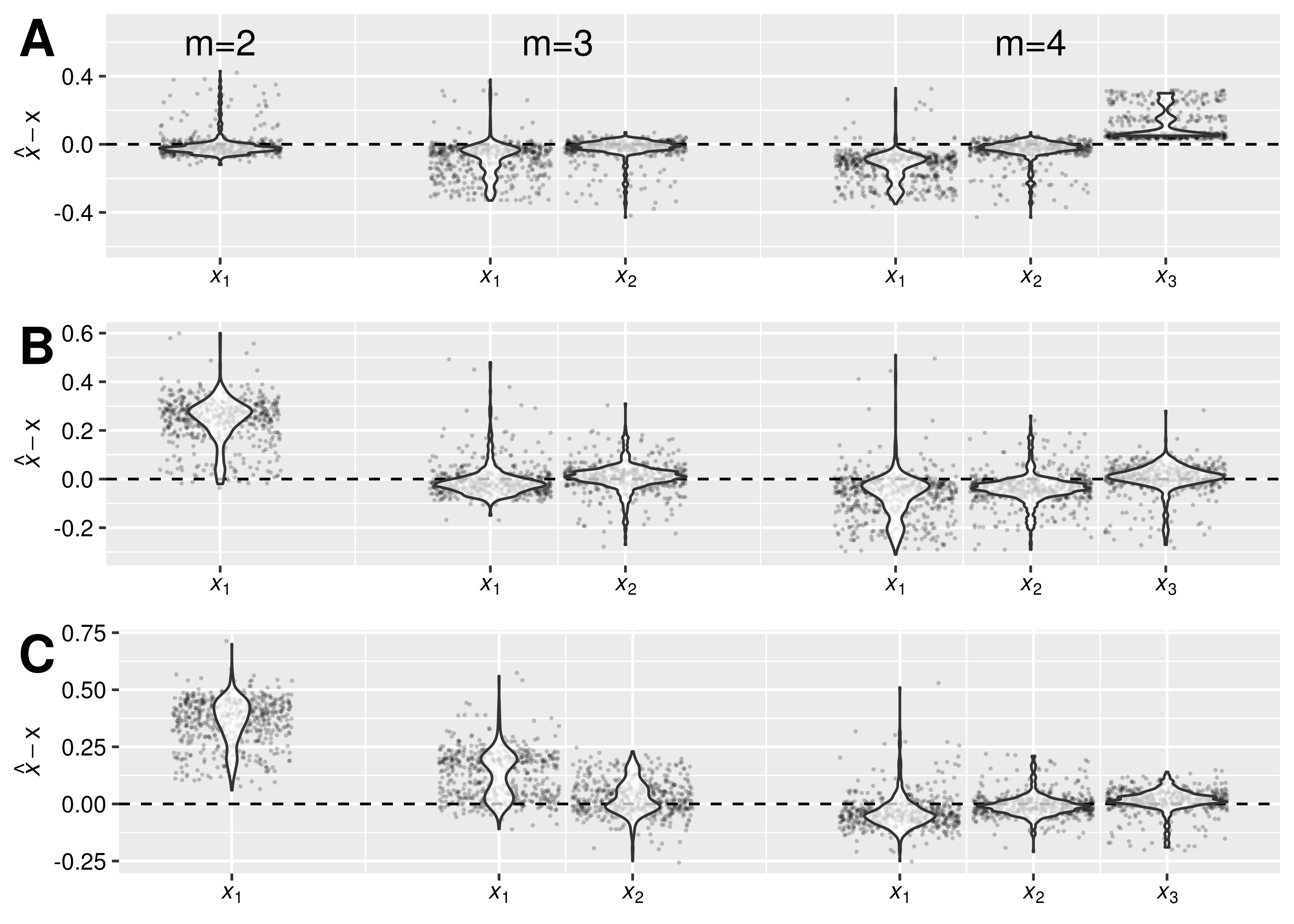

Supplement: msaa134_supplementary_data [file msaa134_supplementary_data.zip › BallerMix_final/figures/FigS56_newMultiAllele_freqDiff_violins.png]

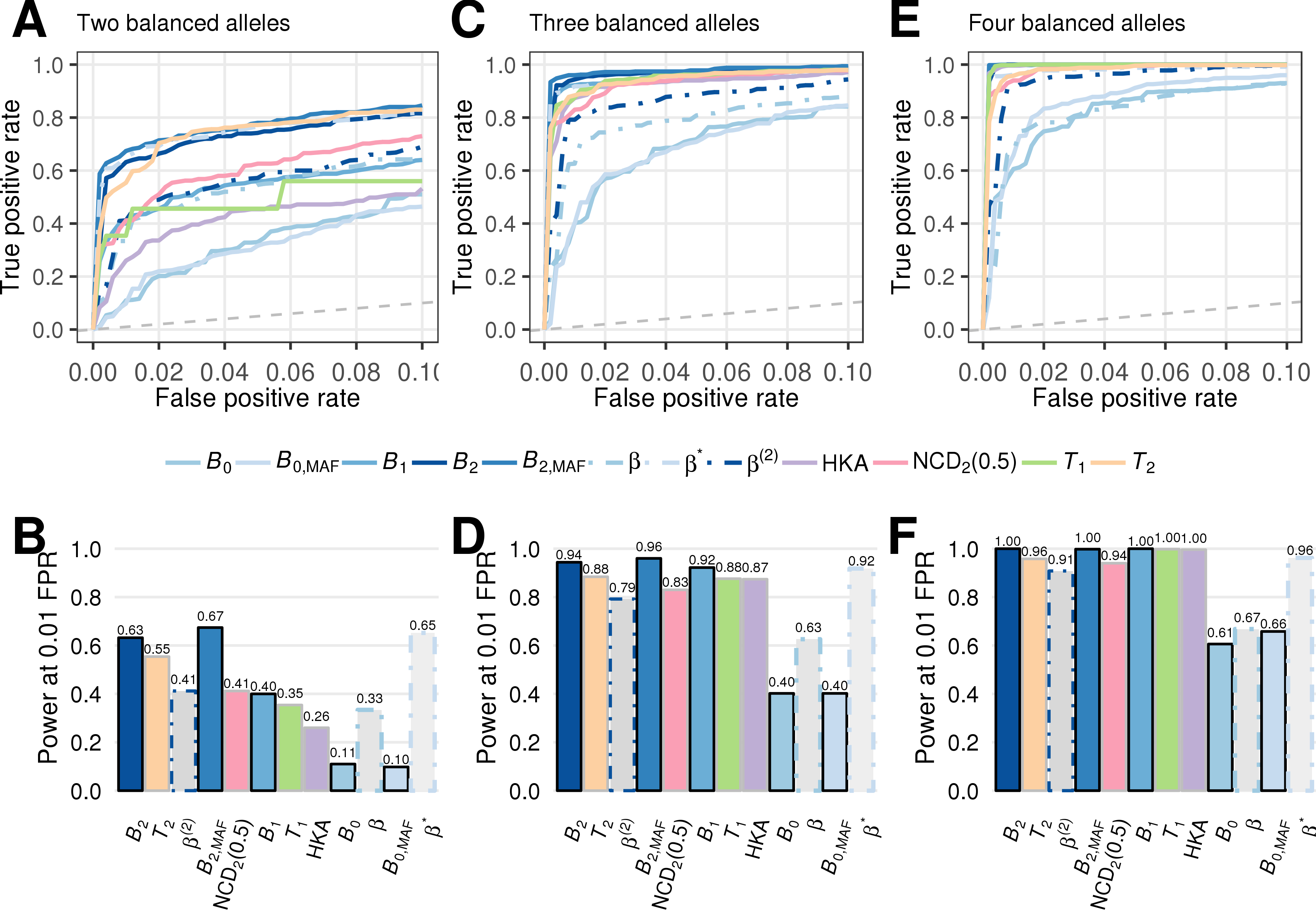

Supplement: msaa134_supplementary_data [file msaa134_supplementary_data.zip › BallerMix_final/figures/FigS57_newHCG_ACTG_234A_01ROC+powerbar_stats+B-m4.png]

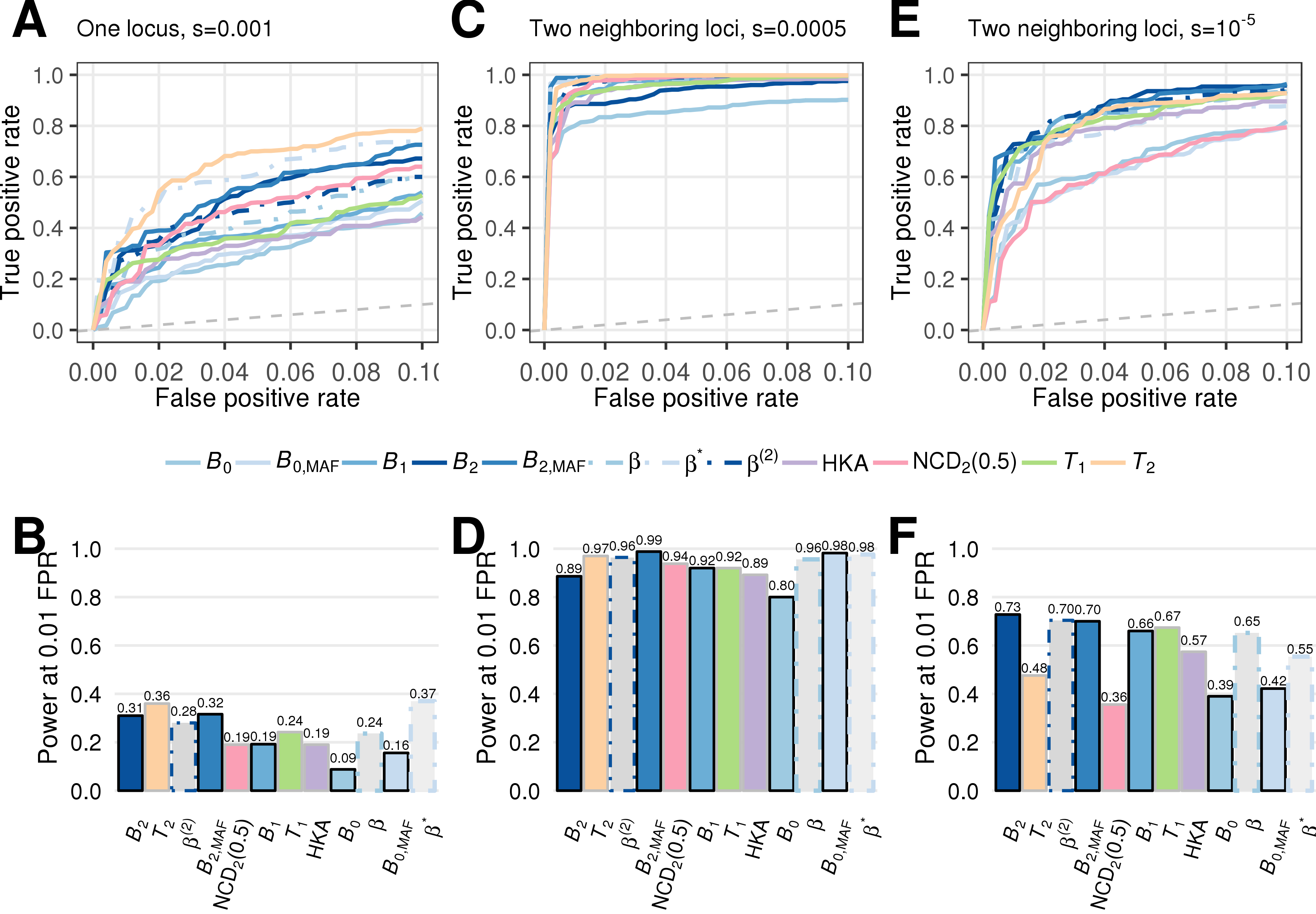

Supplement: msaa134_supplementary_data [file msaa134_supplementary_data.zip › BallerMix_final/figures/FigS58_newHCG_1-v-2loci_01ROC+powerbar_alphaB+stats.png]

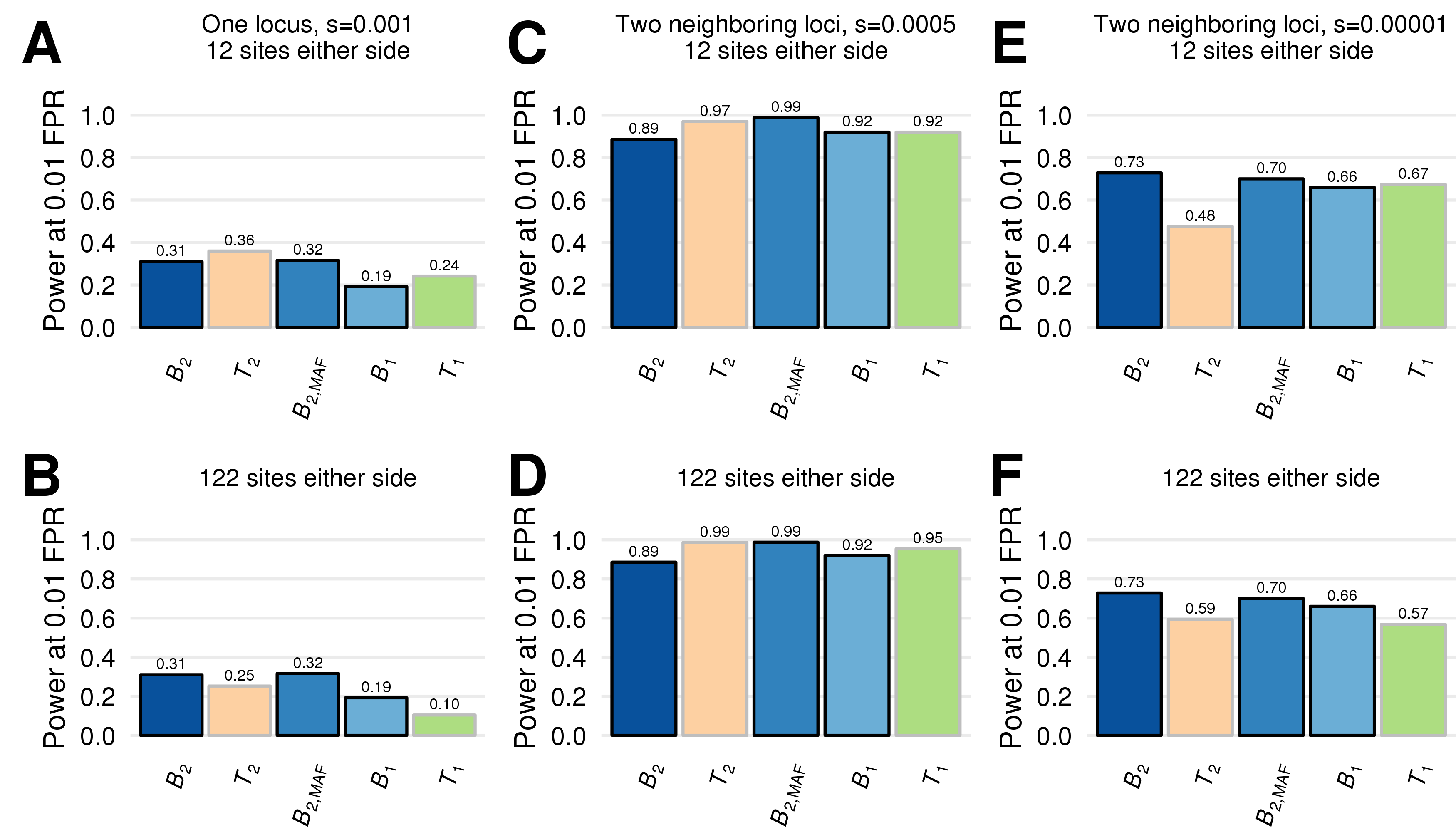

Supplement: msaa134_supplementary_data [file msaa134_supplementary_data.zip › BallerMix_final/figures/FigS59_HCG_1-v-2loci_powerBars_2xTs-Bs.png]

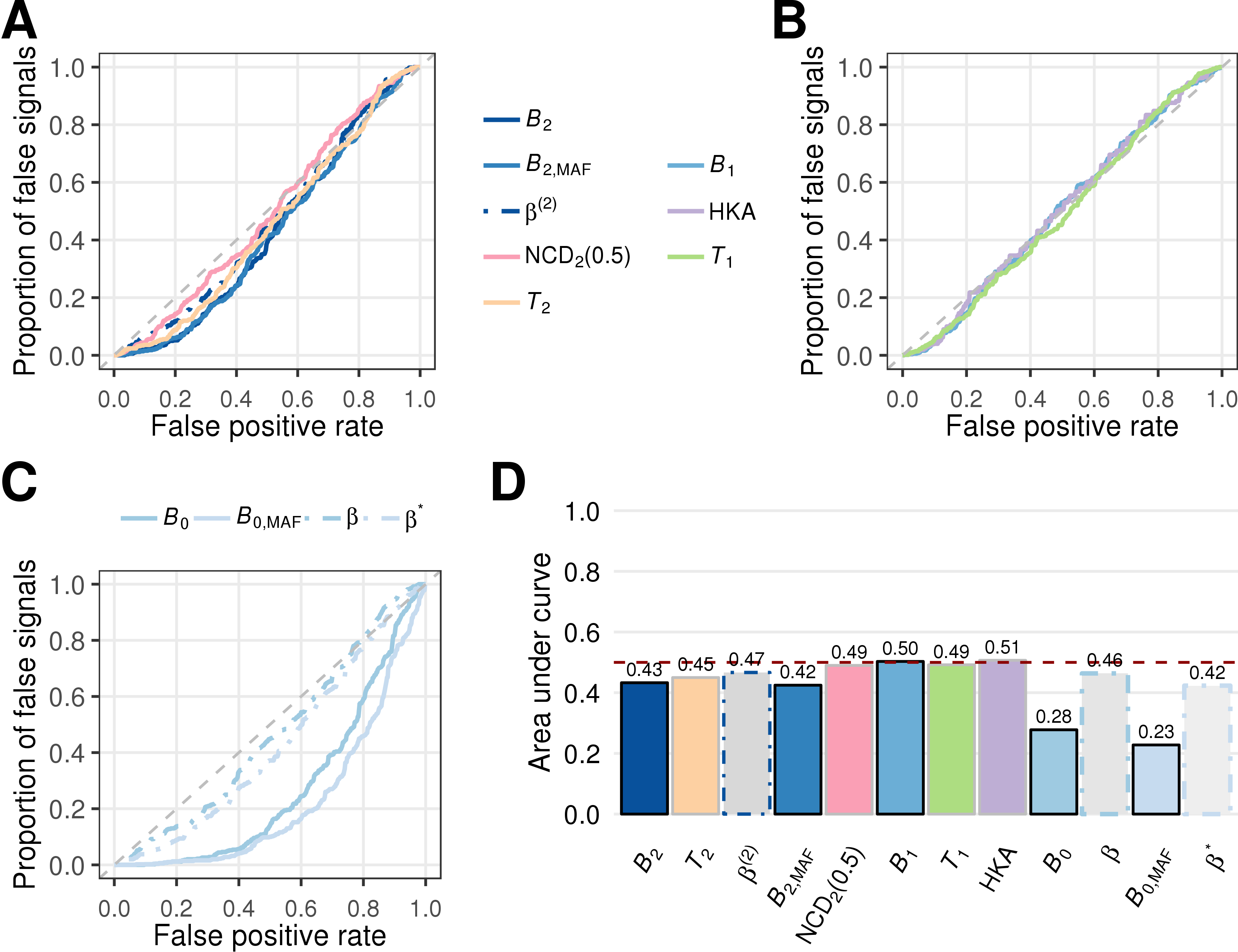

Supplement: msaa134_supplementary_data [file msaa134_supplementary_data.zip › BallerMix_final/figures/FigS6_splitView_HCG_10rec_Neut_ROC+powerbar_alphaB+stats.png]

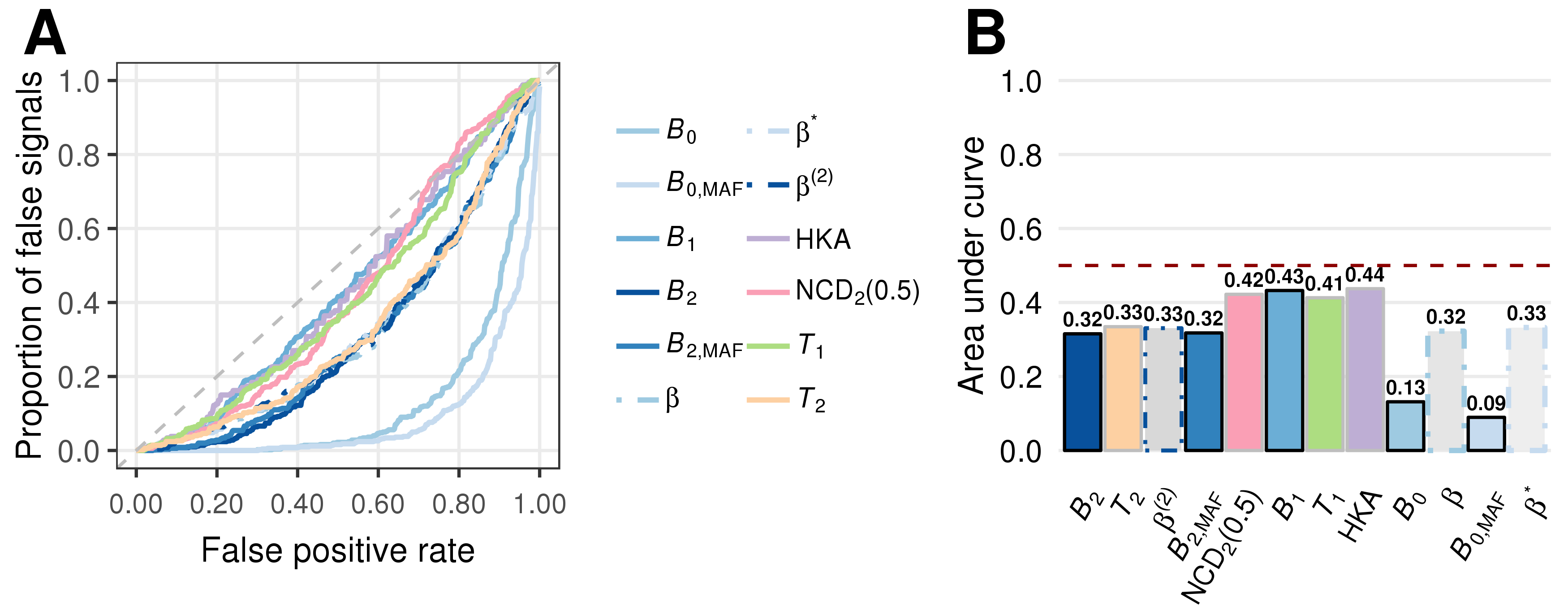

Supplement: msaa134_supplementary_data [file msaa134_supplementary_data.zip › BallerMix_final/figures/FigS7_HCG_100rec_Neut_ROC+powerbar_alphaB+stats.png]

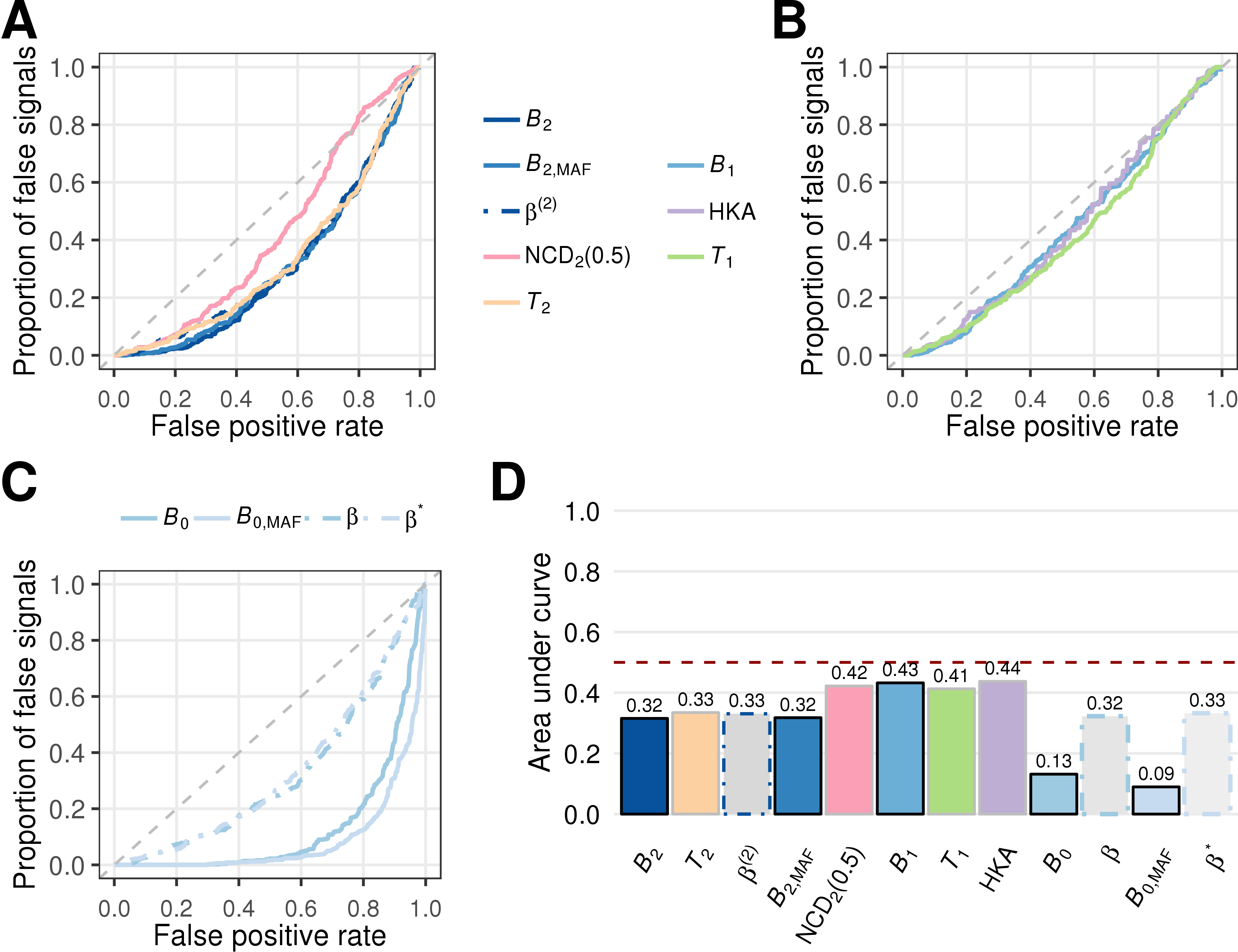

Supplement: msaa134_supplementary_data [file msaa134_supplementary_data.zip › BallerMix_final/figures/FigS8_splitView_HCG_100rec_Neut_ROC+powerbar_alphaB+stats.png]

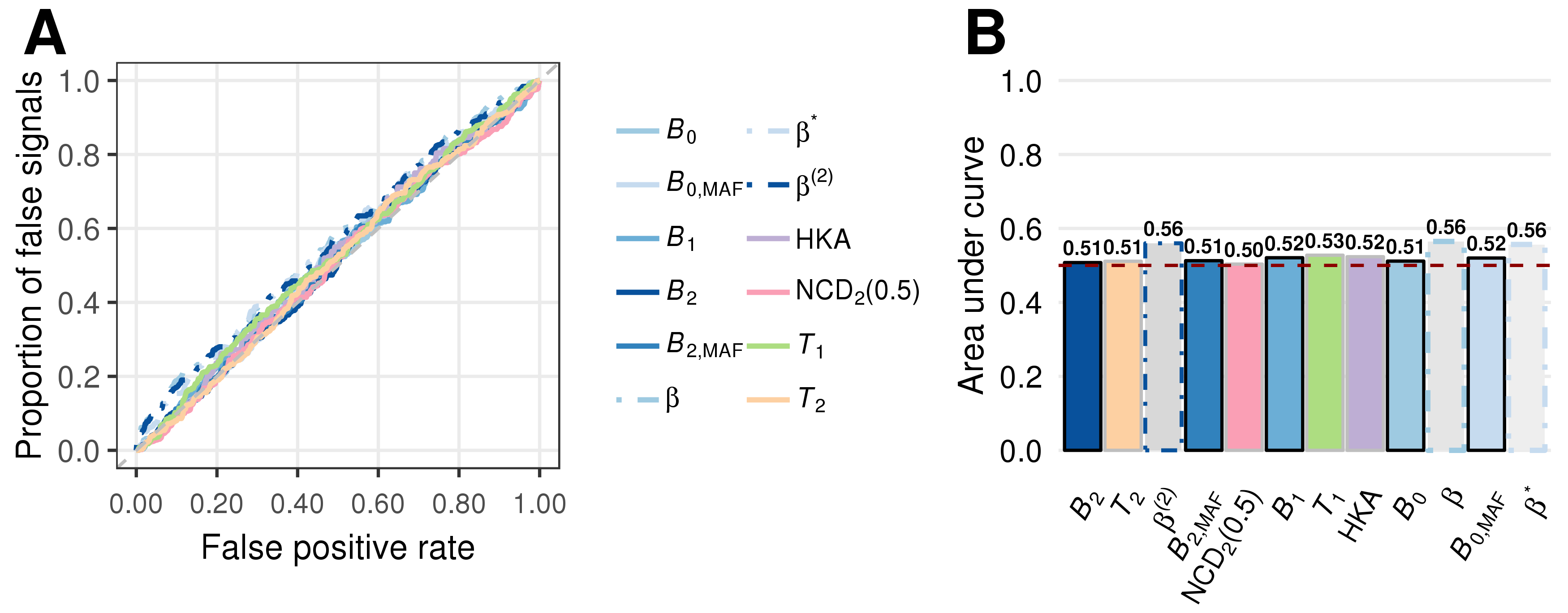

Supplement: msaa134_supplementary_data [file msaa134_supplementary_data.zip › BallerMix_final/figures/FigS9_HCG_localMut_ROC+powerbar_alphaB+stats.png]
